# Yoga versus education for Veterans with chronic low back pain: study protocol for a randomized controlled trial

**DOI:** 10.1186/s13063-016-1321-5

**Published:** 2016-04-29

**Authors:** Robert B. Saper, Chelsey M. Lemaster, A. Rani Elwy, Ruth Paris, Patricia M. Herman, Dorothy N. Plumb, Karen J. Sherman, Erik J. Groessl, Susan Lynch, Shihwe Wang, Janice Weinberg

**Affiliations:** Department of Family Medicine, Boston University School of Medicine and Boston Medical Center, Boston, MA USA; Center for Information Dissemination and Education Resources, VA Boston Healthcare System, Boston, MA USA; Department of Health Policy and Management, Boston University School of Public Health, Boston, MA USA; Boston University School of Social Work, Boston, MA USA; RAND Corporation, Santa Monica, CA USA; Center for Healthcare Organization and Implementation Research, Edith Nourse Rogers Memorial Veterans Hospital, Bedford, MA USA; Group Health Research Institute, Seattle, WA USA; Department of Epidemiology, University of Washington, Seattle, WA USA; VA San Diego Healthcare System, San Diego, CA USA; Department of Family and Preventive Medicine, University of California San Diego School of Medicine, San Diego, CA USA; There & Back Again, Inc., Wakefield, MA USA; Department of Biostatistics, Boston University School of Public Health, Boston, MA USA

**Keywords:** Veterans, Low back pain, Military families, Randomized controlled trial, Yoga, Cost-effectiveness

## Abstract

**Background:**

Chronic low back pain is the most frequent pain condition in Veterans and causes substantial suffering, decreased functional capacity, and lower quality of life. Symptoms of post-traumatic stress, depression, and mild traumatic brain injury are highly prevalent in Veterans with back pain. Yoga for low back pain has been demonstrated to be effective for civilians in randomized controlled trials. However, it is unknown if results from previously published trials generalize to military populations.

**Methods/design:**

This study is a parallel randomized controlled trial comparing yoga to education for 120 Veterans with chronic low back pain. Participants are Veterans ≥18 years old with low back pain present on at least half the days in the past six months and a self-reported average pain intensity in the previous week of ≥4 on a 0–10 scale. The 24-week study has an initial 12-week intervention period, where participants are randomized equally into (1) a standardized weekly group yoga class with home practice or (2) education delivered with a self-care book. Primary outcome measures are change at 12 weeks in low back pain intensity measured by the Defense and Veterans Pain Rating Scale (0–10) and back-related function using the 23-point Roland Morris Disability Questionnaire. In the subsequent 12-week follow-up period, yoga participants are encouraged to continue home yoga practice and education participants continue following recommendations from the book. Qualitative interviews with Veterans in the yoga group and their partners explore the impact of chronic low back pain and yoga on family relationships. We also assess cost-effectiveness from three perspectives: the Veteran, the Veterans Health Administration, and society using electronic medical records, self-reported cost data, and study records.

**Discussion:**

This study will help determine if yoga can become an effective treatment for Veterans with chronic low back pain and psychological comorbidities.

**Trial Registration:**

ClinicalTrials.gov: NCT02224183

**Electronic supplementary material:**

The online version of this article (doi:10.1186/s13063-016-1321-5) contains supplementary material, which is available to authorized users.

## Background

### Low back pain in Veterans

Musculoskeletal pain conditions are the most commonly diagnosed medical problems among the more than two million Veterans from Operations Enduring Freedom, Iraqi Freedom, and New Dawn (OEF/OIF/OND), far surpassing other medical and mental health disorders [1–3]. In a study of 91,000 Veterans receiving care from the Veterans Health Administration (VHA), 43 % reported having any pain, and 63 % of these reported moderate to severe pain [4]. Eighty percent of VHA visits include pain-related complaints [5]. Overreliance on opioids is common and can lead to adverse effects ranging from sedation to dependence, addiction, and death due to overdose [6, 7]. Among pain conditions, chronic low back pain (cLBP) is the most frequent [4] and causes substantial suffering, decreased functional capacity [8], and lower quality of life [9, 10]. The direct costs of chronic pain in the U.S. are estimated to be $100 billion or greater annually [3]. Among Veterans, back pain is a leading cause of disability [11, 12].

Psychological distress, back pain, and disability are strongly correlated. Symptoms of post-traumatic stress [13], depression [6], and traumatic brain injury [1] are highly prevalent in Veterans with cLBP [14]. Among Veterans receiving care from VHA Polytrauma Network sites, 42 % had the triad of chronic pain, post-traumatic stress symptoms (PTSS), and persistent post-concussive symptoms (traumatic brain injury) [7, 15]. Low pain self-efficacy and maladaptive pain coping behaviors (e.g., catastrophizing [16], fear avoidance [17], and substance abuse [18]) compound back pain recovery. Moreover, stigma and other barriers often prevent Veterans from seeking mental health care (e.g., cognitive therapy for PTSS), leading to additional barriers to back pain recovery [19, 20].

According to the Institute of Medicine, “Military family members are an important part of the readiness and well-being of the military force.” [21] Several studies on the impact of chronic pain on family life in civilians have shown poorer couple agreement [22] and lower marital satisfaction [23]. Injured service members and their partners must adapt to the injury’s physical and emotional sequelae, including chronic pain, post-traumatic stress disorder (PTSD), and depression [24, 25]. Partners of injured Veterans experience elevated distress levels along with the greater burden of caregiving for the Veteran, household, and children [26, 27]. Although chronic pain in Veterans is a significant and growing problem [2], its impact on Veteran families has rarely been studied.

### Yoga for low back pain

Yoga is increasingly common with more than 8 % of U.S. adults reporting use in 2012 [28–31]. A 2005 randomized controlled trial (RCT) found a moderate benefit of yoga for improving back-related function in civilian adults with cLBP [32]. This prompted the VHA, American College of Physicians, and American Pain Society to list yoga in clinical practice guidelines as an evidence-based treatment for cLBP [33]. Subsequently, four moderate to large RCTs (*n* = 90–313) [34–37] and six smaller (*n* = 20–60) [38–43] RCTs have also shown yoga to be effective for reducing pain and improving function in civilian adults with cLBP. Meta-analyses support these conclusions [44–46]. Several yoga-cLBP studies found associated psychological benefits in mood and self-efficacy [34, 36, 42, 43, 47]. Yoga research on psychological health is growing, showing promising evidence for benefit in depression [48–56], anxiety [56–59], and insomnia [60, 61]. Yoga classes can also increase social connectedness and spirituality [62].

The 2010 Army Surgeon General Pain Management Task Force Report highlighted that pain is an enormous problem facing Veterans and military families [63]. The report emphasized the importance of partnerships to develop an “integrative and interdisciplinary approach” to pain management, including incorporating “integrative and alternative therapeutic modalities into a patient-centered plan of care” [63]. The report emphasized, “Pain cannot be managed without addressing its relationship to stress,” and shifting from passive to more active treatments “improves the outcome, decreases provider dependent care, and empowers the patient with a sense of control over his or her condition.” The Task Force identified yoga as one of several “Tier 1” complementary modalities as priorities for DoD-VA research and possible integration. A recent VHA report mapping the evidence for yoga on high-impact conditions supported the potential benefit for adults with cLBP [46]. Yoga is increasingly offered to military personnel and Veterans by nonprofit yoga organizations and yoga studios. Among VHA PTSD-specific programs, 29 % offer yoga [64].

Whether results from previously published civilian yoga-cLBP trials generalize to Veteran populations is unknown. In contrast to participants in civilian studies, Veterans with cLBP are more likely to be men with different mechanisms of injury (e.g., direct combat-related trauma or severe non-combat back loading from carrying heavy gear), greater pain severity and disability, and more serious comorbid psychological symptoms [1, 4, 13, 15]. Only two small reports of yoga for cLBP in Veteran settings have been published [65, 66]. These uncontrolled yoga-cLBP clinical programs in Veterans showed promising improvements in pain and depression symptoms. Despite enthusiasm for offering yoga to Veterans, yoga instruction to date in the VHA is not well standardized or widely implemented. More importantly, there is little strong evidence for yoga’s effect on PTSS, depression, or other psychosocial problems. Thus, structured and reproducible yoga protocols for cLBP need to first be adapted to the unique needs of Veterans [3] and then must be rigorously tested for clinical and cost-effectiveness in this population.

### Specific aims

This study will determine if yoga can become a safe, clinically effective, cost-effective, and scalable nonpharmacologic approach to address the physical and psychosocial dimensions of cLBP in Veterans [67]. This study is a 24-week two-arm randomized controlled trial for 120 Veterans with cLBP with four specific aims:

1. Primary aim – to determine the effectiveness of a structured, reproducible 12-week series of hatha yoga classes, supplemented with home practice, compared to an education control group, for decreasing pain intensity and improving back-related function
2. Secondary aim – to evaluate the effectiveness of yoga compared to education for improving PTSS and other psychosocial outcomes, including depression, anxiety, self-efficacy, and coping
3. Secondary aim – to evaluate the cost-effectiveness of yoga for cLBP at 12 weeks and 24 weeks from three perspectives: the Veteran, the Veterans Health Administration, and society
4. Exploratory aim – to explore the effect of cLBP on family relationships, as well as yoga’s impact on the Veteran, family, and family functioning

The results of this study have the potential to impact the approach and management of cLBP according to the Army Surgeon General Pain Management Task Force vision, i.e., having a more integrative and interdisciplinary focus on active self-care approaches that empower Veterans to have greater control of their condition [63].

## Methods/design

### Study design

**Fig. 1:**
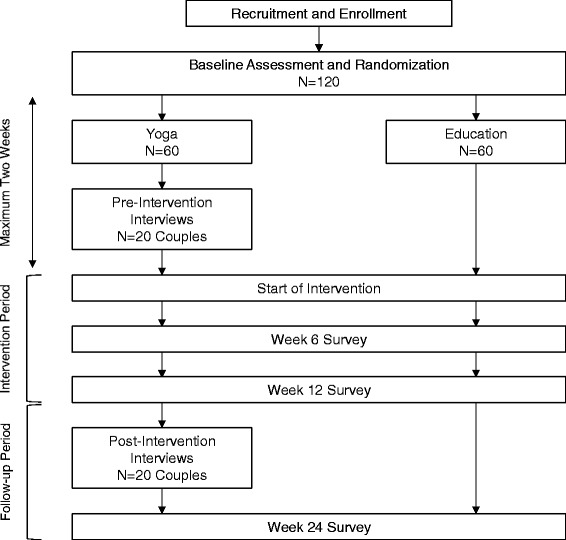
Study flow diagram. The study will enroll and randomize 120 Veterans equally into yoga and education groups. Qualitative interviews will take place with 20 Veterans in the yoga arm and their partners before and after the 12-week intervention period. Interventions are followed by a 12-week follow-up period. Data collection takes place at baseline (prior to randomization), 6 weeks, 12 weeks, and 24 weeks

The trial is a single-blinded 24-week RCT and is divided into two phases: an initial 12-week intervention period followed by a 12-week follow-up period (Fig. 1). Prior to beginning the intervention, Veterans are randomized in a 1:1 ratio into (1) a 12-week series of structured weekly hatha yoga classes supplemented by home yoga practice or (2) education using a comprehensive back pain self-management book, *The Back Pain Helpbook*, supplemented by newsletters that highlight key content [68]. Block-stratified randomization is being used to ensure similar numbers of Veterans in both treatment groups who are ≤45 years old, experience PTSS, and have long-term partners (spouse, live-in partner, or in a live-out committed relationship). During the 12-week follow-up period, yoga participants are encouraged to continue home practice. Those in the education group are encouraged to continue following recommendations from the book.

**Fig. 2:**
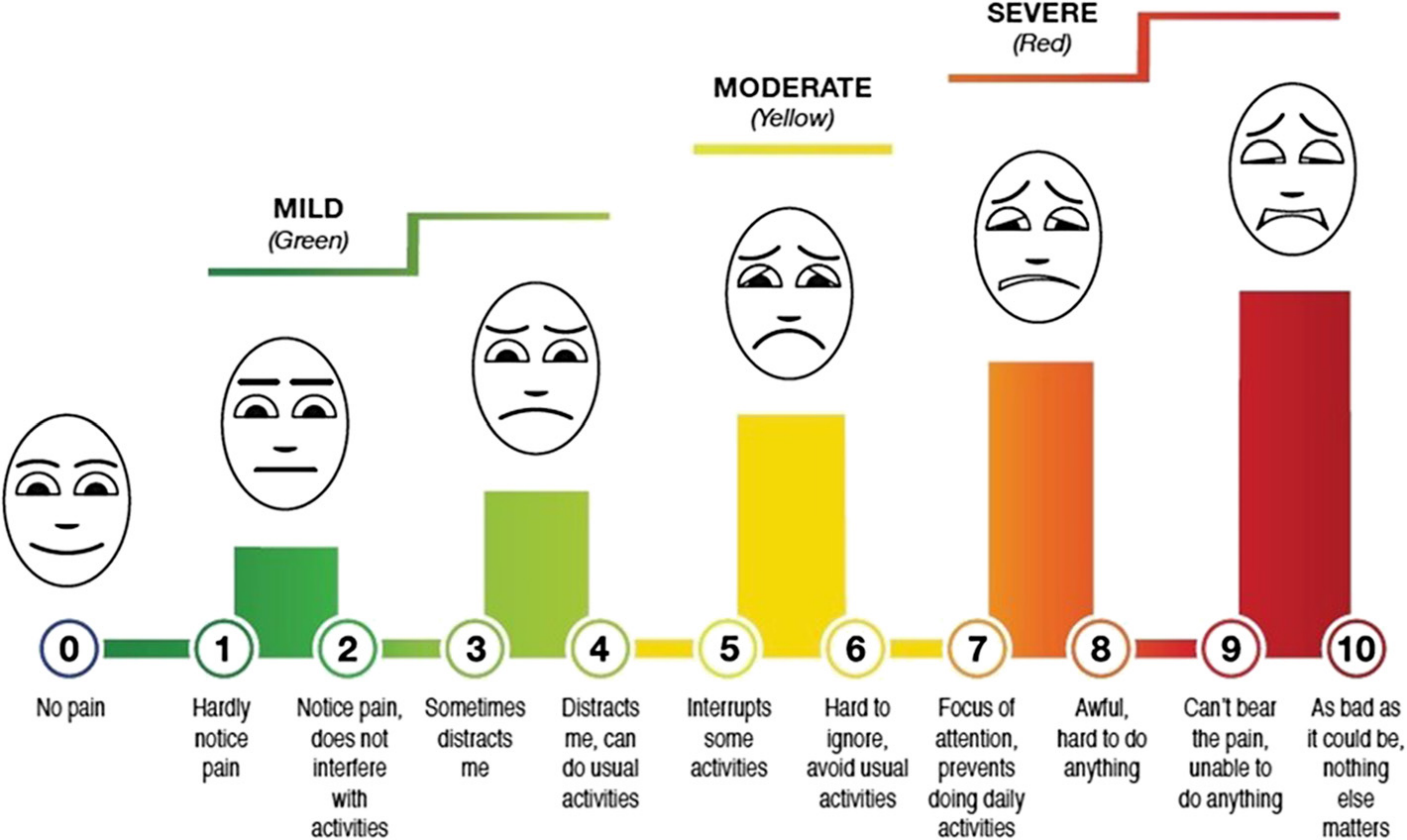
Defense and Veterans Pain Rating Scale. The Defense and Veterans Pain Rating Scale (DVPRS), developed by the Army Surgeon General Pain Management Task Force, is a validated patient-reported pain assessment tool [69, 70]. This integrated graphic tool incorporates a numerical rating scale, descriptors for each numerical rating, “traffic light” color-coding, and a faces scale

The study co-primary endpoints are average pain intensity over the previous 7 days measured by the Defense and Veterans Pain Rating Scale (DVPRS; Fig. 2) [69, 70] and back-related function measured by the Roland Morris Disability Questionnaire (RMDQ; scores range from 0–23 with higher scores reflecting poorer function) [71, 72]. We hypothesize that (1) 12 weeks of weekly yoga will be more effective than education for improving low back pain intensity and back-related function and (2) yoga will be superior compared to education for reducing PTSS in the subset of participants with comorbid PTSS at baseline.

Secondary outcomes include pain medication use, health-related quality of life, and a range of psychological measures targeting key mental health symptoms facing Veterans. Other secondary outcomes include the minimal dataset recommended by the Report of the NIH Task Force on Research Standards for Chronic Low Back Pain [73]. Cost-effectiveness analyses will be performed from the perspective of the Veteran, VHA, and society. To gain a greater understanding of the impact of cLBP on the Veteran and family, we are conducting in-depth interviews with a subset of 20 Veterans in the yoga arm and their partners before and after the yoga intervention.

The Boston University Medical Campus and Bedford VA Institutional Review Boards (IRBs) approved this study.

### Sample size

We assume a two-sided α = 0.05. This is justifiable because, although there are two primary outcomes, they are not independent. We use 2.5 as the standard deviation for the mean pain change score between baseline and 12 weeks, which is what was found in previous yoga-cLBP studies [37]. We also assume a 20 % dropout rate based on previous studies [37]. Thus, a sample size of 120 participants randomized to yoga vs. education provides 97 % power to demonstrate a minimal clinically significant difference in pain score of 2.0 [74]. For the RMDQ change score, if we assume a standard deviation of 5.0 [35, 37] and a minimal clinically significant difference of 3.0 [75], we have 82 % power to show a statistically significant difference between the two groups. Finally, if we assume that 50 % of the study participants with cLBP have a baseline PTSD CheckList – Civilian Version (PCL-C) score ≥30 and a standard deviation for the PCL-C change score of 12 [76, 77], there is 80 % power to demonstrate a minimal clinically meaningful difference in PCL-C of 10 points.

### Inclusion and exclusion criteria

Table 1 lists the eligibility criteria and the corresponding rationale. Inclusion criteria are: ≥18 years old; low back pain present on at least half the days in the past six months; average back pain intensity ≥4 for the previous seven days on a 0–10 numerical rating scale; and Veteran of the U.S. military. Exclusion criteria include: back pain caused by inflammatory conditions (e.g., ankylosing spondylitis), malignancy, fracture, or infection; practiced yoga regularly (more than twice per month) or read *The Back Pain Helpbook* [68] within the past six months; new back pain therapies started in the last month or planned to begin in the next three months; plans to leave the area in the next six months; progressive or severe neurological deficits; lack of consent; unwillingness to be randomized; or any severe medical or psychiatric condition that, in the principal investigator’s judgment, would make participation not possible or unsafe.

**Table 1:** Eligibility criteria

Inclusion Criteria	Rationale
≥18 years old	Chronic low back pain in children results from different causes than those we are studying
Current low back pain present on at least half the days in past 6 months	Condition studied is specifically chronic
Mean low back pain intensity for the previous week ≥4 on a 0 to 10 numerical rating scale (0 = no pain to 10 = worst possible pain)	Back pain severe enough to detect improvement and prevent against floor effects
Veteran of the U.S. military	Defined target population
English fluency sufficient to follow treatment instructions and answer survey questions	Fully informed consent and data collection
Exclusion criteria	Rationale
Significant participation in yoga in the previous 6 months	Possible bias due to current or recent intervention users
Read *The Back Pain Helpbook* in the previous 6 months	
New back pain treatments started within the previous month or anticipated to begin in the next 3 months	
Ankylosing spondylitis	Back pain due to, or possibly result of, specific disease/condition(s)
Active or recent malignancy	
Fracture	
Infection in or around the spine	
Pregnancy	
Other severe disabling chronic medical and/or psychiatric comorbidities deemed by the principal investigator on a case-by-case basis to prevent safe and/or adequate participation in the study (e.g., severe disabling heart failure or lung disease, psychosis)	Comorbid condition(s) pose inappropriate risk to safety or preclude compliance with interventions
Severe or progressive neurological deficits	
Plans to move out of the area in the next 6 months	Known barrier to full study participation
Lack of consent	Research policy
Unwilling to be randomized	

### Recruitment, enrollment, and retention

A multipronged strategy for recruitment is being used:

1. Designate “study champions” within different Bedford VA Hospital clinical areas. The champion publicizes and encourages recruitment within their area through presentations to clinicians and staff during regularly scheduled meetings. Clinical site champions ensure all relevant clinicians and staff are aware of the study and provide flyers with information on the study to Veterans when discussing treatment options for pain at the time of encounter/appointment.
2. Identify in the electronic health records Veterans seen in the previous two years at the Bedford VA Hospital who have back pain on their problem list. Targeted letters signed by clinical site champions, flyers, and opt-out cards are mailed to these Veterans inviting them to join the study.
3. Place study flyers and brochures in exam rooms, waiting rooms, and public areas throughout the Bedford VA Hospital and surrounding community.
4. Use Veteran networks established by co-investigators and stakeholder organizations to advertise the study.

The screening and enrollment process involves (1) eligibility screening and (2) an in-person consent meeting with study staff. Screening for eligibility takes place by telephone using a 12-item questionnaire and is preceded by verbal consent. If the Veteran appears to be eligible based on the telephone screening, he or she is asked to meet in person with research staff for a 30-minute informational meeting. The meeting includes a detailed discussion of study procedures, interventions, risks and benefits of participation, confidentiality, and expectations of participants. For those interested in joining the study, informed consent is obtained both verbally and in writing.

Research staff employ a Plan–Do–Study–Act quality improvement process [78] on a weekly basis to continuously monitor and improve recruitment and retention. Recruitment statistics for the previous week and study-to-date are discussed weekly using a standardized format. Attendance rates for informational consent meetings, yoga classes, and survey visits are also reviewed regularly. Based on these data, the team discusses updates to recruitment and retention strategies if necessary.

### Randomization

The baseline survey is typically completed within 45 days after eligibility screening. If the period since screening exceeds 45 days, eligibility is re-assessed and verified prior to the baseline survey. After completion of the baseline survey, the participant is randomized in a 1:1 ratio to yoga or education using StudyTRAX™ (Macon, GA), a HIPAA-compliant study management software platform. Permuted block randomization with varying block sizes of two or four is used. Stratification is done on three dichotomous variables to assure similar distribution between the two groups:

1. Age (≤45, >45 years). Back pain in older Veterans may be structurally different. Furthermore, age may be a confounding variable to the response to yoga.
2. PCL-C score (<30, ≥30). These cut-offs will help assure equivalent numbers of Veterans with PTSS in both treatment arms in order to compare the effectiveness of the interventions for reducing comorbid PTSS. PTSS may also be an effect modifier for yoga and cLBP.
3. Partnered (yes/no). A partnered Veteran is defined as one who reports having a cohabitating or long-term committed partner/spouse. This helps assure adequate numbers of partnered Veterans in the yoga group available for qualitative interviews.

### Study interventions

The study interventions start within two weeks of randomization to allow adequate time for pre-intervention qualitative interviews. All interventions, related supplies, and transportation to study visits are provided at no cost to participants. Veterans in both treatment arms can continue to see their providers and receive routine care including medication.

#### Hatha yoga

The structured and manualized yoga protocol for cLBP was initially developed by an expert panel led by the principal investigator (RBS) in 2007. It was used in a pilot study of 30 predominantly minority low-income civilian participants [40]. The protocol was further refined in 2012 for a yoga dosing study comparing one vs. two yoga classes per week [37]. This protocol was again adapted for use in a large study of 320 civilians that compared yoga, physical therapy, and education [79]. In order to adapt the yoga protocol to a Veteran population, a panel of 11 experts and stakeholders convened in November 2014. Attendees included Veterans, military leaders, yoga teachers, representatives from organizations focused on providing mind-body therapies to Veterans (There & Back Again^©^, Warriors at Ease^©^), and researchers with expertise in yoga, low back pain, and the VHA. Prior to the meeting, relevant materials (previous yoga for back pain protocols and literature) were distributed for the attendees to review. The full-day in-person meeting included presentations and discussions of unique aspects to teaching yoga to Veterans, class format, segment themes, relaxation exercises, breathing exercises, and specific postures. Based on this input, iterative drafts of the yoga instructor documents were revised and circulated until broad consensus was obtained on the final version.

Yoga classes contain no more than eight to ten Veterans and two yoga teachers to assure a low Veteran-to-instructor ratio (no more than five Veterans to one teacher). This arrangement maximizes effectiveness and safety, and allows flexibility in yoga teacher scheduling based on the study’s needs. Veterans are asked to attend one 75-minute class per week and receive reminder phone calls the day before each scheduled class. Attendance is taken by research staff. We provide $10 travel compensation for each yoga session. Mats and props are provided. Each class includes a yoga breathing exercise (*pranayama*), discussion of yoga philosophical principles, yoga postures (*asanas*), and a relaxation exercise (*svasana*). Veterans are frequently advised to proceed carefully and slowly. The difficulty of postures increases throughout the 12 weeks (Table 2). The 12-week intervention period is divided into four 3-week segments, titled Opening to Something New, Listening to Your Back, Engaging Your Power, and Bringing it Home. Posture variations, modifications, and various aids (e.g., block, chair, strap, wall) are built into the protocol to accommodate a range of abilities. Yoga participants are encouraged to practice at home for 30 minutes on days when they do not have class. To help Veterans practice at home, they receive a guidebook describing and depicting the protocol (see Additional file 1), yoga mat, strap, two blocks, and home practice instructional videos (see Additional file 2-5). Home practice videos portray Veterans in an introductory video describing the value of yoga practice in their own lives, two 30-minute videos that follow the same structure as group yoga classes, and one 20-minute extended breathing and relaxation video. Veterans are given a link to view the home practice videos online or, if requested, they receive a take-home DVD.

**Table 2:** Twelve-week standardized hatha yoga protocol

Yoga posture (*asana)* or class component	Classes incorporating component by segment			
	Segment 1	Segment 2	Segment 3	Segment 4
	Weeks 1–3	Weeks 4–6	Weeks 7–9	Weeks 10–12
	Opening to Something New	Listening to Your Back	Engaging Your Power	Bringing it Home
Breathing exercise	✓	✓	✓	✓
Knees to chest pose^*^	✓	✓	✓	✓
Knees together twist pose	✓	✓	✓	✓
Pelvic tilt pose^*^	✓			
Big toe pose^*^		✓	✓	✓
Cat and cow pose*	✓		✓	
Wheel pose^*^	✓		✓	
Chair pose^*^	✓	✓	✓	
Shoulder openers^*^	✓	✓	✓	✓
Crescent moon pose^*^	✓	✓		✓
Mountain pose^*^	✓	✓	✓	✓
Child's pose^*^	✓	✓	✓	✓
Locust pose	✓	✓	✓	
Sphinx pose^*^	✓	✓		
Cobra pose^*^		✓		✓
Plank pose^*^			✓	✓
Side plank pose			✓	✓
Downward facing dog^*^	✓	✓	✓	✓
Triangle pose^*^ (with and without wall)	✓	✓	✓	✓
Forward bend pose^*^ (with and without wall)	✓			✓
Warrior I pose^*^	✓		✓	✓
Warrior II pose^*^		✓	✓	✓
Wide-leg bend pose^*^ (with and without wall)		✓	✓	
Side hip strengtheners		✓	✓	✓
Eye of the needle pose^*^		✓	✓	✓
Extended leg pose^*^		✓		✓
Baby dancer pose^*^ (modified at the wall)				✓
Bridge pose^*^ (with and without support)	✓	✓	✓	✓
Reclining cobbler pose		✓	✓	✓
Reclining chest opener pose		✓	✓	✓
Legs up the wall pose			✓	✓
Relaxation exercise	✓	✓	✓	✓

*Pose has optional chair modifications described in teacher and participant manuals

All yoga teachers underwent training prior to teaching study classes. A 6-hour in-person training covered the specific yoga practices suggested (e.g., postures, breathing exercises) and information on how to teach a culturally-sensitive yoga class to Veterans. Online webinars provided by Warriors at Ease^©^ included four 90-minute courses on cultural sensitivity, common injuries or conditions in Veterans, the role of the yoga teacher(s), and managing emotional reactions. All teachers received and follow an instructor manual (see Additional file 6), which contains detailed information on the segment themes, week-by-week yoga class structure, teaching individual postures with or without modifications, leading breathing exercises, and guiding Veterans through relaxation. To assess fidelity to the protocol, researchers observe 10 % of classes using a protocol fidelity checklist.

#### Education

*The Back Pain Helpbook* [68] is given to individuals randomized to the education group. This book has been used successfully in previous cLBP studies for educational purposes [32, 80]. The book includes information on how back pain is influenced by posture, emotions, fear avoidance behaviors, and social support. It also encourages pain self-management strategies such as mind-body relaxation techniques, exercise programs, and medications. In addition, Veterans receive a handout after randomization suggesting specific chapters to read throughout the intervention period and brief mailed newsletters every three weeks that highlight main points from the recommended chapters (see Additional file 7). Check-in calls, which are proceeded by a newsletter mailing, are conducted by research staff every three weeks to assess progress and reinforce study retention efforts.

### Data collection

Outcome data are collected at baseline, 6 weeks, 12 weeks, and 24 weeks. Participants in both treatment arms complete paper surveys in person and receive cash honoraria after the completion of each survey. We give $25 at baseline, 6 weeks, and 12 weeks. At the final follow-up at 24 weeks, participants receive $50 after completion of their survey. Paper surveys are double-entered into StudyTRAX™ by research staff who have no knowledge of or access to identifiable participant information or treatment assignment. The two data entries are compared and reconciled.

#### Outcome measures

Table 3 shows the data collection schedule. Our measures include core patient-oriented outcomes for the cLBP trials, including those recommended by the Report of the NIH Task Force on Research Standards for Chronic Low Back Pain [73]. Change in pain intensity and back-related function are the two co-primary outcomes at 12 weeks [63, 71]. Secondary back pain outcomes include pain medicine use in the previous week, including medication class and dosage [81]; health-related quality of life (SF-12) [82]; pain interference [83]; overall improvement (7-point Likert scale, 0 = extremely worsened to 6 = extremely improved); and patient satisfaction with cLBP treatment (5-point Likert scale, 1 = very satisfied to 5 = very dissatisfied) [84]. Psychological secondary outcomes include post-traumatic stress symptoms (PCL-C) [85] and depression symptoms measured using the 9-item Patient Health Questionnaire (PHQ-9) [86]. The Pittsburgh Sleep Quality Index (PSQI) is also administered [87]. Exploratory neuropsychological outcomes include post-concussive symptoms measured using the Neurobehavioral Symptom Inventory (NSI) [88], 7-item Generalized Anxiety Disorder questionnaire (GAD-7) [89], Coping Strategies Questionnaire (CSQ) [90], and Pain Self-Efficacy Questionnaire (PSEQ) [91]. Exploratory social outcomes include marital/family functioning measured using the Dyadic Adjustment Scale (DAS) [92], employment status (Work Productivity and Activity Impairment questionnaire) [93], and housing status. NIH PROMIS® (Patient Reported Outcomes Measurement Information System) tools [94] for outcomes of interest, such as pain, function, depression, anxiety, and sleep, are used to assess how well they correlate with the DVPRS, RMDQ, PHQ-9, GAD-7, and PSQI. At baseline, we also gather standard sociodemographic information and possible covariates, including race, ethnicity, income, education, military service history, medical comorbidities, back pain history, expectation of helpfulness for the different interventions [95], and preference of treatment assignment.

**Table 3:** Schedule of assessments

Measures	Enrollment	Baseline	6 weeks	12 weeks	24 weeks
Screening and enrollment					
Eligibility screening	✓				
Informed consent	✓				
Baseline information					
Sociodemographics*		✓			
Expectations and preference		✓			
Back pain history and comorbidities		✓			
Primary outcomes					
Low back pain intensity (DVPRS)		✓	✓	✓	✓
Back-related function (RMDQ)		✓	✓	✓	✓
Secondary outcomes					
Pain medication use		✓	✓	✓	✓
PTSD CheckList—Civilian version (PCL-C)		✓	✓	✓	✓
Health-related quality of life (SF-12)		✓	✓	✓	✓
Satisfaction with treatment		✓	✓	✓	✓
Global improvement			✓	✓	✓
Cost-effectiveness outcomes					
Work productivity		✓	✓	✓	✓
Medical utilization and cost		✓	✓	✓	✓
Exploratory outcomes					
PROMIS-29 and pain interference		✓	✓	✓	✓
Depression (PHQ-9) and anxiety (GAD-7)		✓	✓	✓	✓
Relationship satisfaction (DAS)		✓		✓	✓
Pain self-efficacy (PSEQ)		✓		✓	✓
Sleep quality (PSQI)		✓		✓	✓
Coping strategies (CSQ)		✓		✓	✓
Post-concussive symptoms (NSI)		✓		✓	✓
Possible covariates and confounders					
Exercise history		✓	✓	✓	✓
Low back pain treatments		✓	✓	✓	✓
Alcohol, drugs, and smoking		✓		✓	✓

*Sociodemographic information includes gender, age, relationship status, ethnicity, race, income, housing, education level, and military service history

DVPRS = Defense and Veterans Pain Rating Scale; RMDQ = Roland Morris Disability Questionnaire; PROMIS = Patient Reported Outcome Measurement Information System; SF-12 = Short Form 12-item Health Survey; PHQ-9 = 9-item Patient Health Questionnaire; GAD-7 = 7-item General Anxiety Disorder survey; DAS = Dyadic Adjustment Scale; PSQI = Pittsburgh Sleep Quality Index; NSI = Neurobehavioral Symptom Inventory

#### Cost-effectiveness

We use a multi-method approach to collect cost data at baseline, 6 weeks, 12 weeks, and 24 weeks. Since our interventions may influence other common comorbidities of cLBP (e.g., depression [52]), we measure both back-related utilization and total medical utilization. Direct medical costs are measured and consist of the cost of (1) implementing the interventions themselves and (2) ongoing medical utilization before, during, and after the intervention. Intervention implementation costs (e.g., non-study-specific staff hours, materials, facility use) are captured from study records and valued at their actual costs. Ongoing total VA medical utilization including visits, hospitalizations, tests, radiology, and medications are taken directly from the electronic medical record system. Direct medical costs are valued at their actual costs to the VA. Any non-VA medical utilization (e.g., chiropractors, out-of-pocket back-related expenses) is obtained from a cost questionnaire completed by the Veteran with the primary initial recall period being the past 6 weeks and is valued at the reported actual price paid by Veterans. Indirect costs (i.e., lost productivity) for employed Veterans will be calculated as the number of lost productive hours (as reported in the Work Productivity and Activity Impairment questionnaire) multiplied by a national average cost of employment for each Veteran’s general job category. Lost productivity costs for those not in the work force are assumed to be captured by their report of overall quality of life [96]. Lost productivity costs for those looking for work will be considered in the sensitivity analyses.

#### Qualitative interviews

Qualitative interviews occur pre-intervention and post-intervention with a subset of partnered yoga participants to explore the impact of cLBP and comorbid mental health disorders on family and marital processes. This will also provide a greater detailed understanding of yoga's perceived impact of on the Veteran and possibly the Veteran’s family. After randomization, a purposive sample of partnered Veterans (*n* = 20 couples) in the yoga arm are recruited to participate. To be eligible for interviews, Veterans must report having a cohabitating or long-term committed partner/spouse. In addition, both the Veteran and the Veteran’s partner must give consent to participate in the interviews. Sampling takes place in a logistically feasible way until the goal sample size is reached.

Separate and conjoint interviews are conducted by a trained qualitative interviewer with the Veteran and his or her partner at two time points – prior to participation in yoga and approximately four weeks after ending classes – in order to maximize the opportunity to authentically discuss the impact of cLBP and yoga on family relationships. Interviews are conducted with each member of the couple individually followed by a short conjoint interview. We encourage couples to attend interviews at the same time. However, if this is not possible, we conduct the two interviews within approximately 72 hours. Semistructured interview guides incorporate the following domains: the Veteran’s perception of the impact of cLBP on himself or herself and the partner or family in terms of mood and functioning; the partner’s perception of the impact of the Veteran’s cLBP on the Veteran’s mood and functioning; the partner’s perception of the Veteran’s cLBP on her/his own and overall family mood and functioning; the attitude of the Veteran and partner toward yoga; the Veteran and partner’s perception of change in the Veteran’s cLBP; the Veteran and partner’s perception of any possible change in psychological concerns including PTSS, depression, and anxiety after yoga; and the Veteran and partner’s perception of any change in overall family mood and functioning after yoga classes (see Additional file 4).

Interviewers obtain informed consent from the Veteran and partner prior to the interviews. Each individual receives $25 for each 60–90 minute interview visit. Interviews take place at a location convenient to the couple.

If a couple completes a pre-intervention interview but declines to complete a second interview, we attempt to replace that couple with another couple.

### Adverse events and data monitoring

The same strategy for collecting adverse event data is implemented for both study arms. Participants are instructed at enrollment to contact research staff immediately if they experience any adverse event during the study. All participants have 24-hour emergency contact information for the site investigator and another member of the research team. All post-baseline surveys and education check-in calls include questions on whether the Veteran believes he or she has incurred any possible adverse events. Veterans in the yoga group are given the opportunity to speak with a research staff member regarding any potential adverse events during attendance and check-ins at yoga classes. Research staff and the principal investigator follow up on all adverse event reports as appropriate. Unanticipated problems (unexpected, related, and serious) are reported to the IRBs and sponsor within 48 hours.

A Data and Safety Monitoring Board (DSMB) reviews the study conduct and results throughout recruitment, data collection, and treatment implementation phases. The DSMB is composed of four members who are not involved in the conduct of the study. The DSMB meets semiannually throughout the study period to review all adverse event reports, accrual progress, and retention. A summary report of the board’s findings and recommendations are submitted to the sponsor and IRBs.

This study will be stopped prior to its completion if: (1) the intervention(s) are associated with adverse effects that significantly impact the risk-benefit ratio; (2) study recruitment or retention becomes futile; (3) any new information becomes available during the trial that necessitates stopping the trial; or (4) other situations occur that might warrant stopping the trial.

### Data analysis

We will conduct both intention-to-treat and per-protocol analyses, with the intention-to-treat analysis being primary. Descriptive statistics including means, medians, and standard deviations for continuous variables, and the number and proportions for categorical variables will be reported. All analyses will be performed at the α = 0.05 level of significance.

The success of randomization will first be assessed. We will compare the two groups on baseline characteristics and demographics, including stratification variables, with Student’s t test or chi-square test, as appropriate, using an α = 0.1 level of significance. Variables that differ between the groups at baseline will be considered possible confounders and adjusted for in subsequent analyses.

#### Primary outcome analyses

The primary hypothesis is that 12 weeks of yoga will be more effective than education for improving pain and function. For both outcomes, we will determine if yoga is superior to education based on a longitudinal model incorporating all measurements across the study period, including baseline, week 6, week 12, and week 24. The primary hypothesis will be tested using a contrast of the change from baseline to week 12 comparing the two groups within this model. To account for correlation among repeated measures for the same individual, we will assume an unstructured covariance for the initial model. A simpler model may be used for the covariance if appropriate. No missing data will be replaced in these analyses, and all available data can be included. The analysis is considered to be unbiased under a missing-at-random assumption. It is possible that any missing data may be non-ignorably missing rather than missing-at-random. We will perform sensitivity analyses to determine if the results are robust to the missing data mechanism. Note that we require statistical significance for both outcomes rather than either outcome to address the issue of multiple testing. We will report mean differences between groups with standard errors, 95 % confidence intervals, and *p* values. If the analysis of the success of randomization finds any imbalance between groups, the above model will be adjusted for these factors.

Per-protocol analysis will also be conducted similarly. Adherence to the yoga protocol will be defined as attending at least 9 of the 12 yoga classes. For education, adherence will be defined as reading at least three-fourths of the book by self-report.

#### Secondary back pain outcome analyses

For pain medication use, we will examine the overall use during the previous week at week 12. First, a chi-square test will be used to compare medication use at week 12 between groups. Next, logistic regression with terms for treatment group and adjusting for potential confounders, including baseline pain medication use, will be used to compare medication usage rates. Use of medication subtypes, including nonsteroidal anti-inflammatory drugs, acetaminophen, and opioids, will be similarly compared. We will report odds ratios with corresponding 95 % confidence intervals and *p* values. Physical and mental health summary scores will be calculated from SF-12 data using population normative data and means compared between groups as for the primary outcomes above. We will also compare the proportion of participants in each group achieving ≥30 % and ≥50 % improvements from baseline for the co-primary outcomes, often considered to correspond to minimal and moderate clinically meaningful change, respectively.

#### Psychological and exploratory analyses

Our hypothesis is that yoga will be superior compared to education for reducing PTSS in the subset of participants with comorbid PTSS (baseline PCL-C ≥30). We will first compare PCL-C scores between groups for the subset of participants who qualify as having PTSS at baseline using methods similar to those described above for primary outcome analyses. We will also examine the whole study sample and the subset of participants with lower baseline PCL-C scores. We stratify for this variable during randomization and thus anticipate similar numbers and baseline characteristics between the two study groups. We will also explore whether a change in PTSS is an effect modifier for our primary outcomes of pain and function. We will similarly compare yoga to education for the subgroup of participants with moderate or greater depressive symptoms (PHQ-9 ≥10) [97] and those with less depressive symptoms. Exploratory subset analyses will also be conducted for Veterans scoring moderate or higher on anxiety symptoms (GAD-7 ≥10) and sleep difficulties (PSQI >5). Similarly, an exploratory subset analysis will be conducted for participants with post-concussive symptoms, defined as scoring ≥20 on the NSI [98].

Since back pain in older Veterans may be different structurally compared to younger Veterans, we will also complete exploratory subset analyses of Veterans ≤45 and >45 years old to assess if age confounds the response to yoga.

To analyze intermediate-term outcomes, we will examine changes from baseline to week 6 using methodology similar to the primary 12-week analyses. Additional subgroup longitudinal analyses can be done using only data from those participants who were 12-week completers. We will also compare the change in pain medication usage over time using a generalized-estimating equation approach or nonlinear mixed effects model to account for the repeated measures of a dichotomous outcome. This approach will parallel the longitudinal analyses for continuous outcomes described above.

#### Cost-effectiveness analyses

All cost-effectiveness analyses will use the intention-to-treat principle. Quality-adjusted life-years (QALYs) will be calculated from the results of the SF-12 based on an algorithm developed by Brazier et al. [99] For the Veteran perspective, we will compare Veterans' incremental out-of-pocket costs (e.g., transportation, over-the-counter medications, and co-payments) to their incremental QALY impacts. For the VHA perspective, incremental direct intervention costs will be compared to ongoing direct medical utilization costs. This cost-benefit analysis will address whether, economically, yoga should be a treatment option for the VA population. For the society perspective, we will conduct a cost-utility analysis by comparing the incremental societal costs for each treatment arm (i.e., direct medical, nonmedical, and productivity costs) to the incremental change in QALYs [100]. We will use bootstrap methods to calculate confidence intervals [101] and perform one-way sensitivity analyses to determine the robustness of our estimates with different assumptions used to value productivity [102]. Combined non-back pain and back pain-related utilization will be used for all of these analyses. We will also perform sensitivity analyses using only back pain-related utilization.

#### Qualitative analyses

Qualitative interviews will be downloaded from digital recorders, transcribed, and cleaned by qualitative research staff. Given the exploratory nature of the interviews, codes will be developed both inductively and deductively using techniques from Grounded Theory [103]. Initially, three research team members will read transcripts from three dyads (six interviews) to identify content categories using the process of line-by-line coding. In order to establish reliability, the team will then confer to discuss codes, condense overlapping areas, and consider alternative possibilities with the goal of developing a coding schema to be used with all interviews. Subsequently, two team members will read each of the remaining pairs of transcripts and continue to conduct focused coding using the decided-upon schema. The team will confer every four transcripts to insure that the codes remain relevant and accurate given the research questions. Where necessary, coding categories will be changed and condensed. This process of line-by-line and focused coding will ultimately lead to axial coding, where larger thematic categories and relationships between them are identified. Matrices will be utilized to compare and contrast code categories and individual cases. The software program NVivo (QSR^©^, Melbourne, Australia) will be used for qualitative data management and analysis.

## Discussion

The proposed RCT will (1) establish a structured reproducible yoga protocol uniquely suited to Veteran populations with cLBP and associated psychological comorbid symptoms; (2) increase our knowledge of the feasibility and impact of yoga on Veterans' cLBP, psychological comorbidities, and family functioning; and (3) provide a strong foundation for larger multisite studies and implementation projects.

The Institute of Medicine report *Relieving Pain in America* recognized that protocols for pain management approaches must be adapted to the unique needs of Veterans and undergo rigorous testing for clinical effectiveness and cost-effectiveness [3]. Cost analyses have been infrequently applied to complementary and integrative medicine treatments in general [104, 105], and to the best of our knowledge and according to the published literature, never for integrative medicine or yoga in a military setting. Group self-care interventions such as yoga, if effective, have the potential to be cost saving to the VHA and cost-effective to society.

Research of yoga as a therapeutic modality is relatively new and even less well developed for military populations. Given the complexity and heterogeneity of yoga practices [106], structured protocols that are evidence-based and reproducible are critical if wide dissemination is to ultimately occur. The design and implementation of our yoga intervention for the unique needs of Veteran populations with cLBP and associated psychological comorbidities is novel. This study adapts an existing structured yoga protocol validated in civilian populations using an expert panel including Veterans and yoga instructors with expertise in teaching Veterans.

The results of this study have the potential to impact the approach and management of cLBP in accordance with the DoD-VA vision, i.e., a more integrative, interdisciplinary focus on active self-care approaches that empower patients to have greater control of their condition [63]. Despite enthusiasm for offering yoga to Veterans, yoga instruction to date is not well standardized or implemented widely. More importantly, there is little strong evidence for yoga’s effect on PTSD, depression, or other psychosocial problems. We anticipate this trial will help determine if yoga can become a safe, clinically effective, cost-effective, and scalable nonpharmacologic approach to address the physical and psychosocial dimensions of cLBP in Veterans.

## Trial status

This trial is currently recruiting participants.

### Consent

Written informed consent was obtained from the yoga home practice video actor(s) for publication of this manuscript and accompanying videos. A copy of the written consent is available for review by the Editor-in-Chief of this journal.

## Additional files

Additional file 1: Participant Yoga Manual. A take-home guidebook for yoga participants describing and depicting yoga exercises to aid home practice. (PDF 4.52 mb) Additional file 2: Yoga Home Practice Introduction Video. An introductory video that presents Veterans describing the value of yoga practice in their own lives and explains how to set up home practice. (MOV 315 mb) Additional file 3: Yoga Home Practice Video Weeks 1-6. A 30-minute home practice video that parallels yoga classes in weeks 1-6 of the study. (MOV 912 mb) Additional file 4: Yoga Home Practice Video Weeks 7-12. A 30-minute home practice video that parallels yoga classes in weeks 7-12 of the study. (MOV 1.33 gb) Additional file 5: Yoga Breathing and Relaxation Video. A 20-minute video containing extended breathing and relaxation exercises. (MOV 703 mb) Additional file 6: Yoga Teacher Manual. A training and reference manual given to study yoga instructors that includes detailed information on the segment themes, weekby- week yoga class structure, teaching individual postures with or without modifications, teaching breathing exercises, and leading Veterans through integrated relaxation. (PDF 3.52 mb) Additional file 7: Manual of Procedures. Comprehensive manual of standard operating procedures for recruitment, enrollment, data collection/management, interventions, qualitative interviews, and adverse events. (PDF 5.66 mb)

## Abbreviations

cLBP: Chronic low back pain
CSQ: Coping Strategies Questionnaire
DAS: Dyadic Adjustment Scale
DVPRS: Defense and Veterans Pain Rating Scale
GAD-7: 7-item General Anxiety Disorder questionnaire
NCCIH: National Center for Complementary and Integrative Health
NSI: Neurobehavioral Symptom Inventory
OEF: Operation Enduring Freedom
OIF/OND: Operations Iraqi Freedom/New Dawn
PCL-C: PTSD CheckList – Civilian version
PHQ-9: 9-item Patient Health Questionnaire
PROMIS: Patient-Reported Outcomes Measurement Information System
PSEQ: Pain Self-Efficacy Questionnaire
PSQI: Pittsburgh Sleep Quality Index
PTSD: Post-Traumatic Stress Disorder
PTSS: Post-traumatic stress symptoms
QALY: Quality-adjusted life year
RCT: Randomized controlled trial
RMDQ: Roland-Morris Disability Questionnaire
SF-12: Short form 12-item health survey
VA: Department of Veteran Affairs
VHA: Veterans Health Administration
