# Supplementary material for: Yoga versus education for Veterans with chronic low back pain: study protocol for a randomized controlled trial
Source: Trials. 2016 Apr 29;17:224. doi: 10.1186/s13063-016-1321-5 (PMC4850721; doi:10.1186/s13063-016-1321-5)
Supplement: Additional file 1: — Participant Yoga Manual. A take-home guidebook for yoga participants describing and depicting yoga exercises to aid home practice. (PDF 4.52 mb) [file 13063_2016_1321_MOESM1_ESM.pdf]

Yoga Home Practice Guidebook

A Study Comparing Yoga and Education for Veterans with Chronic Low Back Pain

Veterans Back to Health

© Boston Medical Center
Veterans Back to Health Trial

Table of Contents

Welcome 1

Study Contact Information 2

Making the Most Out of Your Yoga Experience 3

Setting up Your Home Practice 4

Completing Your Home Practice Log 5

Yoga Poses for Home Practice

- Segment 1: Opening to Something New 6
- Segment 2: Listening to Your Back 10
- Segment 3: Engaging Your Power 14
- Segment 4: Bringing it Home 18

Instructions for Specific Yoga Poses

- Baby Dancer Pose 22
- Baby Dancer Pose in Chair 23
- Big Toe Pose 24
- Bridge Pose 25
- Bridge Pose in Chair 26
- Cat/Cow Pose 27
- Cat/Cow Pose in Chair 28
- Chair Dog (Wall Dog) Pose 29
- Chair Pose 30
- Chair Pose in Chair 31
- Child’s Pose 32
- Child’s Pose in Chair 33
- Cobra Pose 34

© Boston Medical Center
Veterans Back to Health Trial

Table of Contents

Crescent Moon Pose 35
Crescent Moon Pose in Chair 36
Downward Dog Pose 37
Eye of the Needle Pose 38
Eye of the Needle Pose in Chair 39
Final Resting Pose 40
Forward Bend Pose 41
Forward Bend Pose in Chair 42
Knees to Chest Pose 43
Knees to Chest Pose in Chair 44
Knees Together Twist Pose 45
Legs up the Wall Pose 46
Locust Pose 47
Mountain Pose 48
Mountain Pose in Chair 49
Pelvic Tilt Pose 50
Pelvic Tilt Pose in Chair 51
Plank Pose 52
Reclining Chest Opener Pose 53
Reclining Cobbler Pose 54
Shoulder Opener Pose 55
Shoulder Opener Pose in Chair 56
Side Hip Strengtheners 57
Side Plank Pose 58
Sphinx Pose 59

© Boston Medical Center
Veterans Back to Health Trial

Table of Contents

Sphinx Pose in Chair 60
Table Top with Leg Extended Pose 61
Triangle Pose 62
Triangle Pose in Chair 63
Warrior I Pose 64
Warrior I Pose in Chair 65
Warrior II Pose 66
Warrior II Pose in Chair 67
Warrior at Wall Pose 68
Wheel Pose 69
Wheel Pose in Chair 70
Wide-Leg Forward Bend Pose 71

© Boston Medical Center
Veterans Back to Health Trial

Welcome

Back to Health is a study that compares yoga and education for low back pain. To help us find out which of these is best for back pain, we ask that you are as committed as possible to the study. Please make your best effort to come to all the yoga classes, fill out all the questionnaires honestly, and practice yoga at home. We do realize that emergencies or unavoidable conflicts happen. If you cannot make a class, or have any concerns about the study, please contact the study staff at any time.

We have designed the yoga classes specifically for people with chronic low back pain. The yoga teachers have all been trained in this type of yoga. The classes are aimed at people who have done little or no yoga before. Experts have designed the classes to be as safe as possible for people with chronic low back pain. Over 12 weeks, you will be gradually taught how to do the yoga exercises. Your safety is very important. If you feel that you have any worsened pain, injury, or change in your health status during the study, please let a member of our study team know as soon as possible. Please feel free to call us at **781-687-2000 ext. 5520** at any time if you have any questions or concerns.

We recommend that you practice 30 minutes per day on days when you do not have class. This guidebook was created to assist you in your yoga practice. It will help you recreate what you are learning in class as you begin your home practice. All the poses you learn in class are described in this guidebook along with helpful diagrams. You can keep this booklet at home where you practice yoga. You don’t need to bring it with you to classes or any other study visits.

© Boston Medical Center
Veterans Back to Health Trial

Important Study Contact Information

If you cannot make a class or need to get in touch with our study team, please call us at **781-687-2000 ext. 5520**

Other Study Contact Information

Shihwe Wang
VA Principal Investigator
(781) 687-3538

Dorothy Plumb
VA Project Manager
(781) 687-2520

Rani Elwy
VA Investigator
(781) 687-2861

© Boston Medical Center
Veterans Back to Health Trial

Making the Most Out of Your Yoga Experience

1. Wear loose, comfortable clothing that is easy to move in.
2. Practice 30 minutes every day you do not have yoga class.
3. Practicing with bare feet helps you have grip the yoga mat. But if you prefer socks, that is okay too.
4. Come to class each week. If for any reason you need to miss a class, let the study staff know. We will try to help you make up the missed class.
5. Be sure to turn off cell phones and electronic devices when you practice to limit distractions.
6. Do not eat right before practicing yoga. It's best to practice on an empty stomach.
7. Ask for help whenever you have questions. The teachers are there to help you succeed.
8. Listen to your own body. Do not do anything that causes severe pain or you think may be unsafe. There are always options to help you do yoga in a way that is most comfortable for you.
9. Yoga is not a competition. Try not to compare yourself to others or judge yourself. Yoga practice is unique for everyone and only you know what feels best for you.
10. The use of the breath is what makes yoga different from other exercise. Try to remember to take slow and even breaths with each pose and movement. If you lose the breath, just come back to it.
11. If for <u>any</u> reason you can't or don't want to do a certain yoga exercise, that's okay. Just let the teacher know or sit quietly until you're ready to join back in. If you have a history of head injury, avoid any postures that lower your head below your heart as this can be dangerous. For each exercise, there are many options the teacher can tell you about to help you customize your yoga practice.
12. Consider bringing water to class or having it nearby during home practice.

© Boston Medical Center
Veterans Back to Health Trial

Setting Up Your Home Practice

This book is designed to help you practice yoga at home. Home practice reinforces what you do in class. We suggest practicing at home for 30 minutes on the days you do not have class. Even though you are taking a class with others, your practice is your own – your experience is genuine and unique. Please bring any questions or concerns that come up during your home practice to your teacher when you have class. You'll have time to speak with your teacher(s) individually before and after class. Here are a few suggestions to make your home yoga practice a success:

1. **Find space** at home where you can practice. If possible, leave your yoga items such as your mat, block and strap there. It needs to be large enough so you can stretch upwards as well as to the sides. Try to find a space that is free of clutter, quiet, and private.

2. **Pick a time** that you can be alone and not be interrupted. Many people find that early morning or evening works best, and this is often when your stomach is empty as well.

3. **Ask for support from your family and friends.** If you live with others, let them know about your need to practice yoga every day. Ask for their support. Ask them to please not interrupt you while you practice.

4. **Wear comfortable clothing** that allows you to move easily and consider taking your socks off for better balance and grip on your mat.

5. **Make your practice area personal and attractive.** Place a few items where you practice that have special meaning for you. Keep the space clean and free from distractions. If you'd like, play calming music to aid in relaxation and help keep you focused on practice.

© Boston Medical Center
Veterans Back to Health Trial

Completing Your Home Practice Log

1. Begin a new home practice log each week.
2. Simply check off whether or not you have practiced yoga at home and for how many minutes each day.
3. You may write comments about your yoga practice or to help you keep track of when you attended class.
4. Please bring your home practice log to class and hand it in each week.

Fill out the dates and start a new log each week

You can write comments here if you want to. You might decide to make note of the days you went to class to help you keep track.

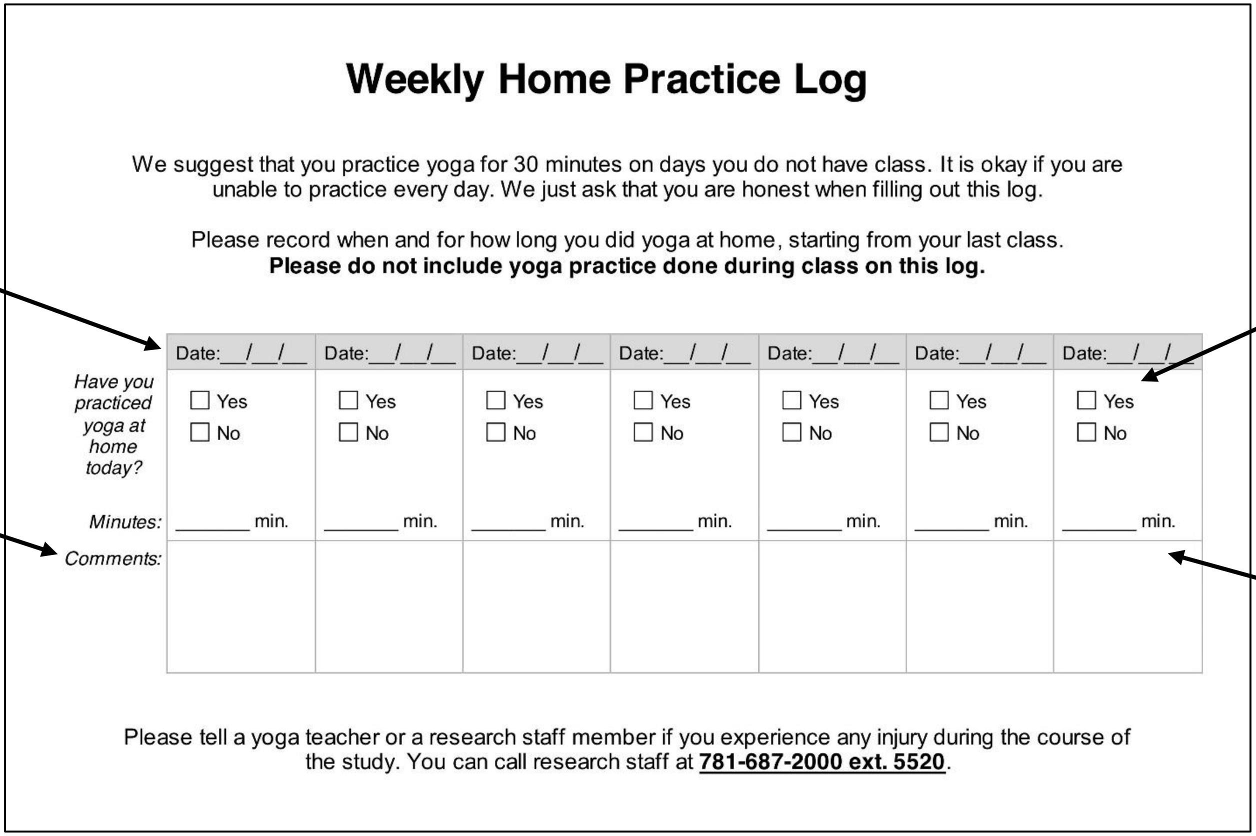

Weekly Home Practice Log

We suggest that you practice yoga for 30 minutes on days you do not have class. It is okay if you are unable to practice every day. We just ask that you are honest when filling out this log.

Please record when and for how long you did yoga at home, starting from your last class.
Please do not include yoga practice done during class on this log.

	Date:__/__/__	Date:__/__/__	Date:__/__/__	Date:__/__/__	Date:__/__/__	Date:__/__/__	Date:__/__/__
Have you practiced yoga at home today?	☐ Yes ☐ No	☐ Yes ☐ No	☐ Yes ☐ No	☐ Yes ☐ No	☐ Yes ☐ No	☐ Yes ☐ No	☐ Yes ☐ No
Minutes:	_______ min.	_______ min.	_______ min.	_______ min.	_______ min.	_______ min.	_______ min.
Comments:							

Please tell a yoga teacher or a research staff member if you experience any injury during the course of the study. You can call research staff at **781-687-2000 ext. 5520**.

Check 'Yes' if you have practiced or 'No' if you have not each day **(don't include practice in class)**

Write the number of minutes you practiced, if applicable

© Boston Medical Center
Veterans Back to Health Trial

Yoga Poses for Home Practice

Segment One (Weeks 1-3): Opening to Something New

You can select a few of the following warm-ups to start your home practice:

1. Knees to Chest Pose
2. Knees Together Twist Pose
3. Pelvic Tilt Pose
4. Cat/Cow Pose
5. Wheel Pose
6. Mountain Pose
7. Shoulder Opener Pose
8. Crescent Moon Pose

After the warm-ups, you can do a few or all of the below yoga postures:

1. Chair Pose
2. Warrior at Wall Pose
3. Triangle at Wall Pose
4. Forward Bend Pose
5. Child's Pose in Chair
6. Chair Dog Pose
7. Sphinx Pose

After some yoga postures, finish with these cool-down poses:

1. Supported Bridge Pose
2. Knees to Chest Pose
3. Knees Together Twist Pose

End your practice with a short relaxation. You can choose from the following relaxation positions:

1. Final Resting Pose
2. Supported Final Resting Pose
3. Reclining Chest Opener

© Boston Medical Center
Veterans Back to Health Trial

Segment One: Opening to Something New

Options for Warm-Ups

Knees to Chest Pose

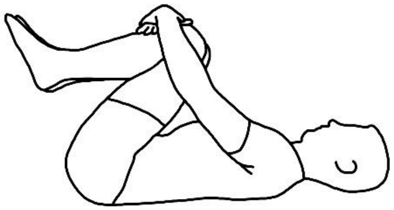

Knees Together Twist Pose

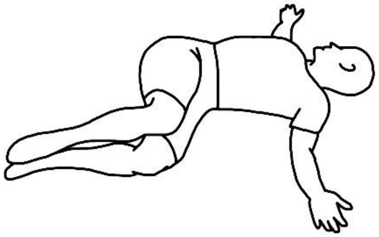

Pelvic Tilt Pose

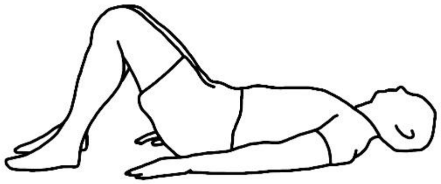

Cat/Cow Pose

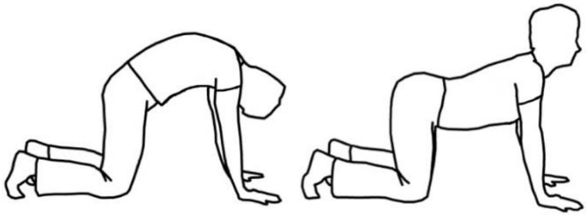

Wheel Pose

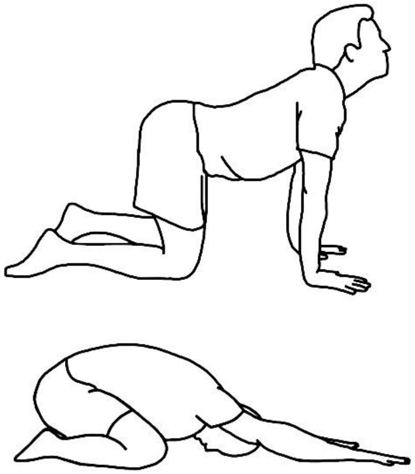

Mountain Pose

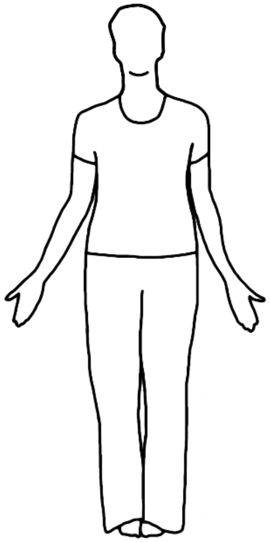

Shoulder Opener Pose

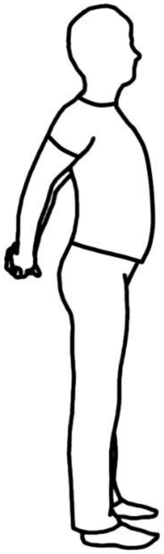

Crescent Moon Pose

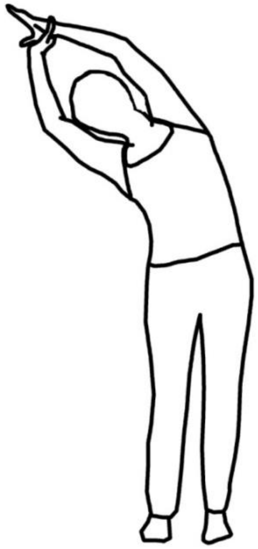

© Boston Medical Center
Veterans Back to Health Trial

Segment One: Opening to Something New

Options for Yoga Postures

Child's Pose in Chair

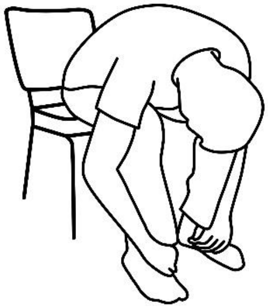

Sphinx Pose

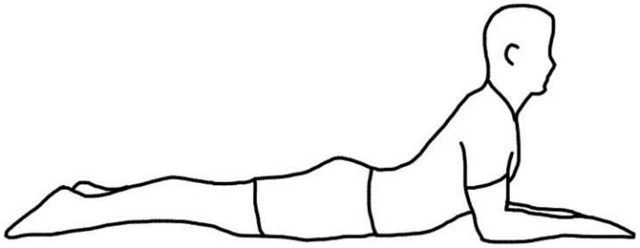

Chair Dog Pose

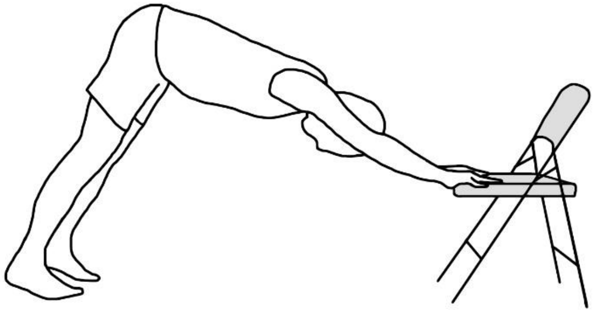

Chair Pose

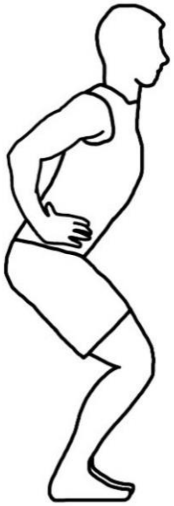

Warrior at Wall Pose

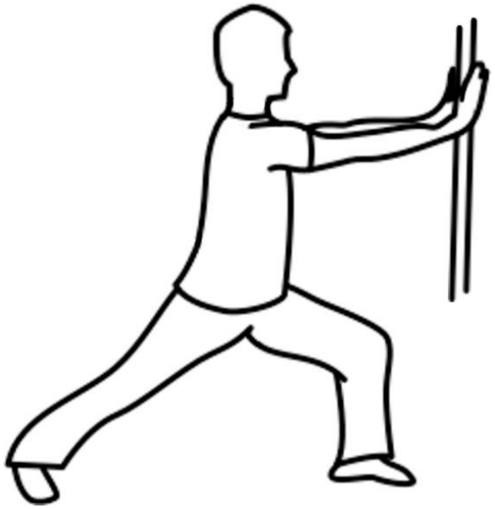

Forward Bend Pose

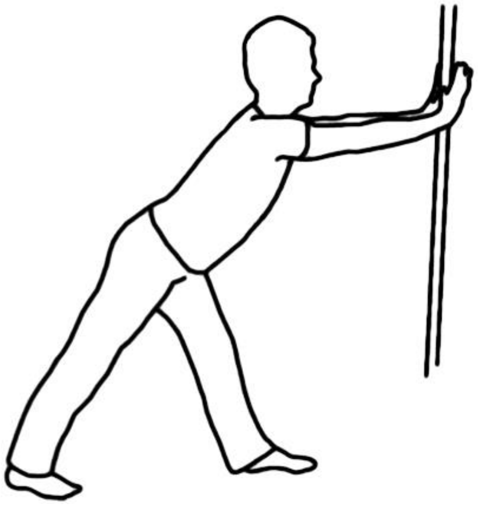

Triangle at Wall Pose

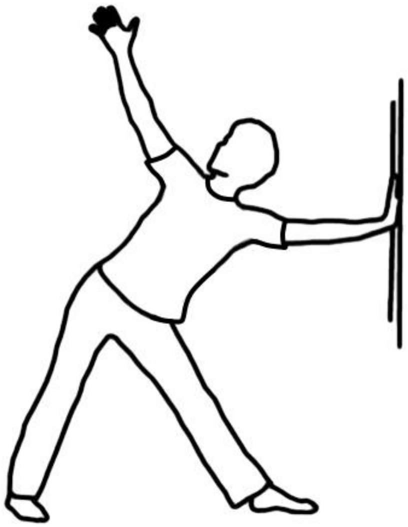

© Boston Medical Center
Veterans Back to Health Trial

Segment One: Opening to Something New

Options for Cool-Down Postures

Supported Bridge Pose

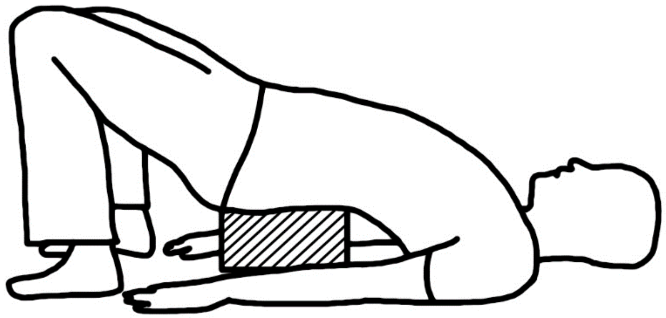

Knees to Chest Pose

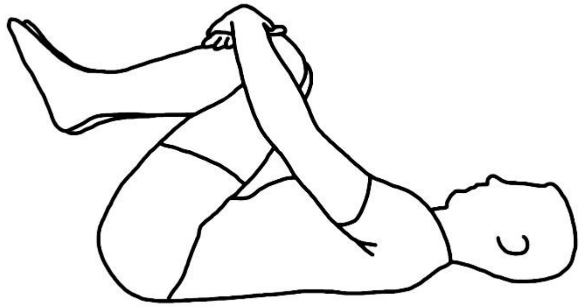

Knees Together Twist Pose

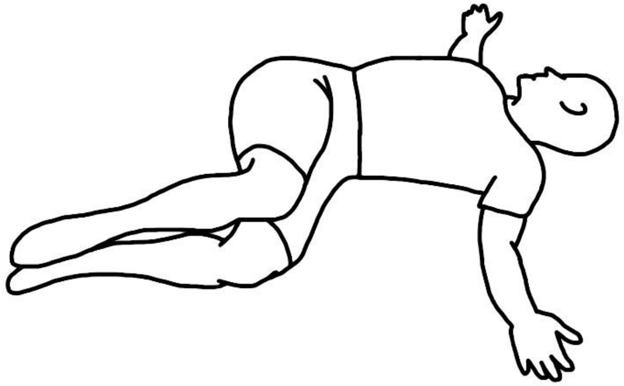

© Boston Medical Center
Veterans Back to Health Trial

Yoga Poses for Home Practice

Segment Two (Weeks 4-6): Listening to Your Back

You can select a few of the following warm-ups to start your home practice:

1. Knees to Chest Pose
2. Knees Together Twist Pose
3. Big Toe Pose
4. Table Top Pose
5. Mountain Pose
6. Shoulder Opener Pose
7. Crescent Moon Pose
8. Chair Pose

After the warm-ups, you can do a few or all of the below yoga postures:

1. Triangle at Wall Pose
2. Warrior at Wall Pose
3. Wide-Legged Forward Bend Pose at Wall
4. Chair Dog Pose
5. Child's Pose in Chair
6. Locust Pose (Legs Only)
7. Sphinx Pose
8. Side Hip Strengtheners

After some yoga postures, finish with all or some of these cool-down poses:

1. Supported Bridge Pose
2. Knees Together Twist Pose
3. Knees to Chest Pose
4. Reclining Cobbler Pose

End your practice with a short relaxation. You can choose from the following relaxation positions:

1. Final Resting Pose
2. Supported Final Resting Pose
3. Reclining Chest Opener Pose

© Boston Medical Center
Veterans Back to Health Trial

Segment Two: Listening to Your Back

Options for Warm-Ups

Knees to Chest Pose

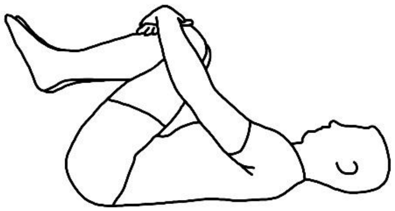

Knees Together Twist Pose

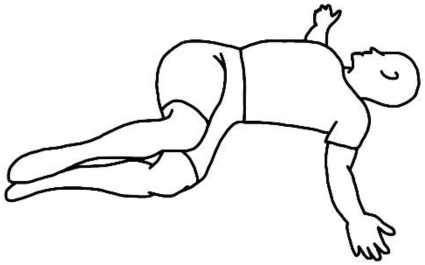

Big Toe Pose

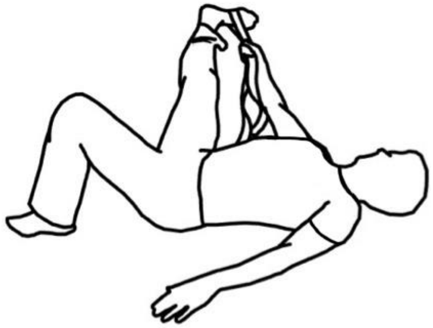

Table Top Pose

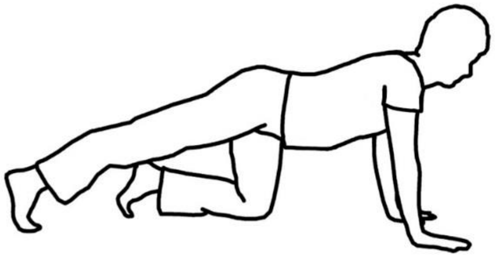

Mountain Pose

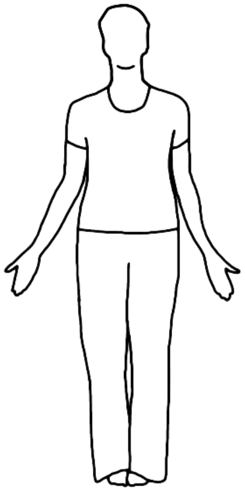

Shoulder Opener Pose

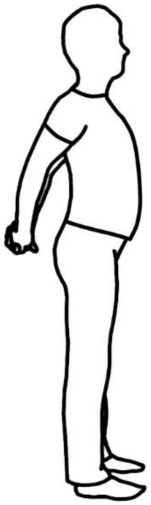

Crescent Moon Pose

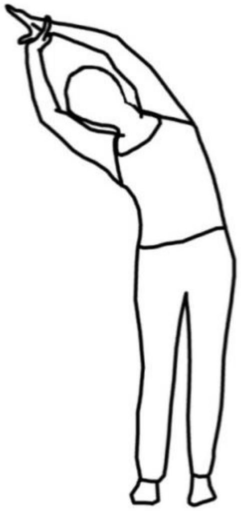

Chair Pose

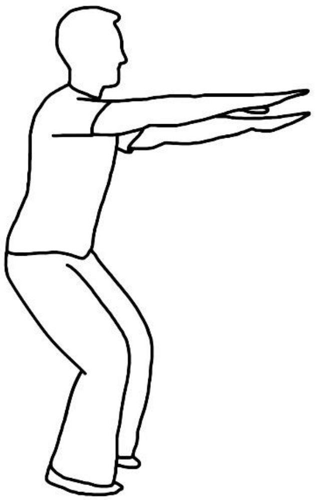

© Boston Medical Center
Veterans Back to Health Trial

Segment Two: Listening to Your Back

<u>Options for Yoga Postures</u>

Triangle at Wall Pose

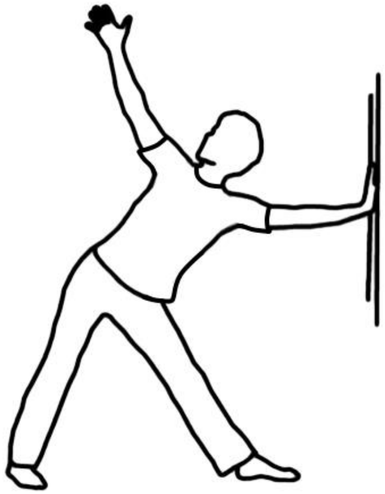

Warrior at Wall Pose

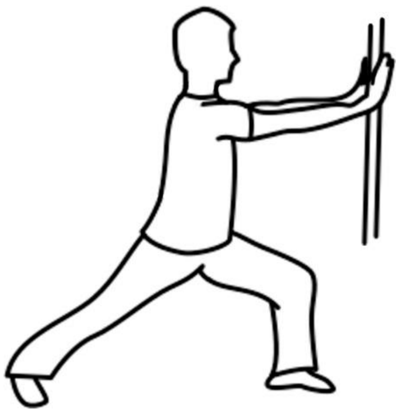

Wide-Leg Bend at Wall

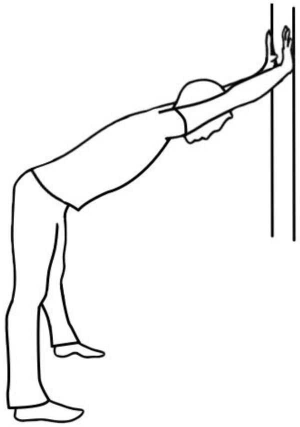

Chair Dog Pose

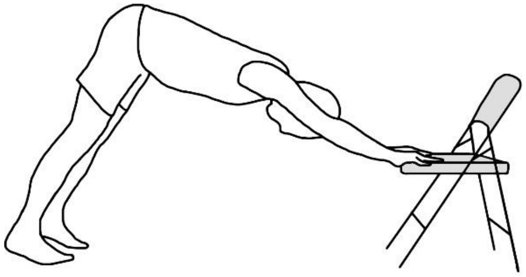

Side Hip Strengtheners

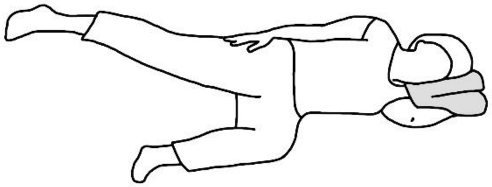

Locust Pose

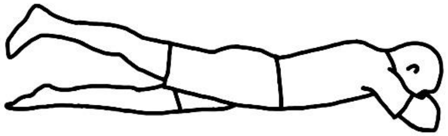

Sphinx Pose

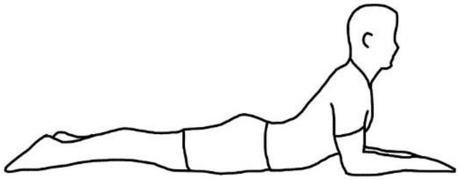

Child's Pose in Chair

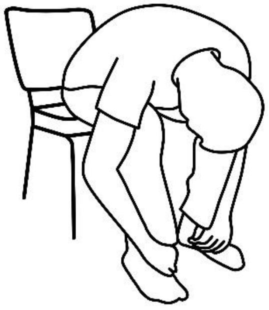

© Boston Medical Center
Veterans Back to Health Trial

Segment Two: Listening to Your Back

Options for Cool-Down Postures

Supported Bridge Pose

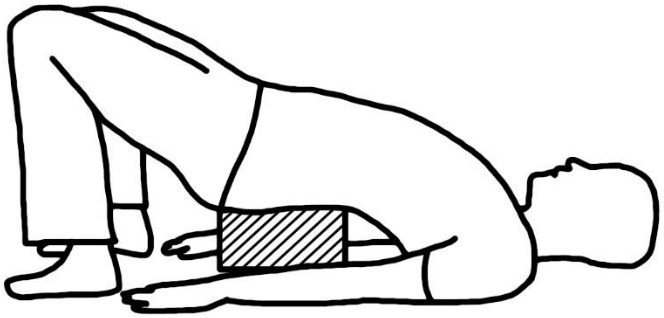

Knees Together Twist Pose

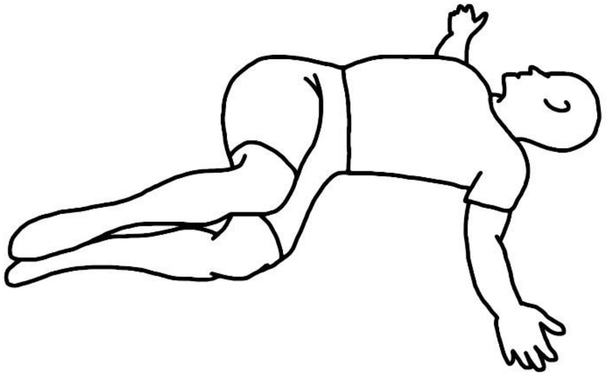

Knees to Chest Pose

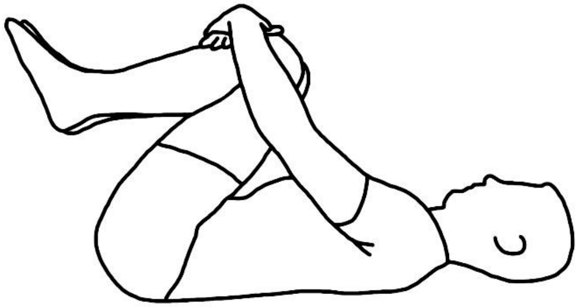

Reclining Cobbler Pose

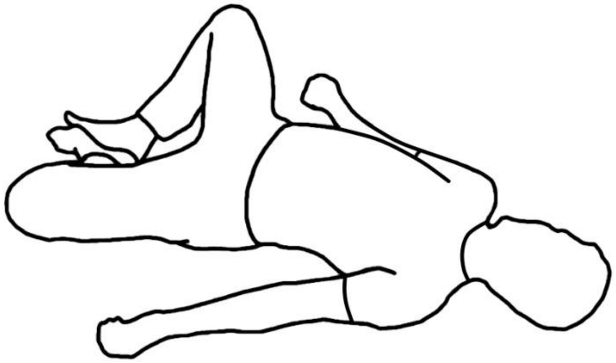

© Boston Medical Center
Veterans Back to Health Trial

Yoga Poses for Home Practice

Segment Three (Weeks 7-9): Engaging Your Power

You can select a few of the following warm-ups to start your home practice:

1. Knees to Chest Pose
2. Knees Together Twist Pose
3. Big Toe Pose
4. Cat/Cow Pose
5. Wheel Pose
6. Mountain Pose
7. Shoulder Opener Pose
8. Chair Pose

After the warm-ups, you can do a few or all of the below yoga postures:

1. Triangle Pose
2. Warrior I Pose
3. Wide-Legged Forward Bend Pose
4. Child's Pose
5. Downward Dog Pose
6. Plank Poses
7. Side Hip Strengtheners
8. Eye of the Needle Pose

After some yoga postures, finish with these cool down postures:

1. Bridge Pose
2. Knees Together Twist Pose
3. Knees to Chest Pose
4. Reclining Cobbler Pose
5. Legs up the Wall Pose

End your practice with a short relaxation. You can choose from the following relaxation positions:

1. Final Resting Pose
2. Supported Final Resting Pose
3. Reclining Chest Opener Pose

© Boston Medical Center
Veterans Back to Health Trial

Segment Three: Engaging Your Power

Options for Warm-Ups

Knees to Chest Pose

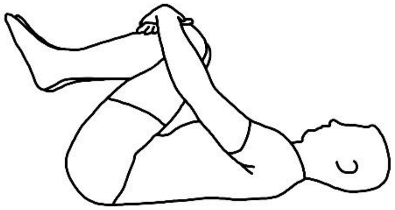

Knees Together Twist Pose

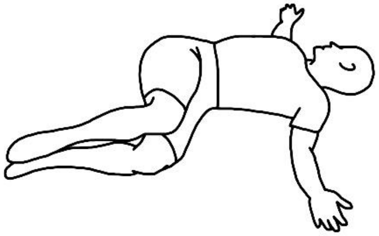

Big Toe Pose

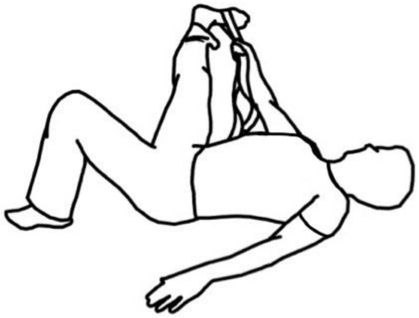

Cat/Cow Pose

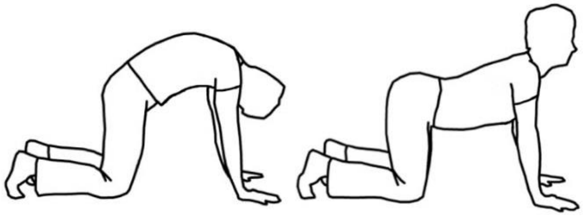

Wheel Pose

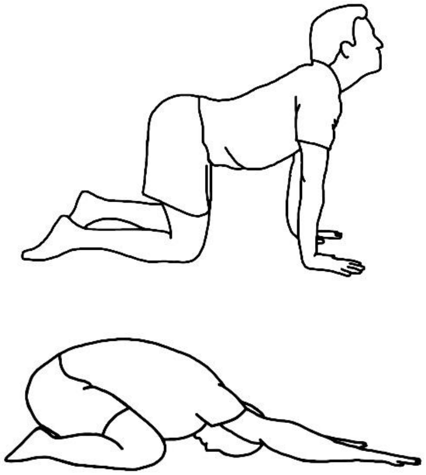

Mountain Pose

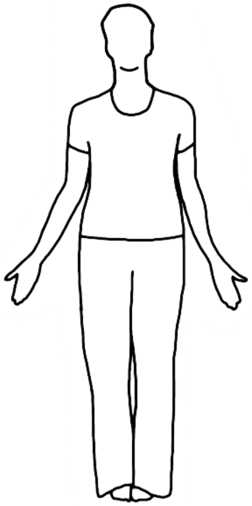

Shoulder Opener Pose

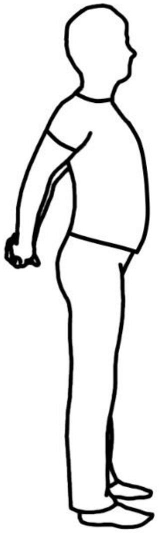

Chair Pose

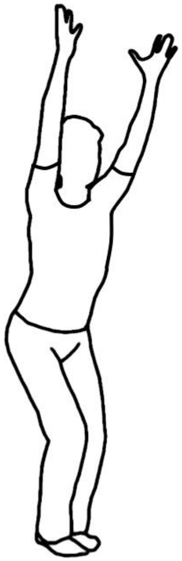

© Boston Medical Center
Veterans Back to Health Trial

Segment Three: Engaging Your Power

Options for Yoga Postures

Triangle Pose

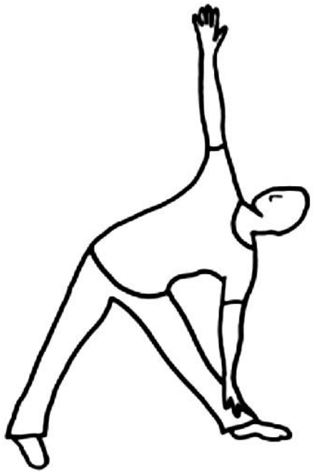

Warrior I Pose

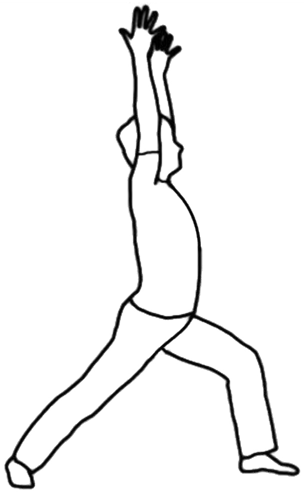

Wide-Leg Forward Bend

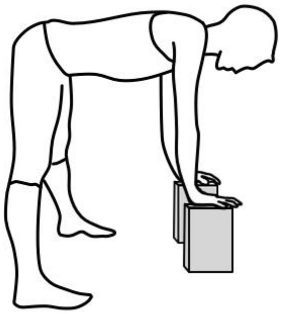

Plank and Side Plank Poses

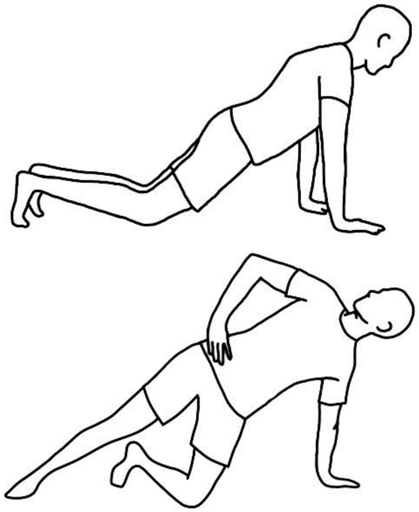

Side Hip Strengtheners

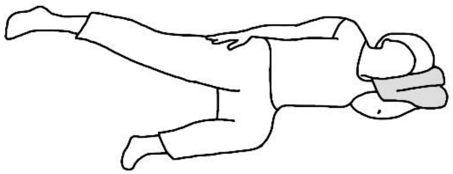

Downward Dog Pose

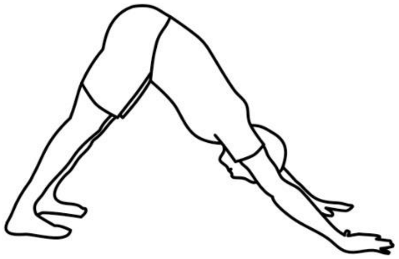

Child's Pose

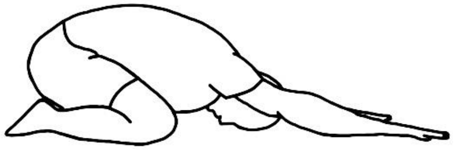

Eye of the Needle Pose

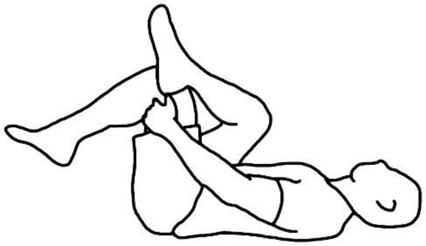

© Boston Medical Center
Veterans Back to Health Trial

Segment Three: Engaging Your Power

Options for Cool-Down Postures

Bridge Pose

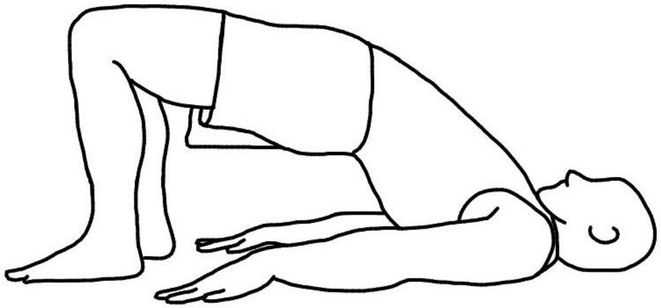

Knees Together Twist Pose

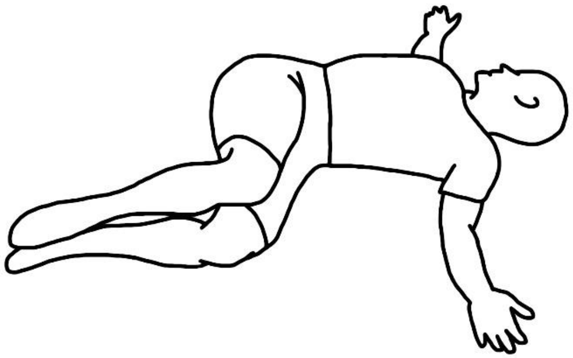

Knees to Chest Pose

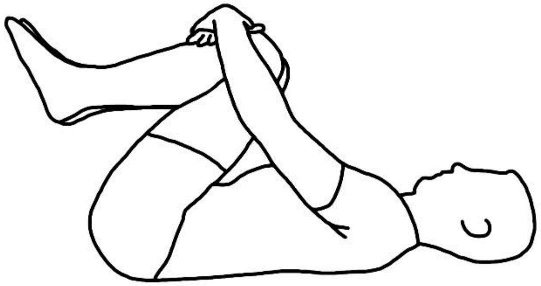

Reclining Cobbler Pose

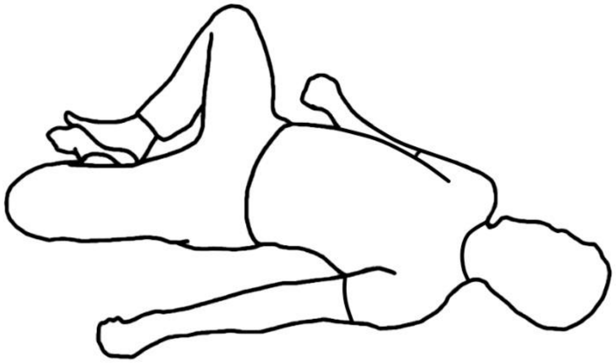

Legs up the Wall Pose

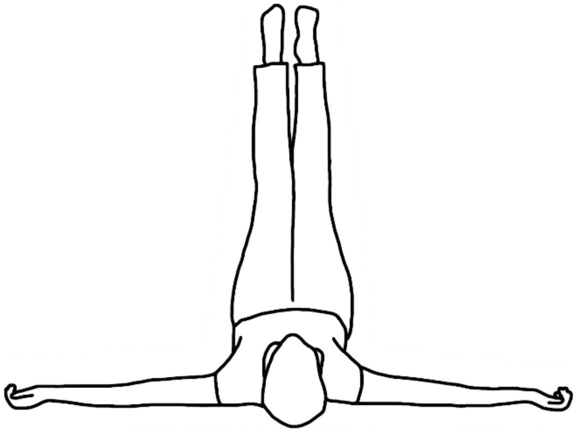

© Boston Medical Center
Veterans Back to Health Trial

Yoga Poses for Home Practice

Segment Four (Weeks 10-12): Bringing it Home

You can select a few of the following warm-ups to start your home practice:

1. Knees to Chest Pose
2. Knees Together Twist Pose
3. Big Toe Pose
4. Child's Pose
5. Mountain Pose
6. Shoulder Opener Pose

After the warm-ups, you can do a few of the below yoga postures:

1. Downward Dog Pose
2. Plank Pose
3. Side Plank Pose
4. Cobra Pose
5. Side Hip Strengtheners
6. Triangle Pose
7. Warrior I or II Pose
8. Baby Dancer Pose

After some yoga postures, finish with these cool-down poses:

1. Bridge Pose
2. Knees Together Twist Pose
3. Knees to Chest Pose
4. Reclining Cobbler Pose
5. Legs up the Wall Pose

End your practice with a short relaxation. You can choose from the following relaxation positions:

1. Final Resting Pose
2. Supported Final Resting Pose
3. Reclining Chest Opener Pose

© Boston Medical Center
Veterans Back to Health Trial

Segment Four: Bringing it Home

Options for Warm-Ups

Knees to Chest Pose

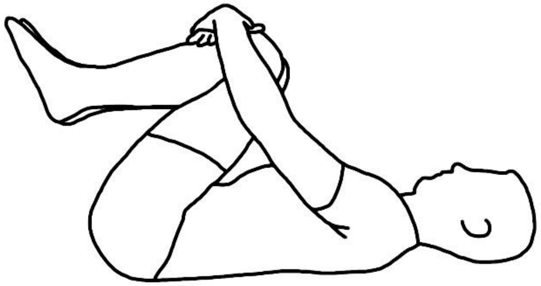

Knees Together Twist Pose

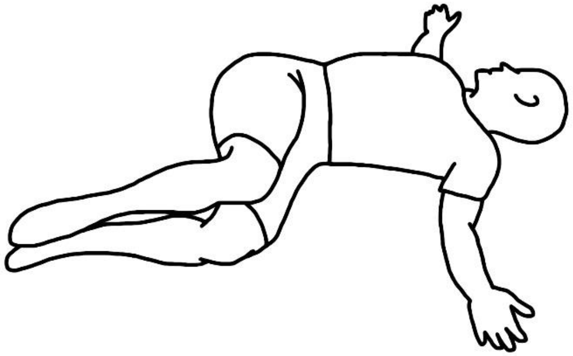

Big Toe Pose

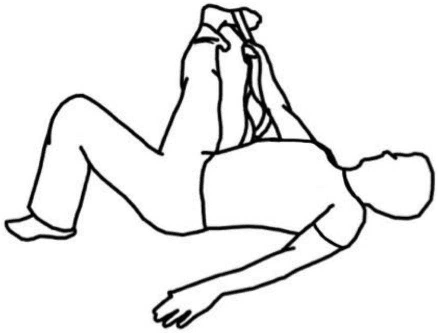

Mountain Pose

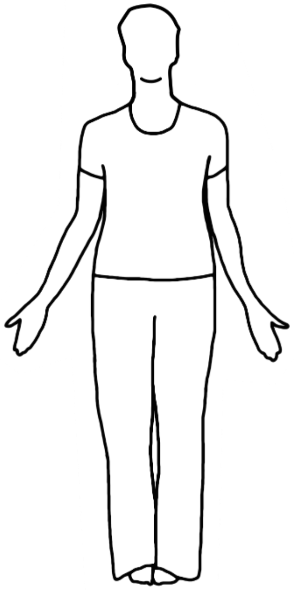

Child's Pose

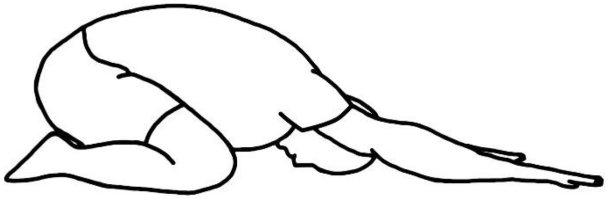

Shoulder Opener Pose

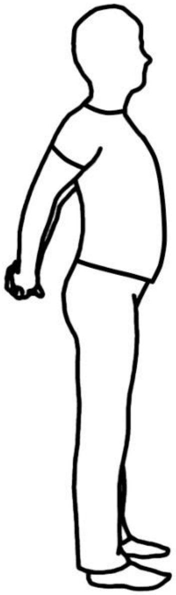

© Boston Medical Center
Veterans Back to Health Trial

Segment Four: Bringing it Home

Options for Yoga Postures

Downward Dog Pose

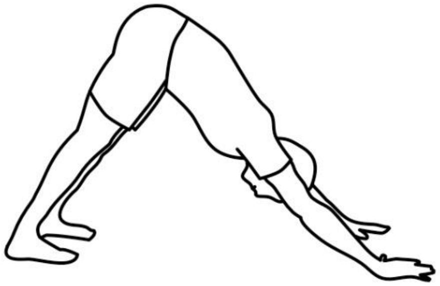

Plank Pose

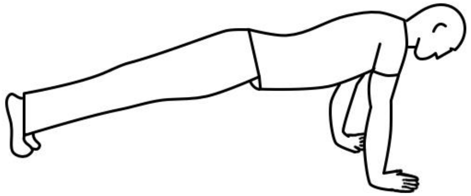

Side Plank Pose

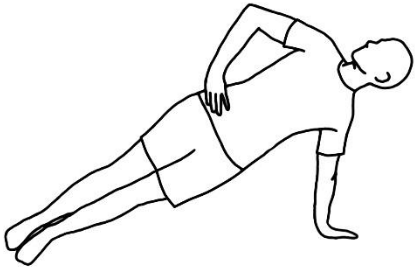

Warrior II Pose

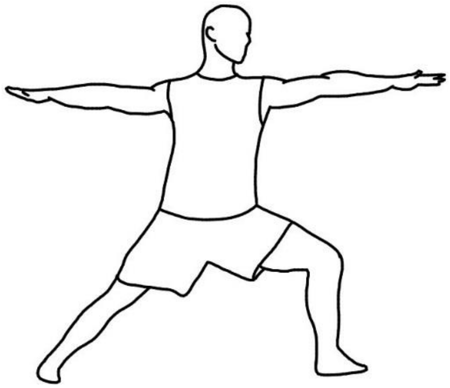

Baby Dancer Pose

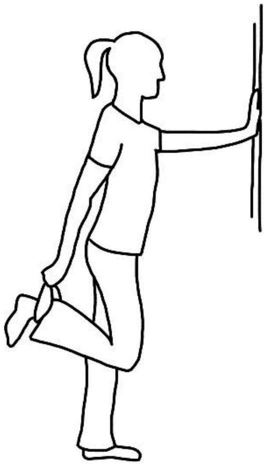

Side Hip Strengtheners

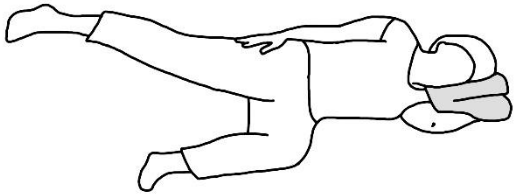

Cobra Pose

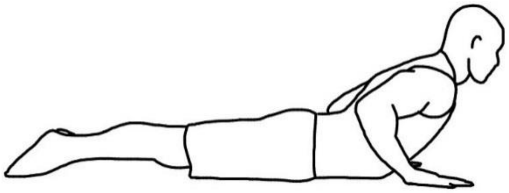

Triangle Pose

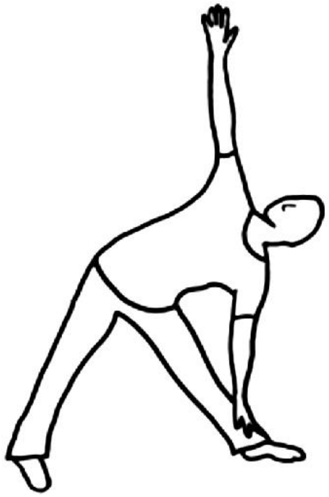

© Boston Medical Center
Veterans Back to Health Trial

Segment Four: Bringing it Home

Options for Cool-Down Postures

Knees to Chest Pose

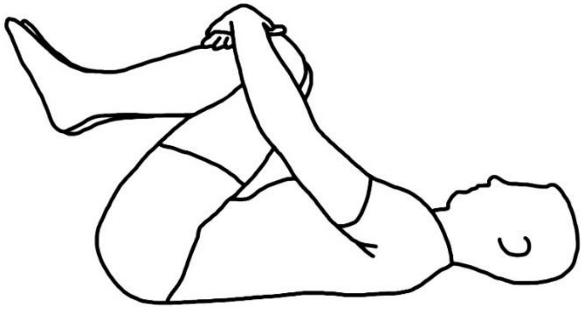

Knees Together Twist Pose

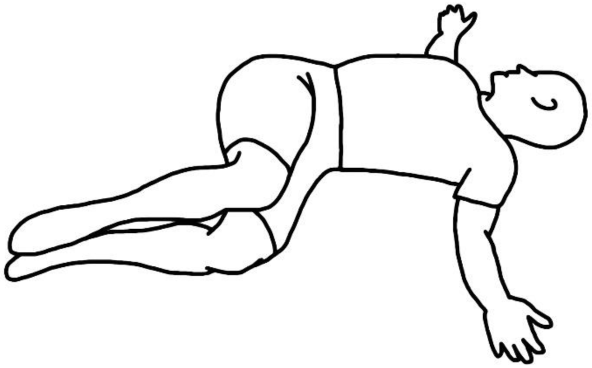

Reclining Chest Opener Pose

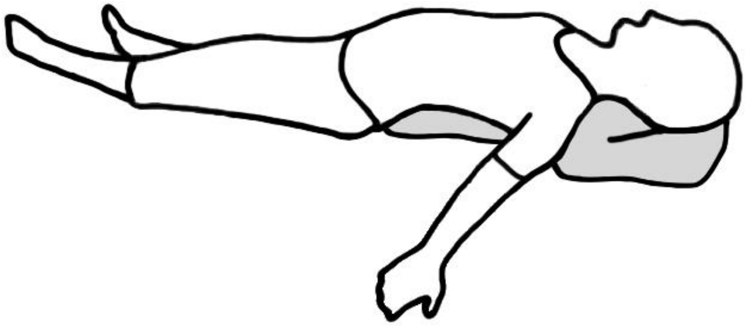

Reclining Cobbler Pose

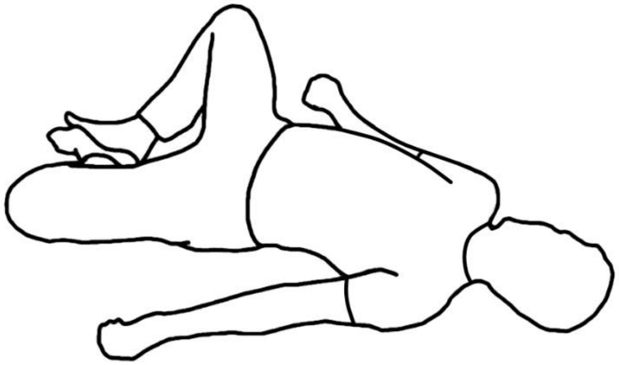

© Boston Medical Center
Veterans Back to Health Trial

Instructions for Specific Yoga Poses

Baby Dancer Pose

Instructions

1. Start by standing with your feet hip-width apart and with your left side facing a wall and your left hand on the wall (or the back of a chair).
2. Shift your weight onto your left leg and bend your right knee so that your right foot is behind you.
3. Lift your right foot with the help of a strap around your ankle and hold either the strap with your right hand.
4. Raise your left hand while still touching the wall.
5. Release your right foot and come back to Mountain Pose.
6. Repeat on the other side, with the right side of your body against the wall.

Other Options

- You can transition to doing this pose without the using a wall or a chair for balance when your body is ready.
- TO DO THE POSE SITTING IN A CHAIR: See Baby Dancer Pose in Chair (p. 23).

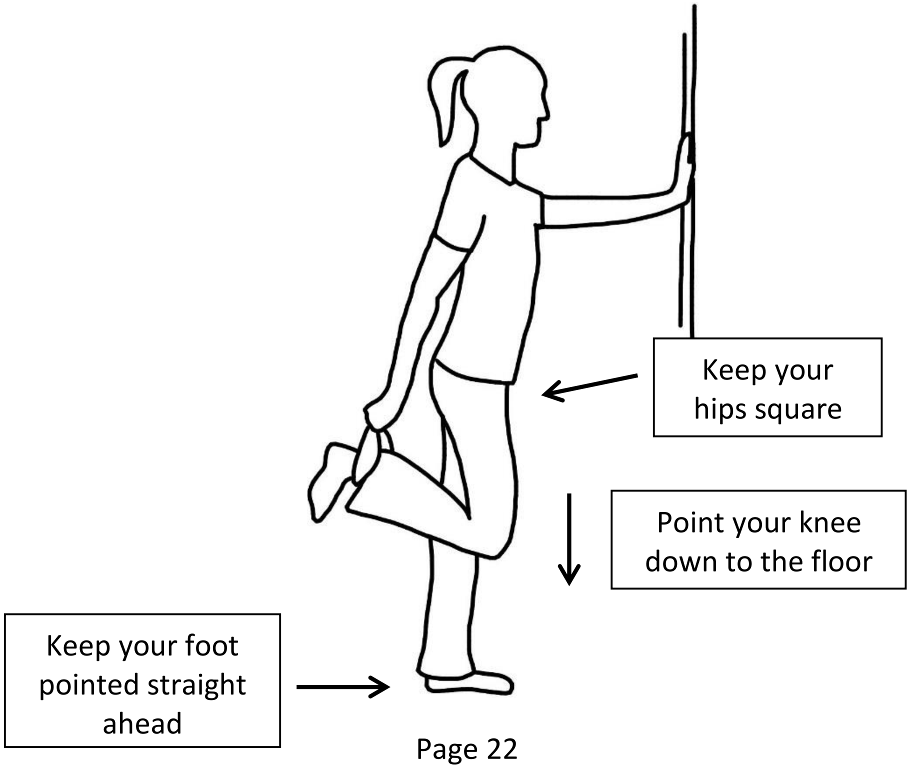

© Boston Medical Center
Veterans Back to Health Trial

Instructions for Specific Yoga Poses

Baby Dancer Pose in Chair

Instructions

1. Sit on the edge of a chair, facing to the side.
2. With one hand on the back of the chair for support, take one foot back to the side of the chair and let your knee drop down to point to the floor.
3. Hold your ankle or put a belt around your ankle to bring your foot up. You may feel the stretch in the front of your thigh.
4. Release your foot and move to the other side of the chair to repeat other side.

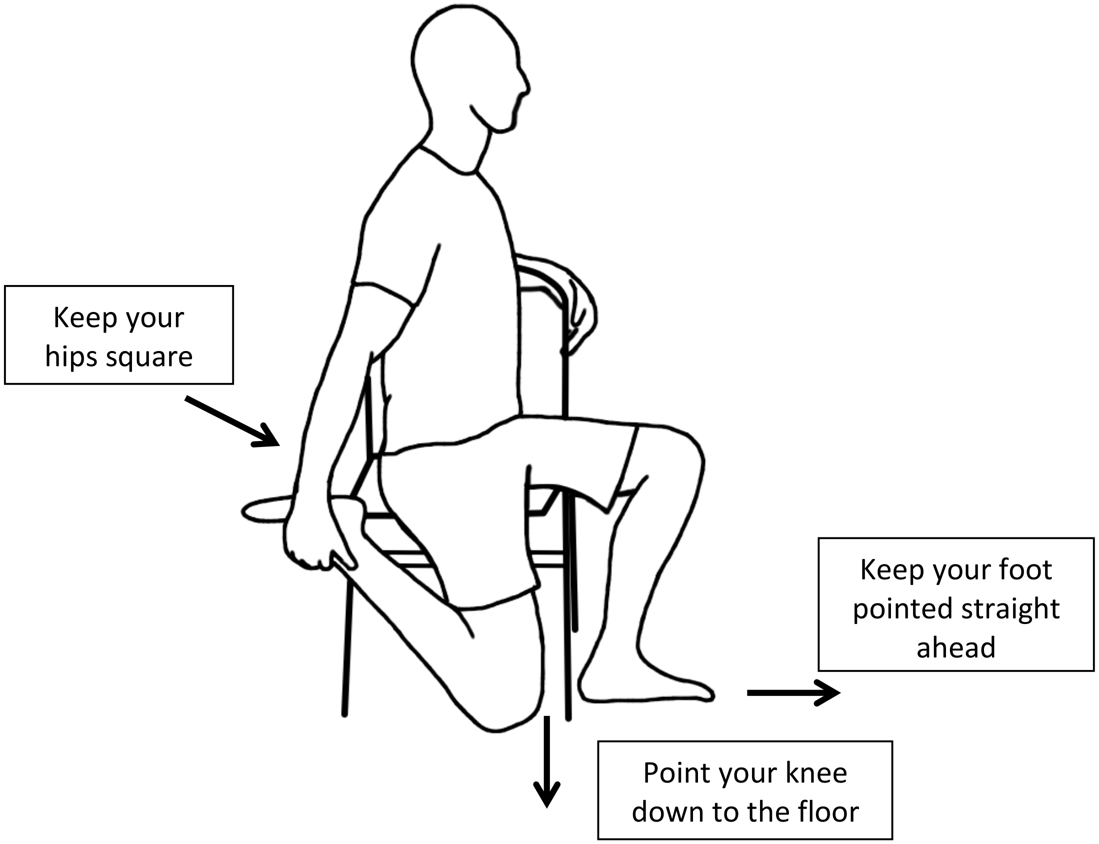

© Boston Medical Center
Veterans Back to Health Trial

Instructions for Specific Yoga Poses

Big Toe Pose

1. Lying on your back with both knees bent, loop belt around the bottom of your right foot while holding both ends of the belt with your right hand.
2. Straighten your leg as much as possible. Press your right foot up into the belt while gently pulling your arms down towards the floor.
3. Move your right leg out to the right and let your left knee move to the left if it wants to. Then bring your leg back straight.
4. Repeat, moving your leg out and up three times.
5. Bend your knee and take the belt off.
6. Repeat on your left leg.

<u>Other Options</u>

- Bend and straighten your leg, without moving leg to the side, several times using a belt for support.
- Lie on your back with your feet facing a wall or a chair with your knees bent, place one leg up on the wall or on the seat of a chair as straight as possible using the wall or chair for support.
- <u>TO DO THE POSE SITTING IN A CHAIR</u>: With your legs bent and your feet on the floor, place one leg straight up onto a block or another chair seat (no picture available).

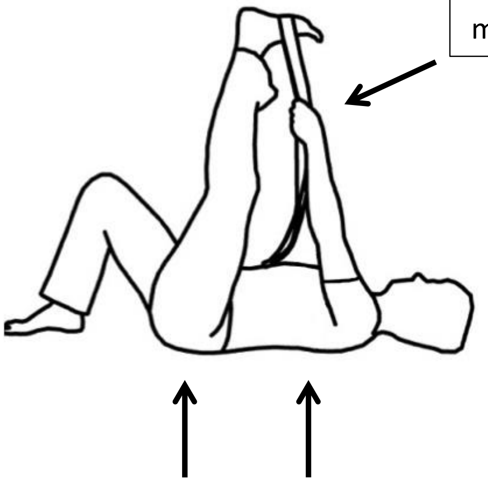

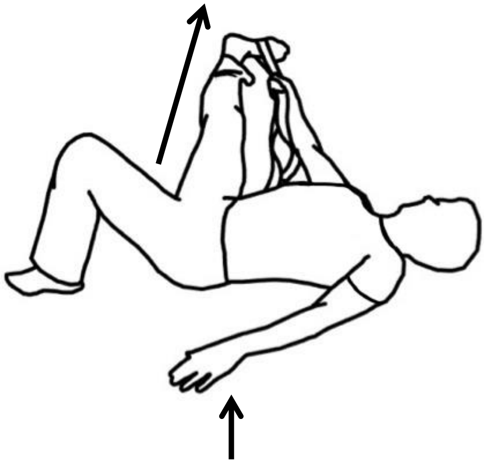

© Boston Medical Center
Veterans Back to Health Trial

Instructions for Specific Yoga Poses

Bridge Pose

<u>Instructions</u>

1. Lie on your back with your knees bent and your feet flat on the floor. Keep your knees and feet parallel.
2. Lift your hips and place a block under your tailbone for support. Keeping your hips rested on the block, lift your chest without lifting your head or shoulders.
3. To do the pose without a block, press your lower back and feet into the mat and then lift your tailbone up. Keep lifting from the back of your thighs until your back is off of the floor.
4. Reach your hands towards your ankles, holding them for stability if you can reach easily.
5. Slowly roll back down to the mat starting with your upper back.

If this pose causes increased back pain, you can keep your back on the floor and do gentle pelvic tilts instead.

<u>Other Options</u>

- Walk your feet further away from your hips if you feel discomfort in your knees.
- Place a block between your knees or a belt around your thighs to keep your thighs parallel.
- <u>TO DO THE POSE SITTING IN A CHAIR</u>: See Bridge Pose in Chair (p. 26).

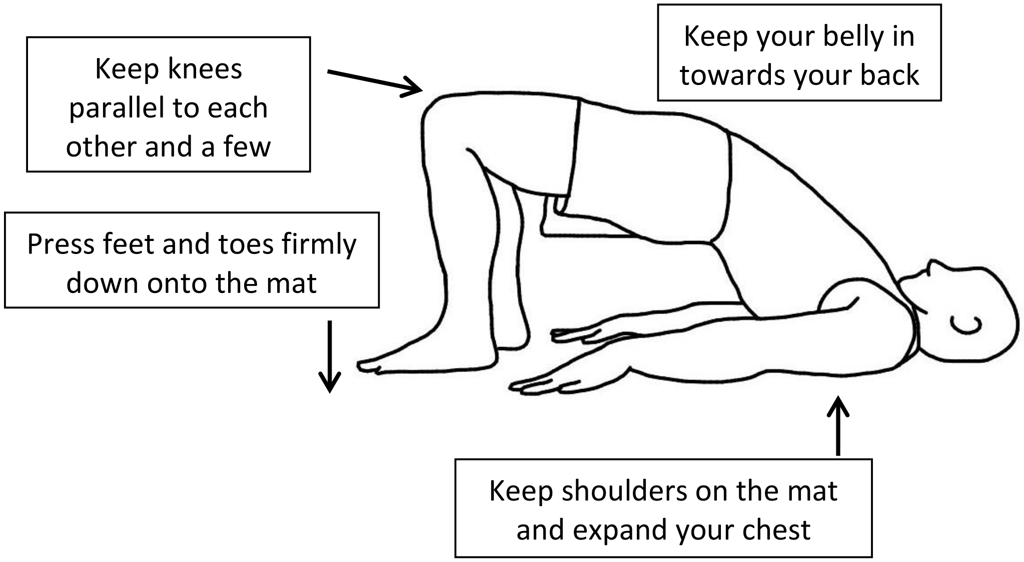

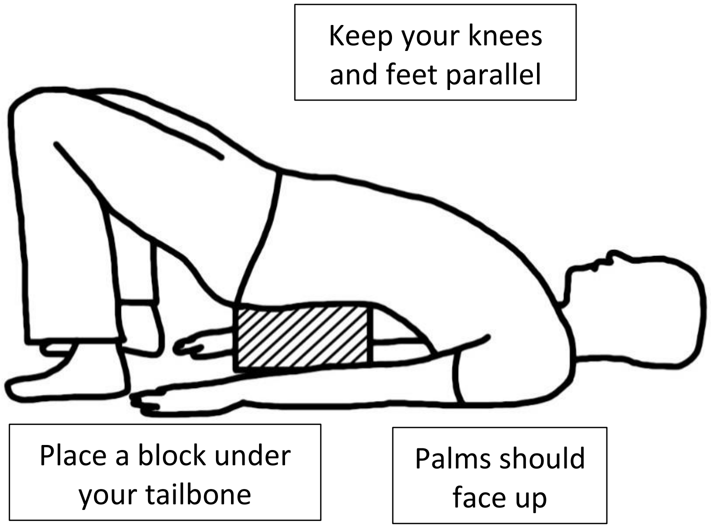

© Boston Medical Center
Veterans Back to Health Trial

Instructions for Specific Yoga Poses

Bridge Pose in Chair

Instructions

1. Sit on the edge of a chair and hold the sides of the chair. Or if you have a chair with arms, hold the arms of the chair.
2. Holding the chair, lean back and lift the hips up off the chair, making sure *not* to lift from the lower back but instead lift from backs of thighs.
3. Slowly lower back down to the chair.

If this pose causes any pain in your back, arms, or hands, you can do gentle pelvic tilts while seated in a chair instead.

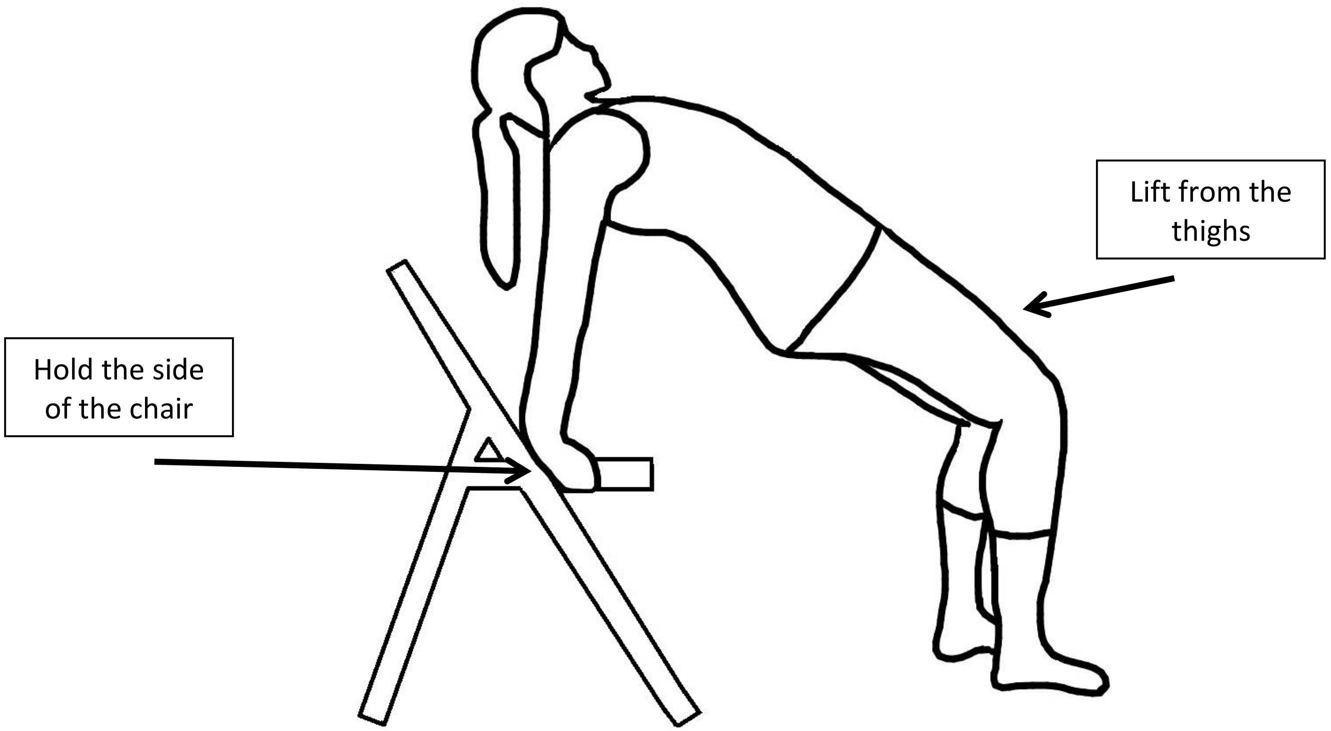

© Boston Medical Center
Veterans Back to Health Trial

Instructions for Specific Yoga Poses

Cat/Cow Pose

1. Begin on all fours with your back flat. Place your hands on the mat directly under your shoulders and your knees under your hips.
2. Press your hands into the mat and spread your fingers.
3. To move into Cat Pose, exhale and lower your chin to your chest and gently round your back.
4. Next, to move into Cow Pose, lift your head and your tailbone toward the ceiling.

To prevent injury, make sure to look forward or slightly down. Looking up to the ceiling *can* cause a neck injury if your head bends too far backwards.

Other Options

- If your wrists are uncomfortable, make a fist with both hands with your thumbs facing down.
- If your knees or hands are uncomfortable, roll the sides of the mat under your knees or the top of the mat under your hands.
- TO DO THE POSE SITTING IN A CHAIR: See Cat/Cow Pose in Chair (p. 28).

Cat

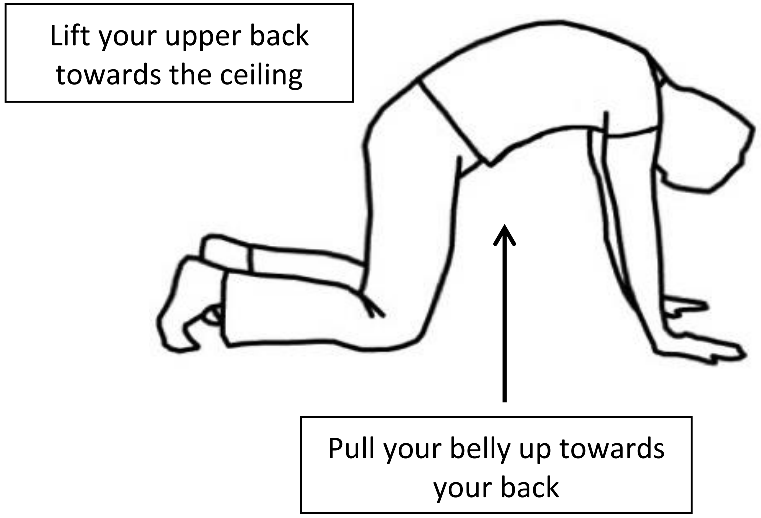

Cow

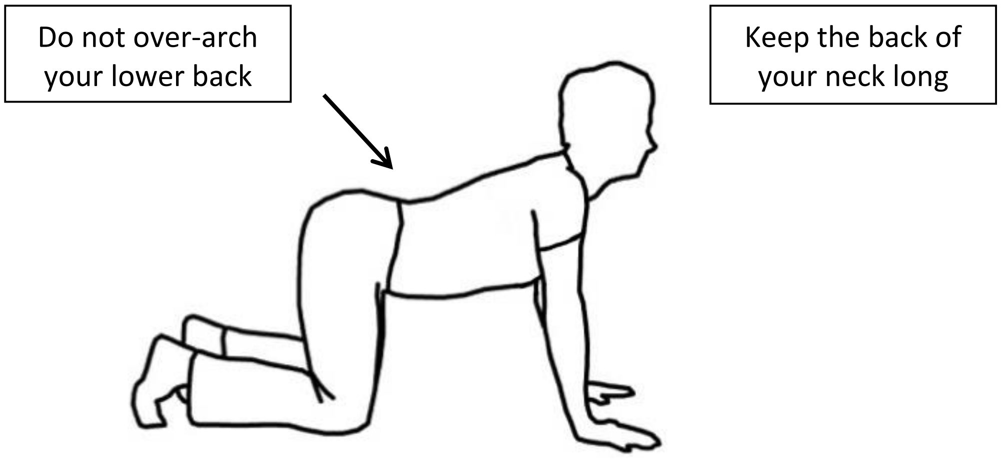

© Boston Medical Center
Veterans Back to Health Trial

Instructions for Specific Yoga Poses

Cat/Cow Pose in Chair

<u>Instructions</u>

1. Sit in a chair with your feet under your knees and your knees hip-width apart. Place hands on thighs, face down.
2. Exhale, make your back round and tuck your chin toward your chest.
3. Inhale and straighten your back.
4. Exhale, gently lift your head and chest up, slightly arching your back.
5. Repeat several times.
6. Rest, sitting straight up.

To prevent injury, make sure to look forward. Looking up to the ceiling *can* cause a neck injury if your head bends too far backwards.

Cat

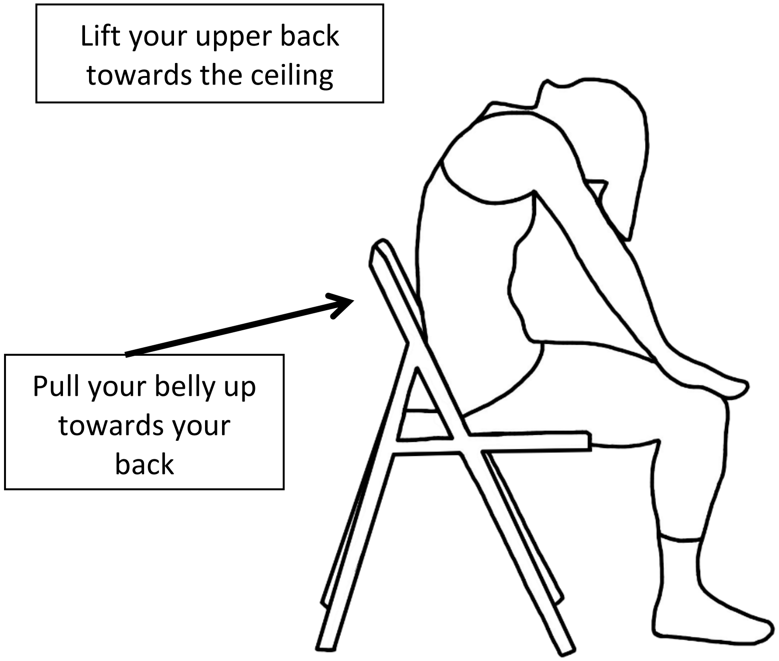

Cow

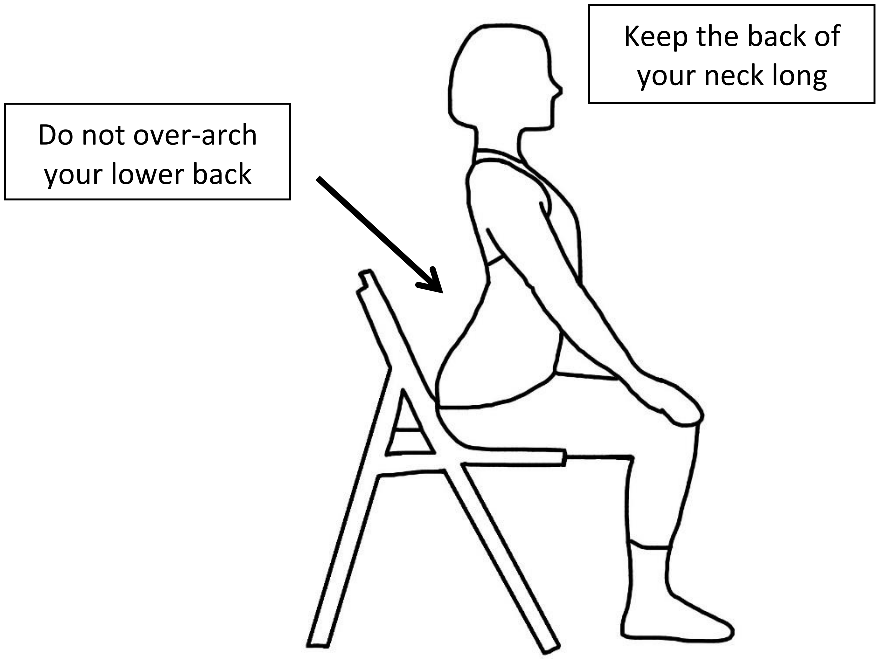

© Boston Medical Center
Veterans Back to Health Trial

Instructions for Specific Yoga Poses

Chair Dog Pose

<u>Instructions</u>

1. Place a chair so that the back (or front) of the chair is against a wall and the chair won't move.
2. Stand facing the chair with feet hip-width apart. Bend forward from hips, placing hands on the seat (or back) of the chair.
3. Stand far enough from the chair so that the legs angle back and the arms, legs and back are all straight. Move your heels towards the floor until you feel a gentle stretch. Keep your head in line with the arms.

<u>Other Options</u>

- If there is a history of shoulder injury, keep your elbows soft or bend your arms.
- With chair facing wall, place hands on back of chair for less inversion.
- <u>WALL DOG POSE</u>: Stand facing the wall with feet hip-width apart. Bend forward from hips, placing hands on the wall and walk hands up the wall slightly higher than shoulder height. Stand far enough from the wall so that the legs angle back with the arms, legs and back all straight. Move heels down towards the floor until you feel a gentle stretch while gently pressing hands into the wall. Keep your head in line with the arms (see picture below on right).

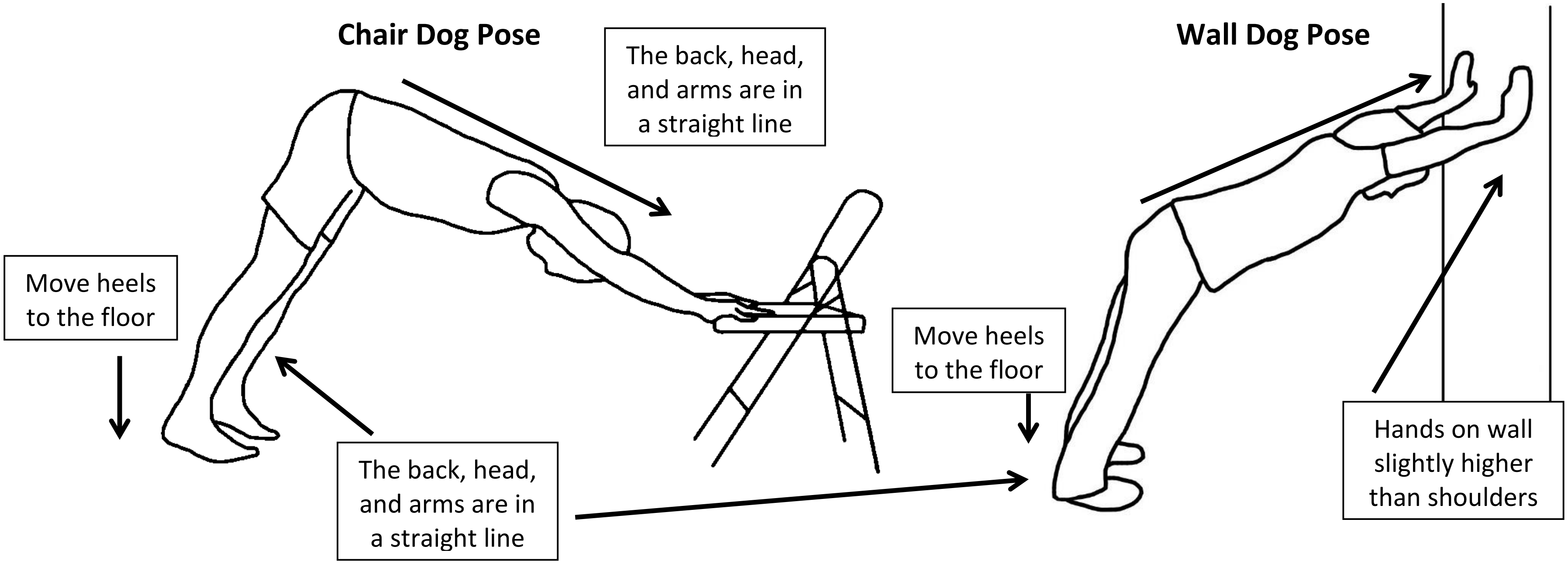

© Boston Medical Center
Veterans Back to Health Trial

Instructions for Specific Yoga Poses

Chair Pose

<u>Instructions</u>

1. Start by standing with your feet hip-width apart. Place your hands on your hips, and bend your knees as if you are about to sit in a chair.
2. Move your weight back onto your heels and bring your stomach in. Begin this pose by keeping your hands on your waist. When you are comfortable, you can raise your arms in front of you at shoulder height or raise your hands straight up over your head.
3. To end the pose, straighten your legs and bring your arms back down.

<u>Other Options</u>

- Using a wall for support: Stand with your back on a wall and your feet and knees together facing away from the wall. Bend your knees, making sure you're your knees are pointing over feet and press your lower back into wall. Slowly raise your arms up over your head.
- Face the wall, using it to support you while you do Chair Pose with your fingertips on the wall.
- <u>TO DO THE POSE SITTING IN A CHAIR</u>: See Chair Pose in Chair (p. 31).

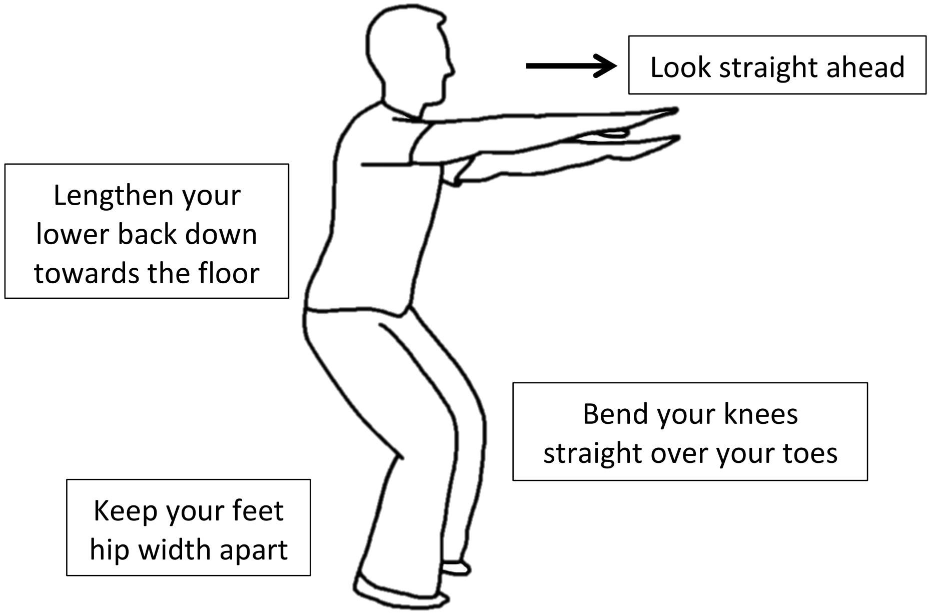

© Boston Medical Center
Veterans Back to Health Trial

Instructions for Specific Yoga Poses

Chair Pose in Chair

<u>Instructions</u>

1. Sit on the edge of a chair with your feet under your knees.
2. Lean slightly forward, moving your body weight onto your feet.
3. While remaining seated, raise arms overhead, keeping your chest lifted and your tailbone moving down towards the seat of the chair.

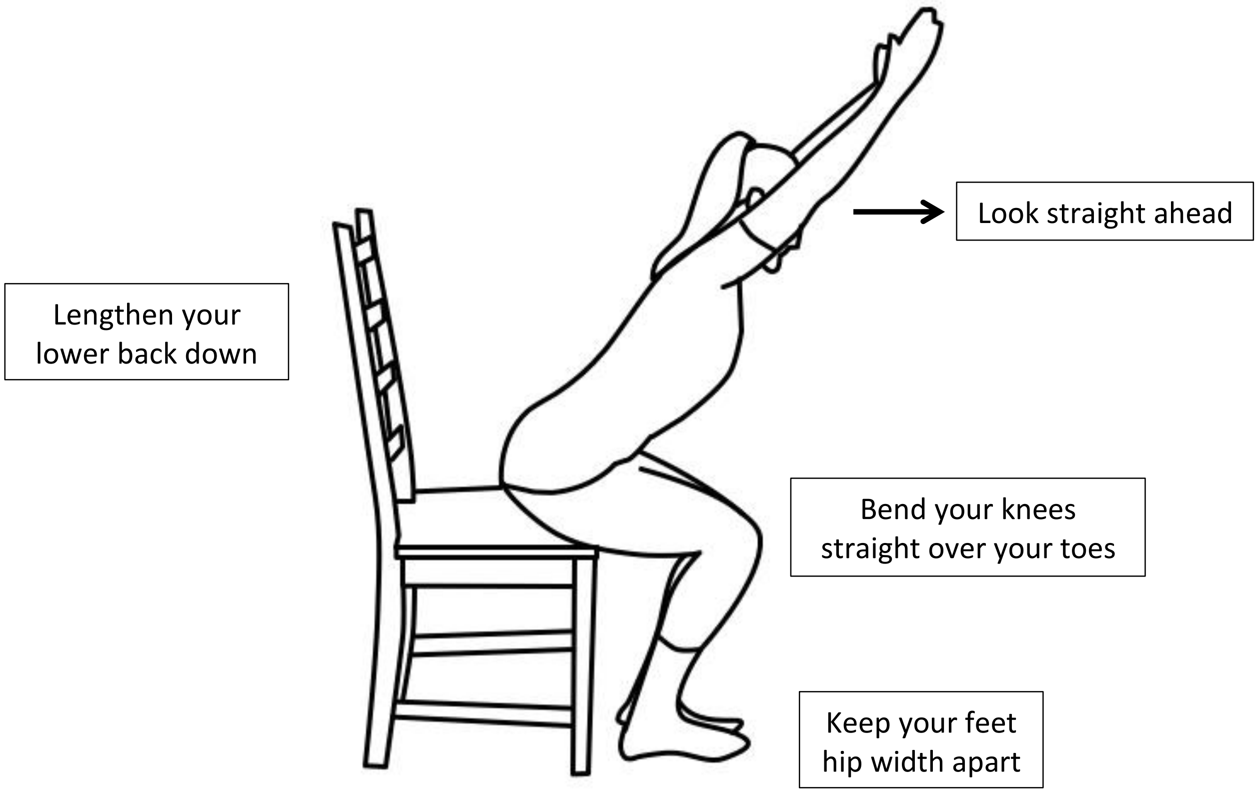

© Boston Medical Center
Veterans Back to Health Trial

Instructions for Specific Yoga Poses

Child's Pose

1. Start on your hands and knees with a flat back and with your hands under your shoulders and your knees under your hips.
2. You might want to move your knees further apart from one another, while keeping your toes together.
3. Slowly move your hips towards your heels and your forehead to the mat.
4. Stretch your arms in front keeping palms down or let arms rest by your side, reaching towards your heels, with palms up.

Other Options

- If there is intense sensation in your knees, you can place a folded blanket behind your knees. Or you can place a block or rolled blanket between your heels and hips.
- You can place a rolled blanket under your ankles for added support.
- If your feet cramp, keep your toes curled under your feet.
- TO DO THE POSE SITTING IN A CHAIR: See Child's Pose in Chair (p. 33).

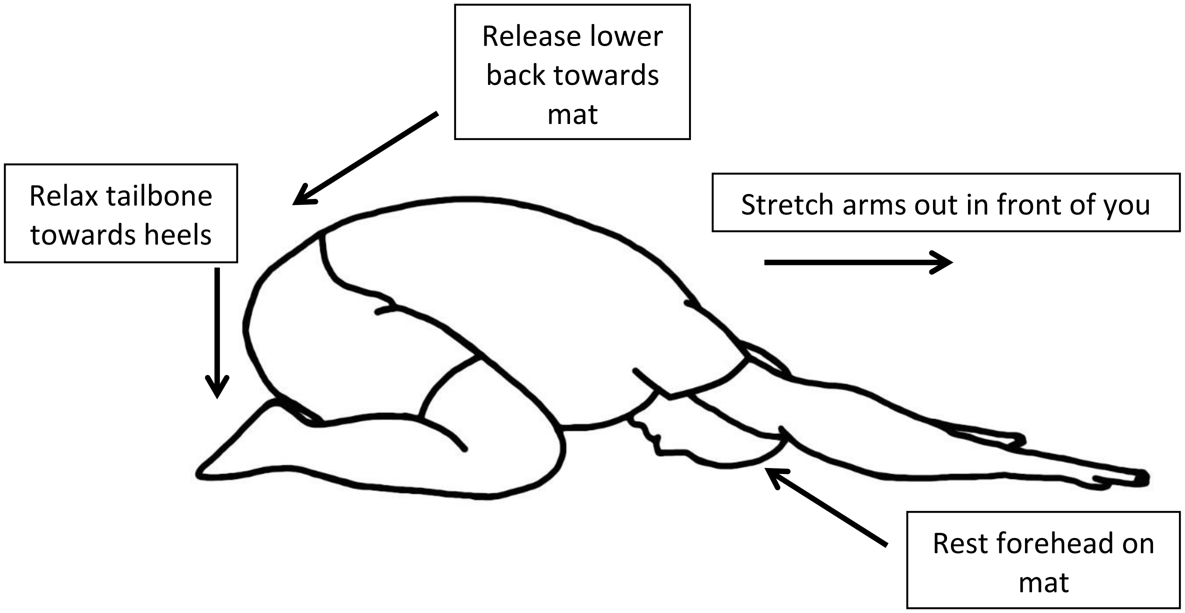

© Boston Medical Center
Veterans Back to Health Trial

Instructions for Specific Yoga Poses

Child's Pose in Chair

Instructions

1. Start by sitting in a chair, placing feet firmly on floor, hip-width apart.
2. With your hands on your thighs and your palms facing down, slowly bend forward between your legs.
3. Your hands can stay on your thighs or hang towards the floor or blocks.

Other Options

- Place another chair in front of you to rest your head on (either the back of the chair or the front of the chair).
- Place folded blankets on your lap to support your upper body (see picture below on right).

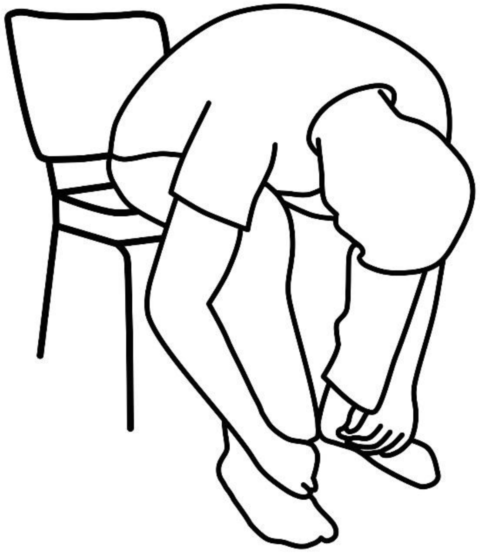

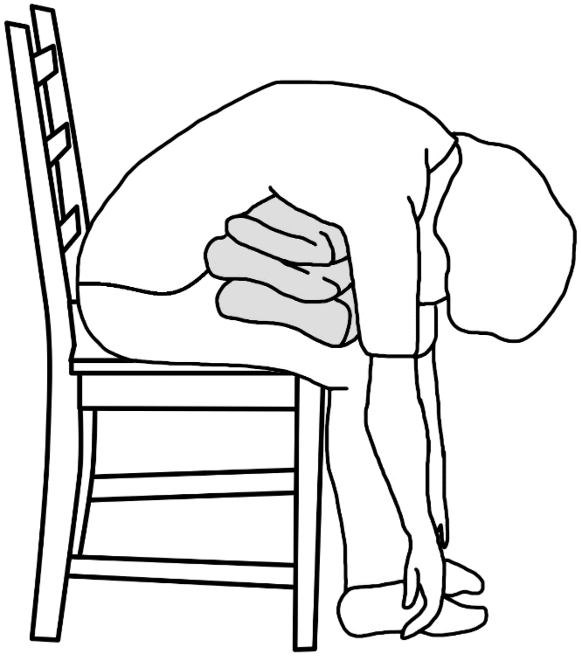

© Boston Medical Center
Veterans Back to Health Trial

Instructions for Specific Yoga Poses

Cobra Pose

Instructions
1. Lie on your stomach with your chin or forehead on the mat and your hands at shoulder level with your palms face down on the mat.
2. Point your feet behind you with your toes touching the mat.
3. Press down into the mat with your hands and lift first your head and then chest. **Look forward and not up**.

To prevent injury, make sure to look forward or slightly down. Looking up to the ceiling *can* cause neck injury if the head bends too far backwards.

Other Options
- TO DO THE POSE SITTING IN A CHAIR: Follow instructions for Sphinx Pose in Chair (p. 60).

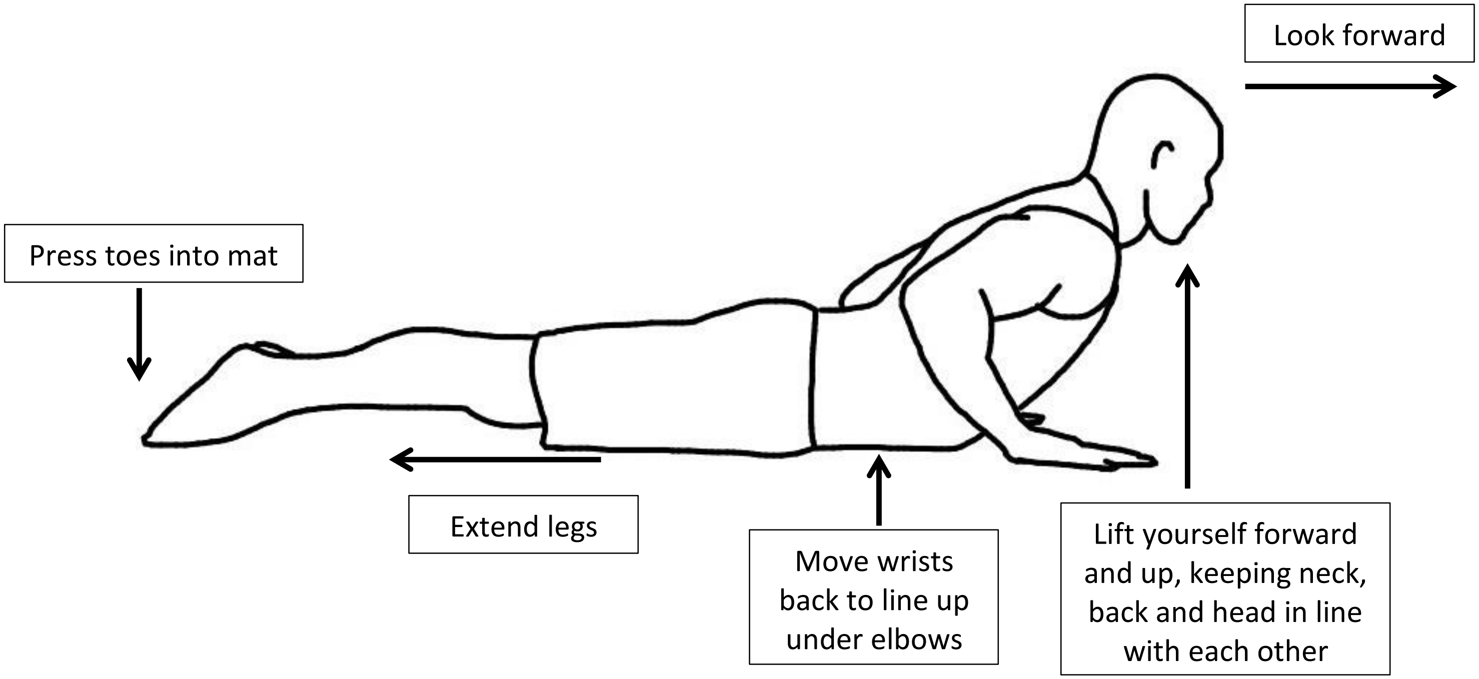

© Boston Medical Center
Veterans Back to Health Trial

Instructions for Specific Yoga Poses

Crescent Moon Pose

Instructions
1. Start by standing with your feet hip-width apart or wider and place your hands on your hips.
2. Slowly bend to the right side as your left hip moves to the left. Bend until you feel the stretch over your ribs.
3. Come back to center and bend to the left while your right hip moves to the right.

Other Options
- When your body is ready, you can move your left arm up and overhead toward the right with the palm facing down while you bent to the right, keeping your right hand on your hip. Repeat on the left side, moving right arm up towards the left and keeping left hand on hip.
- Another option with your arms is to bend to the right while holding your left wrist with your right hand. Then you can bend to the left while holding your right wrist with your left hand. This can help deepen the side stretch.
- Keep both hands on the waist while bending sideways.
- TO DO THE POSE SITTING IN A CHAIR: See Crescent Moon Pose in Chair (p. 36).

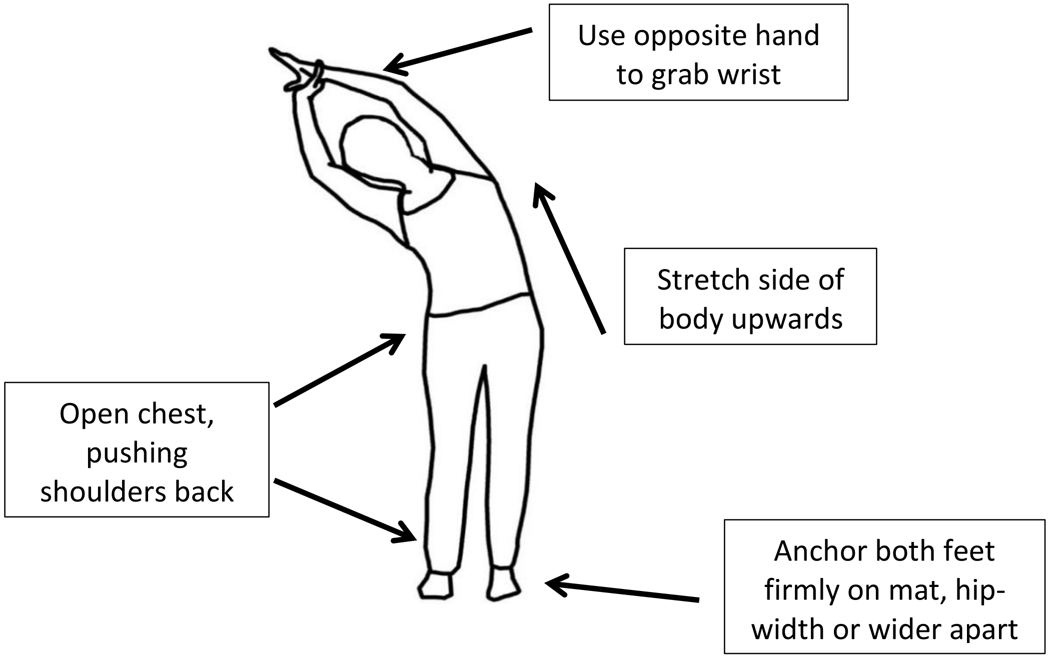

© Boston Medical Center
Veterans Back to Health Trial

Instructions for Specific Yoga Poses

Crescent Moon Pose in Chair

<u>Instructions</u>

1. Sit upright in a chair with your back straight. Place your hands on your hips or on the sides of the chair.
2. Slowly bend to the right side until you feel the stretch over your ribs.
3. Come back to center and repeat on the left.

<u>Other Options:</u>

- When your body is ready, you can move your left arm up and overhead toward the right with the palm facing down while you bent to the right, keeping your right hand on your hip or on the side of the chair. Repeat on the left side, moving right arm up towards the left and keeping left hand on your hip or on the side of the chair.
- Another options with your arms is to hold the left wrist overhead with the right hand while bending to the right. Then you can bend to the left while holding your right wrist with your left hand. This can help deepen the side stretch.

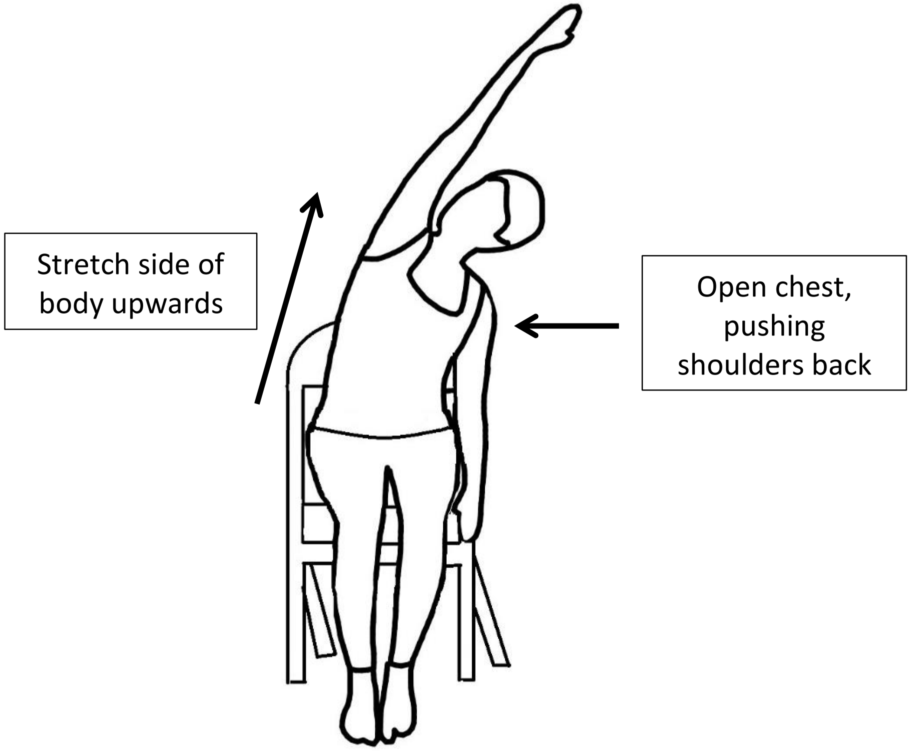

© Boston Medical Center
Veterans Back to Health Trial

Instructions for Specific Yoga Poses

Downward Dog Pose

Instructions

1. Start on your hands and knees with your hands directly under your shoulders and your knees directly under your hips.
2. Walk your hands forwards a few inches and press your palms into the mat.
3. Lift your hips and straighten your legs while pressing your heels down towards the floor.
4. When you’re ready, come back to your hands and knees.

Take caution with this pose if you have a history of head injury. Instead, you can practice Chair or Wall Dog Poses (p. 29).

Other Options

- To do this pose with your hands on a chair or wall, see instructions for Chair Dog Pose (p. 29).
- TO DO THE POSE SITTING IN A CHAIR: Sit on edge of a chair, extend both of your legs out, hips weigh apart and raise both arms straight up over head, shoulder distance apart.

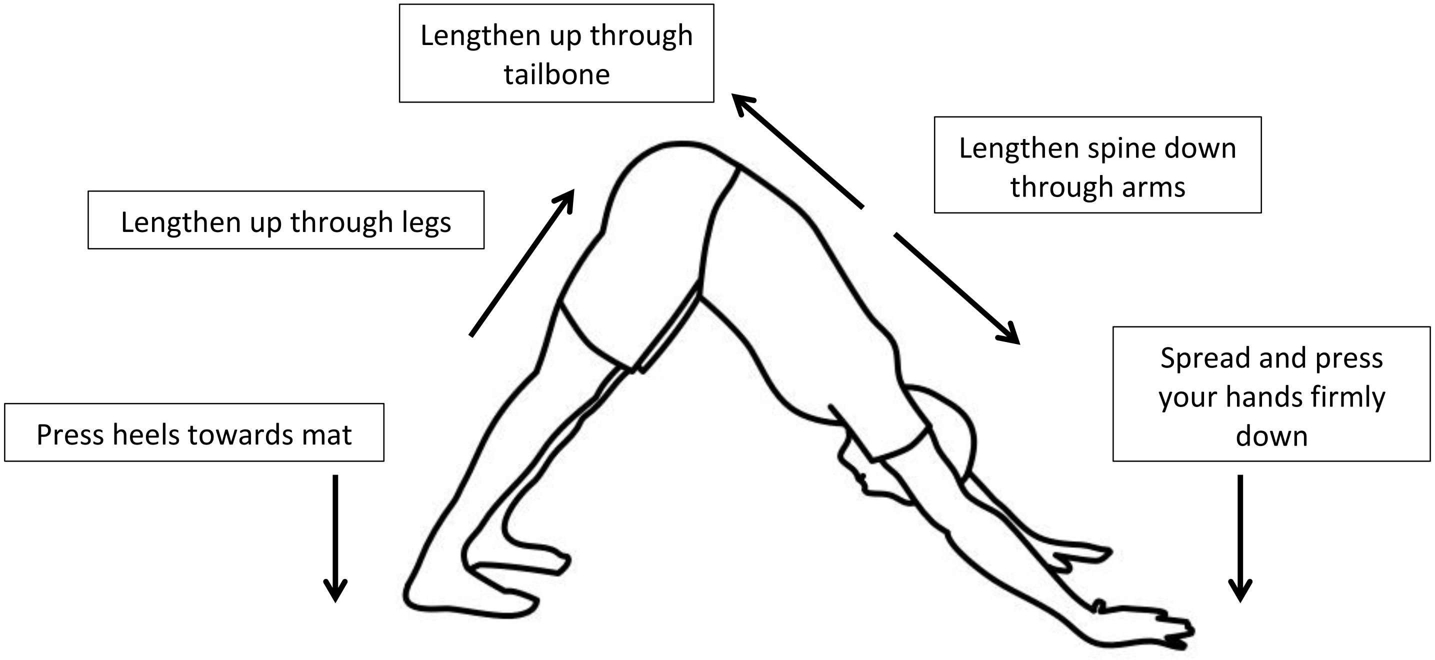

© Boston Medical Center
Veterans Back to Health Trial

Instructions for Specific Yoga Poses

Eye of the Needle Pose

Instructions

1. Starting by lying on your back with both feet on the floor, place your right ankle on your left knee and flex your right foot (as opposed to pointing the toe).
2. With your right hand, gently push your right thigh, just below your right knee, away from your head. Keep your hips, spine, and head on the floor and relax your neck. This may be enough stretch for you.
3. For a deeper stretch, clasp your hands behind your left thigh and hug it in toward your torso, keeping your head on the floor.
4. Hold for a minute, and repeat on the other side with your left ankle on your right knee.

Other Options

- TO DO THE POSE SITTING IN A CHAIR: See Eye of the Needle Pose in Chair (p. 39).

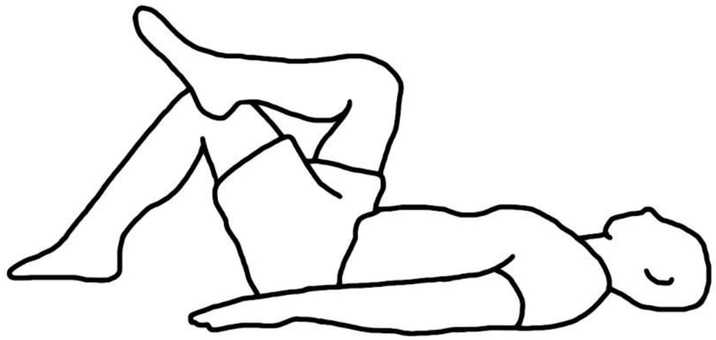

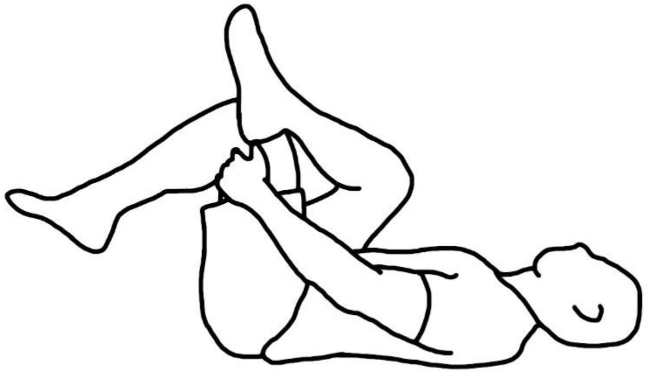

© Boston Medical Center
Veterans Back to Health Trial

Instructions for Specific Yoga Poses

Eye of the Needle Pose in Chair

<u>Instructions</u>

1. Sit in a chair with your feet flat on the floor, hip-width apart.
2. Bring your right foot up and turn your leg out to place your right ankle on your left thigh just above your knee. This may be enough of a stretch in your hips.
3. For an additional stretch, gently press down on your right thigh while sitting up straight.
4. Move right foot back to floor. Repeat with left ankle on right thigh.

<u>Other Options</u>

- For an even deeper stretch, lean forward while keeping your back and neck straight.

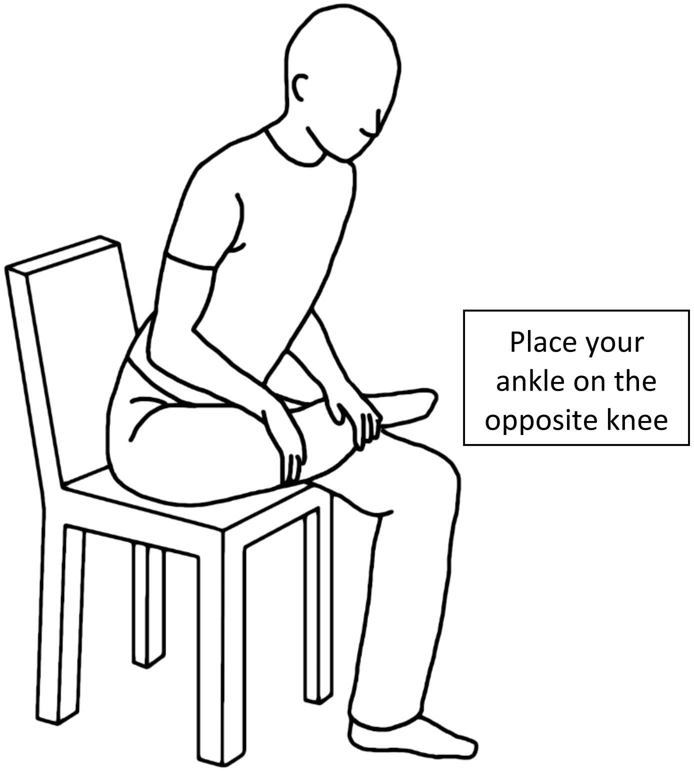

© Boston Medical Center
Veterans Back to Health Trial

Instructions for Specific Yoga Poses

Final Resting Pose

Instructions

1. Lie on your back with your legs slightly wider than hip-width and your arms slightly away from your body with palms facing up.
2. Relax your whole body including your face and let your body be heavy.
3. Relax in this pose or another comfortable resting position at the end your practice for at least 5 minutes.

Other Options

- Cover yourself with a blanket.
- If your back is uncomfortable, you can place a rolled blanket under knees. You can also place your lower legs on a chair seat.
- Side relaxation: Lie on your side with a blanket under your head, another blanket under your top arm and another between your knees.
- Belly Relaxation: Lie on your belly, with your feet slightly turned in toward each other and your hands or a blanket folded under your head.
- TO DO THE POSE SITTING IN A CHAIR: Sit on a chair with your spine supported with a rolled blanket behind your back and a blanket over your legs (no picture available).

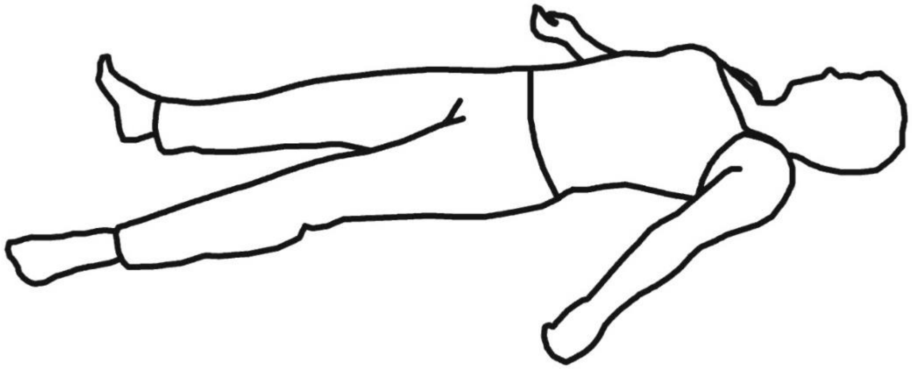

Final Resting Pose without support

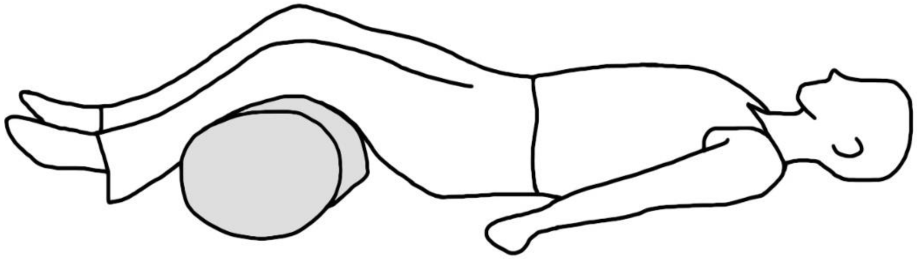

Final Resting Pose with support

© Boston Medical Center
Veterans Back to Health Trial

Instructions for Specific Yoga Poses

Forward Bend Pose

Instructions

1. To do the pose facing a wall, step your right leg forward towards the wall while stepping your left foot back away from the wall. Lower your back so that it is as parallel to the floor as possible.
2. After you are comfortable with this pose at the wall, try doing the same thing with your hands on the back of a chair, the seat of a chair, or a table instead of the wall.

If you have a history of head injury, be careful not to lower your head too far.

Other Options

- You can place your hands on the back of a chair or a table for a stretch that is deeper than doing this pose at a wall.
- TO DO THE POSE SITTING IN A CHAIR: See Forward Bend Pose in Chair (p. 42).

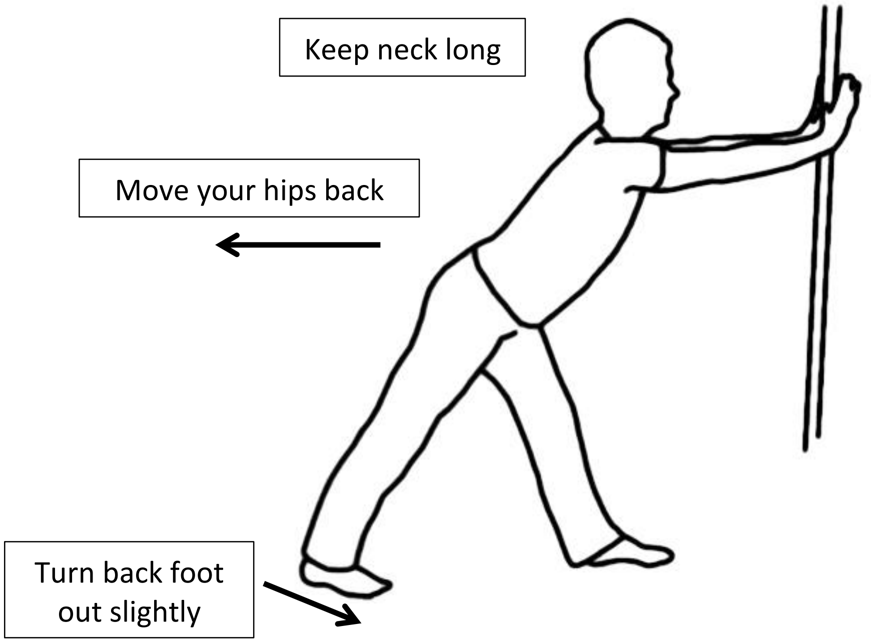

© Boston Medical Center
Veterans Back to Health Trial

Instructions for Specific Yoga Poses

Forward Bend Pose in Chair

Instructions

1. Sit in a chair and extend one leg straight forward with your heel on the floor. Keep your other leg bent with your foot on the floor.
2. Exhale, bend forward at your hips until there is a feeling of a stretch in the back of your leg. Inhale and sit back up.
3. Repeat and hold for 3 breaths.
4. Repeat on other side.

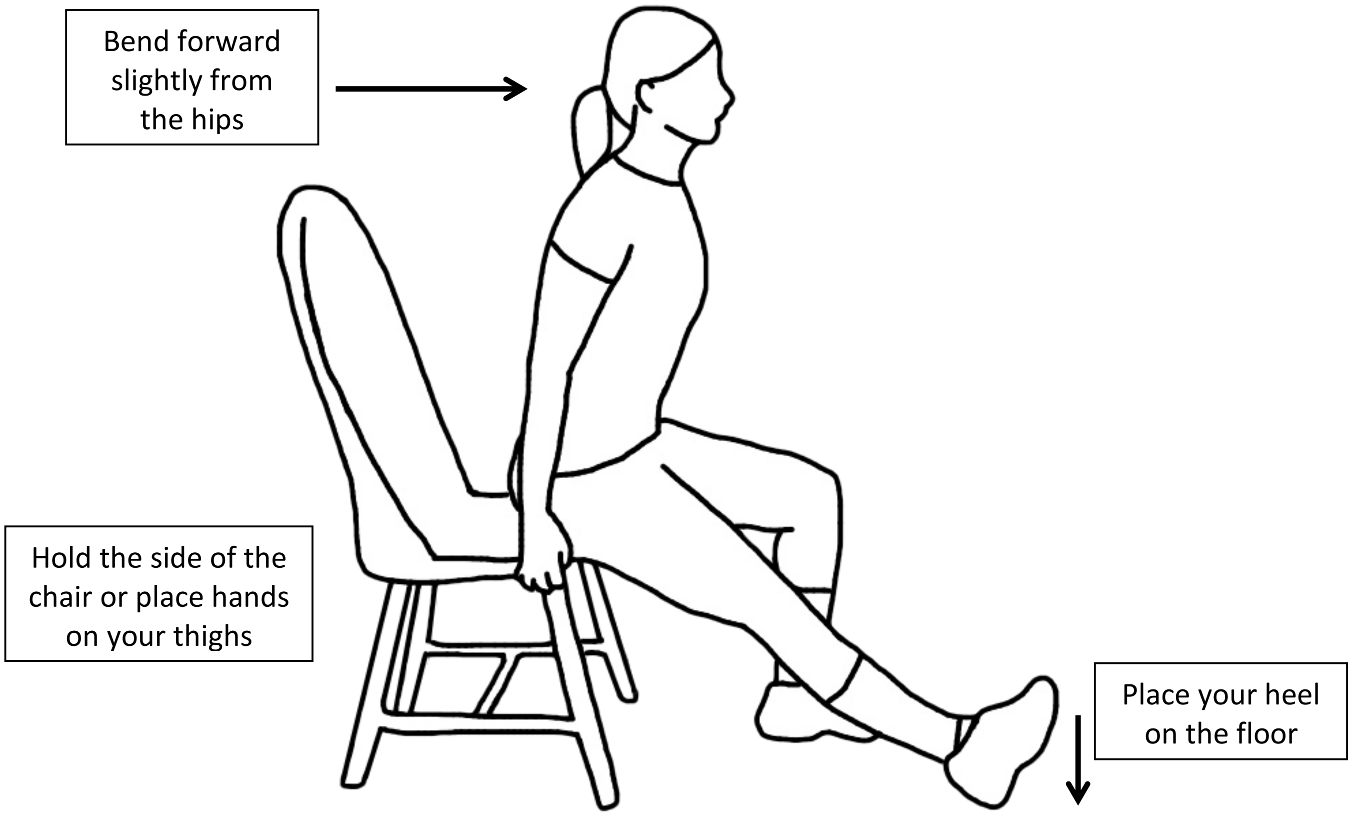

© Boston Medical Center
Veterans Back to Health Trial

Instructions for Specific Yoga Poses

Knees to Chest Pose

1. Lie on your back with your knees bent.
2. Lift one knee up to your chest while holding on to that leg around your shin or thigh.
3. Press your back into the ground.
4. Repeat with your other knee.
5. Repeat bringing both knees to your chest.

<u>Other Options</u>

- Use a belt around your shin or thigh if you cannot reach them with your hands.
- If you cannot bring your knees to chest with your hands or a belt, place feet on a wall or a chair.
- <u>TO DO THE POSE SITTING IN A CHAIR</u>: See Knee to Chest Pose in Chair (p. 44).

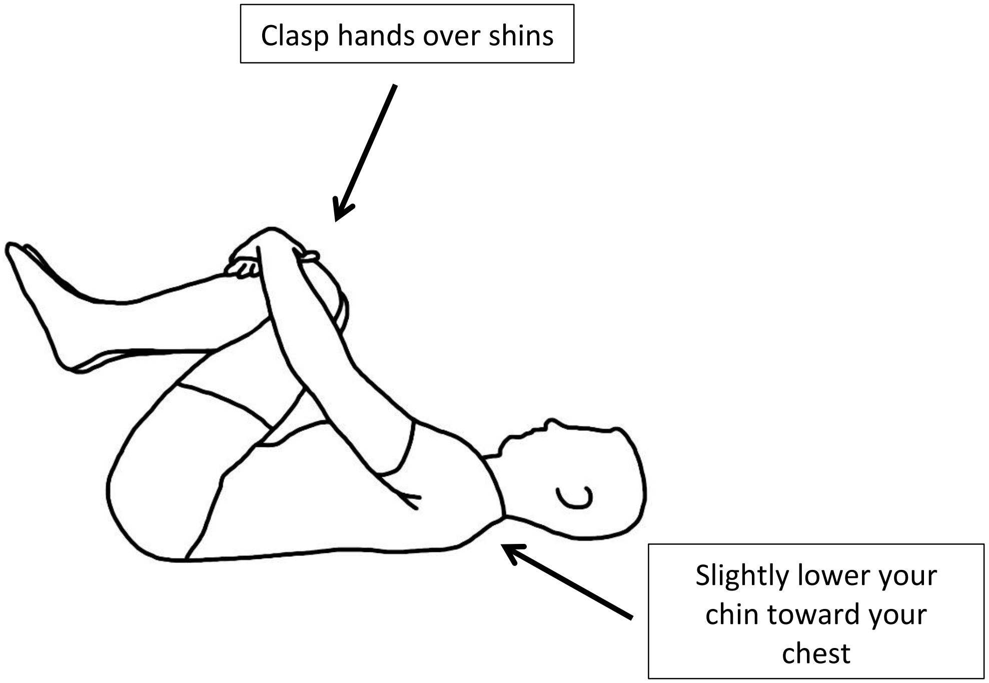

© Boston Medical Center
Veterans Back to Health Trial

Instructions for Specific Yoga Poses

Knees to Chest Pose in Chair

Instructions

1. Sit in a chair with your feet flat on the floor.
2. Raise one knee, holding your leg up with your hands or with a belt under the thigh.
3. Repeat with the other leg, holding your leg up.
4. Alternate bringing knees up towards chest.

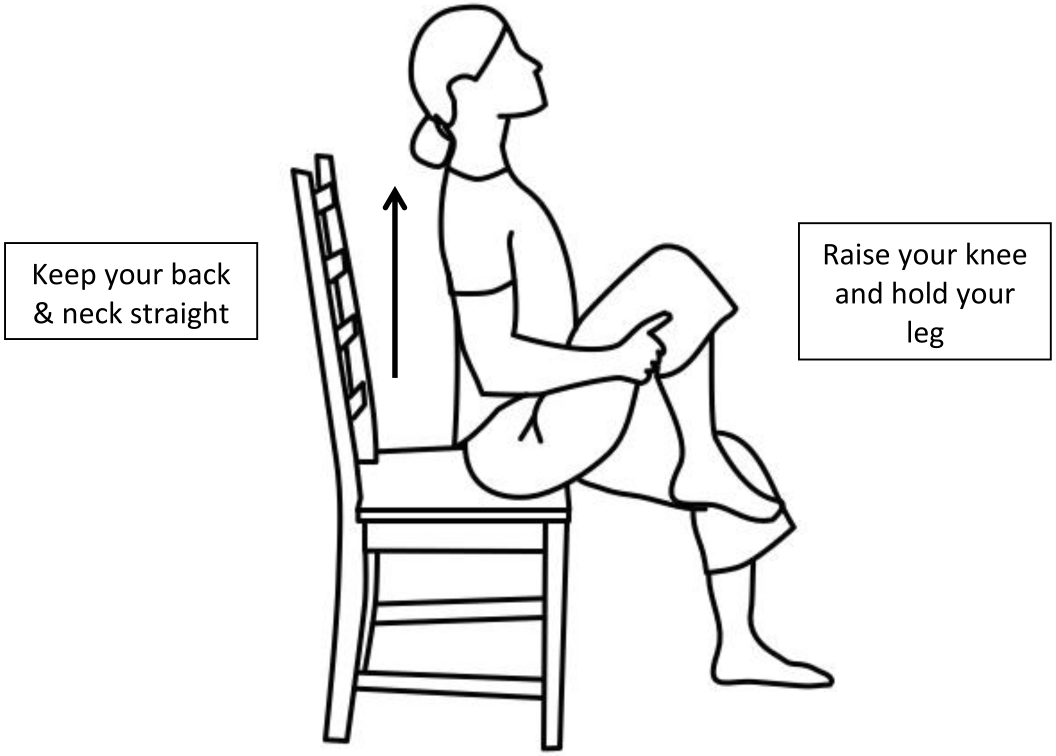

© Boston Medical Center
Veterans Back to Health Trial

Instructions for Specific Yoga Poses

Knee Together Twist Pose

Instructions

1. Begin by lying on your back.
2. Bring both knees to your chest and then lower them towards the floor on the left, keeping your right shoulder on the mat.
3. Bring your knees back to the middle. Move your knees down towards the floor on the right, keeping your left shoulder on the mat.
4. For warm-up, repeat three times moving between the left and right sides.
5. For cool-down, hold the pose and come back to center.

Other Options

- If your shoulder does not stay down on the floor, place a blanket or block under your knees on each side so they do not go as far towards the floor.

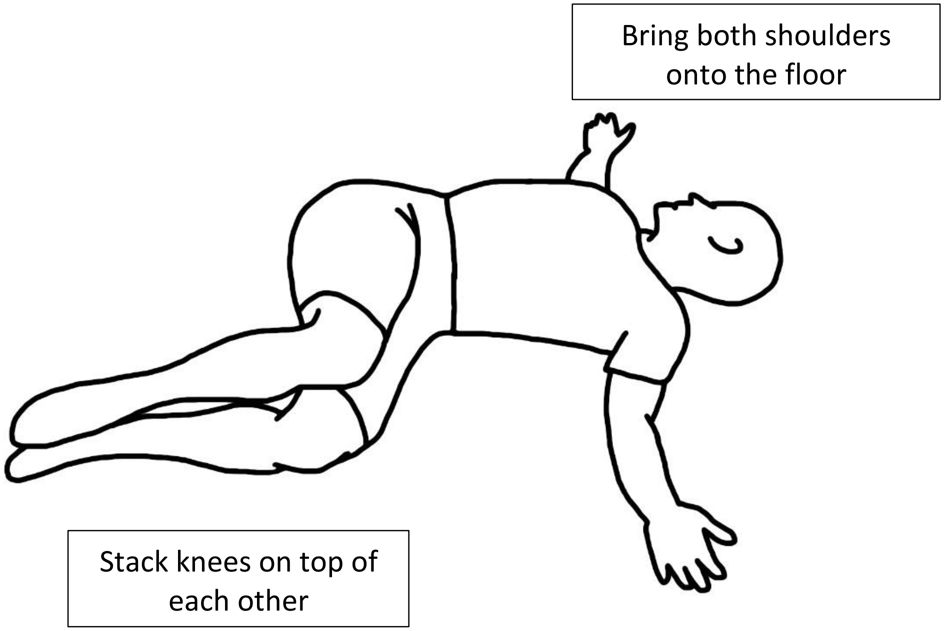

© Boston Medical Center
Veterans Back to Health Trial

Instructions for Specific Yoga Poses

Legs up the Wall Pose

Instructions

1. Begin by sitting with your left side against the wall.
2. Gently turn your body to the left and bring your legs up onto the wall. Use your hands for balance during this movement.
3. Lower your back to the floor and lie down. Rest your shoulders and head on the floor. Let your arms rest open at your sides with your palms facing up.
4. Gently shifting your weight from side-to-side, scoot your buttocks close to the wall. It is okay if your buttocks don't touch the wall, as long as you are in a comfortable position. Let your hips relax, dropping toward the floor.
5. To sit back up, slowly scoot yourself away from the wall and gently slide your legs down to the right side. Use your hands to help press yourself back up into a seated position.

Other Options

- You can place a pillow or rolled blanket under your lower back for extra support. Shift your lower back onto the support before bringing your legs up the wall. Use your hands for balance during this movement.
- For greater support under your neck, place a small, rolled towel beneath the back of your neck.
- If you would prefer, this pose can be done resting your legs on the seat of a chair instead of a wall. With the chair facing you, follow the instructions above but bend your knees and place your calves on the seat of the chair. Keep your knees straight above your hip joint.

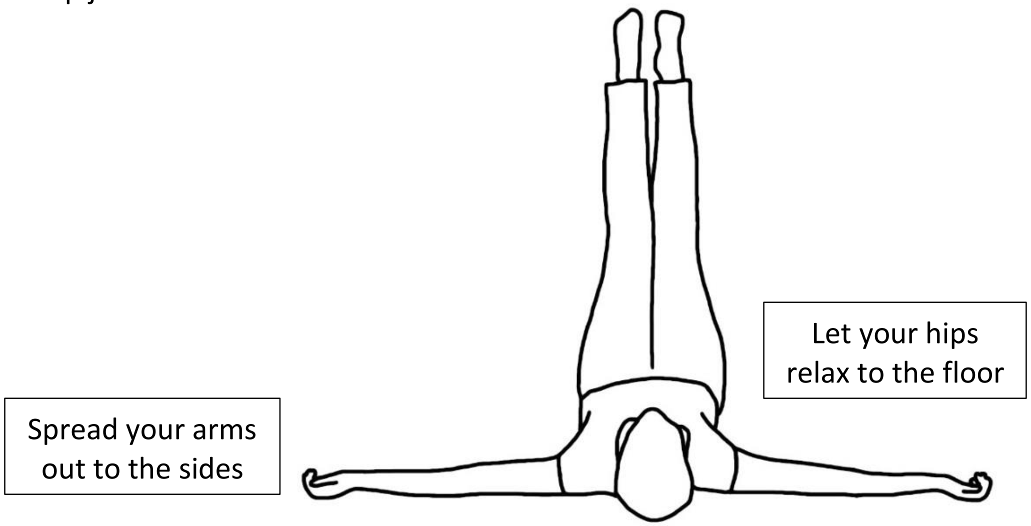

© Boston Medical Center
Veterans Back to Health Trial

Instructions for Specific Yoga Poses

Locust Pose

Instructions

1. Lie on your stomach with your arms folded in front of you and your forehead on the back of your hands.
2. Legs Only: Start by raising one leg at a time. After you have tried this a few times, you can raise both legs together.
3. Upper Body Only: Lower your legs, and then with your arms down by the sides of your body and your palms facing up, raise your head, chest and arms, while pressing your legs down.
4. After you are more comfortable with this pose, you can try to lift your head, upper chest, arms and legs off the mat at the same time.
5. To get out of the pose, slowly release and lower your body back to the mat.

To prevent injury, make sure to look forward or slightly down. Looking up to the ceiling *can* cause neck injury if the head bends too far backwards.

Other Options

- While lifting legs only, place pillows under pelvis if needed.
- Stand facing a chair: Bend forward and hold onto the seat of a chair. Extend one leg back, inhale, slowly raise the leg straight up off the floor. Exhale, bring it down. Repeat on the other side.
- TO DO THE POSE SITTING IN A CHAIR: Place your feet firmly on the floor with your arms by your sides and allow your shoulders to drop down away from your ears. Roll your shoulders back to open up your chest (no picture available).

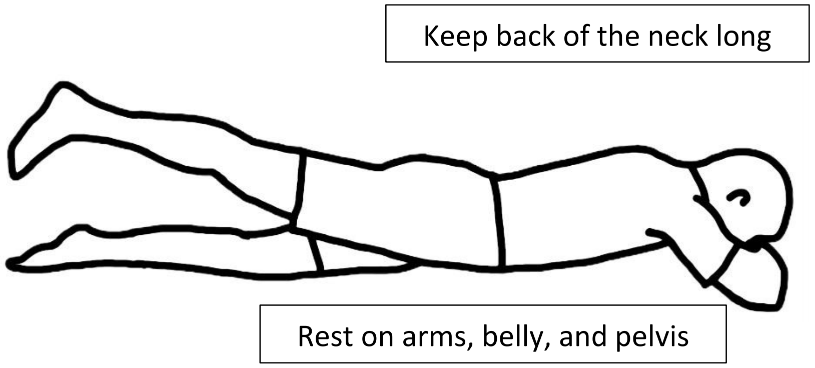

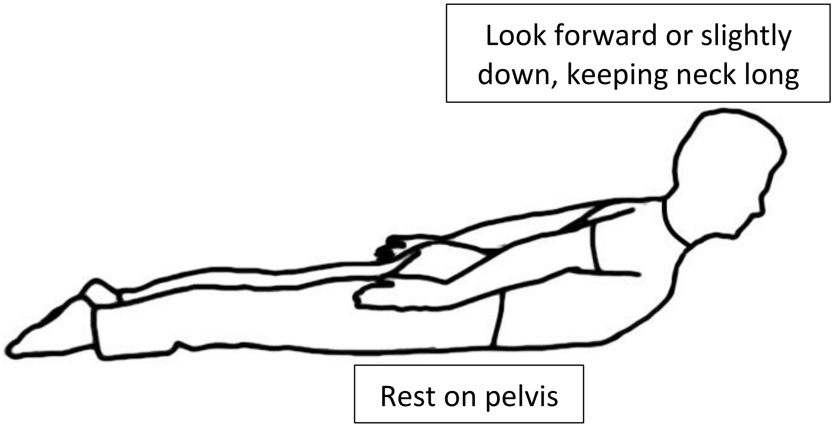

© Boston Medical Center
Veterans Back to Health Trial

Instructions for Specific Yoga Poses

Mountain Pose

Instructions

1. Stand straight with your feet facing forward and hip distance apart. Let your weight bear equally on all parts of your feet.
2. Allow your back to stretch and lengthen while looking forward. Keep your head over your shoulders, your shoulders over your hips and your hips over your ankles.

Other Options

- Stand with back against wall.
- Lie on the floor with your legs straight and your feet pressing into wall. With your arms down by your sides, press your shoulders down toward the floor.
- TO DO THE POSE SITTING IN A CHAIR: See Mountain Pose in Chair (p. 49).

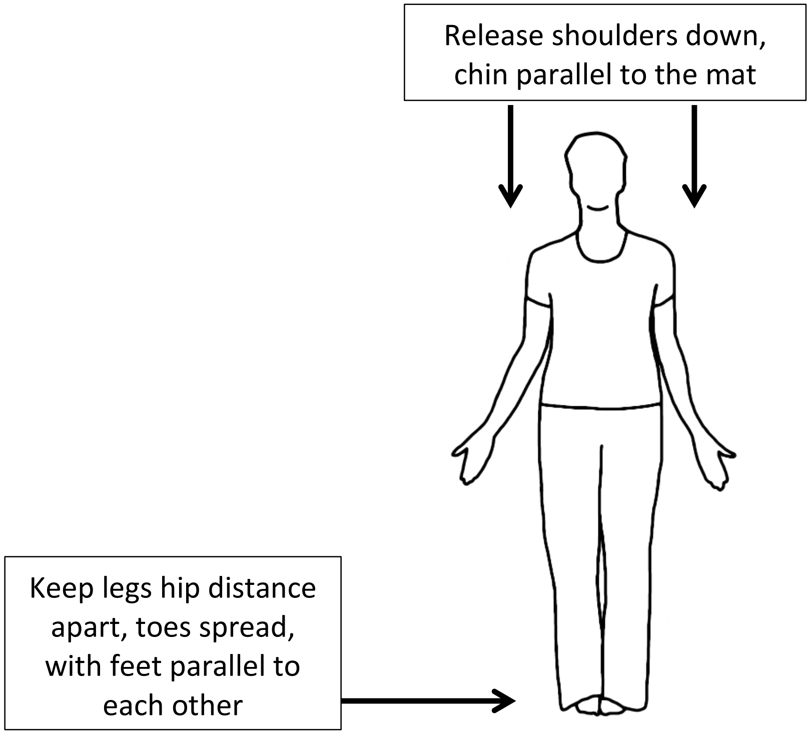

© Boston Medical Center
Veterans Back to Health Trial

Instructions for Specific Yoga Poses

Mountain Pose in Chair

Instructions

1. Sit in a chair with your back straight and your feet flat on the floor, hip-width apart and directly under your knees.
2. With your hands on your hips, move your hips slightly forward and backward to find the center.
3. Keep your back straight so that your head and shoulders are in line with your hips. Allow your back to stretch and lengthen while looking forward. Keep your head over your shoulders, your shoulders over your hips and your knees over your ankles.
4. Move your arms straight down by the side of your body.

Release shoulders down, chin parallel to the floor

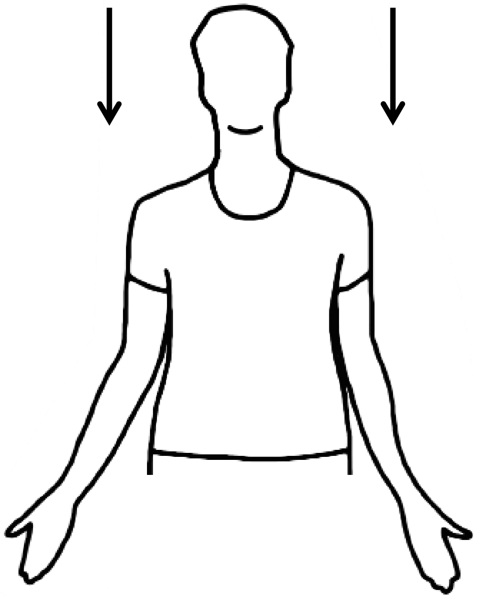

Keep feet hip-distance apart, toes spread, with feet parallel to each other

© Boston Medical Center
Veterans Back to Health Trial

Instructions for Specific Yoga Poses

Pelvic Tilt Pose

Instructions

1. Lie on your back with your knees bent and the bottoms of your feet on the mat.
2. Extend your arms down towards your feet with your palms facing down.
3. Gently press your lower back into the mat.
4. Relax your back and then repeat several times.

Other Options

- If your knees are moving out wider than hip distance, use a block between your knees to keep them closer together.
- Stand with your back and head against a wall with your feet away from the wall and slightly bent. Gently press your lower back into the wall.
- TO DO THE POSE SITTING IN A CHAIR: See Pelvic Tilt Pose in Chair (p. 51).

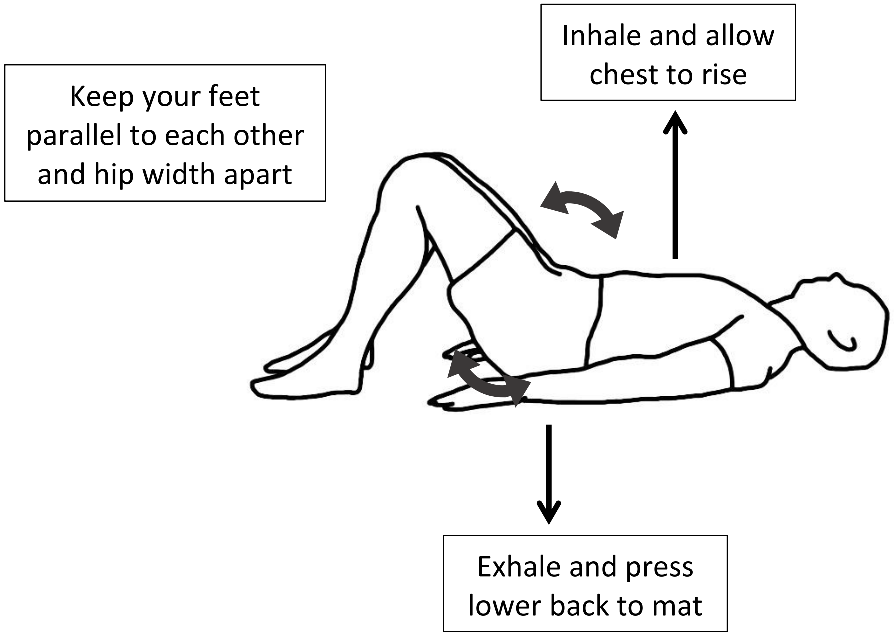

At Wall

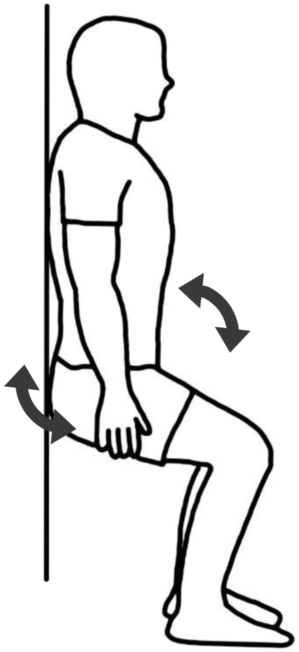

© Boston Medical Center
Veterans Back to Health Trial

Instructions for Specific Yoga Poses

Pelvic Tilt Pose in Chair

Instructions

1. Sit up straight in a chair with your feet firmly planted on the floor hip-width apart. Let your arms hang by your sides.
2. Place a block length-wise behind your back and gently press your lower back into the block. Relax your back and move back to sitting upright.
3. Repeat several times.

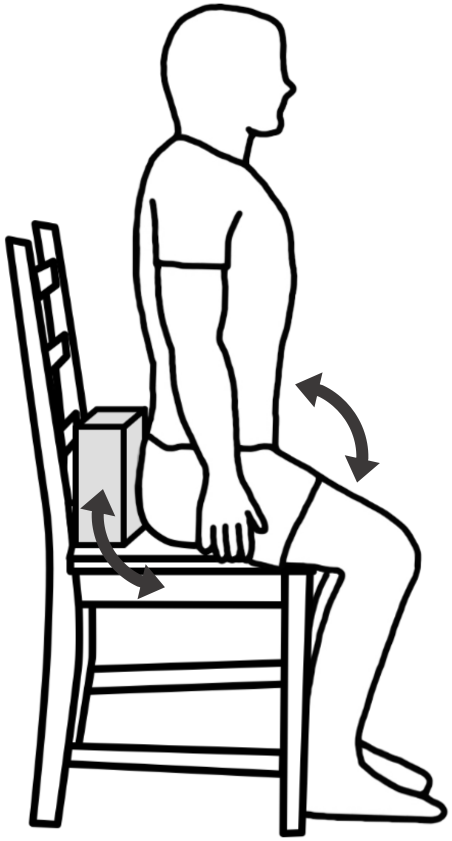

Place a block length-wise behind your back

Keep your feet parallel to each other and hip width apart

© Boston Medical Center
Veterans Back to Health Trial

Instructions for Specific Yoga Poses

Plank Pose

Instructions

1. Start on your hands and knees with your back flat and level like a table. Look down between your hands, lengthening your neck and bringing your abdominals in toward your spine.
2. Move one leg behind you, keeping your leg straight and placing your toes on the floor.
3. When you're ready, move your other leg behind you, bringing your body and head into one straight line.
4. Keep your body straight and don't let your hips sink too low. If your butt sticks up in the air, realign your body so your shoulders are directly above your wrists.
5. To release, slowly lower onto your knees, one leg at a time.

Other Options

- Place hands on the seat of a chair instead of the floor.
- You can lower your knees to the floor into Half Plank Pose. Be sure to keep your head and spine in a straight line.
- If your wrists are uncomfortable, you can roll the top edge of your mat under your hands.

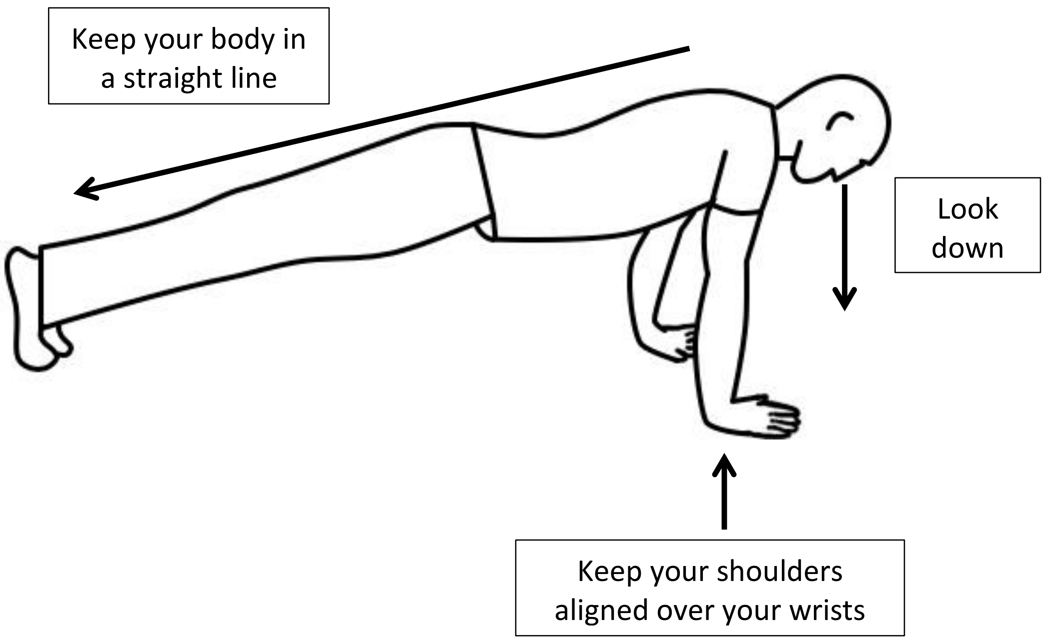

Half Plank Pose

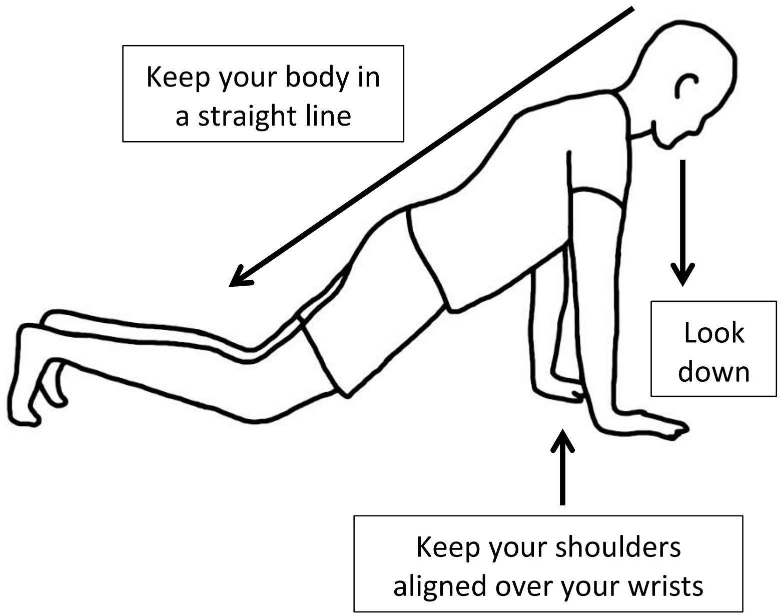

© Boston Medical Center
Veterans Back to Health Trial

Instructions for Specific Yoga Poses

Reclining Chest Opener

Instructions

1. Place a folded blanket horizontally across the mat. Lie down on your back with the blanket under your upper back.
2. If your neck is uncomfortable, place a blanket under your head.

Other Options

- For more of a chest opening, use two blankets or a block under your upper back.
- If you have lower back discomfort, bend your knees or come out of the pose.

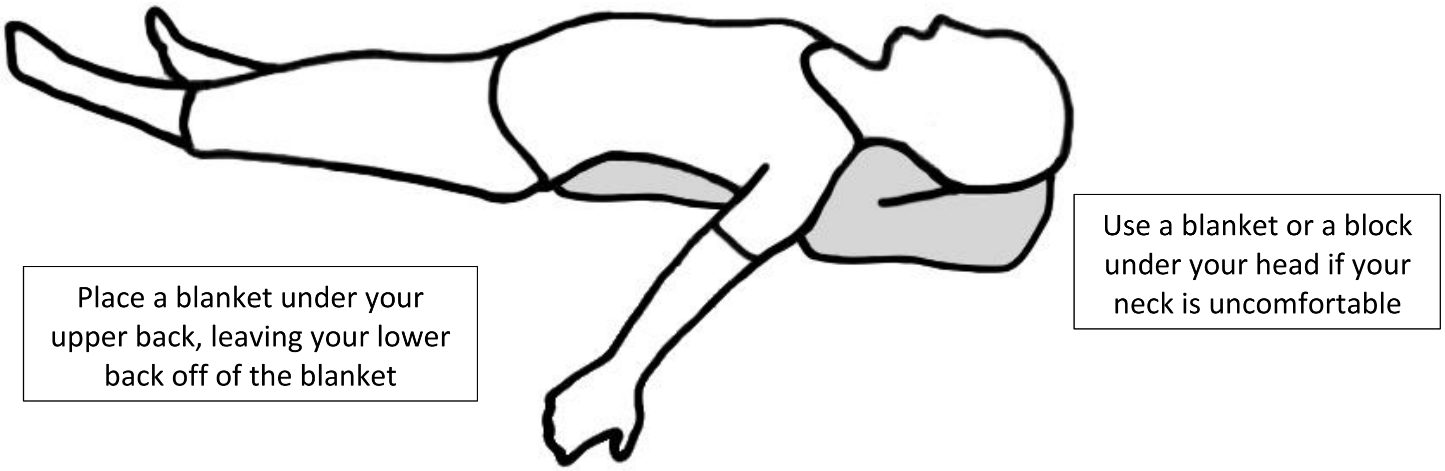

© Boston Medical Center
Veterans Back to Health Trial

Instructions for Specific Yoga Poses

Reclining Cobbler Pose

Instructions

1. Lie on your back with your knees bent. Bring the bottoms of your feet together so your knees can open to the sides.
2. Place folded blankets or bolsters on either side of your thighs. Gently lower your knees to either side with the bottoms of your feet still touching.
3. Relax your shoulders and place your arms to the sides, palms up.

Other Options

- If your lower back is uncomfortable, fold 1-2 blankets lengthwise to support length of spine from waist to head. Don't have the blankets right up to the tailbone, but rather have space from waist to tailbone.
- If your back is uncomfortable after coming out of posture, lie flat on the floor and hug both knees to the chest, gently rocking side to side.

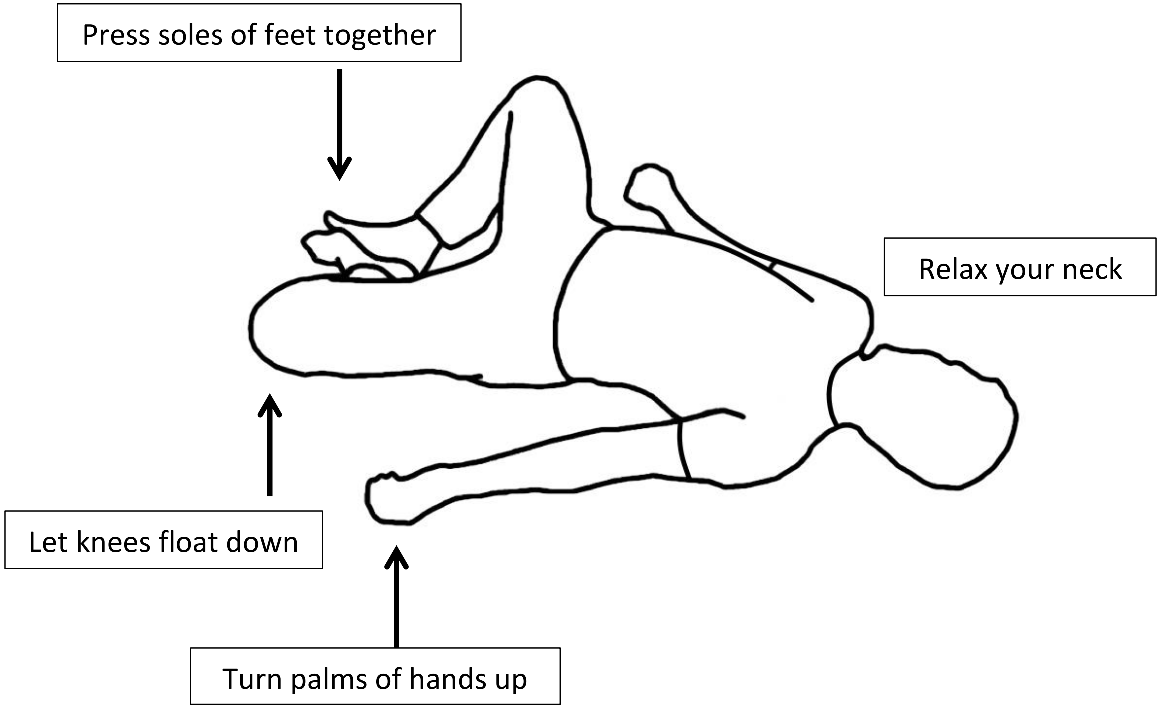

© Boston Medical Center
Veterans Back to Health Trial

Instructions for Specific Yoga Poses

Shoulder Opener Pose

Instructions

1. Start by standing with your feet hip-width apart, raise your arms straight up overhead and reach for the sky.
2. For the second stretch, place your hands behind your back and clasp your hands together. Slowly raise your arms so there is a comfortable stretch. Do not bend forward and keep your back straight. Breathe and move your shoulders back towards each other.
3. For the third shoulder stretch, place your arms in front and lace your fingers together with your palms facing away from you. Slowly lift your arms until you feel the stretch. Keep your back straight.
4. Slowly lower your arms down and release your hands.

Other Options

- If your hands cannot reach one another behind you or in front of you, use a belt between your hands.
- TO DO THE POSE SITTING IN A CHAIR: See Shoulder Opener Pose in Chair (p. 56).

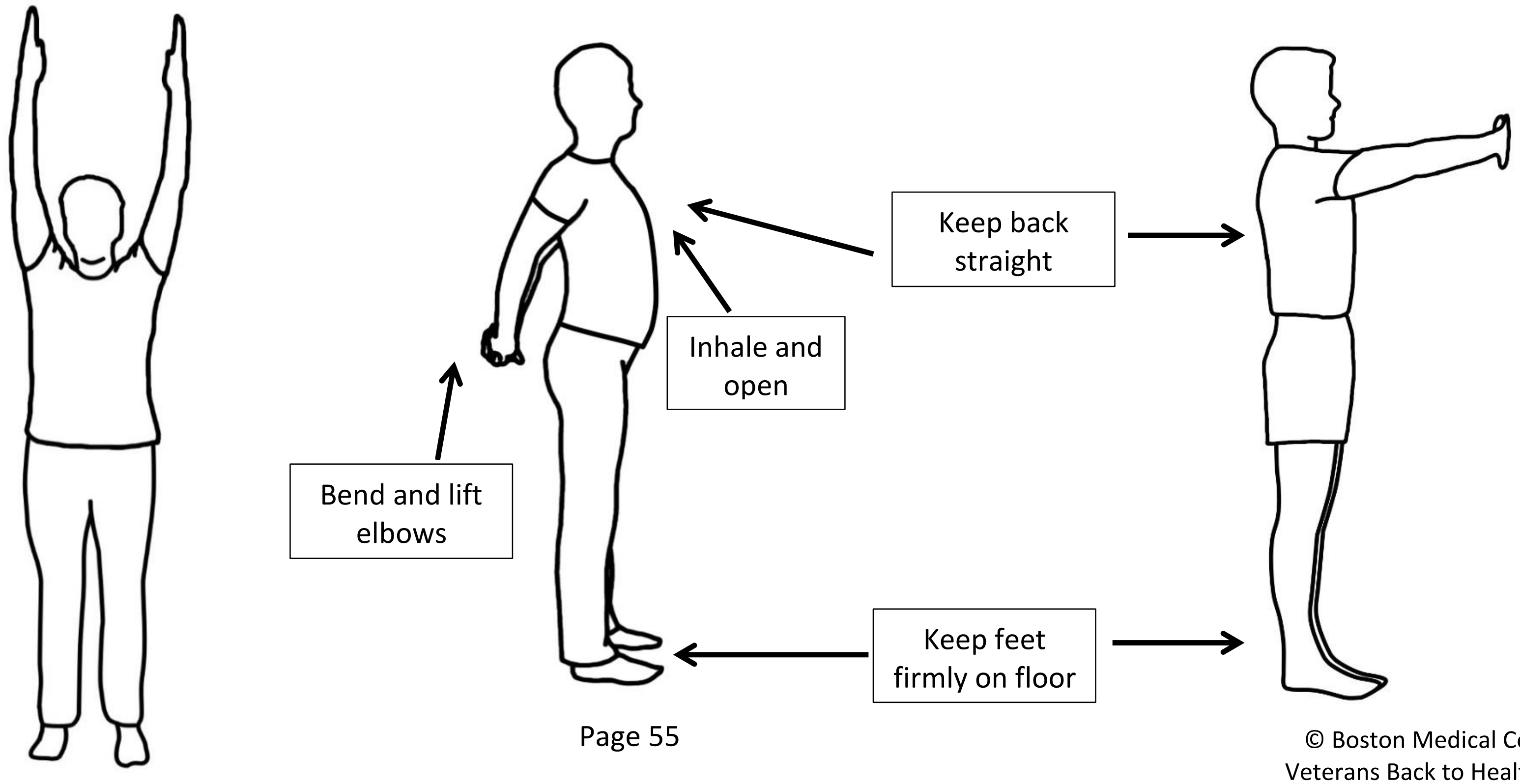

© Boston Medical Center
Veterans Back to Health Trial

Instructions for Specific Yoga Poses

Shoulder Opener Pose in Chair

Instructions

1. Sit on the edge or in the middle of a chair with your back straight and your feet firmly on the floor, hip-width apart. Keep your back straight during all these stretches.
2. For the 1st stretch, raise your arms straight up overhead and reach for the sky, lengthening your body.
3. For the 2nd stretch, place your hands behind you and clasp your hands together. Slowly raise your arms to a comfortable stretch. Do not bend forward and keep your back straight. Breathe and move your shoulders back towards each other.
4. For the 3rd stretch, place your arms in front and lace your fingers together with your palms facing away from you. Slowly lift your arms until you feel the stretch. Keep your back straight.

Other Options

- If your hands cannot reach one another behind you (2nd stretch) or in front of you (3rd stretch), use a belt.

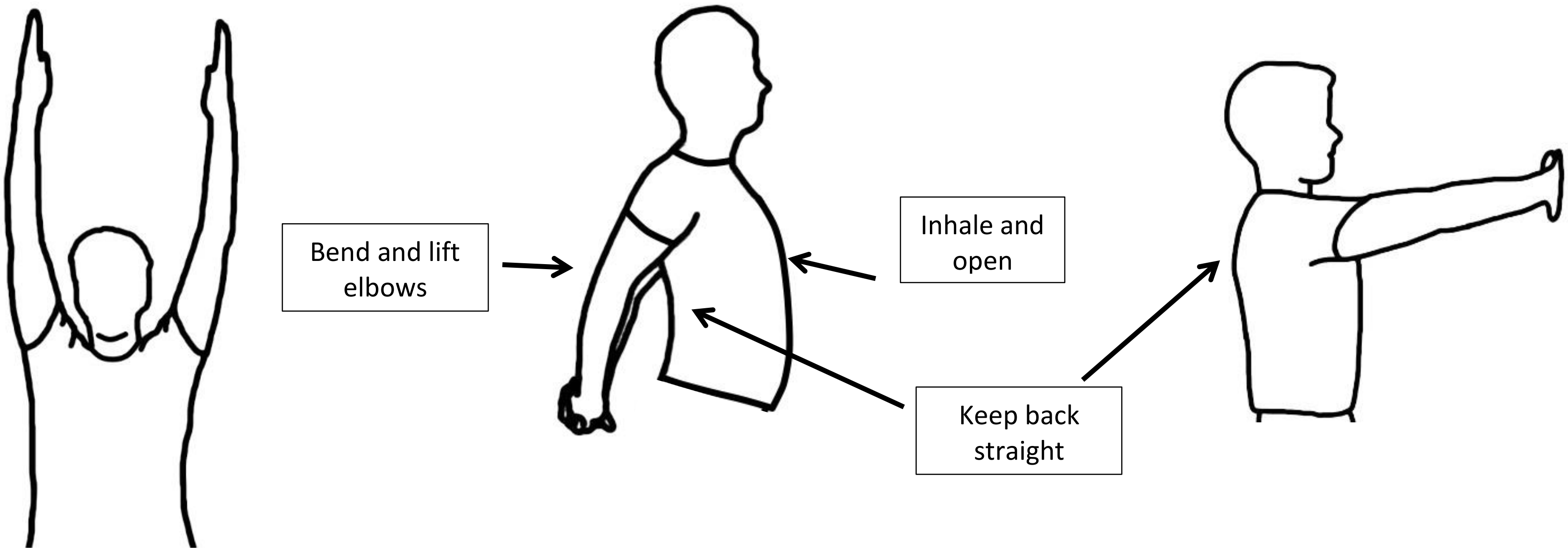

© Boston Medical Center
Veterans Back to Health Trial

Instructions for Specific Yoga Poses

Side Hip Strengtheners

<u>Instructions</u>

1. Lie on your right side with a blanket under your head to keep your neck comfortable. Make 90° angles with your legs so your knees are in line with your belly and your ankles in line with your knees, hips stacked on top of one another.
2. Extend the left leg out so the heel is in line with the crown of the head, allowing the lower leg to be relaxed. Rotate the left thigh inward, so the knee and toes point down.
3. Inhale and lift the left leg, hold; then exhale and slowly release the leg down. Begin with a single repetition and gradually increase up to three repetitions, if appropriate.
4. If at any point the weight of the leg feels too heavy, you can bend your knee.
5. Repeat laying on left side, lifting the right leg.

<u>Other Options</u>

- Instead of extending your leg before lifting, lying on the right side, isolating the uppermost portion of your outer thigh, lift the left leg on the in breath, allowing the lower leg to be relaxed. On the exhale, slowly lower the left thigh down. Start with two repetitions and gradually increase, if appropriate, to six repetitions in this position.

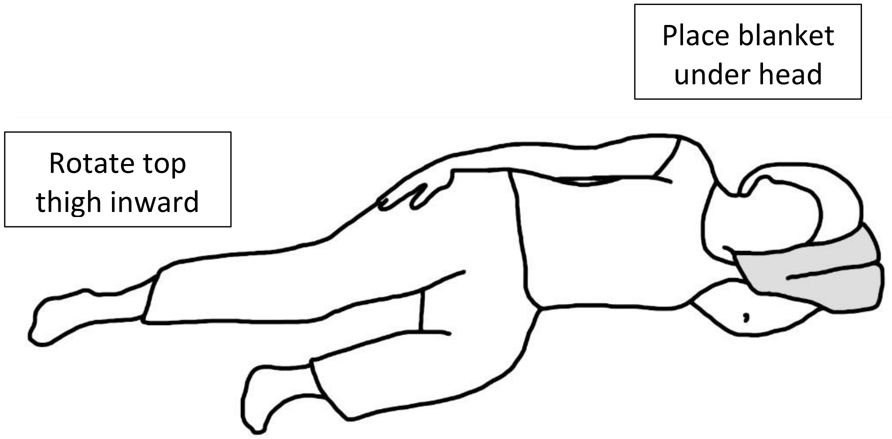

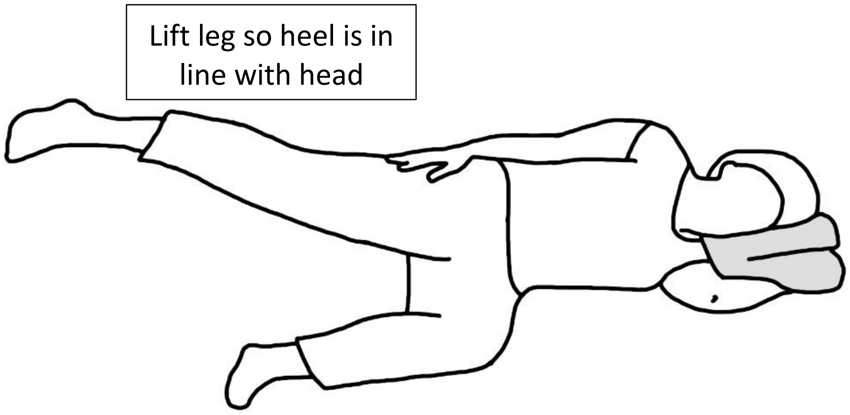

© Boston Medical Center
Veterans Back to Health Trial

Instructions for Specific Yoga Poses

Side Plank Pose

<u>Instructions</u>

1. From a plank position (p. 52), shift onto the outside edge of your left foot and stack your right foot on top of your left foot.
2. As you move your right hand onto your right hip, turn your torso to the right. Support the weight of your body on the outer edge of your left foot and your left hand. Align your body and keep it in a straight line.
3. Keep your head in a neutral position or gently turn it to gaze up at the ceiling, being careful not to overextend the neck.
4. Come back into Plank Pose and repeat on right side.

Avoid this pose if you have an arm, wrist, or shoulder injury that prevents you from holding your weight on one arm.

<u>Other Options</u>

- Keep your bottom knee and shin on the ground for support. You can also bend your arm and rest your weight on your elbow. Keep your hips lifted and your body in a straight line.
- Instead of keeping your hand on your hip, you can rest your arm on your side if it is more comfortable.
- Place hands on the seat of a chair instead of the floor. Put the chair against a wall to make sure it is secure.

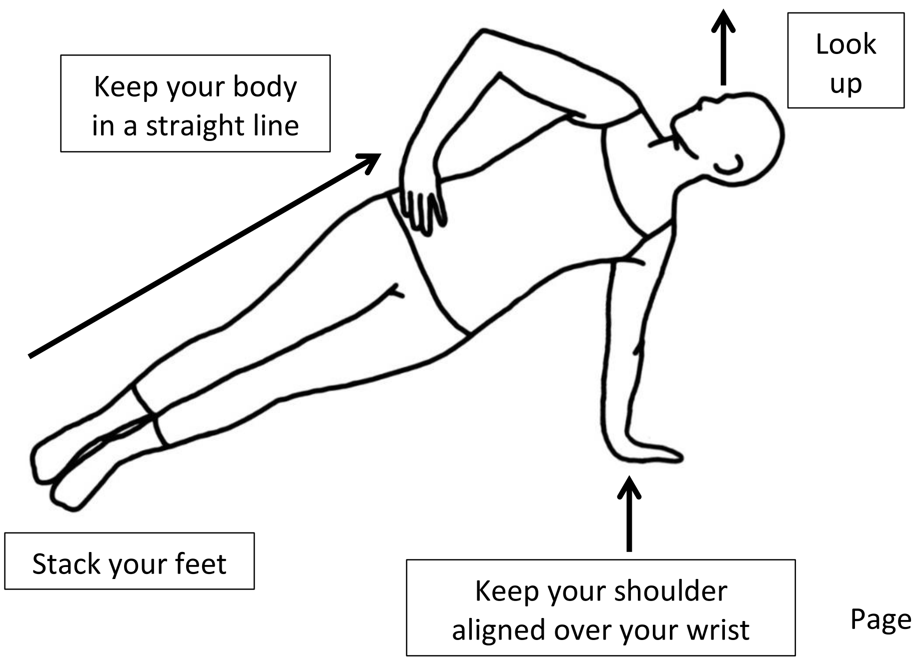

Half Side Plank Pose

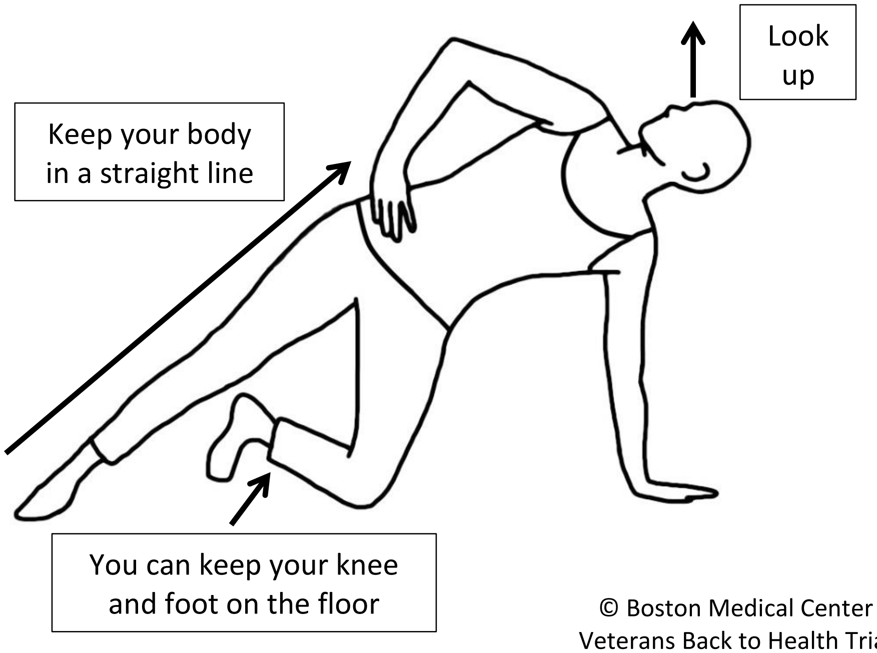

© Boston Medical Center
Veterans Back to Health Trial

Instructions for Specific Yoga Poses

Sphinx Pose

Instructions
1. Lie on your stomach with your chin or forehead on the mat and your elbows under your shoulders, close to your body.
2. Point your legs behind you with your toes on the mat.
3. Press your forearms into the mat and lift your head and chest up.

To prevent injury, make sure to look forward or slightly down. Looking up to the ceiling *can* cause neck injury if the head bends too far backwards.

Other Options
- TO DO THE POSE SITTING IN A CHAIR: See Sphinx Pose in Chair (p. 60).

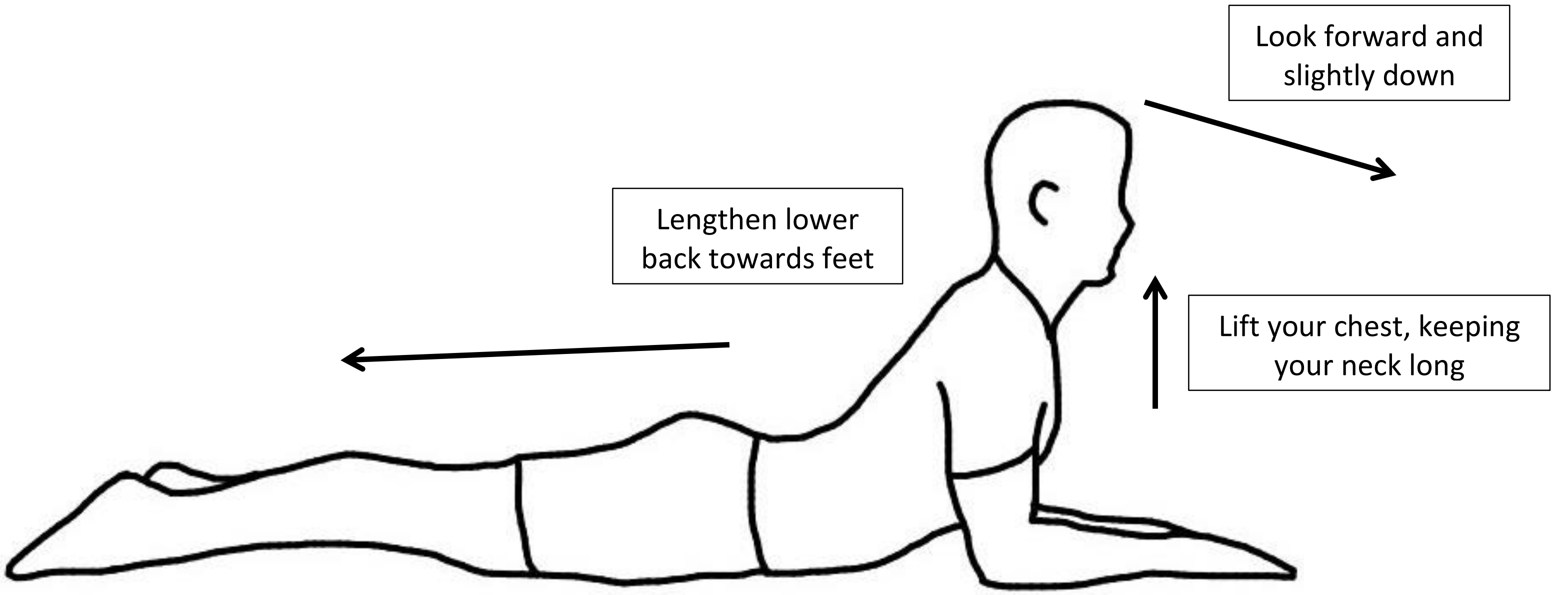

© Boston Medical Center
Veterans Back to Health Trial

Instructions for Specific Yoga Poses

Sphinx Pose in Chair

Instructions

1. Sit in a chair with your feet firmly on the floor.
2. With your hands on your thighs, allow your shoulders to drop down away from your ears. Roll your shoulders back to open your chest.
3. Move your shoulder blades down your back, away from your ears and slightly in onto your back ribs being careful not to let your abdomen push forward.
4. Pressing down gently on your thighs, lift the front of your chest up while gently pulling the shoulders down and look forward slightly upwards keeping the back of the neck long.

To prevent injury, make sure to look forward. Looking up to the ceiling *can* cause neck injury if the head bends too far backwards.

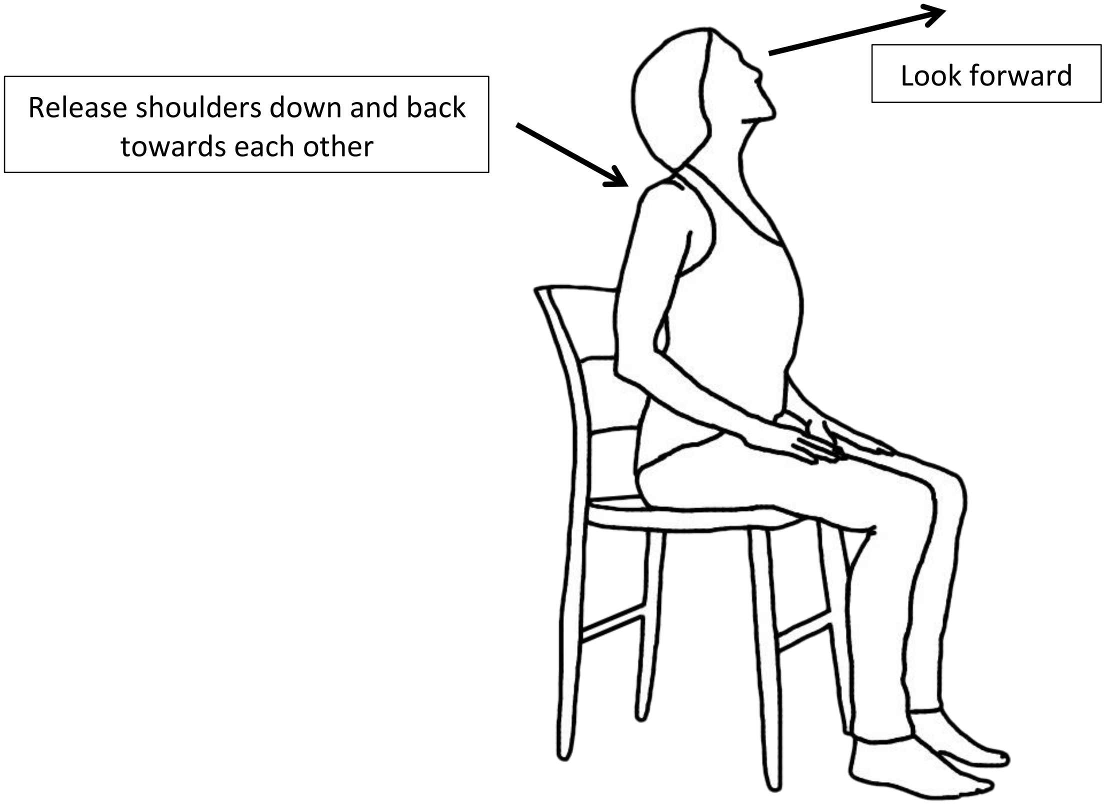

© Boston Medical Center
Veterans Back to Health Trial

Instructions for Specific Yoga Poses

Table Top with Leg Extended Pose

Instructions
1. Start on your hands and knees with your back flat and level like a table. Move your right leg behind you with your leg straight and your toes on the floor.
2. Bring your leg back so your knee is on the mat.
3. Repeat on the other side, stretching your left leg behind you.

To prevent injury, make sure to look forward or slightly down. Looking up to the ceiling *can* cause neck injury if the head bends too far backwards.

Other Options
- If your wrist is uncomfortable, make a fist with both hands resting your weight on your knuckles with thumbs facing down.
- If your knees or hands are uncomfortable, you can roll the sides of the mat under your knees or the top of the mat under your hands.
- Rest your forearms on the mat instead of your hands.
- Standing with hands on the back of a chair (bending forward) or against the wall, extend one leg at a time back with your toes down on the floor.
- TO DO THE POSE SITTING IN A CHAIR: See instructions for Forward Bend Pose in Chair (p. 42).

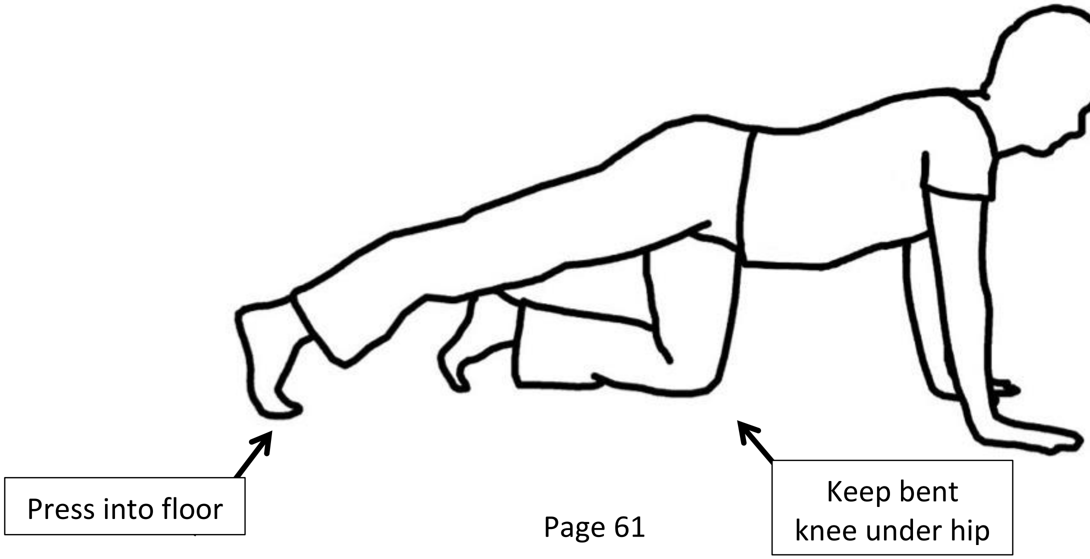

© Boston Medical Center
Veterans Back to Health Trial

Instructions for Specific Yoga Poses

Triangle Pose

Instructions
1. Start by standing with your feet hip-width apart with the left side of your body facing the wall and your left hand on the wall.
2. Turn your left leg towards the wall with your toes close to or touching the wall.
3. Turn your right foot slightly in towards the wall.
4. Bend at your left hip towards the wall and slide your left hand up the wall.
5. Turn around and repeat on the other side (right side facing the wall).

Other Options
- Place right hand on a chair seat when bending to the right, and vice versa on left.
- When you're ready to try this pose without using a wall or chair for balance, stand with your feet hip-width apart and spread your arms out to the sides at shoulder height. Turn your right foot to the right and your left foot slightly to the right. Bend at the right hip and place your right hand on your right leg and you move your left arm straight up. Keep your legs, arms, and back straight. Inhale and bring your body and arms back to center. Repeat on the other side. See picture below on right side.
- TO DO THE POSE SITTING IN A CHAIR: See Triangle Pose in Chair (p. 63).

Triangle Pose at Wall

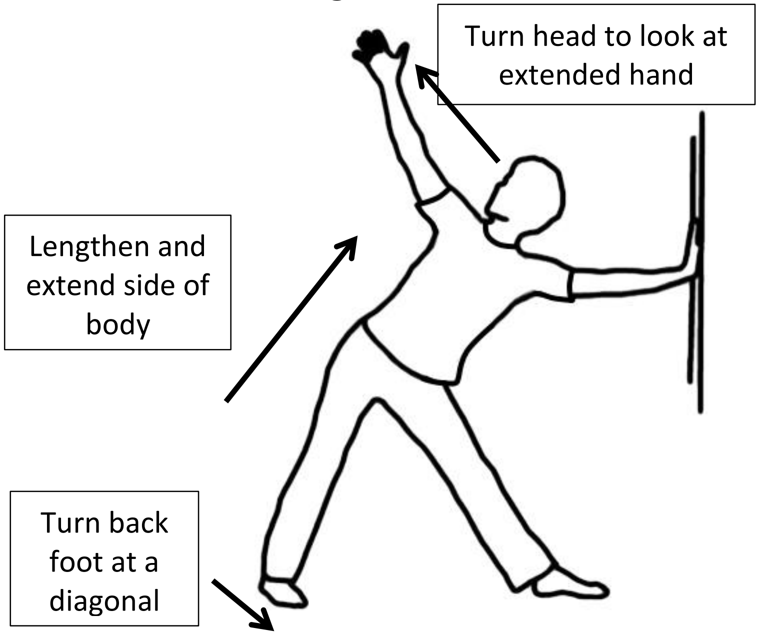

Triangle Pose

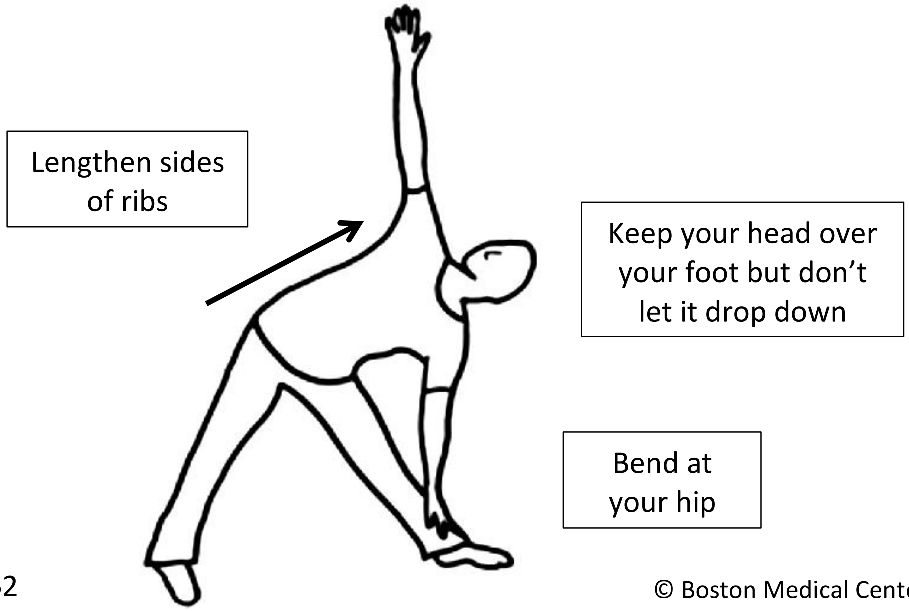

© Boston Medical Center
Veterans Back to Health Trial

Instructions for Specific Yoga Poses

Triangle Pose in Chair

<u>Instructions</u>

1. Sitting in a chair, extend your right leg out to the side, turning the whole leg out 90° with the toes pointing to the side wall. Your left leg should be bent with the foot on the floor, toes pointing forward.
2. Stretch your arms out to your sides at shoulder height.
3. Bend at the hips to the right side, bending directly over your extended leg and place the right hand on the leg for support.
4. Inhale and bring your arms and body back to center. Repeat on the other side, extending the left leg out to the side.

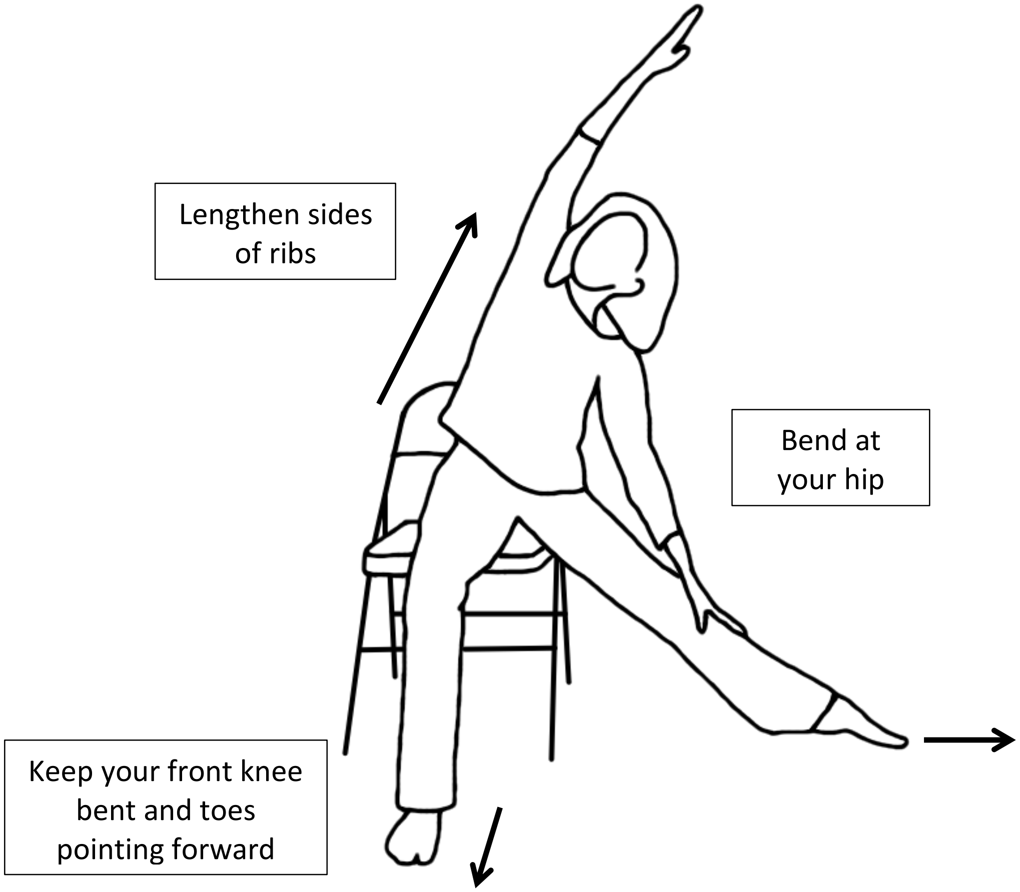

© Boston Medical Center
Veterans Back to Health Trial

Instructions for Specific Yoga Poses

Warrior I Pose

<u>Instructions</u>

1. Start by standing with your feet hip-width apart and step your right foot back a few feet. Turn your right foot out at a diagonal and adjust your stance to find a good balance. You might want to move your left foot to the left a bit.
2. Place your hands on your hips to guide your hips towards the front.
3. Bend into your left leg so your knee is on top of your ankle. Keep your right leg straight. Press into your right heel for balance.
4. Raise your arms straight up over your head next to your ears, reaching for the ceiling. Drop your shoulders down.
5. Bring your arms down and step forward to a neutral standing position with feet hip-width apart. Repeat on the other side, moving your left foot back and bending your right knee.

<u>Other Options</u>

- Bend your elbows so your arms are like goalposts or keep your hands on your hips, still lifting your belly with a straight back.
- Move your back foot slightly to the side, away from the center of the body to make a wider stance if you feel unsteady.
- Warrior at Wall Pose (p. 70) or place hands on a chair in front of you to steady yourself.
- <u>TO DO THE POSE SITTING IN A CHAIR</u>: See Warrior I Pose in Chair (p. 65).

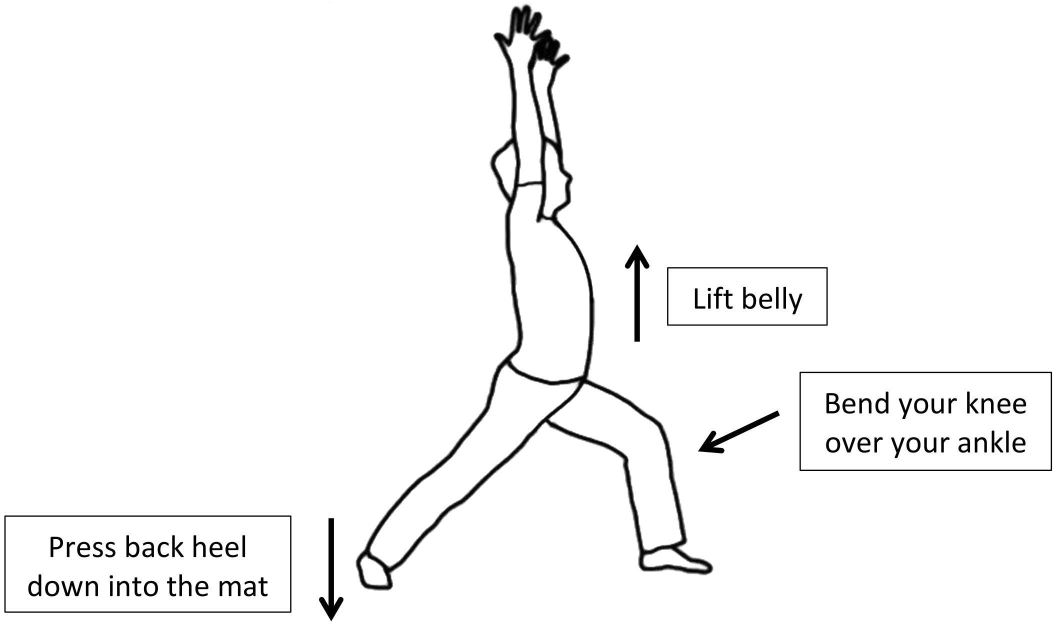

© Boston Medical Center
Veterans Back to Health Trial

Instructions for Specific Yoga Poses

Warrior I Pose in Chair

Instructions

1. Using a chair without arms, sit sideways on the chair with your right leg bent so that your knee is over your ankle and your left leg is extended behind you with your foot turned out at an angle. Keep your left heel off the floor to relieve pressure in your left knee if needed.
2. Facing sideways on the chair, raise your arms up overhead and lift chest.
3. If lifting the arms up is uncomfortable in your back or your shoulders, bend your elbows to make your arms like goalposts or keep your hands on your hips.
4. Repeat on the other side with your left leg bent and your right leg extended behind you with your right foot turned out at an angle. Keep your right heel off the floor to relieve pressure in your right knee if needed.

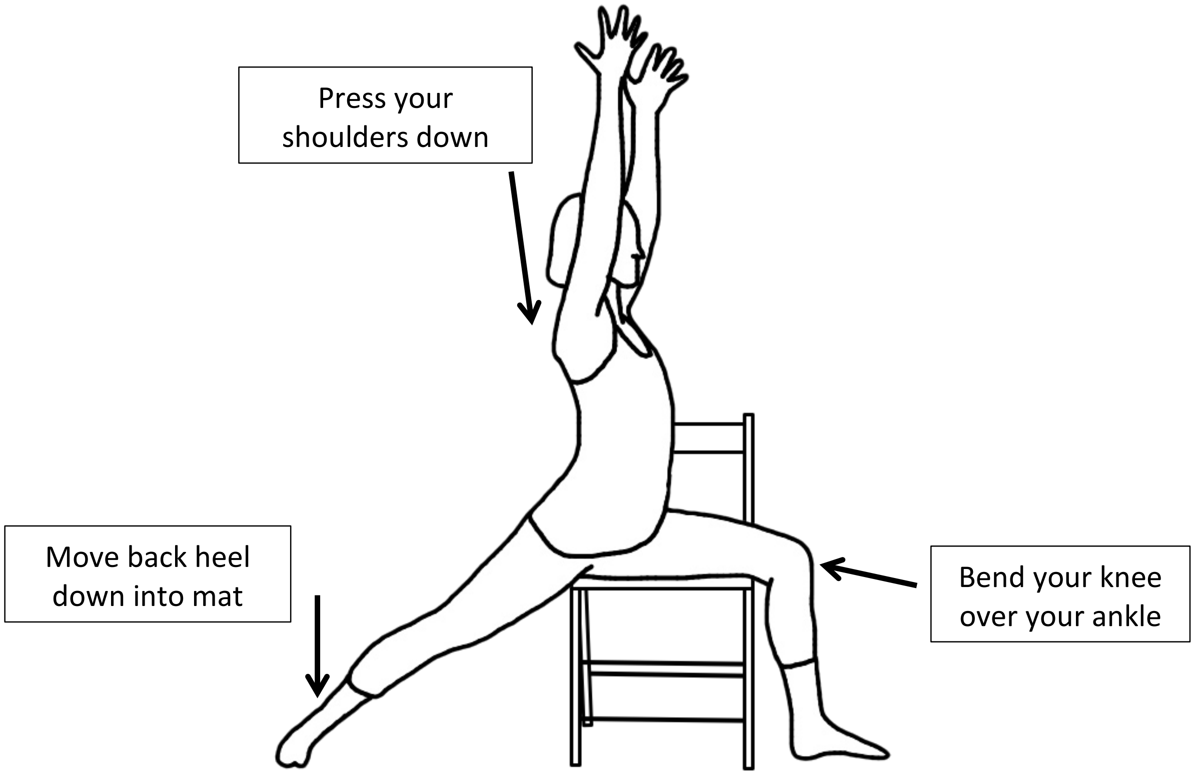

© Boston Medical Center
Veterans Back to Health Trial

Instructions for Specific Yoga Poses

Warrior II Pose

Instructions

1. Start by standing with your feet hip-width apart and step your right foot out wider than hip-width apart.
2. Stretch your arms out to the sides at shoulder height with your shoulder blades wide and your palms facing down.
3. Turn your right foot slightly inward, keeping the right leg straight, and turn your left foot out so it points to the left side. Keep your left heel in line with your right heel.
4. Turn your left thigh outward and, on an exhale, bend your left leg so your knee is on top of your left ankle.
5. Keep your right leg straight. Press your right heel firmly to the floor. Hold the pose for 30 seconds to 1 minute.
6. To come up, inhale, bring your arms down and step back to a standing position. Repeat on the other side, bending your right knee.

Other Options

- Warrior at Wall Pose (p. 68)
- Keep your hands on your hips if your shoulders are unsteady.
- TO DO THE POSE SITTING IN A CHAIR: See Warrior II Pose in Chair (p. 67)

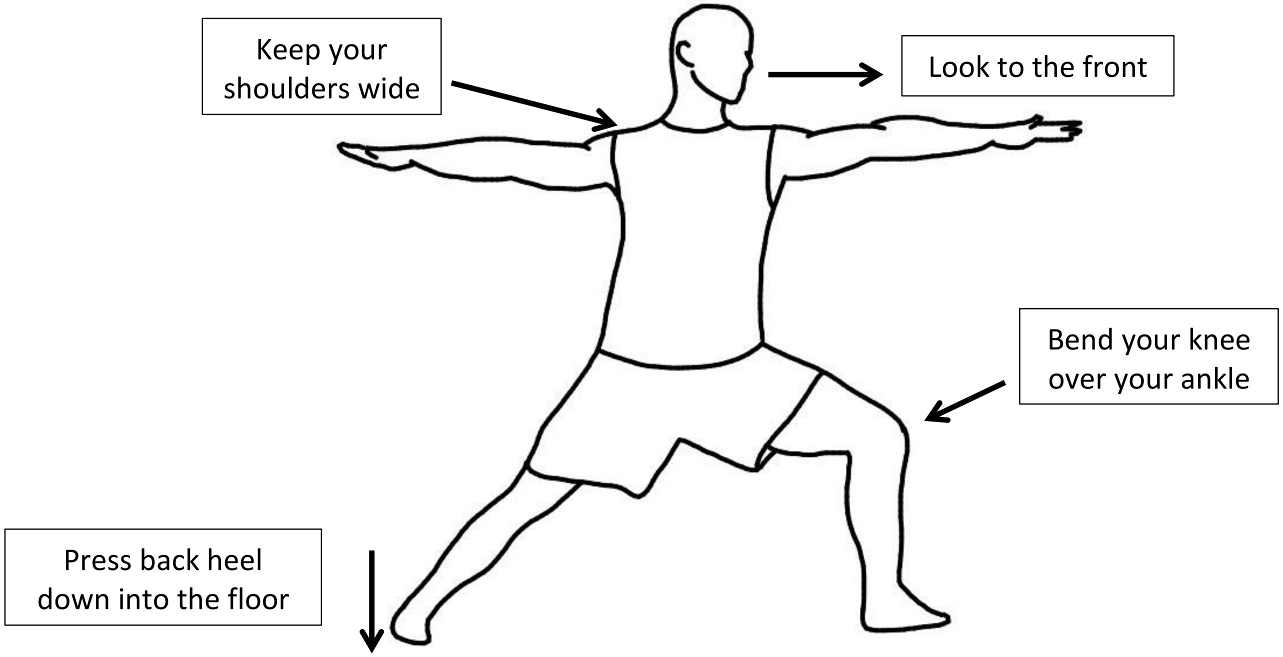

© Boston Medical Center
Veterans Back to Health Trial

Instructions for Specific Yoga Poses

Warrior II in Chair Pose

Instructions

1. With the right knee bent into a right angle, sit the right thigh on the chair seat with the left leg stretching back in the opposite direction. The torso is facing the front, not sideways.
2. Keeping the spine vertical with the shoulders aligned over the hips, stretch both arms out away from each other at shoulder height. Look to the right.
3. If lifting the arms is uncomfortable in your back or your shoulders, bend your elbows to make your arms like goalposts or keep your hands on your hips.
4. Repeat on the other side with your left thigh on the chair and your left knee bent. Extend your right leg to the side.

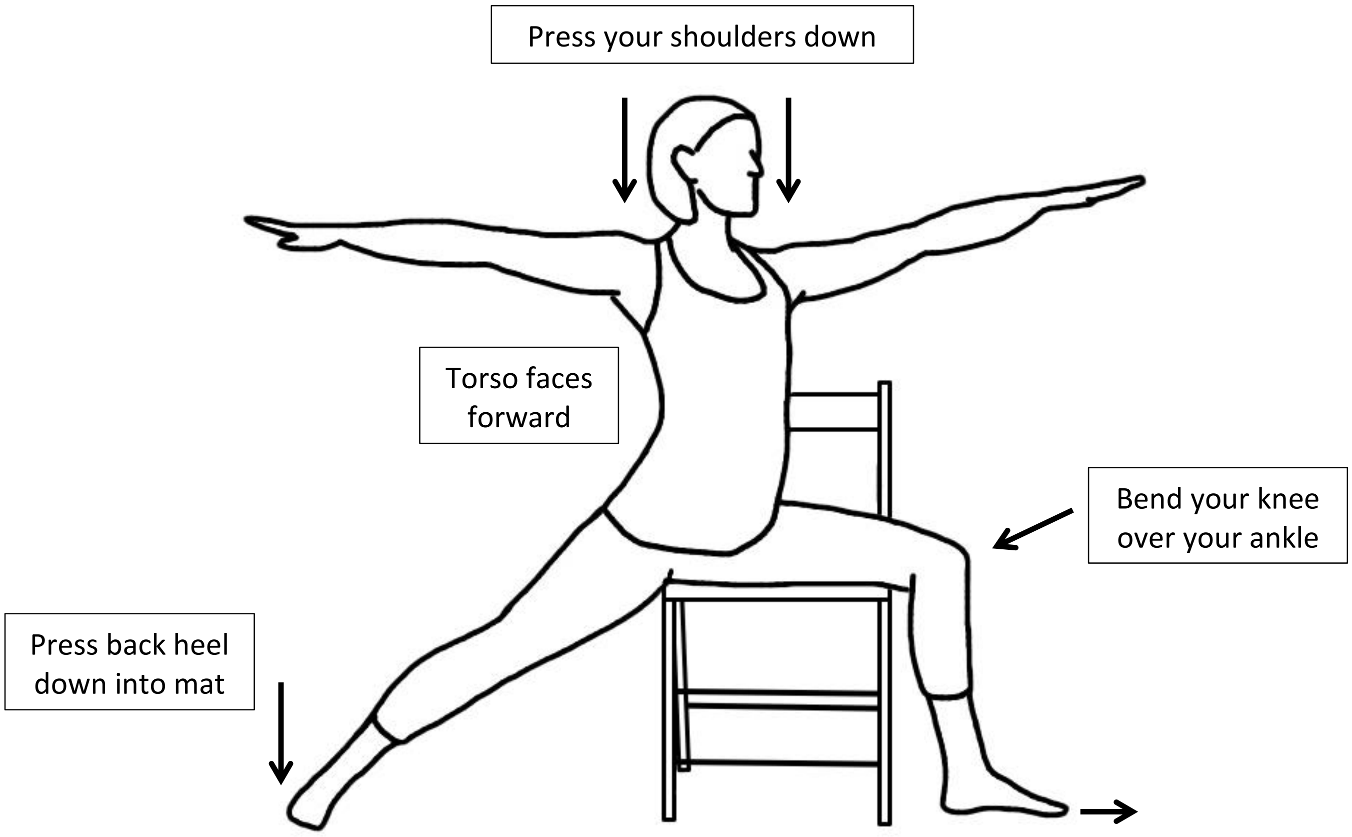

© Boston Medical Center
Veterans Back to Health Trial

Instructions for Specific Yoga Poses

Warrior at Wall Pose

<u>Instructions</u>

1. Start facing the wall with your feet hip-width apart and your hands on the wall at shoulder height, arms straight. Step your right foot up to the wall and your left leg back as far as possible, bend your right knee over your ankle.
2. Turn your left foot out slightly so there is a stretch in the back of your leg.
3. Straighten and move your right leg back. Repeat on the other side.

<u>Other Options</u>

- Allow the back of the heel to come off floor.
- Place a block between knee and wall pushing knee into block.
- Lean into wall, placing head on wall.
- <u>TO DO THE POSE SITTING IN A CHAIR</u>: See Warrior I Pose in Chair (p. 65).

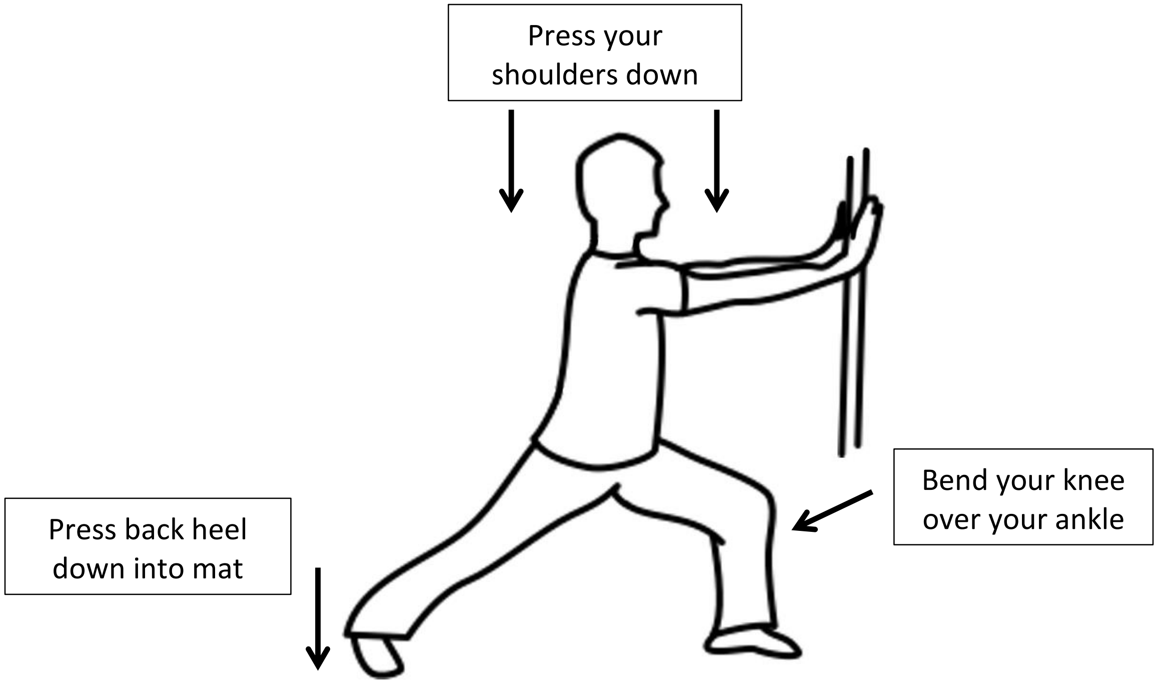

© Boston Medical Center
Veterans Back to Health Trial

Instructions for Specific Yoga Poses

Wheel Pose

Instructions

1. Start on your hands and knees, with your hands shoulder-width apart and your knees hip-width apart.
2. As you inhale, lift your tailbone and your chest up, slightly arching your back while keeping your neck long and your chin slightly down.
3. On an exhale, gently drop your head and tuck your tailbone under as you stretch all the way back to your heels.
4. Repeat this in a flowing motion, inhaling to lift your chest and tailbone, and exhaling to move towards your heels.

To prevent injury, make sure to look forward or slightly down. Looking up to the ceiling *can* cause a neck injury if your head bends too far backwards.

Other Options

- If your knees or hands are uncomfortable, you can roll the mat under your knees or under your hands for padding.
- If your feet cramp, you can keep your toes curled under your feet.
- <u>TO DO THE POSE SITTING IN A CHAIR</u>: See Wheel Pose in Chair (p. 70).

Inhale

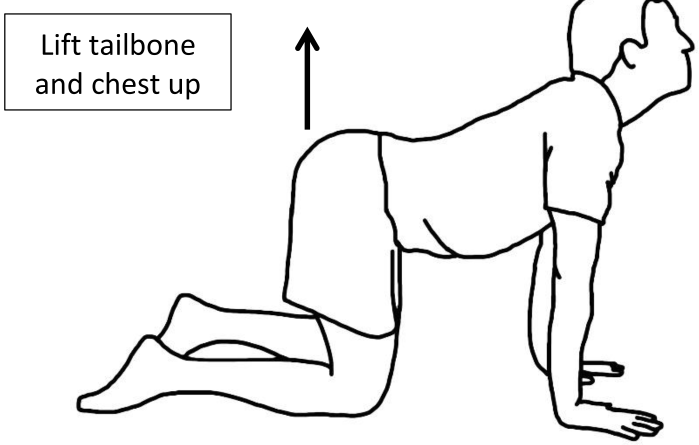

Exhale

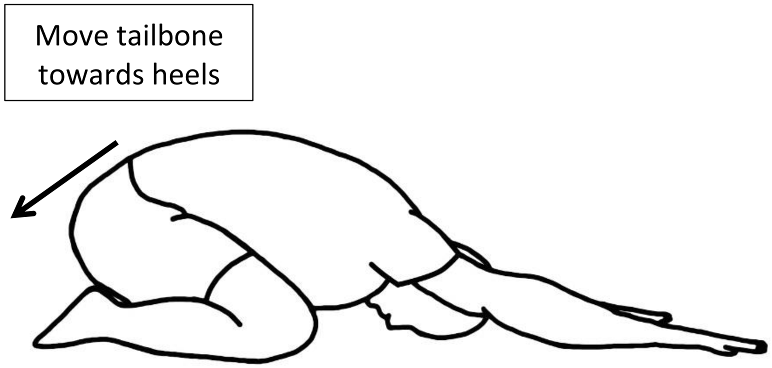

© Boston Medical Center
Veterans Back to Health Trial

Instructions for Specific Yoga Poses

Wheel Pose in Chair

<u>Instructions</u>

1. Sit at the edge of a chair with your thighs parallel to the ground, knees hip-width apart, and your feet under your knees. Your hands can rest palms down on your thighs or your knees.
2. On an inhale, gently lift your head and chest up, slightly arching your back.
3. On an exhale, slowly bend forward, so that your head moves toward the floor and your hands move down your legs toward your calves. Or you can keep your hands on your thighs/knees if you prefer,
4. Repeat this in a flowing motion, inhaling to open your chest, and exhaling to bend forward.

To prevent injury, make sure to look forward. Looking up to the ceiling *can* cause a neck injury if your head bends too far backwards.

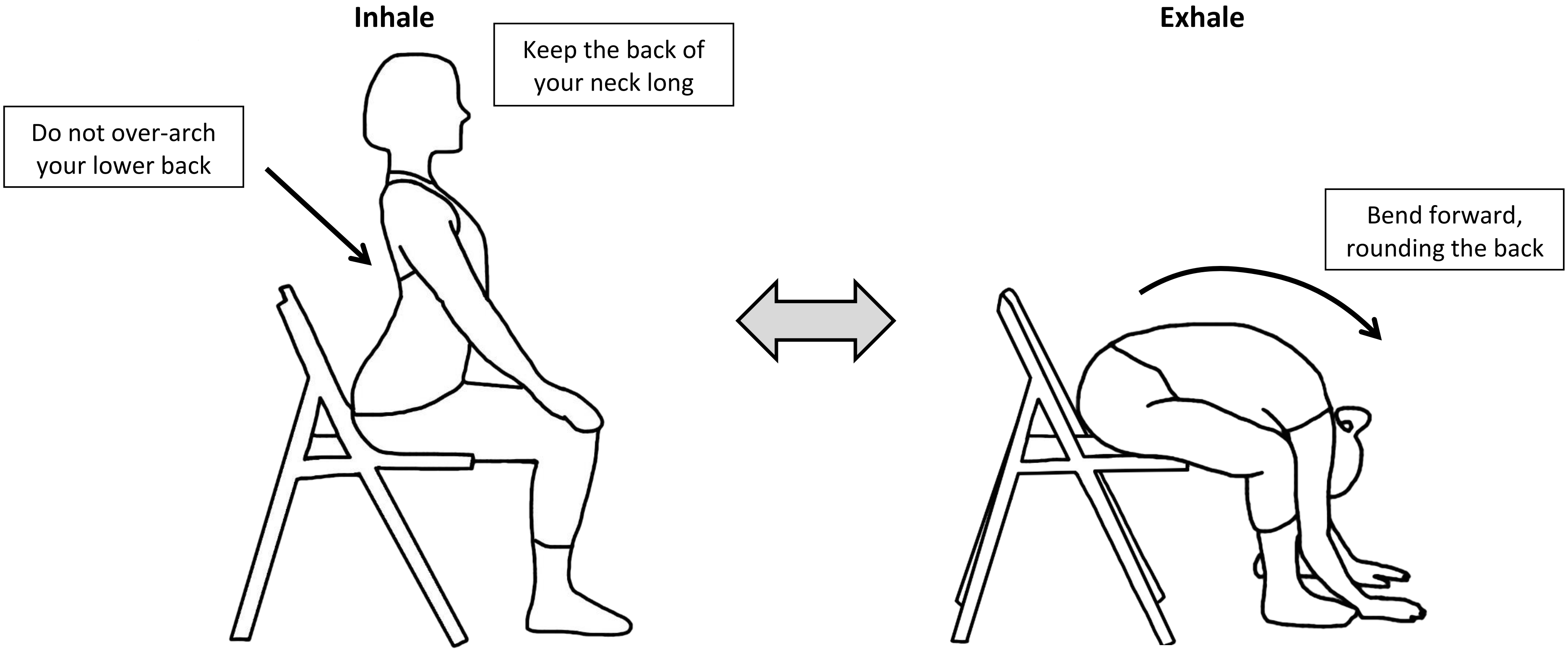

© Boston Medical Center
Veterans Back to Health Trial

Instructions for Specific Yoga Poses

Wide-Leg Forward Bend Pose

<u>Instructions</u>

1. Start by standing with your feet hip-width apart facing a wall and move your feet into a wide stance, about 4 feet apart.
2. Inhale and lift your chest, making the front torso slightly longer than the back.
3. With your hands on the hips, bend forward from your hips, keeping your back straight with the back of your head staying in line with your tailbone.
4. Place your hands on the wall, a chair, or blocks. Keep the weight balanced evenly in your feet and keep your legs straight.
5. Stay in the pose anywhere from 30 seconds to 1 minute.
6. To come out, lift and lengthen your front torso, keeping a flat back, into Mountain Pose.

<u>Other Options</u>

- Bend your knees a little to make the forward bend easier.

Wide-Leg Forward Bend at Wall

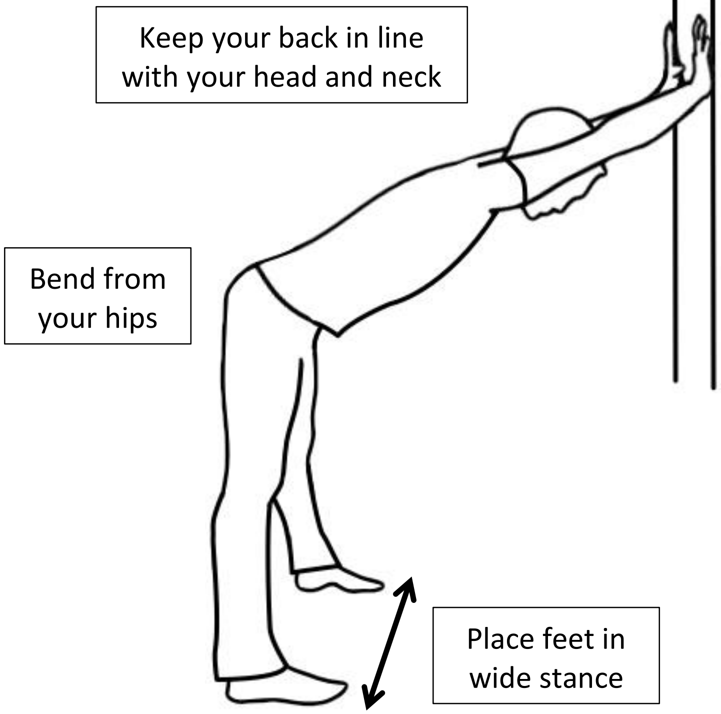

Wide-Leg Forward Bend with Blocks

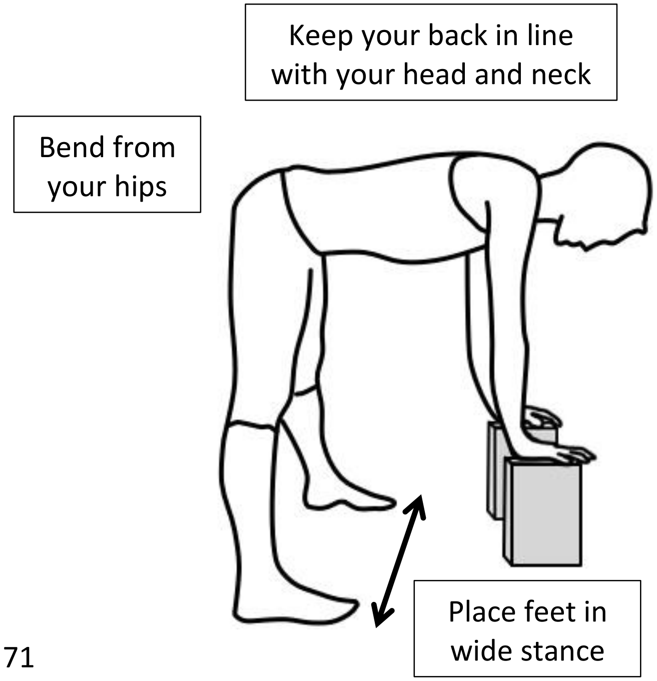

© Boston Medical Center
Veterans Back to Health Trial
