# Supplementary material for: Yoga versus education for Veterans with chronic low back pain: study protocol for a randomized controlled trial
Source: Trials. 2016 Apr 29;17:224. doi: 10.1186/s13063-016-1321-5 (PMC4850721; doi:10.1186/s13063-016-1321-5)
Supplement: Additional file 6: — Yoga Teacher Manual. A training and reference manual given to study yoga instructors that includes detailed information on the segment themes, weekby- week yoga class structure, teaching individual postures with or without modifications, teaching breathing exercises, and leading Veterans through integrated relaxation. (PDF 3.52 mb) [file 13063_2016_1321_MOESM6_ESM.pdf]

A Study Comparing Yoga and Education for Veterans with Chronic Low Back Pain

Veterans Back to Health

Yoga Teacher Manual

© Boston Medical Center
Veterans Back to Health Trial

Table of Contents

Acknowledgements 1
Introduction from Principal Investigator 2
Staff Contact Information 4
Veterans Back to Health Study Design 5
Yoga Teacher Training 6
Overview of Home Practice 7
Yoga Teacher Sickness and Absence Policy 8
Yoga for Veterans with Chronic Low Back Pain 9
    Anatomy of Non-Specific Low Back Pain for Yoga Teachers 9
    Chronic Low Back Pain in Military Veterans 9
    Psychological Comorbidities and the Polytrauma Triad 10
    Treatment for Chronic Low Back Pain 11
    Yoga for Low Back Pain 12
    Studies of Yoga for Veterans with Low Back Pain 13
Teaching Yoga to Veterans 14
    Teaching Yoga Poses 14
    Guiding Breathing and Relaxation Exercises 16
    After Class Reflections 16
Yoga Teacher Roles and Responsibilities 18
Veterans Back to Health Yoga Protocol 19
    Protocol Development 19
    Adherence to Protocol 19
The Four Segments 20
Yoga Class Structure 21
The 12 Principles of Yoga Philosophy 23
Segment One: Opening to Something New 24
Segment Two: Listening to Your Back 27
Segment Three: Engaging Your Power 30
Segment Four: Bringing it Home 33
Guidelines for Teaching Specific Yoga Poses 36
Guidelines for Teaching Yoga Breathing Exercises 69
Guidelines for Teaching Yoga Relaxation Exercises 74
Poems and Readings 78
Bibliography 90

© Boston Medical Center

Appendices.. 95
Supplemental Poems and Readings.. 96
Yoga Teacher Notes Form.. 104
Yoga Home Practice Log 105

Acknowledgements

We would like to express our deepest gratitude to the many individuals and groups who contributed to writing, reviewing, and testing this yoga study protocol and manual.

Members of a panel of yoga, back pain, and military experts convened at Samueli Institute (Alexandria, VA) in November 2014: LTG Eric Schoomaker (Ret.), MD PhD, Karen Sherman, PhD MPH, Erik Groessl, PhD, Susan Lynch, JD E-RYT, COL Patricia Lillis (Ret.), MD MHA E-RYT, Robin Carnes, MBA E-RYT, Andrea Lucie, MA, and Audrey Schoomaker, RN BSN E-RYT.

There & Back Again (thereandbackagain.org), founded by Susan Lynch, and Warriors at Ease (warriorsatease.org), co-founded by Pat Lillis and Robin Carnes, were both instrumental in the adaptation of this manual for Veterans as well as developing in-person and web-based materials for yoga instructor training.

We would like to recognize Carol Faulkner, CIYT, for her vital work on previous versions of this manual and the current manual.

We would also like to thank research staff and other yoga experts who worked on earlier versions of this manual, including Chelsey Lemaster, MS MPH, Shayna Egan, MPH, Margo Godersky, Martina Tam, Julia Keosaian, MPH, Christian Cerrada, Danielle Dresner, MPH, Anna Dunwell, MFA E-RYT, and Maya Breuer, E-RYT.

This publication was made possible by grant number 3R01AT005956-05S1 from the National Center for Complementary and Integrative Health (NCCIH) at the National Institutes of Health. Its contents are solely the responsibility of the authors and do not necessarily represent the official views of NCCIH.

© Boston Medical Center
Veterans Back to Health Trial

Introduction from Principal Investigator

Welcome to the *Veterans Back to Health* study! *Veterans Back to Health* is a comparative effectiveness randomized controlled trial (RCT) funded by the National Institutes of Health (NIH) and National Center for Complementary and Integrative Health (NCCIH). The study compares two interventions – yoga and education – for the treatment of chronic low back pain (cLBP) in a U.S. Veteran population. CLBP is a widespread issue that affects up to 10% of U.S. adults and results in over $50 billion per year in direct health care costs. CLBP is especially prevalent in Veterans; it is the most common pain condition in the military, and causes substantial physical and psychological suffering, reduction in force readiness, and high economic cost to the military. Several recent studies suggest yoga is an effective treatment for the physical pain caused by cLBP, but yoga may have additional psychological and economic benefits. These benefits include improved mood, stress reduction, and lower health care costs. Education on self-care for cLBP has also been studied and has been found to improve patient outcomes. Education can come in various forms, such as provider counseling, written materials, and/or support. While both yoga and education have been shown to be effective in treating cLBP in civilians, no study has compared the effects of yoga and education in a Veteran population. Identifying new safe and effective alternatives to pain medications that address the physical and psychosocial dimensions of cLBP in the highly affected Veteran population is urgently needed. This study was designed to address this important gap in research. *Veterans Back to Health* will compare two treatment groups:

1. A standardized 12-week yoga protocol delivered in a class format and supplemented by home practice
2. An education program that includes a comprehensive book on evidence-based self-care approaches for management of LBP supplemented by newsletters

The major outcomes of interest in the study are back pain intensity and back-related function. Medication use, quality of life, satisfaction, psychological parameters, and cost are some of the other important outcomes that will be measured.

We have designed a hatha yoga protocol specifically for this study. An earlier version of the yoga protocol was developed in 2006-07 by an expert panel after a systematic review of lay and scientific literature on yoga and low back pain. Panel members had experience in several styles of hatha yoga. This protocol was used in a 2007 pilot study of yoga versus usual care for 30 patients with cLBP. It was further refined through a 2012 yoga dosing study, where 95 participants were randomized to either once-weekly or twice-weekly yoga classes. This protocol was then adapted for use in a large study for 320 participants that compared yoga, physical therapy, and education. In order to further adapt the yoga protocol to a Veteran population, a panel of individuals with expertise in working with Veterans met to discuss additional modifications. Learning how to teach the protocol and adhering to the protocols described herein are essential for the study to be valid and the results generalizable.

In order to give your best effort to the study, it is critical to take care of yourself by taking the time to eat properly, get enough sleep and taking personal time. Make sure you have time before class to prepare yourself so you are ready to give each class your best. Reading this manual thoroughly and making sure you know the lessons, postures and modifications will help you to guide Veterans safely through the study. For interested instructors, this manual provides

© Boston Medical Center
Veterans Back to Health Trial

a comprehensive background on cLBP in military Veterans, psychological comorbidities, treatment of cLBP, and yoga for cLBP (see pp. 9-13). The Veterans in this study may have complicated physical and psychological medical histories, so please keep their needs in mind at all times during the classes. Give your support to each Veteran to guide them through beginning their own yoga practice and continuing on with home practice beyond the duration of the study.

As a member of the yoga team, your participation in the study is extremely appreciated. Your commitment to providing the best possible yoga intervention to the participants is critical for the success of the study. We acknowledge that yoga teachers' preferences and teaching styles may vary significantly, and it may be difficult sometimes to follow a prescribed treatment protocol when you may think someone could benefit from something different. For the purposes of this study, however, we ask you to please follow this protocol as closely as possible. Do not add any poses that are not included in this manual as we have taken a lot of time to carefully choose these poses to ensure the safety of the Veterans in this study. As questions about the protocol, study, or logistics arise, please feel free to speak with anyone involved in the study (see contact information on p. 4) and we will do our best to assist in an appropriate and timely manner.

Again, thank you for your enthusiasm and dedication to the *Veterans Back to Health* study!

Sincerely,

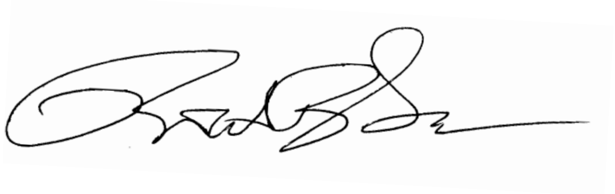

Robert B. Saper, MD MPH
Boston Medical Center Principal Investigator
Robert.Saper@bmc.org
(617) 414-6276

© Boston Medical Center
Veterans Back to Health Trial

Study Staff Contact Information

Boston Medical Center

Robert Saper, MD MPH
Principal Investigator
Robert.Saper@bmc.org
Office: (617) 414-6276

Chelsey Lemaster, MS MPH
Site Project Manager
Chelsey.Lemaster@bmc.org
Office: (617) 414-6201

Alex Femia, MS
Research Assistant
Alexandra.Femia@bmc.org
Office: (617) 414-6249

Bedford VA Hospital

Shihwe Wang, PhD
Site Investigator
Shihwe.Wang@va.gov
Office: (781) 687-3538

Dorothy Plumb, MA
Site Project Manager
Dorothy.Plumb@va.gov
Office: (781) 687-2520

© Boston Medical Center
Veterans Back to Health Trial

Veterans Back to Health Study Design

The *Veterans Back to Health* study is a 24-week comparative effectiveness randomized controlled trial of once per week yoga classes versus an educational book on self-care for chronic low back pain (cLBP) in 120 Veterans recruited from the Bedford VA Hospital. The 24-week trial starts with an initial 12-week intervention period followed by a 12-week follow-up period.

For the 12-week intervention period, Veterans are randomized in a 1:1 ratio into (1) a standardized once-per-week hatha yoga class supplemented by home practice; or (2) education delivered through a self-care book supplemented by newsletters. The study co-primary endpoints are the Defense and Veterans Pain Rating Scale and back-related function measured using the 23-question modified Roland Morris Disability Questionnaire. Yoga participants receive yoga materials (i.e., mat, block, strap) and videos to help them with home practice. Education participants receive *The Back Pain Helpbook* and newsletters every 3 weeks.

For the 12-week follow-up period, Veterans will be encouraged to continue with their yoga home practice or following the recommendations from the book on their own, without direct instruction.

The diagram below illustrates the overall study design:

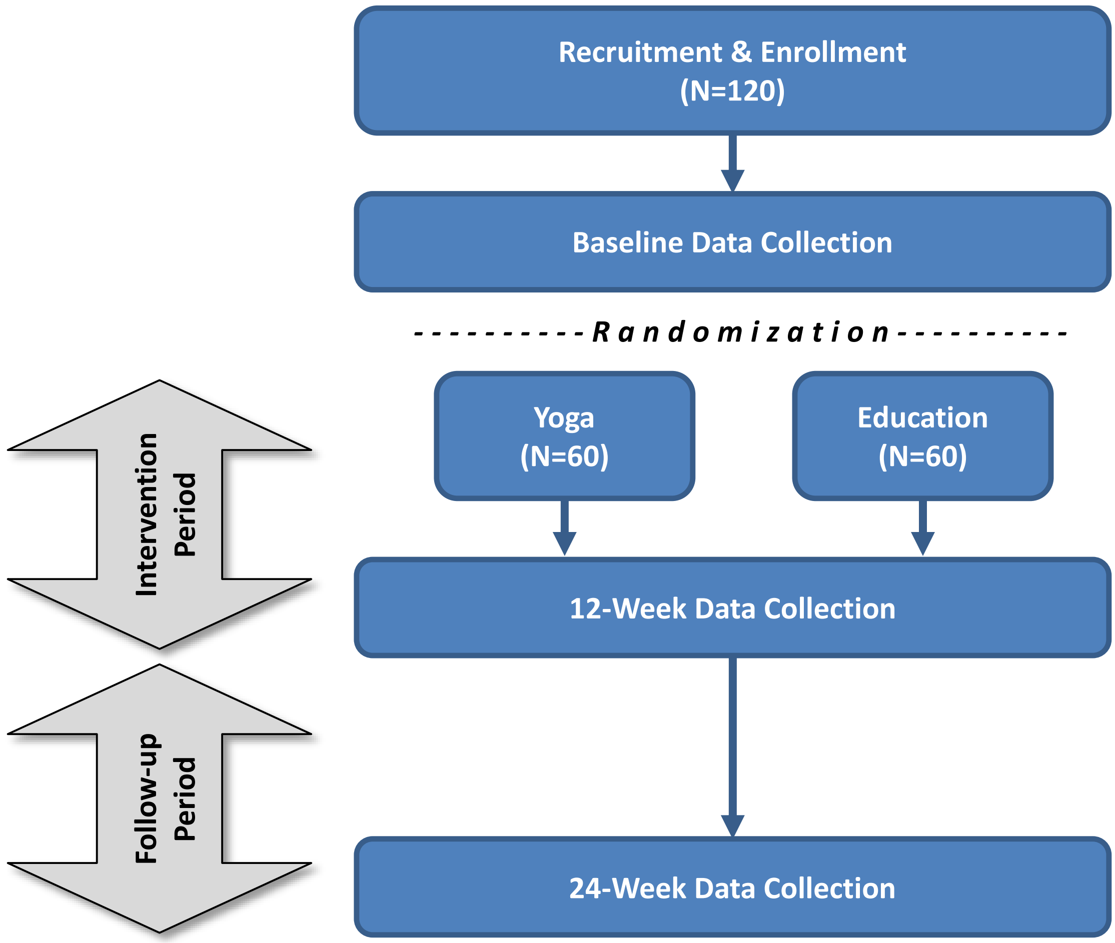

© Boston Medical Center
Veterans Back to Health Trial

Yoga Teacher Training

Study-Specific Training

All yoga teachers on the yoga study team have substantial professional experience in teaching yoga and specifically those with back pain. Training specifically for this study therefore is mostly focused on learning the specific treatment protocols used and how to best teach to Veterans. These may be more or less familiar to each yoga teacher, depending on his or her own educational background and professional experience. This manual is only a part of the preparation for this study. Study-specific training each yoga teacher must complete to participate in the study includes the following steps:

1. Read this training manual thoroughly.
2. Complete training webinars provided by Warriors at Ease and participate in a live group discussion about low back pain and teaching yoga to Veterans.
3. Attend a day-long in-person training on the study yoga protocol.

Data Collection

The data for this study will be collected by the study staff, *not* the yoga teachers. Data collection by the research staff usually occurs before yoga class and is conducted in such a way to minimize any interference to the class. However, we do ask participants to fill out a <u>Home Practice Log</u> each week and submit it when they attend class (see p. 7). Study staff will be available to collect these logs, but yoga teachers should collect any remaining logs that are not given directly to staff or are completed after class. Yoga teachers should encourage participants to complete logs truthfully and regularly. They can explain that these logs help staff get a better idea of how much practice participants are able to do and better understand how yoga helps back pain. Blank logs will be available.

Yoga teachers will also be responsible for taking attendance at each class. Veterans in this study will receive transportation reimbursement for each yoga class they attend. Study staff will hand out travel reimbursements (in the form of gift cards) each week at yoga classes. Keeping track of attendance and transportation allowances is ***<u>very</u>*** important for the research study and every effort should be made to ensure it is done accurately.

After class, yoga teachers will have an opportunity to fill out a <u>Yoga Teacher Notes</u> sheet. Teachers can write down any relevant notes and observations about the participants, the yoga protocol, and/or their own reflections about the progress of the class. These may be read by research staff but will be kept with the yoga supplies for teachers to refer back to, if needed.

Lastly, yoga teachers will be responsible for contacting research staff if they learn of any potential adverse events. An “adverse event” is defined as any untoward medical occurrence, such as illness or injury, regardless of relation to the study or yoga practice. Teachers are encouraged to call staff immediately if they become aware of any adverse events, whether they have occurred during class or outside of class.

© Boston Medical Center
Veterans Back to Health Trial

Overview of Home Practice

Veterans will be encouraged to practice for 30 minutes each day on days when they do not attend class. They will be provided with a participant handbook, yoga mat, block(s), and strap to aid home practice. Members of our study team have worked with NKP Media, Inc. to write, film, and produce a series of yoga home practice videos specifically featuring Veterans practicing the yoga study protocol. Yoga participants will be given a specific link to where the videos will be placed online. If Veterans report that they do not have consistent internet access at home, DVDs will be provided with the videos.

We will ask yoga participants to record their home practice on a Home Practice Log and turn in their completed logs each week when they attend yoga class. Home Practice Logs will be given to yoga participants with their home practice supplies after randomization, so participants should have enough logs to fill out at home. However, yoga teachers should have blank logs on hand when participants come to class, in case they forgot to bring them from home or need to take more to fill out.

After class, yoga teachers should remind participants to turn in their completed Home Practice Logs and practice at home each day if possible. Yoga teachers should also encourage participants that did not already turn in a completed log to fill one out before leaving. All collected logs should have the participant's name written at the top, otherwise the staff will not be able to tell who the log belongs to.

Here is an example of what a completed Home Practice Log might look like:

John Doe

Weekly Home Practice Log

We suggest that you practice yoga for 30 minutes on days you do not have class. It is okay if you are unable to practice every day. We just ask that you are honest when filling out this log.

Please record when and for how long you did yoga at home, starting from your last class.
Please do not include yoga practice done during class on this log.

	Date: 1/1/15	Date: 1/2/15	Date: 1/3/15	Date: 1/4/15	Date: 1/5/15	Date: 1/6/15	Date: 1/7/15
Have you practiced yoga at home today?	☒ Yes ☐ No	☐ Yes ☒ No	☒ Yes ☐ No	☒ Yes ☐ No	☒ Yes ☐ No	☐ Yes ☒ No	☐ Yes ☒ No
Minutes:	25 min.	____ min.	15 min.	30 min.	25 min.	____ min.	____ min.
Comments:							class

Please tell a yoga teacher or a research staff member if you experience any injury during the course of the study. You can call research staff at **781-687-2000 ext. 5520**.

Version 1.0

STAFF USE ONLY	
COLLECTION	DATA ENTRY
Initials: ________ Date: ________	Initials: ________ Date: ________

© Boston Medical Center
Veterans Back to Health Trial

Yoga Teacher Sickness and Absence Policy

Foreseeable Absences

Your commitment to be present and teach at all assigned yoga class is critical for the participants and success of the study. However, we do understand there may be unusual or unforeseen circumstances necessitating that you miss class. Should you need to be absent from a class, please notify Dorothy Plumb (contact information on p. 4) at least two weeks prior to the class date. She will send a substitute request email to the participating yoga teachers to arrange for an alternate. If no one has responded within 48 hours, she will follow up with phone calls to find a fill-in.

Sickness

If you are ill and unable to teach a class, please do your best to contact Dorothy Plumb at least 24 hours in advance. If this is not possible, notify Dorothy no later than 9:00 AM the day *of* class if you teach in the evening or no later than 12:00 PM the day *before* class if you teach in the morning. She will try to arrange for a substitute teacher for your class.

Emergency

In the rare event of an emergency the day of a class, please call Dorothy Plumb. Please do not use email, text messages, or voicemail in the event of an emergency.

© Boston Medical Center
Veterans Back to Health Trial

Yoga for Veterans with Chronic Low Back Pain

Low back pain (LBP) is the most common type of pain in the United States,[1,2] resulting in substantial morbidity,[3] disability,[4,5] and cost[6,7] to society. Approximately one-fourth of U.S. adults experience LBP at least one day over a three-month period.[2] LBP accounts for 34 million doctor visits with family physicians and primary care internists each year.[3] Annual direct costs for LBP care in the U.S. are more than $50 billion and indirect costs (e.g., limited productivity) are estimated to be even greater.[8] Back pain patients incur up to 75% more medical expenditures than patients without back pain.[6,7] Back injury is the leading and most expensive cause of workers' compensation claims in the U.S.[4,5] The cost of chronic pain in the U.S. is an estimated at $560 to $635 billion annually.[9]

Chronic low back pain (cLBP) lasting more than 12 weeks affects an estimated 5-10% of U.S. adults.[1,2,5] Physicians identify a definite anatomic source for back pain in only a small minority of patients. Examples include a large herniated disc, spinal canal stenosis, or vertebral compression fracture. The majority of patients however are classified as having non-specific cLBP.[1] Non-specific cLBP accounts for a majority of back-related health expenditures.

CLBP is a complex condition;[1] the causes, mediators and mechanisms are multiple but poorly defined. CLBP results from factors in multiple interrelated domains (e.g., physical, mental, emotional, social, cultural, spiritual), and also has symptomatic manifestations in multiple domains.[10] To best treat cLBP and its related conditions, a treatment that can address more than one domain may be more helpful than a treatment such as pain medication that addresses only the physical domain. Thus, the role that psychology plays in treatment, especially for those individuals without a clear explanation for the cause of their pain, should be addressed.

Anatomy of Non-Specific Low Back Pain for Yoga Teachers

Muscles in the back, abdominals, hip, and legs work together to support the spine. Back muscles and abdominal muscles tend to weaken with age since many of them are not used often in day-to-day activities. Similarly, an LBP episode that lasts for more than two weeks can lead to muscle weakness if the individual avoids using the painful muscles. This can end up causing more LBP because the muscles of the back are less able to help support the spine.

Tight or tense muscles can also cause LBP. Chronic stress causes back muscles to tighten in a "fight-or-flight" response. Tight hamstring muscles in the back of the thighs can pull down on the pelvis, which in turn increases stress on the low back. Patients with tight hamstrings tend to develop LBP, and those with LBP tend to develop tight hamstrings. Weak abdominal muscles can cause hip flexor muscles (iliopsoas, inner thigh muscles, piriformis) to tighten in response, which then causes an increase in the normal curve of the low back and worsening pain.

Given the complex relationship between muscles in the back and surrounding areas of the body, it is important to emphasize both stretching and strengthening these muscles in order to address LBP caused by muscle problems.

Chronic Low Back Pain in Military Veterans

Musculoskeletal pain conditions are the most commonly diagnosed medical problems among the more than two million Veterans from Operations Enduring Freedom, Iraqi Freedom, and New Dawn (OEF/OIF/OND), far surpassing other medical and mental health disorders.[11-13] In a study of 91,000 Veterans receiving care from the Veterans Health Administration (VHA), 43%

© Boston Medical Center
Veterans Back to Health Trial

reported “any” pain and among those reporting pain, 63% reported moderate to severe pain.[14] Eighty percent of VHA visits include pain-related complaints.[15] Twenty percent of returning military require prescription pain medication.[16] Over reliance on opioids is common, which can lead to adverse effects ranging from sedation to dependence, addiction, and death due to accidental or intentional overdose.[16,17] Among pain conditions, cLBP is the most frequent (58%)[14] and causes substantial suffering, decreased functional capacity,[4] and lower quality of life.[18,19] Back pain is a leading cause of disability among Veterans (21%).[20,21]

The types of injuries and pain common among the Veteran population differ significantly from those commonly seen in the general population. The etiologies of LBP are incredibly varied and no broad general statements can be made. However, there are a few causes worth mentioning. The literature on injuries in Veterans focuses primarily on injuries that occur during basic training, as these injuries are common and cause many people to discontinue service. The majority of these injuries are lower extremity musculoskeletal injuries, involving the hip, knee, ankle, and foot.[22] Much of military training centers on weight-bearing physical activities, such as marching or running. Thus, the most common types of musculoskeletal injuries are associated with overuse from repetitive physical activity.[22] Running can also contribute to knee problems as well as tightness in the hip flexors and extensors.

Beyond initial training, injuries in the military are often very occupation-specific. For example, those who carry heavy packs on patrol commonly incur injuries to the knees and ankles. The population that has served in aviation tends to have issues with severe early-onset arthritis and disc disease due to frequent experience of high G-force and hard landings. These acute issues continue to persist even after leaving active duty, leading to chronic pain. Older Veterans are becoming increasingly similar to the general U.S. aging population who suffer from typical chronic diseases of aging adults, including heart disease, diabetes, and obesity.

Psychological Comorbidities and the Polytrauma Triad

Psychological distress, back pain, and disability are strongly correlated. Symptoms of post-traumatic stress,[23] depression (anhedonia, low self-esteem, irritable, sadness, lethargy),[17] and traumatic brain injury (memory problems, poor concentration, change in executive function)[12] are highly prevalent in Veterans with cLBP.[24] As a result, more than 22 Veterans commit suicide every day.[25] Sleep disorders are also invariably associated with chronic pain and mental distress.[16,26] Over 95% of prescription sedatives are used for pain and sleep.[16] Low pain self-efficacy, maladaptive pain coping behaviors (e.g., catastrophizing,[27] fear avoidance,[28] substance use[29]), and barriers to seeking evidence-based mental health care compound back pain recovery.[30,31] Among Veterans receiving care from Polytrauma Network Sites, 42% of OEF/OIF Veterans had the clinical triad of chronic pain, post-traumatic stress disorder (PTSD), and persistent post-concussive symptoms.[16,32] Given the high rate of co-occurrence, it is not surprising that many post-concussive and PTSD symptoms overlap such as depression, anxiety, insomnia, appetite changes, irritability, concentration difficulty, and fatigue.[33] The polytrauma triad is considered the “signature injury” in returning OEF/OIF/OND military personnel.[34]

The hallmarks of PTSD are re-experiencing (flashbacks, nightmares), avoidance (feeling emotionally numb, losing interest in activities), and hyperarousal (being jumpy, on-edge, insomnia) symptoms.[23] PTSD has been described as a sensory processing disorder. According to

© Boston Medical Center
Veterans Back to Health Trial

the National Institute of Mental Health, "*When in danger, it's natural to feel afraid. This fear triggers [the] 'fight-or-flight' response, a healthy reaction meant to protect a person from harm. But in PTSD, this reaction is changed or damaged. People who have PTSD may feel stressed or frightened even when they're no longer in danger.*"[35] In a yoga class, PTSD would most likely manifest as irritable behavior, possible angry outbursts with minimal or no obvious provocation, difficulty with concentration, or an exaggerated startle response. Yoga can promote effective sensory, motor, and cognitive processing, which greatly aids the healing process. A direct and powerful way for Veterans to better self-regulate is through control of the breath.

Moral injury is a relatively new concept in the conversation about the effects of war.[36] Moral injury has been defined as "perpetrating, failing to prevent, bearing witness to, or learning about acts that transgress deeply held moral beliefs and expectations."[37] This is not a diagnosis, but rather an explanation for many Veterans' emotional responses to the effects of war that are not sufficiently captured by PTSD.

The increased use of high energy explosive devices and repetitive exposure to blasts in recent conflicts has yielded an unprecedented level of blast-related injuries. A blast (or pressure) wave can cause a traumatic brain injury (TBI), which varies from very obvious external head injuries to mild concussions that show no immediate visible effects.[12] The vast majority of people who sustain a mild traumatic brain injury (mTBI), also known as a concussion, recover completely with little or no intervention. Sustaining multiple concussions, not all of which may cause significant alteration of consciousness, can cause long-term subtle effects including difficulty concentrating, irritability, slight personality changes, or perhaps impaired judgment wherein the brain does not properly filter out inappropriate comments.[38] Severe or repetitive brain injuries can lead to persistent post-concussive symptoms, including headache, heightened sensitivity to light and sound, dizziness, and memory impairment.[33] Individuals with a recent or severe history of brain injury should be cautious about lowering their head below their heart during daily or physical activities, as this may cause a headache. A blast wave can also cause injuries to the inner and middle ear, even if there are not any obvious external injuries or if hearing protection devices are worn. In addition to hearing loss, damage to the structures in the inner ear can cause dizziness and issues with balance.[39] This may be especially pronounced if the head is turned far away from the midline of the body (e.g., a far side-bend in Triangle Pose or Crescent Moon Pose). Veterans that have sustained a brain injury benefit from a sense of safety in group settings with a regular routine, firm start and stop times, and the absence of criticism. In this case, this is best achieved by close adherence to the protocol as designed.

Treatment for Chronic Low Back Pain

Although there are a range of conventional pharmacologic, non-pharmacologic, and surgical procedures used for non-specific cLBP, most patients report only modest or moderate relief at best. Evidence-based clinical practice guidelines recommended initial management for non-specific cLBP should include advice to remain physically active and education on back self-care.[40] These guidelines recommend several conventional non-pharmacologic therapies including exercise therapy and cognitive-behavioral therapy as well. If necessary, medication with strong evidence for benefitting cLBP, such as acetaminophen or non-steroidal anti-

© Boston Medical Center
Veterans Back to Health Trial

inflammatory drugs (e.g., ibuprofen, naproxen), may be judiciously used. Nonetheless, patient satisfaction with the effectiveness of conventional cLBP treatment is relatively low.[41]

The 2010 Army Surgeon General's Pain Management Task Force Report stressed the importance of a Department of Defense–Veterans Administration (DoD-VA) partnership to develop an "integrative and interdisciplinary approach" to pain management, including incorporating "integrative and alternative therapeutic modalities into a patient-centered plan of care."[42] The Report emphasized "pain cannot be managed without addressing its relationship to stress" and shifting to more active than passive treatments "improves the outcome, decreases provider dependent care, and empowers the patient with a sense of control over his or her condition." The Task Force identified yoga as one of several "Tier 1" complementary modalities as priorities for DoD-VA research and possible integration.[42] Yoga was also included as an evidence-based therapy in DoD-VA clinical guidelines for cLBP.

Yoga for Low Back Pain

Yoga is increasingly common[43] with over 8.7% of U.S. adults reporting use in 2012.[44] Yoga is a "mind-body" practice consisting of three main components: (1) physical postures (*asanas)*; (2) specialized breathing techniques (*pranayama*); and (3) meditation. A 2005 RCT found a moderate benefit of yoga for improving back-related function in civilian adults with cLBP.[45] This prompted the VHA, American College of Physicians, and American Pain Society to list yoga in clinical practice guidelines as an evidence-based treatment for cLBP.[40] Subsequently, four moderate-large RCTs (n=90-313)[46-49] and five smaller RCTs (n=20-60)[50-54] have also shown yoga to be effective for reducing pain and improving function in civilian adults with cLBP. Meta-analyses support these conclusions.[55,56] Several yoga-cLBP studies found associated psychological benefits in mood[46,54] and self-efficacy.[48,57] Yoga research on psychological health is growing, showing promising evidence for benefit in depression,[58-66] post-traumatic stress,[67,68] anxiety,[66,69-71] and insomnia.[72,73] Yoga classes can also increase social connectedness and spirituality.[74]

Current clinical management of cLBP in Veterans is predominantly a provider-centered, pharmacologic, passive approach with the goal of reducing perceived pain intensity. This is in contrast to the VHA Strategic Goal #1 for 2013-2018: "Provide Veterans personalized, proactive, and patient-driven health care."[75] To move closer to this vision, the VHA established the Office of Patient-Centered Care and Cultural Transformation in 2011.[76] According to Director Tracy W. Gaudet, MD, "*Until we employ a personalized strategy that considers the Veteran's unique conditions, needs and circumstances, addressing the full range of physical, emotional, mental, social, spiritual and environmental influences, we will not optimally help our Veterans to minimize disease or regain and maintain their health. Our goal is to design a system where we partner with our Veterans to be mission ready for their lives, optimizing their health in service of what matters to them.*"[77] Many studies suggest yoga may favorably impact physical as well as psychosocial problems, offering the more patient-centered, integrative approach to cLBP treatment that the VHA strives to offer.

© Boston Medical Center
Veterans Back to Health Trial

Yoga for Veterans with Chronic Low Back Pain

Studies of Yoga for Veterans with Low Back Pain

The IOM report *Pain in America* recognized that protocols for pain management approaches must be adapted to the unique needs of Veterans[13] and undergo rigorous testing for clinical effectiveness and cost effectiveness. It is unknown if results from previously published civilian yoga-cLBP trials will generalize to military populations. In contrast to participants in civilian studies, Veterans with cLBP are more likely to be men with different mechanisms of injury (e.g., direct combat-related trauma; severe non-combat back loading from carrying 100 pound gear), greater pain severity and disability; and more serious comorbid psychological symptoms. There are currently two published reports of yoga for cLBP in military settings, both conducted at the VHA San Diego.[78,79] These uncontrolled yoga-cLBP clinical programs in Veterans showed promising improvements in pain and depression.

Despite enthusiasm for offering yoga to Veterans, yoga instruction to date is not well standardized or implemented widely in the VA. More importantly, there is little evidence for yoga's effect on cLBP, PTSD, depression, or other psychosocial concerns in Veterans. Thus, *Veterans Back to Health* will (1) establish a structured reproducible yoga protocol uniquely suited to Veteran populations with cLBP and associated psychological comorbid symptoms; (2) develop web-based delivery systems to assist Veterans in practicing yoga at home; (3) increase our knowledge of the feasibility and impact of yoga for Veterans' cLBP, psychological comorbidities, and family functioning; and (4) provide a strong foundation for larger multi-site studies and implementation projects. Achievement of these aims has the potential to impact the approach and management of cLBP in accordance with the DoD-VA vision, i.e., a more integrative, interdisciplinary, focus on active self-care approaches that empower patients to have greater control of their condition.[42] For these reasons, our study will determine if yoga can become a safe, clinically effective, cost-effective, and scalable non-pharmacologic approach to address the physical *and* psychosocial dimensions of cLBP in Veterans.[80]

© Boston Medical Center
Veterans Back to Health Trial

Teaching Yoga to Veterans

As any experienced yoga teacher knows, it is important to always be aware of the population you are teaching. It is always a good idea to come to class prepared with knowledge about the population's culture, needs, strengths, and limitations. This is especially true when working with a unique population such as Veterans. Most, if not all, Veterans in this study will have never practiced yoga before. Teachers can help them learn by encouraging them to explore new movements, educating them about these movements, and providing them with choices to personalize their practice. Teachers should also be prepared to handle an adverse reaction, especially during breathing and relaxation exercises.

Teachers may tell Veterans why we move a certain way in a yoga pose and why certain movements may be beneficial for the back while others may not be. Teachers may explain that back pain may be made worse by tight muscles in the low back, hips, and legs, and explain how the poses may address this issue. For example, teachers may wish to point out that Baby Dancer Pose emphasizes a stretch of quadriceps (a.k.a. "quads") in the front of the thigh and how other poses stretch "opposing" muscles in the back of the thighs (e.g., Downward Dog and Forward Bends stretch hamstrings). It is also beneficial for Veterans to understand how integrating breathing techniques with movements as taught by teachers can help prevent injury and prompts the nervous system to induce relaxation. Many Veterans will want to understand how and why yoga works for them so that they can apply this knowledge outside of class and maybe even pass it on. Veterans may have negative preconceptions about yoga, which teachers should be prepared to respond to during class. Providing the Veterans in this study with specific information about yoga practice empowers them, presents yoga as a legitimate activity for Veterans, and even gives them something concrete to tell others who may not be as willing to try yoga.

When teaching yoga to Veterans, the yoga teacher is responsible for creating a safe, caring, and non-judgmental yoga class environment. One important aspect of this environment is offering validation and acknowledgement to the Veterans. For those that are skeptical of yoga practice or have heard negative or false rumors about yoga practice, it is okay to acknowledge that yoga practice can be different, challenging, and sometimes strange ("woo-woo" or "new-agey") to them. Teachers can use the theme of Segment 1 ("*Opening to Something New*") to encourage Veterans to give yoga a try, even if it is different than what they are used to. Sanskrit words should not be used during the yoga classes for this study.

Teaching Yoga Poses: Language, Modifications, Supporting Self-Awareness

Military culture places an emphasis on following orders, never giving up or showing weakness, and mottos such as "getting it right the first time" and "no pain, no gain." This type of thinking is not helpful when learning to practice yoga. When Veterans enter a VA where there are trappings of that "prior life," many may subconsciously revert back to some of their old thinking patterns. The yoga teachers in this study should use calm and inviting language that is friendly, clear, strong, and never condescending. Veterans may not respond well to flowery language and metaphors during yoga practice. Plain and easy to comprehend directions are more likely to be well-received. Even as teachers modify their instruction to suit their class of Veterans, it is important to be genuine in your choice of words. Veterans will immediately recognize and appreciate your authenticity.

© Boston Medical Center
Veterans Back to Health Trial

Teaching Yoga to Veterans

One way to create a safe and trusting environment is to encourage Veterans to feel in control of their own body and yoga practice. All poses in this study have modifications and variations to choose from. Teachers should emphasize that a modification to a pose (e.g., adding a prop, repositioning the arms) does not mean that they are doing it wrong or that they have to settle for an easier version. Veterans could make negative associations with the word “modification” so teachers may want to offer them as “options” available to make the pose more comfortable or accessible. It may serve the group better to have everyone try a modification and then tell the class, “Do the version that feels best to you.” The Veteran will feel more in control of their movements and will be encouraged to become familiar with their own strengths and limitations, which may vary from day-to-day. Teachers should notice if individuals are actually opting for modifications that they truly need or seem to be averse to modifications altogether. Language that allows the Veteran to have control includes invitatory phrases such as “if you’d like,” or “when you are ready.” During yoga poses, teachers might also say, “*If you are experiencing any discomfort or pain, please be willing to come out of the pose*” or, “*As we hold the pose, remember that you are welcome to bring yourself out of the pose whenever you are ready.*”[81]

A common misconception about yoga is that it is only for “flexible people” that “stand on their heads.” Consistent with the military culture to “get it right” and conceal weakness, Veterans may become discouraged by their lack of flexibility or limitations in some poses. Teachers should acknowledge these frustrations and encourage Veterans to become more familiar with their bodies’ sensations. Teachers may also need to continually emphasize that yoga practice is unique for each person and Veterans should not compare their personal abilities or preferences to others. This will help remove any competition from the class, which is prominent in military culture. Some example language or phrases that teachers can use are:

- *Notice how this pose feels and say “hello” to any sensations in your body*
- *You may feel this or you may not, just observe* (instead of telling them what they should notice or feel)
- *Think of these sensations as your body talking to you, telling you what is okay for today* (letting them know that what does not work today might be easier later on)
- *If this difficult for you right now, that is okay. You can consider trying…* (offering an option to customize the pose)

It is important for Veterans to build body awareness through their yoga practice. This includes changing their relationship with their pain. Yoga teachers should encourage them to get to know their body by identifying where pain or discomfort is located, what positions naturally feel good for them, and what emotions may be connected to their physical feelings. Acceptance of pain may be a completely new concept. Through yoga practice, Veterans will be able to learn how to understand their body and differentiate pain (e.g., from an injury) from the feeling of a stretch, soreness, or other expected sensations.

Some Veterans may not be comfortable with teachers touching them to help adjust their poses. Never assume it is permissible to touch, even if you have helped the person in previous classes. Teachers can ask by saying things like, “Can I help you adjust your right leg?” General questions such as “Can I touch you?” are not recommended. Always let the Veteran know it is fine if they

© Boston Medical Center
Veterans Back to Health Trial

are not comfortable with touch and they can adjust their poses with verbal direction if they prefer.

Guiding Breathing and Relaxation Exercises

Teachers should always be cognizant of the possibility of post-traumatic stress reactions in a yoga class and should understand the relationship between trauma and the body. This is more likely to come up during breathing or relaxation exercises than during yoga postures. Yoga teachers can use the following techniques to help Veterans feel relaxed:

- As opposed to a yoga class in the general population, do not give Veterans a lot of quiet space without guidance. Otherwise, their minds could drift to a traumatic or stressful experience in the past.
- Tell them to anchor themselves to their breath or following the instructor's voice. Remind them to stay present in the moment and return to their anchor if they have drifted. Teachers may like to give little rocks or crystals to Veterans as a tangible reminder to be present and to stop intrusive thoughts.
- Provide Veterans with the choice to close their eyes or keep them open. Similarly, some Veterans may be uncomfortable lying on their back due to past trauma. Provide them with options to help them feel safe and comfortable. To be covered with a blanket may help some Veterans feel safe.
- Allow Veterans to sleep during relaxation if that is what occurs. Insomnia can be a significant issue for many Veterans.
- Know what to do if a Veteran has an abreaction. Use a gentle but firm voice to help them come back to the present moment. Encourage them to return to their focus on their breath. A teacher could say, "Return to the focus on your breathing. Breathe in through the nose and out through the mouth, noticing the temperature of the air as you breath in, noticing the rib cage expanding and contracting with each breath…"

In general, teachers should encourage Veterans to use techniques they learn outside of class to help them recognize how they deal with the world (e.g., using breathing techniques when they get angry, inpatient, etc.). This is a good reminder for the end of class.

After Class Reflections

After the relaxation exercise in class, teachers should prompt Veterans to share their thoughts or reflections about their personal yoga practice if they feel comfortable. Encourage Veterans to offer honest feedback and let them know it is okay to perceive that they "did not do it right" or "could have done it better." Validate that they are exactly where they are supposed to be and the body will open when it is ready. Hearing other Veterans in the class describe how they feel can help others that might not be able to find the words to describe their feelings. It can also help normalize the challenges of yoga practice. Teachers may even choose to offer stories about their own challenges with specific yoga poses. For example, "*I use to get really frustrated when I fell out of balance poses. Then I realized that's how I deal with life off the mat. It provided me with great insight.*" This can encourage trust and help the Veterans feel a connection with others, both of which may not come easily to them. Always let Veterans know it is fine if they do not want to say anything. Teachers can also use this reflection time to draw

© Boston Medical Center
Veterans Back to Health Trial

comparisons to last class and highlight change, especially after many weeks have passed. It may be helpful for teachers to let Veterans know that it is normal for their back to feel a bit worse before it gets better. A teacher could say, “*In yoga practice, you are waking up muscles that might not have been used in a long time. It is normal for these muscles to be a bit sore while they get used to being used again.*”

© Boston Medical Center
Veterans Back to Health Trial

Yoga Teacher Roles and Responsibilities

Typically, two yoga teachers will teach each class. One teacher will be responsible for leading the class through the poses for the week. The other teacher will help individual participants with modifications as appropriate. Teachers may choose to alternate roles week to week; this is left up to teacher discretion based on experience and preference. As a reminder, yoga teachers should always dress appropriately for the population they are teaching. Do not wear revealing attire and avoid clothing or jewelry with symbols (e.g., peace signs, religious symbols, etc). Teacher roles and responsibilities are outlined as follows:

On the First Day of Class (Arrive 45 minutes early)

- Have one chair behind each mat so that can start by sitting down.
- Welcome participants into the space and introduce yourselves.
- Inform participants about logistics (e.g., location of bathrooms, where to put their items), taking off shoes, not eating in class, cleaning mats, turning off cell phones, etc.
- Assure participants about confidentiality within the group, letting them know they can talk outside of class about their own experience, but not about other classmates.
- Review “Before Each Class” below.

Before Each Class (arrive at least 15 minutes early)

- Check in with the other yoga teacher to share any relevant information about participants as well as any other issues or concerns.
- Turn on lights, clear space, and set up the props (mats, straps, blocks, blankets, chairs). Make sure there is sufficient room between mats for a teacher to be able to walk around and model postures. Create space for participants’ belongings away from the yoga space.
- Have attendance sheet, pens, and extra Home Practice Logs near the door and in the same place each week.
- Be sure everyone signs the attendance sheet legibly and remind participants to note if contact information has changed. Make sure the attendance sheet is dated and that all participants present are recorded.
- Place index cards with participants’ first names next to their mat. Consider placing participants who need extra help closer to the teacher or near a wall for support.

During Class

- Position yourself so that participants can see you and you can see them during class.
- Assist participants with props and provide support to participants when needed.

After Class

- Remind participants to practice at home and fill out Home Practice Logs.
- Share cleaning materials with participants and show them how to clean their mats. Store yoga supplies and forms securely, and leave the space as it was found.
- Use the Yoga Teacher Notes sheet to record any relevant information and/or observations from the class. Call study staff directly if any supplies or forms need to be replenished or if you need to report any potential adverse events.

© Boston Medical Center
Veterans Back to Health Trial

Veterans Back to Health Yoga Protocol

Protocol Development

Veterans are encouraged to participate in this study if they are experiencing chronic low back pain (cLBP) and wish to achieve a higher level of functioning and minimization or alleviation of pain. We have designed a hatha yoga protocol specifically for this study. An earlier version of the yoga protocol was developed in 2006-07 by an expert panel led by the Principal Investigator, Dr. Saper, after a systematic review of lay and scientific literature on yoga and low back pain. Panel members had experience in several styles of hatha yoga including Anusara, Ashtanga, Iyengar, and Kripalu. This protocol was used in a 2007 pilot study of yoga compared to usual care for 30 patients with cLBP. It was further refined through a 2012 yoga dosing study where 95 participants attended either once or twice-weekly yoga classes. The yoga dosing study found both class frequencies similarly effective for improving back pain and function. A version of this yoga protocol was also used in the original Back to Health study, a large study for 320 participants that compared yoga, physical therapy, and education. Additional changes have been made to the protocol based on yoga teacher and participant feedback from previous studies. In order to adapt the yoga protocol to a Veteran population, a panel of individuals with expertise in yoga and working with Veterans had a daylong convening to discuss additional modifications.

Adherence to Protocol

As a member of the yoga team, your participation in the study is extremely appreciated. Your commitment to providing the best possible yoga intervention to the participants is critical for the success of the study. We acknowledge that yoga teachers' preferences and teaching styles may vary significantly, and it may be difficult sometimes to follow a prescribed treatment protocol when you may think someone could benefit from something different. For the purposes of this study, however, we ask you to please try to follow this protocol as closely as possible. **However, teachers should always prioritize the safety and comfort of Veterans when teaching a yoga class.** As questions about the protocol, study, or logistics arise, please feel free to speak with anyone involved in the study (see contact information on p. 4) and we will do our best to assist in an appropriate and timely manner.

These yoga classes for cLBP are different from regular yoga classes in that the curriculum includes standardized format and teaching directives that can be replicated and delivered by any qualified yoga teacher. The intervention phase is 12 weeks long. Participants in the yoga group will take one 75-minute yoga class per week. The twelve weeks are divided into four segments. Each segment has a name and lasts three weeks. The segments build upon what participants have learned in previous segments. Each segment has a unique set of poses and yoga philosophies. The goal of the four segments is to teach basic hatha yoga postures in a carefully calibrated sequence that supports healing of the back. The four segments are entitled: *Opening to Something New (Segment 1)*, *Listening to Your Back (Segment 2)*, *Engaging Your Power (Segment 3),* and *Bringing it Home (Segment 4)*.

© Boston Medical Center
Veterans Back to Health Trial

The Four Segments

	Segment 1: Opening to Something New			Segment 2: Listening to your Back			Segment 3: Engaging your Power			Segment 4: Bringing it Home		
Week	1	2	3	4	5	6	7	8	9	10	11	12

Segment 1: Opening to Something New (Weeks 1-3)

In this first segment, participants are introduced to yoga and the possibility of change in their back pain and change in themselves. They are taught how controlled breath work is the foundation for yoga. Participants practice a variety yoga breathing exercises. They are introduced to intentional relaxation and practice several poses such as Child's Pose, Bridge Pose, Knees to Chest, Knees Together Twist, and Pelvic Tilts. Each week briefly highlights an aspect of yoga philosophy (defining yoga, self-compassion, awareness). How the yoga philosophy concept applies both to yoga practice and daily life is emphasized.

Segment 2: Listening to your Back (Weeks 4-6)

Participants are taught how to listen to their individual bodies and their back and to continue to develop self-compassion. They are encouraged to begin building strength in standing postures, developing stability in balancing poses, stretching in gentle backbends, and learning to use the muscles to support the spine—all elements to help their low back pain. Participants are taught to use their experience on the mat to create a foundation of balance in their lives by trusting their own experience and listening to their bodies. Participants continue with postures learned in the first segment and learn new postures such as Big Toe Pose, Side Hip Strengtheners, and Eye of the Needle Pose. Yoga philosophy principles highlighted in Segment 2 include acceptance, self-control, and honesty.

Segment 3: Engaging Your Power (Weeks 7-9)

Participants are challenged to apply the strength and self-awareness that they have started to discover in more challenging standing, twisting, and back bending postures. They are encouraged to focus on the strength and mobility in their low back as they try more difficult postures such as Plank Pose, Side Plank Pose, and Warrior II. Participants are introduced to the yogic concepts of gratitude, balance, and moderation.

Segment 4: Bringing it Home (Weeks 10-12)

Participants perform more challenging postures including Triangle Pose and Baby Dancer Pose. The notions of healthy lifestyle, compassion for others, and community are presented in this segment.

© Boston Medical Center
Veterans Back to Health Trial

Yoga Class Structure

Each class throughout the 12 weeks has a similar structured format:

Curriculum Elements	Time (min)
Check-in<br>Yoga Philosophy<br>Breathing Exercise	8
Yoga Postures	55
Relaxation<br>Closing	12
**Total Time**	**75**

Check-in

Check-in takes places at the beginning of each yoga class. Teachers should check-in with each participant. The yoga teacher should ask participants how their yoga practice is proceeding. Participants should be particularly encouraged to share both progress and any setbacks or injuries from their yoga home practice. Encourage participants to let the teacher know if they are experiencing any new symptoms during or after the class. Create an atmosphere of *safety* and *acceptance* so participants can express both positive and negative experiences. Acknowledge and validate all responses without judgment. Of course, it is fine if a participant does not want to share anything. Comment as needed.

Introduction to Yoga Principles

This section is intended to familiarize participants with the philosophical principles of yoga. These principles provide the participants with a framework for health and wellness while promoting awareness and self-discipline. Teachers utilize poems, readings, and commentaries related to principles to inspire participants to practice greater self-care. Suggested readings for each week are in this manual and supplementary readings are in Appendix 1. The manual gives "talking points" that can be used either verbatim or as suggestions to guide your teachings. The principles are discussed in a completely secular manner and using language that Veterans will be receptive to. We will cover the following topics:

- Week 1 (Defining Yoga)
- Week 2 (Self-Compassion)
- Week 3 (Awareness)
- Week 4 (Acceptance)
- Week 5 (Self-Control)
- Week 6 (Honesty)
- Week 7 (Gratitude)
- Week 8 (Balance)
- Week 9 (Moderation)
- Week 10 (Healthy Lifestyle)
- Week 11 (Compassion for Others)
- Week 12 (Community)

Please see a summary of The 12 Yoga Principles on pg. 23.

Breathing Exercise

Over the course of the 12-week curriculum, awareness of normal breathing, lengthening the inhale and exhale, and the ocean breath are taught. Teachers should model and demonstrate

© Boston Medical Center
Veterans Back to Health Trial

all breathing techniques. Yoga breathing techniques must be emphasized with every posture as well as during breathing exercises.

Yoga Postures

The yoga postures have been selected for their safety and potential to help chronic low back pain. They gradually increase in difficulty over the 12-week intervention. Support the participants through individual attention, yoga props as needed, and modifications to accommodate individual needs. Help participants learn that props are their friends. The postures are intended to transition participants from more strenuous poses to the closing relaxation. Do not introduce poses that are not included in this manual.

This manual lists a menu of poses for each week for teachers to use as a reference during class (pp. 24-35). It is not expected that all poses will be taught in each individual class. These menus provide a range of opportunities for teaching to Veterans. Highly mobile Veterans may be able to progress to do the full range of poses in this manual. A more disabled Veteran may never be able to do certain poses. Therefore, it is up to teachers' discretion to lead the Veterans through the protocol as appropriate.

Relaxation Exercise

The relaxation exercise is led by the yoga teacher while the participants are in Final Resting Pose (*Savasana*) or another comfortable position. Never use the term "corpse pose" for this position. Relaxation provides participants an opportunity to integrate the different experiences of yoga practice. This part of the practice is meant to draw participants into a deeper state of relaxation. Particularly at the beginning of the study, yoga teachers should give clear guidance to participants throughout the relaxation section.

Relaxation is a crucial component of the program, providing participants with regularly occurring opportunities to renew, restore and recharge with yoga. Participants are encouraged to set aside actions, thoughts, stress, worries and the events of the day and focus on the present moment in class. Different relaxation techniques can be used, including muscle relaxation and body scan. There is a menu of relaxation exercises listed in this manual. In contrast to classes with civilians, long periods of silence should be avoided with Veterans.

Closing

The purpose of the closing is to allow time for reflection and sharing of the Veterans' yoga experience. Ask participants in a non-judgmental way if they would like to share their thoughts, experiences, or feelings after the class. Let them know it is okay if they do not want to speak. Each week, teachers should remind participants to practice at home and fill out Home Practice Logs. Encourage participants that did not bring a completed log to fill one out for the previous week before leaving class, even if they were not able to practice at home. Say goodbye to everyone by name, making eye contact.

© Boston Medical Center
Veterans Back to Health Trial

The 12 Yoga Principles

for the *Veterans Back to Health* study

These principles are explicitly introduced at the beginning of each class through yoga philosophy. Below are talking points to guide you. You may use these verbatim or supplement these topics based on your own experience. There are also specific supplemental readings for each week (p. 75) that reinforce these principles. Incorporate these principles throughout the class as appropriate. If you would like more guidance than the below, please speak with study staff.

- **Week 1 (Defining Yoga):** Veterans are given a context of the yoga intervention. They are introduced to yoga as tool to help relieve their back pain through a union of body, mind, and breath. They learn how to enter, be in, and exit postures safely and mindfully. Introduce the concept that lessons learned on the yoga mat may be helpful off the mat for overall health and wellbeing.
- **Week 2 (Self-Compassion):** Veterans are encouraged to practice compassion toward themselves and others. The difference between harmful pain and healthy discomfort in poses is discussed to help guide their relationship with pain.
- **Week 3 (Awareness):** Veterans are introduced to the idea of self-awareness and encouraged to be aware of when their bodies are telling them to make adjustments in their poses. Similarly, with greater awareness in life, we can work toward changing old unhealthy habits.
- **Week 4 (Acceptance)**: Veterans are encouraged to accept themselves, their challenges (including back pain), and the current moment, practicing this acceptance in each pose and guiding them towards a different relationship with pain. They are encouraged to also carry this self-acceptance into daily life by accepting other people and their challenges.
- **Week 5 (Self-Control):** Like achieving any goal in life, Veterans are taught that achieving greater relief of their back pain with yoga requires practice, willpower, and patience. With self-control, we can move toward doing poses that might be initially difficult.
- **Week 6 (Honesty):** Veterans are encouraged to be honest with themselves in their practice of yoga, as well as to recognize their own limitations and accept that they may have to do poses differently than others in the class.
- **Week 7 (Gratitude):** Veterans are encouraged to feel grateful for what they have right now. In yoga practice, Veterans are encouraged to focus on what they can do, instead of what is challenging in the moment.
- **Week 8 (Balance):** The concepts of effort and ease in yoga postures are discussed. Veterans are taught how to balance effort and ease in their yoga practice and encouraged to seek similar balance in their daily lives.
- **Week 9 (Moderation):** Veterans are encouraged to be in control of their desires and to seek balance in their lives. In yoga practice, it is better to practice a little bit of yoga frequently than to practice a lot of yoga every once in a while.
- **Week 10 (Healthy Lifestyle)**: Veterans are taught to become aware of the healthy lifestyle elements of yoga. They are encouraged to incorporate healthy habits into their lives outside of yoga (e.g., healthy food, healthy relationships, clean environment, etc).
- **Week 11 (Compassion for Others):** Veterans are encouraged to practice being the person they would like to be (e.g. compassion not judgment, friendliness instead of anger, joy for others instead of jealousy).
- **Week 12 (Community):** Veterans are encouraged to look to each other for support in their practice. This gives a sense of common ground and connection not only within ourselves but with all people.

© Boston Medical Center
Veterans Back to Health Trial

Segment 1: Opening to Something New

Week 1

Check-in, Yoga Principle, Breathing Exercise	8 minutes

- Introduce teachers and explain setup of class
- Take attendance, learning how to pronounce names
- Welcome participants to the yoga intervention to learn techniques for managing back pain
- Share goals and ask for their commitment to the program
- Discuss <u>Defining Yoga</u>: Veterans should be given a context and overview of the yoga intervention. Introduce yoga as a tool to help relieve their low back pain through a union of body, mind, breath, and soul. Let them know that although yoga may not *cure* their back pain, regular practice can substantially reduce their pain and improve how they function in daily activities. Teach them how to enter, be in, and exit postures safely and mindfully.
- Breathing exercise (p. 69)

Yoga Postures	55 minutes

1. Knees to Chest Pose
2. Knees Together Twist Pose
3. Pelvic Tilt Pose
4. Cat/Cow **OR** Wheel Pose
5. Mountain Pose
6. Shoulder Opener Pose
7. Crescent Moon Pose (Hands on Hips)
8. Chair Pose (Hands on Hips)
9. Warrior at Wall Pose
10. Triangle Pose (at Wall)
11. Forward Bend at Wall Pose
12. Child's Pose (in Chair)
13. Chair Dog Pose
14. Locust Pose (One Leg at a Time)
15. Sphinx Pose
16. Bridge Pose (Supported)
17. Knees Together Twist Pose
18. Knees to Chest Pose

Relaxation Exercise	8-10 minutes

- Prepare the body for relaxation by leading into Final Resting Pose or another comfortable position.
- Yoga teachers should give clear guidance to participants throughout the relaxation exercise (p. 71).
- Encouraged participants to set aside actions, thoughts, stress, worries and the events of the day and focus on the present moment in class.

Closing	2-4 minutes

- Praise participants for doing their first yoga class and ask them to share their initial thoughts or reflections if they are comfortable. Let them know all classes will end with an opportunity to share with the group and explain how this can be helpful.
- Remind participants to practice yoga at home for 30 minutes each day as they are able and to fill out a Home Practice Log to turn in at the next class. Have blank logs out for participants to take if they need to.
- Remind participants to be aware of their breath during their yoga practice and also throughout the day.
- Say good-bye to all participants <u>by name</u>.

© Boston Medical Center
Veterans Back to Health Trial

Segment 1: Opening to Something New

Week 2

**Check-in**	**8 minutes**

- Welcome participants by name and take attendance.
- Ask participants what came up for them since last class in regards to the yoga intervention, practicing at home, and keeping practice logs.
- Discuss setting up a realistic practice schedule.
- Discuss Self-Compassion: Encourage Veterans to practice compassion toward others and themselves. Discuss the difference between harmful pain and healthy discomfort in poses to help guide a healthy relationship with pain ("body sensations").
- Breathing exercise (p. 69)

**Yoga Postures**	**55 minutes**

1. Knees to Chest Pose
2. Knees Together Twist Pose
3. Pelvic Tilt Pose
4. Cat/Cow **OR** Wheel Pose
5. Mountain Pose
6. Shoulder Opener Pose
7. Crescent Moon Pose (Hands on Hips)
8. Chair Pose (Hands on Hips)
9. Warrior at Wall Pose
10. Triangle Pose (at Wall)
11. Forward Bend at Wall Pose
12. Child's Pose (in Chair)
13. Chair Dog Pose
14. Locust Pose (One Leg at a Time)
15. Sphinx Pose
16. Bridge Pose (Supported)
17. Knees Together Twist Pose
18. Knees to Chest Pose

**Relaxation Exercise**	**8-10 minutes**

- Prepare the body for relaxation by leading into Final Resting Pose or another comfortable position.
- Yoga teachers should give clear guidance to participants throughout the relaxation exercise.
- Encouraged participants to set aside actions, thoughts, stress, worries and the events of the day and focus on the present moment in class.

**Closing**	**2-4 minutes**

- Praise participants for today's yoga practice and ask them to share their thoughts or reflections if they are comfortable. Reiterate how this can be helpful as everyone is learning yoga together.
- Remind participants to practice yoga at home and to fill out Home Practice Logs to turn in at the next class. Have blank logs out for participants to take.
- Collect completed Home Practice Logs and ask those that did not turn one in to fill it out before leaving, even if they were not able to practice.
- Say good-bye to all participants by name.

© Boston Medical Center
Veterans Back to Health Trial

Segment 1: Opening to Something New

Week 3

Check-in	8 minutes

- Welcome participants by name and take attendance.
- Ask participants how their practice is going, allowing them to share the successes and/or challenges they have encountered during practice.
- Ask participants to consider setting a home practice goal for the coming week.
- Discuss Awareness**:** Introduce Veterans to the idea of self-awareness and encourage them to be aware of when to make adjustments in their poses. Similarly, with awareness in life we can work toward changing old unhealthy habits and creating new health ones.
- Breathing exercise (p. 69)

Yoga Postures	55 minutes

1. Knees to Chest Pose
2. Knees Together Twist Pose
3. Pelvic Tilt Pose
4. Cat/Cow **OR** Wheel Pose
5. Mountain Pose
6. Shoulder Opener Pose
7. Crescent Moon Pose (Hands on Hips)
8. Chair Pose (Hands on Hips)
9. Warrior at Wall Pose
10. Triangle Pose (at Wall)
11. Forward Bend at Wall Pose
12. Child's Pose (in Chair)
13. Chair Dog Pose
14. Locust Pose (Legs Only)
15. Sphinx Pose
16. Bridge Pose (Supported)
17. Knees Together Twist Pose
18. Knees to Chest Pose

Relaxation Exercise	8-10 minutes

- Prepare the body for relaxation by leading into Final Resting Pose or another comfortable position.
- Yoga teachers should give clear guidance to participants throughout the relaxation exercise.
- Encouraged participants to set aside actions, thoughts, stress, worries and the events of the day and focus on the present moment in class.

Closing	2-4 minutes

- Allow participants to share thoughts or reflections if they are comfortable.
- Remind participants to practice yoga at home and to fill out Home Practice Logs to turn in at the next class. Have blank logs out for participants to take.
- Collect completed Home Practice Logs and ask those that did not turn one in to fill it out before leaving, even if they were not able to practice.
- Say good-bye to all participants by name.

© Boston Medical Center
Veterans Back to Health Trial

Segment 2: Listening to Your Back

Week 4

**Check-in**	**8 minutes**

- Welcome participants by name and take attendance.
- Ask participants to take a quick moment to think about if they have noticed any changes (positive or negative) since beginning yoga. Do not prompt for positive responses though acknowledge and validate responses without judgment.
- Discuss Acceptance: Encourage Veterans to accept themselves, their challenges, and the current moment, practicing this acceptance in each pose and guiding them towards a different relationship with pain. Encouraged them to also carry this self-acceptance into daily life and to the acceptance of other people and their challenges.
- Breathing exercise (p. 69)

**Yoga Postures**	**55 minutes**

1. Knees to Chest Pose
2. Knees Together Twist Pose
3. Big Toe Pose
4. Table Top with Leg Extended Pose
5. Mountain Pose
6. Shoulder Opener **OR** Crescent Moon Pose (One Arm Up)
7. Chair Pose (Arms Parallel to Floor)
8. Triangle Pose (Heel at Wall)
9. Warrior II Pose (Heel at Wall)
10. Wide-Leg Bend (at Wall) **OR** Chair Dog Pose
11. Child's Pose (in Chair)
12. Locust Pose (Legs **OR** Upper Body Only)
13. Sphinx **OR** Cobra Pose
14. Side Hip Strengtheners
15. Eye of the Needle Pose
16. Bridge Pose (Supported **OR** Unsupported)
17. Knees Together Twist Pose
18. Knees to Chest Pose
19. Reclining Cobbler **OR** Reclining Chest Opener Pose

**Relaxation Exercise**	**8-10 minutes**

- Prepare the body for relaxation by leading into Final Resting Pose or another comfortable position.
- Yoga teachers should give clear guidance to participants throughout the relaxation exercise.
- Encouraged participants to set aside actions, thoughts, stress, worries and the events of the day and focus on the present moment in class.

**Closing**	**2-4 minutes**

- Allow participants to share thoughts or reflections if they are comfortable.
- Remind participants to practice yoga at home and to fill out Home Practice Logs to turn in at the next class. Have blank logs out for participants to take.
- Collect completed Home Practice Logs and ask those that did not turn one in to fill it out before leaving, even if they were not able to practice.
- Say good-bye to all participants by name.

© Boston Medical Center
Veterans Back to Health Trial

Segment 2: Listening to Your Back

Week 5

**Check-in**	**8 minutes**

- Welcome participants by name and take attendance.
- Ask participants how their practice is going, allowing them to share the successes and/or challenges they have encountered during practice.
- Discuss goals for yoga practice in the coming weeks.
- Discuss Self-Control: Like achieving goals in life, staying with our practice of yoga requires practice, will power, and patience. With self-control, we can move toward doing whole poses that might be initially difficult for us.
- Breathing exercise (p. 69)

**Yoga Postures**	**55 minutes**

1. Knees to Chest Pose
2. Knees Together Twist Pose
3. Big Toe Pose
4. Table Top with Leg Extended Pose
5. Mountain Pose
6. Shoulder Opener **OR** Crescent Moon Pose (One Arm Up)
7. Chair Pose (Arms Parallel to Floor)
8. Triangle Pose (Heel at Wall)
9. Warrior II Pose (Heel at Wall)
10. Wide-Leg Bend (at Wall) **OR** Chair Dog Pose
11. Child's Pose (in Chair)
12. Locust Pose (Legs **OR** Upper Body Only)
13. Sphinx **OR** Cobra Pose
14. Side Hip Strengtheners
15. Eye of the Needle Pose
16. Bridge Pose (Supported **OR** Unsupported)
17. Knees Together Twist Pose
18. Knees to Chest Pose
19. Reclining Cobbler **OR** Reclining Chest Opener Pose

**Relaxation Exercise**	**8-10 minutes**

- Prepare the body for relaxation by leading into Final Resting Pose or another comfortable position.
- Yoga teachers should give clear guidance to participants throughout the relaxation exercise.
- Encouraged participants to set aside actions, thoughts, stress, worries and the events of the day and focus on the present moment in class.

**Closing**	**2-4 minutes**

- Allow participants to share thoughts or reflections if they are comfortable.
- Remind participants to practice at home and complete Home Practice Logs.
- Ask participants that did not turn in a Home Practice Log to fill one out before leaving, even if they were not able to practice in the past week.
- Say good-bye to all participants by name.

© Boston Medical Center
Veterans Back to Health Trial

Segment 2: Listening to Your Back

Week 6

**Check-in**	**8 minutes**

- Welcome participants by name and take attendance.
- Ask participants how their home practice is going. Acknowledge the challenges of learning to listen to their bodies and keeping up with yoga practice.
- Discuss Honesty: Encourage Veterans to be honest with themselves in their practice of yoga as well as to recognize their own limitations and accept that they may have to do poses differently than others in the class.
- Breathing exercise (p. 69)

**Yoga Postures**	**55 minutes**

1. Knees to Chest Pose
2. Knees Together Twist Pose
3. Big Toe Pose
4. Table Top with Leg Extended Pose
5. Mountain Pose
6. Shoulder Opener **OR** Crescent Moon Pose (One Arm Up)
7. Chair Pose (Arms Parallel to Floor)
8. Triangle Pose (Heel at Wall)
9. Warrior II Pose (Heel at Wall)
10. Wide-Leg Bend (at Wall) **OR** Chair Dog Pose
11. Child's Pose (in Chair)
12. Locust Pose (Legs **OR** Upper Body Only)
13. Sphinx **OR** Cobra Pose
14. Side Hip Strengtheners
15. Eye of the Needle Pose
16. Bridge Pose (Supported **OR** Unsupported)
17. Knees Together Twist Pose
18. Knees to Chest Pose
19. Reclining Cobbler **OR** Reclining Chest Opener Pose

**Relaxation Exercise**	**8-10 minutes**

- Prepare the body for relaxation by leading into Final Resting Pose or another comfortable position.
- Yoga teachers should give clear guidance to participants throughout the relaxation exercise.
- Encouraged participants to set aside actions, thoughts, stress, worries and the events of the day and focus on the present moment in class.

**Closing**	**2-4 minutes**

- Allow participants to share thoughts or reflections if they are comfortable.
- Remind participants to practice at home and complete Home Practice Logs.
- Ask participants that did not turn in a Home Practice Log to fill one out before leaving, even if they were not able to practice in the past week.
- Say good-bye to all participants by name.

© Boston Medical Center
Veterans Back to Health Trial

Segment 3: Engaging Your Power

Week 7

**Check-in**	**8 minutes**

- Welcome participants by name and take attendance.
- As participants come in, consider individual needs as the postures this week become slightly more challenging. Adapt teaching and support as necessary while discouraging competition.
- Remind participants to push themselves only as far as they are comfortable during practice. They always have the option of adjusting the poses to be more comfortable.
- Discuss Gratitude: Encourage Veterans to feel grateful for what they have right now. In yoga practice, have them focus on what they *can* do, instead of what is challenging in the moment.
- Breathing exercise (p. 69)

**Yoga Postures**	**55 minutes**

1. Knees to Chest Pose
2. Knees Together Twist Pose
3. Big Toe Pose
4. Cat/Cow **OR** Wheel Pose
5. Mountain Pose
6. Shoulder Opener **OR** Chair Pose (Arms Up)
7. Triangle Pose (Heel at Wall, if needed)
8. Warrior II **OR** Warrior I Pose (Heel at Wall)
9. Wide-Leg Bend Pose (with Chair **OR** Blocks)
10. Child's Pose (in Chair **OR** on Floor)
11. Downward Dog **OR** Chair Dog Pose
12. Plank Pose (with Chair **OR** Half)
13. Side Plank Pose (with Chair **OR** Half)
14. Side Hip Strengtheners
15. Locust Pose (Legs **OR** Upper Body Only)
16. Eye of the Needle Pose
17. Bridge Pose (Supported **OR** Unsupported)
18. Knees Together Twist Pose
19. Knees to Chest Pose
20. Reclining Cobbler **OR** Reclining Chest Opener **OR** Legs up the Wall Pose

**Relaxation Exercise**	**8-10 minutes**

- Prepare the body for relaxation by leading into Final Resting Pose or another comfortable position.
- Yoga teachers should give clear guidance to participants throughout the relaxation exercise.
- Encouraged participants to set aside actions, thoughts, stress, worries and the events of the day and focus on the present moment in class.

**Closing**	**2-4 minutes**

- Allow participants to share thoughts or reflections if they are comfortable.
- Remind participants to practice at home and complete Home Practice Logs.
- Ask participants that did not turn in a Home Practice Log to fill one out before leaving, even if they were not able to practice in the past week.
- Remind participants to allow their bodies to lead them to the postures that feel good to their bodies during home practice.
- Say good-bye to all participants by name.

© Boston Medical Center
Veterans Back to Health Trial

Segment 3: Engaging Your Power
Week 8

**Check-in**	**8 minutes**

- Welcome participants by name and take attendance.
- Ask participants to reflect on their success and/or challenges with following their bodies and maintaining their breath through the yoga poses. Allow them to share if they feel comfortable.
- Discuss Balance: Discuss the concepts of effort and ease in yoga postures. Teach Veterans how to balance effort and ease in their yoga practice and encouraged them to seek similar balance in their daily lives.
- Breathing exercise (p. 69)

**Yoga Postures**	**55 minutes**

1. Knees to Chest Pose
2. Knees Together Twist Pose
3. Big Toe Pose
4. Cat/Cow **OR** Wheel Pose
5. Mountain Pose
6. Shoulder Opener **OR** Chair Pose (Arms Up)
7. Triangle Pose (Heel at Wall, if needed)
8. Warrior II **OR** Warrior I Pose (Heel at Wall)
9. Wide-Leg Bend Pose (with Chair **OR** Blocks)
10. Child's Pose (in Chair **OR** on Floor)
11. Downward Dog **OR** Chair Dog Pose
12. Plank Pose (with Chair **OR** Half)
13. Side Plank Pose (with Chair **OR** Half)
14. Side Hip Strengtheners
15. Locust Pose (Legs **OR** Upper Body Only)
16. Eye of the Needle Pose
17. Bridge Pose (Supported **OR** Unsupported)
18. Knees Together Twist Pose
19. Knees to Chest Pose
20. Reclining Cobbler **OR** Reclining Chest Opener **OR** Legs up the Wall Pose

**Relaxation Exercise**	**8-10 minutes**

- Prepare the body for relaxation by leading into Final Resting Pose or another comfortable position.
- Yoga teachers should give clear guidance to participants throughout the relaxation exercise.
- Encouraged participants to set aside actions, thoughts, stress, worries and the events of the day and focus on the present moment in class.

**Closing**	**2-4 minutes**

- Allow participants to share thoughts or reflections if they are comfortable.
- Remind participants to practice at home and complete Home Practice Logs.
- Ask participants that did not turn in a Home Practice Log to fill one out before leaving, even if they were not able to practice in the past week.
- Say good-bye to all participants by name.

© Boston Medical Center
Veterans Back to Health Trial

Segment 3: Engaging Your Power

Week 9

Check-in	8 minutes

- Welcome participants by name and take attendance.
- Query participants about how they feel about their progress with yoga and their back pain.
- Discuss <u>Moderation</u>: Encourage Veterans to be in control of their desires and to seek balance in their lives. In yoga practice, it is better to practice a little bit of yoga frequently than to practice a lot of yoga every once in a while.
- Breathing exercise (p. 69)

Yoga Postures	55 minutes

1. Knees to Chest Pose
2. Knees Together Twist Pose
3. Big Toe Pose
4. Cat/Cow **OR** Wheel Pose
5. Mountain Pose
6. Shoulder Opener **OR** Chair Pose (Arms Up)
7. Triangle Pose (Heel at Wall, if needed)
8. Warrior II **OR** Warrior I Pose (Heel at Wall)
9. Wide-Leg Bend Pose (with Chair **OR** Blocks)
10. Child's Pose (in Chair **OR** on Floor)
11. Downward Dog **OR** Chair Dog Pose
12. Plank Pose (with Chair **OR** Half)
13. Side Plank Pose (with Chair **OR** Half)
14. Side Hip Strengtheners
15. Locust Pose (Legs **OR** Upper Body Only)
16. Eye of the Needle Pose
17. Bridge Pose (Supported **OR** Unsupported)
18. Knees Together Twist Pose
19. Knees to Chest Pose
20. Reclining Cobbler **OR** Reclining Chest Opener **OR** Legs up the Wall Pose

Relaxation Exercise	8-10 minutes

- Prepare the body for relaxation by leading into Final Resting Pose or another comfortable position.
- Yoga teachers should give clear guidance to participants throughout the relaxation exercise.
- Encouraged participants to set aside actions, thoughts, stress, worries and the events of the day and focus on the present moment in class.

Closing	2-4 minutes

- Allow participants to share thoughts or reflections if they are comfortable.
- Remind participants to warm up at home before doing more energetic postures.
- Remind participants to practice at home and complete Home Practice Logs.
- Ask participants that did not turn in a Home Practice Log to fill one out before leaving, even if they were not able to practice in the past week.
- Say good-bye to all participants <u>by name</u>.

© Boston Medical Center
Veterans Back to Health Trial

Segment 4: Bringing it Home

Week 10

**Check-in**	**8 minutes**

- Welcome participants by name and take attendance.
- Ask participants how their yoga practice is going and how their backs feel.
- Discuss Healthy Lifestyle: Teach the Veterans how to become aware of the healthy lifestyle elements of yoga. Encourage them to incorporate healthy habits into their lives outside of yoga (e.g., healthy food, healthy relationships, and clean environment).
- Breathing exercise (p. 69)

**Yoga Postures**	**55 minutes**

1. Knees to Chest Pose
2. Knees Together Twist Pose
3. Big Toe Pose
4. Table Top with Leg Extended Pose
5. Child's Pose
6. Downward Dog Pose
7. Plank Pose (Half, then Full)
8. Side Plank Pose (Half, then Full)
9. Cobra Pose
10. Side Hip Strengtheners
11. Mountain Pose
12. Shoulder Opener **OR** Crescent Moon Pose
13. Triangle Pose
14. Warrior II **OR** Warrior I Pose
15. Baby Dancer Pose
16. Forward Bend Pose
17. Eye of the Needle Pose
18. Bridge Pose (Supported **OR** Unsupported)
19. Knees Together Twist Pose
20. Knees to Chest Pose
21. Reclining Cobbler **OR** Reclining Chest Opener **OR** Legs up the Wall Pose

**Relaxation Exercise**	**8-10 minutes**

- Prepare the body for relaxation by leading into Final Resting Pose or another comfortable position.
- Yoga teachers should give clear guidance to participants throughout the relaxation exercise.
- Encouraged participants to set aside actions, thoughts, stress, worries and the events of the day and focus on the present moment in class.

**Closing**	**2-4 minutes**

- Allow participants to share thoughts or reflections if they are comfortable.
- Encourage participants to take any feelings of ease and calm they find in class into other areas of their lives.
- Remind participants to practice at home and complete Home Practice Logs.
- Ask participants that did not turn in a Home Practice Log to fill one out before leaving, even if they were not able to practice in the past week.
- Say good-bye to all participants by name.

© Boston Medical Center
Veterans Back to Health Trial

Segment 4: Bringing it Home

Week 11

Check-in **8 minutes**

- Welcome participants by name and take attendance.
- Ask participants if they have any questions about yoga and/or yoga practice.
- Discuss Compassion for Others: Encourage Veterans to practice being the person they would like to be (e.g. compassion not judgment, friendliness instead of anger, joy for others instead of jealousy).
- Breathing exercise (p. 69)

Yoga Postures **55 minutes**

1. Knees to Chest Pose
2. Knees Together Twist Pose
3. Big Toe Pose
4. Table Top with Leg Extended Pose
5. Child's Pose
6. Downward Dog Pose
7. Plank Pose (Half, then Full)
8. Side Plank Pose (Half, then Full)
9. Cobra Pose
10. Side Hip Strengtheners
11. Mountain Pose
12. Shoulder Opener **OR** Crescent Moon Pose
13. Triangle Pose
14. Warrior II **OR** Warrior I Pose
15. Baby Dancer Pose
16. Forward Bend Pose
17. Eye of the Needle Pose
18. Bridge Pose (Supported **OR** Unsupported)
19. Knees Together Twist Pose
20. Knees to Chest Pose
21. Reclining Cobbler **OR** Reclining Chest Opener **OR** Legs up the Wall Pose

Relaxation Exercise **8-10 minutes**

- Prepare the body for relaxation by leading into Final Resting Pose or another comfortable position.
- Yoga teachers should give clear guidance to participants throughout the relaxation exercise.
- Encouraged participants to set aside actions, thoughts, stress, worries and the events of the day and focus on the present moment in class.

Closing **2-4 minutes**

- Allow participants to share thoughts or reflections if they are comfortable.
- Remind participants to practice at home and complete Home Practice Logs.
- Ask participants that did not turn in a Home Practice Log to fill one out before leaving, even if they were not able to practice in the past week.
- Say good-bye to all participants by name.

© Boston Medical Center
Veterans Back to Health Trial

Segment 4: Bringing it Home

Week 12

Check-in	8 minutes

- Welcome participants by name and take attendance.
- Ask participants how their home practice is going. Acknowledge the challenges of learning to listen to their bodies and keeping up with yoga practice.
- Discuss Community: Encourage Veterans to look to each other for support in their practice. This gives a sense of common ground and connection not only within ourselves but with all people.
- Breathing exercise (p. 69)

Yoga Postures	55 minutes

1. Knees to Chest Pose
2. Knees Together Twist Pose
3. Big Toe Pose
4. Table Top with Leg Extended Pose
5. Child's Pose
6. Downward Dog Pose
7. Plank Pose (Half, then Full)
8. Side Plank Pose (Half, then Full)
9. Cobra Pose
10. Side Hip Strengtheners
11. Mountain Pose
12. Shoulder Opener **OR** Crescent Moon Pose
13. Triangle Pose
14. Warrior II **OR** Warrior I Pose
15. Baby Dancer Pose
16. Forward Bend Pose
17. Eye of the Needle Pose
18. Bridge Pose (Supported **OR** Unsupported)
19. Knees Together Twist Pose
20. Knees to Chest Pose
21. Reclining Cobbler **OR** Reclining Chest Opener **OR** Legs up the Wall Pose

Relaxation Exercise	8-10 minutes

- Prepare the body for relaxation by leading into Final Resting Pose or another comfortable position.
- Yoga teachers should give clear guidance to participants throughout the relaxation exercise.
- Encouraged participants to set aside actions, thoughts, stress, worries and the events of the day and focus on the present moment in class.

Closing	2-4 minutes

- Ask participants to share what yoga has done for them and what they are taking home from the lessons, if they are comfortable.
- Ask participants that did not turn in a Home Practice Log to fill one out before leaving, even if they were not able to practice in the past week.
- Say good-bye to all participants by name and encourage them to continue yoga practice on their own. Remind them of how much they have learned and the tools they have gained to manage pain and other concerns. **Also remind them that the study is not over for another three months.** You can mention that study staff will follow-up with them periodically and that they can call staff at any time.

© Boston Medical Center
Veterans Back to Health Trial

Guidelines for Teaching Specific Yoga Poses

Baby Dancer Pose 37
Big Toe Pose 38
Bridge Pose 39
Cat/Cow Pose 40
Chair Pose 41
Chair Dog Pose 42
Child's Pose 43
Cobra Pose 44
Crescent Moon Pose 45
Downward Dog Pose 46
Eye of the Needle Pose 47
Forward Bend Pose 48
Knees to Chest Pose 49
Knees Together Twist Pose 50
Legs up the Wall Pose 51
Locust Pose 52
Mountain Pose 53
Pelvic Tilt Pose 54
Plank Pose 55
Reclining Chest Opener Pose 56
Reclining Cobbler Pose 57
Shoulder Opener Pose 58
Side Hip Strengtheners 59
Side Plank Pose 60
Sphinx Pose 61
Table Top Pose 62
Triangle Pose 63
Warrior I Pose 64
Warrior II Pose 65
Warrior at Wall Pose 66
Wheel Pose 67
Wide-Leg Bend Pose 68

© Boston Medical Center
Veterans Back to Health Trial

Guidelines for Teaching Specific Yoga Poses

Baby Dancer Pose

Description: Standing quadriceps (front-thigh muscles) and hip flexor stretch that helps release tension in the lower back and hips.

How to teach: Stand with the left side of your body against the wall and your left hand on the wall (or on the back of a chair). Find a steady point in front of you to focus on to help you balance. Gradually shift your weight to the left leg. Inhale and bend right knee so the right foot is behind you. Hold the right foot or ankle with the right hand or belt. Stand firmly on the left leg, keeping it straight. Take a moment to balance yourself so that knees are in alignment with your hips. Hold here and breathe into the stretch lifting from your abdominal muscles, stretching down the front of the thigh, and tucking the tailbone. Slowly release bent leg to the floor. Come back to Mountain Pose. Repeat on opposite side.

Modifications:

1. If you have difficulty reaching back far enough to hold your foot due to tight shoulders or quads, wrap a strap around the right foot and hold the other end of the strap with the right hand. If the strap is long enough, you can put it over your shoulder so that when you pull it down in front of you, your leg comes up further.
2. Participants can transition to removing their hand from the wall but still standing close to the wall in case they need to lean on it for support.
3. Sitting in Chair: Sit on edge of chair, take one foot back to the side of the chair and let the knee drop down to face the floor. If able, hold ankle or place a belt around ankle.

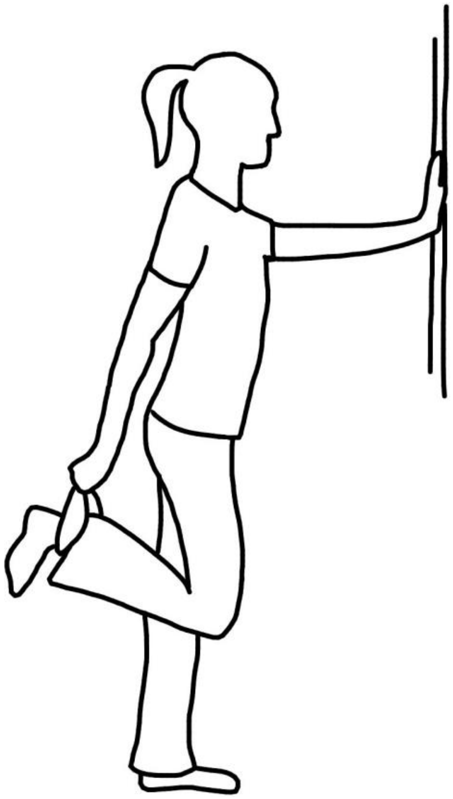

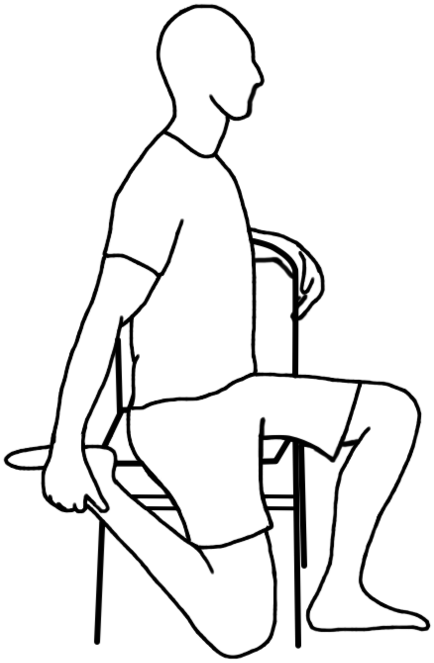

Baby Dancer in Chair

© Boston Medical Center
Veterans Back to Health Trial

Guidelines for Teaching Specific Yoga Poses

Big Toe Pose

Description: Supine pose to stretch hips (psoas muscles), thighs, and hamstrings.

How to teach: Lie supine (on the back) on the floor with knees bent and soles of feet flat on the mat. A folded blanket can be placed under the head for support. Place belt on sole of right foot and straighten leg. Allow shoulders to relax down toward floor and keep tailbone on floor. Press right foot up overhead while pulling arms and shoulders down. Inhale, straighten leg. Take belt in right hand, bring left hand to floor. Exhale, move right leg out to right side while keeping opposite hip on floor and let left knee move out to the left. Inhale, bring leg back up straight. Repeat, moving leg out and up again three times, keeping leg straight and hips on the floor, moving with each breath. End with leg straight up. Bend knee and take belt off and repeat on left leg.

Modifications:

1. Bend and straighten leg several times using a belt for support.
2. With leg straightened, circle leg in one direction and then in the other direction several times using a belt for support.
3. Lying on back at wall with feet facing wall, knees bent, place one leg up on wall as straight as possible using the wall for support.
4. Lying on back with feet facing chair seat, knees bent, rest right foot on edge of chair seat. Raise right leg up as straight as possible. Repeat on other side using the chair for support.
5. Sitting in Chair: Sit in a chair, with legs bent and feet on floor, place one leg straight up onto block or another chair seat (or whatever height will give a comfortable stretch depending on ability).

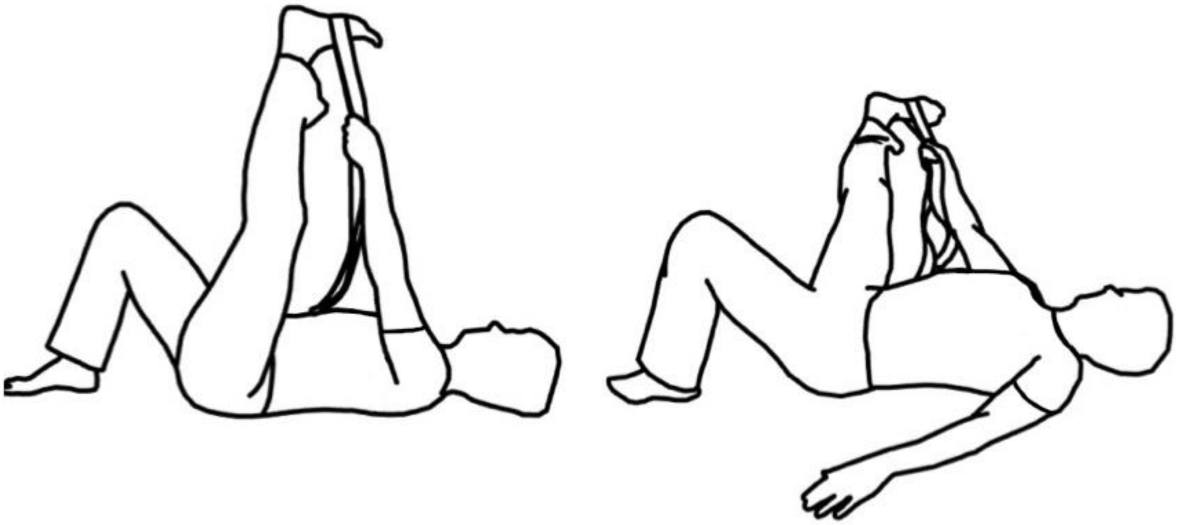

© Boston Medical Center
Veterans Back to Health Trial

Guidelines for Teaching Specific Yoga Poses

Bridge Pose

Description: Mild inversion pose that opens the chest and shoulders as well as stretches the spine, back of neck, thighs, and hip flexors (psoas muscles).

How to teach: Lying on back with knees bent, feet flat on mat and hip distance apart, walk feet in toward hips as far as possible. Keep thighs and feet parallel and do not let knees splay out. Reach hands toward ankles. Turn upper arms and shoulders down toward floor and tuck shoulders in toward each other. Inhale, press feet into the floor, lifting tailbone up until back is up off floor. Press arms down into floor and lift upper back up, bring chest toward chin. Exhale, relax neck, drawing chin to the chest to keep your head straight and not looking to the sides. Slowly roll down back to the mat starting with the upper back, one vertebra at a time. Keep tailbone lifted while rolling down until body is all the way down. Rest back on the floor.

If the pose causes back pain, keep back on floor and do gentle pelvic tilts (p. 54).

Modifications:

1. If discomfort in knees, walk feet further away from hips.
2. Place a block between knees or a belt around thighs to keep thighs parallel.
3. Sitting in Chair: With the hands on chair seat or holding the arms of the chair, lean back and lift the hips up off the chair, making sure not to lift from the lower back but instead lift from backs of thighs. If you cannot put weight on hands or arms, do Pelvic Tilt in chair (p. 54).

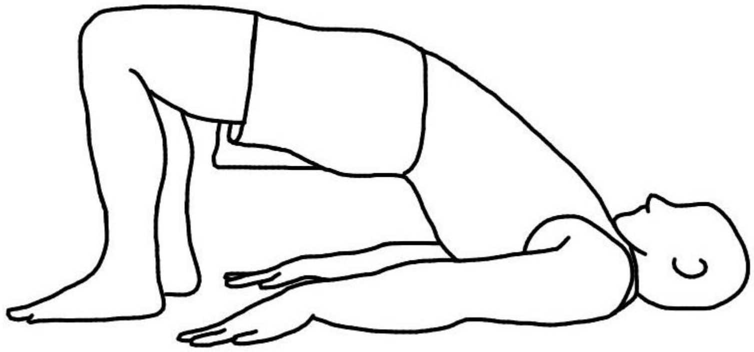

Bridge

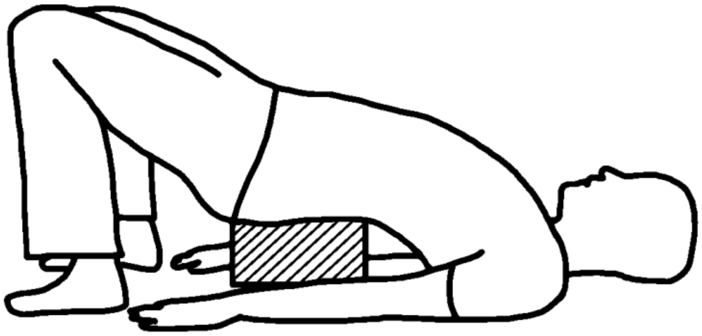

Supported Bridge

© Boston Medical Center
Veterans Back to Health Trial

Guidelines for Teaching Specific Yoga Poses

Cat/Cow Pose

Description: Gentle flow of two poses that brings flexibility to the spine, stretches the back, and opens the chest, while encouraging the breath to be slow and coordinated with movements.

How to teach: To do cat posture, begin on all fours. Place hands under shoulders and hips over knees. Press hands into mat. Exhale, tuck chin toward chest, tuck tailbone down toward floor, and round spine up, lifting abdomen up toward spine. To move into cow posture, inhale and lift top of the head, keeping back of neck lengthened. Drop the back down toward mat, lifting tailbone. Keep abdomen lifted up into body to prevent overarching in lower back. Slowly flow between cat and cow posture, exhaling and inhaling respectively.

To prevent injury, caution participants to not overextend the neck. Encourage them to keep the spine of the neck in line with the spine of the upper back and to look forward (not up) or slightly down.

Modifications:

1. If flat hands cause wrist discomfort, make a fist with both hands so that weight rests on knuckles and thumbs face down.
2. If participants need more cushioning under knees or hands, roll the sides of the mat under knees or the top of mat under hands or use a blanket to pad the mat.
3. The above can be done standing behind a chair with hands on back of chair or in front of chair with hands on seat.
4. Sitting in Chair: Sit on chair with feet under knees and knees hip-width apart. Exhale, gently round the spine and tuck the chin toward the chest. Inhale and straighten up. Exhale, gently lift head and chest up, slightly arching the back. Repeat several times. Rest, sitting straight up.

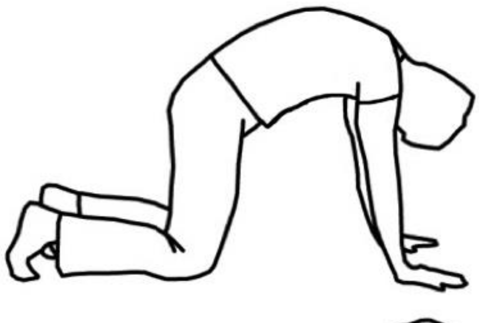

Cat

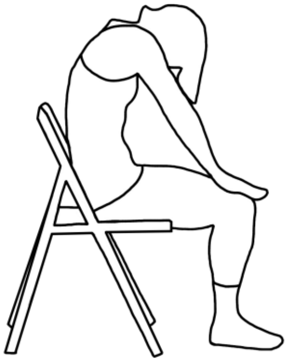

Cat in Chair

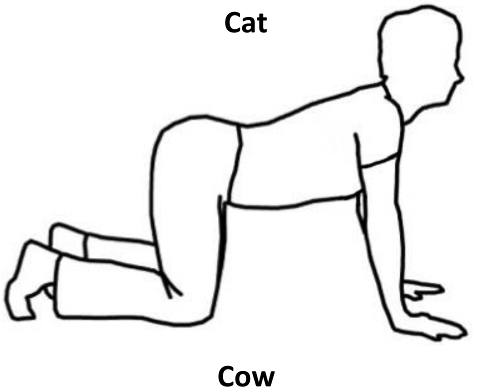

Cow

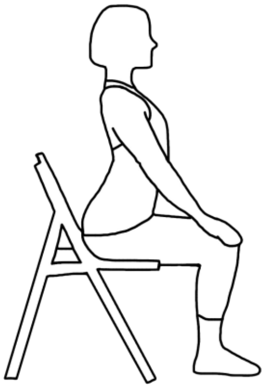

Cow in Chair

© Boston Medical Center
Veterans Back to Health Trial

Guidelines for Teaching Specific Yoga Poses

Chair Pose

Description: Standing pose that strengthens the thighs and ankles, while toning the shoulders, buttocks, hips, and back.

How to teach: From Mountain Pose, place hands on hips. Exhale, bend knees over feet, keeping them pointed forward, while also bending at hips as if about to sit in a chair. Drop tailbone down toward the floor while lifting chest up. Move weight back into heels and draw abdomen in. In Segments 2 (weeks 4-6), participants can inhale and extend arms in front parallel to the floor. In Segment 3 (weeks 7-9), participants may be ready to try extending arms straight above the head. If this causes you to lose your balance, keep your hands on your hips. Exhale, move legs and hips back slightly to bring weight back on heels. Be careful not to hyperextend the lower back and keep the tailbone tucked. Inhale, straighten legs and bring arms down to come back to Mountain Pose.

Modifications:

1. Place block between knees or thighs to keep knees pointing forward.
2. Stand with back on wall but feet and knees away from wall so that back is leaning on the wall with feet and knees together (unless there is knee pain or balance issues, then separate). Bend knees, making sure that knees are pointing forward and press lower back into wall. Slowly raise arms up overhead.
3. Stand facing a wall with feet hip-width apart and arm-length away from the wall. Place your fingertips on the wall at shoulder height. Follow directions above for teaching pose.
4. Sitting in Chair: Sit with feet under knees. Lean slightly forward, taking body weight onto feet. While remaining seated, raise arms over head, keeping chest and back ribs lifted and tailbone moving down toward chair seat.

Hands on Hips

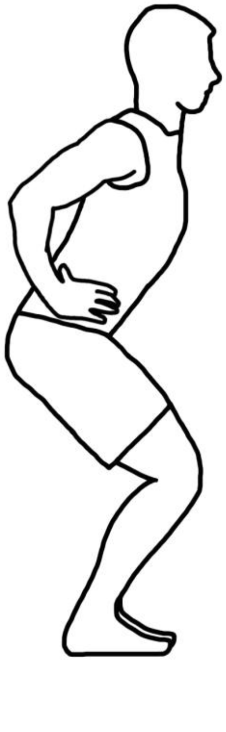

Hands Parallel to Floor

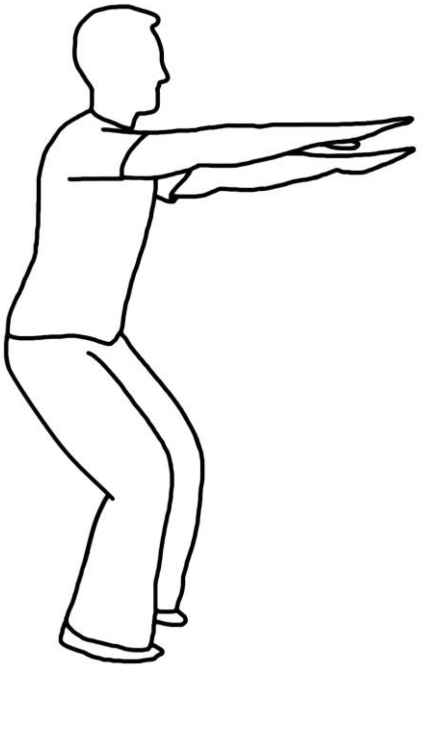

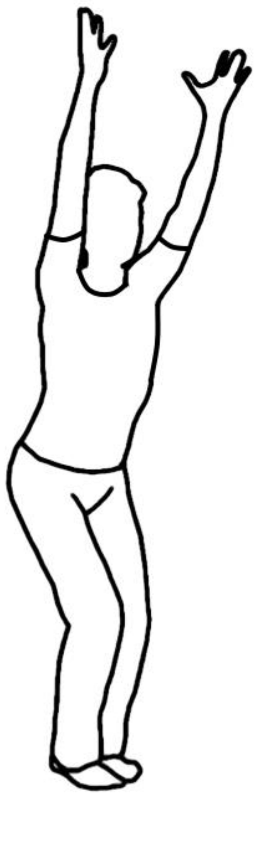

© Boston Medical Center
Veterans Back to Health Trial

Chair Dog Pose

Description: Standing pose with less inversion than Downward Dog that still stretches the hamstrings, shoulders, calves, hands, and spine.

How to Teach: Place a chair on top of the mat so that the back of the chair is against a wall. Stand facing the chair with feet hip-width apart. Bend forward from hips, placing hands on the seat of the chair. Stand far enough from the chair so that the legs angle back with the arms, legs and back are all straight. Press down through the feet and pull up through the thighs. Pull thighs and hips back away from the chair while gently pressing hands into the chair. Keep head in line with the arms.

Modifications:

1. If there is a history of shoulder injury, modify straight arms for soft or bent elbows.
2. With chair facing wall, place hands on back of chair for less inversion.
3. To do this pose with your hands on a wall, stand facing the wall with feet hip-width apart. Bend forward from hips, placing hands on the wall and walk hands up the wall slightly higher than shoulder height. Stand far enough from the wall so that the legs angle back with the arms, legs and back all straight. Press down through the feet and pull up through the thighs. Pull thighs and hips back away from the wall while gently pressing hands into the wall. Keep head in line with the arms.
4. Sitting in Chair: see Modification #4 for Downward Dog (p. 46).

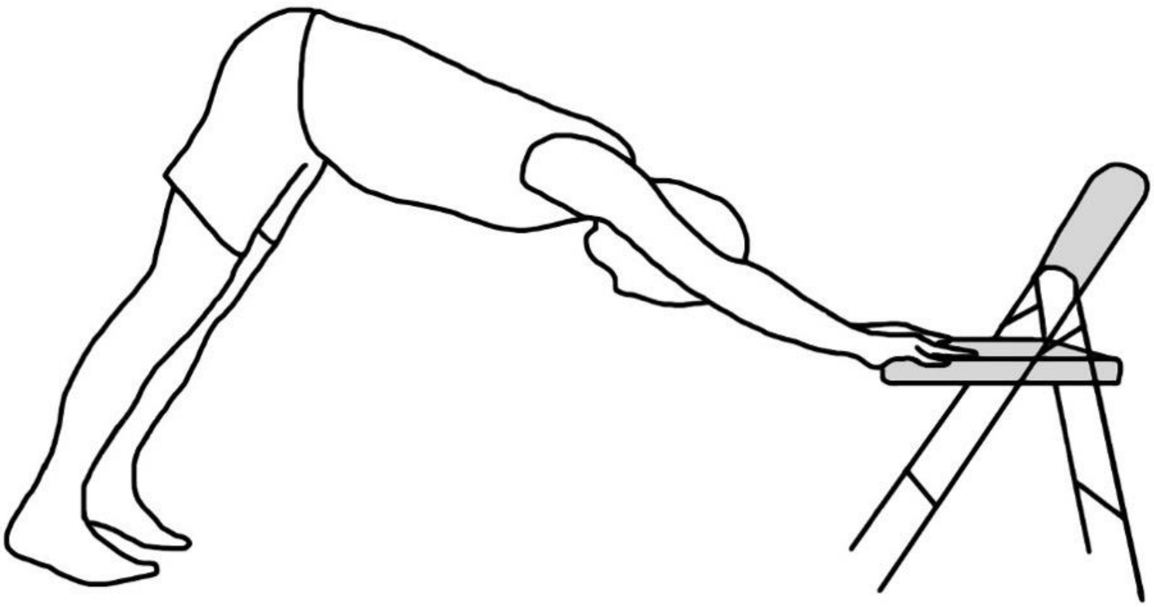

© Boston Medical Center
Veterans Back to Health Trial

Guidelines for Teaching Specific Yoga Poses

Child's Pose

Description: Resting pose to stretch the hips, thighs, and ankles while gently relaxing the muscles on the front of the body and passively stretching the muscles of the back torso.

How to teach: Start by kneeling, then sit comfortably on heels. Take knees apart while keeping toes together. Bend forward bringing head to floor. Extend arms forward on the floor overhead or to the sides reaching towards the ankles with palms facing up.

Modifications:
1. If there is knee discomfort, place a folded blanket behind knees between shin and thigh, or place a folded blanket under the knees for additional padding.
2. If there is ankle discomfort, place a rolled blanket under ankles.
3. If head does not come to floor, place a blanket under head.
4. If the feet cramp, keep toes curled under.
5. Sitting in Chair: Place feet firmly on floor. Take knees apart, aligning feet with knees. With hands on thighs, palms down, slowly bend forward between legs. Hands can stay on thighs or move to the floor or blocks. Another chair can be placed in front to allow head to rest on back or seat of chair.

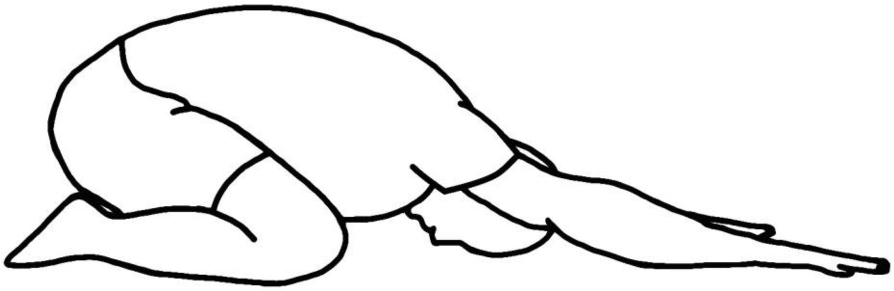

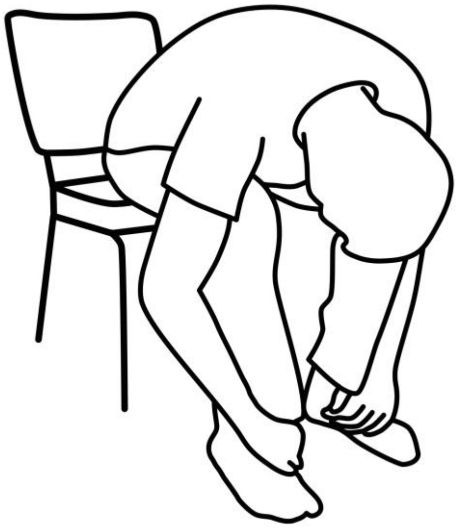

Child's Pose in Chair

© Boston Medical Center
Veterans Back to Health Trial

Guidelines for Teaching Specific Yoga Poses

Cobra Pose

<u>Description</u>: Beginning backbend that stretches the chest, increases flexibility of spine, and strengthens the spine and shoulders

<u>How to teach</u>: Lie flat on stomach, with legs slightly wider than hip distance apart. Place tops of feet on the floor and keep toes pressed into floor throughout pose. Relax shoulders and put forehead on the mat. Place palms face down at armpit level, with fingers facing forward. Keep elbows bent at side. Inhale, push palms down against the floor and use back muscles to lift head and then chest, slowly, gazing forward. Minimize weight on hands or wrists by engaging back muscles. Press pelvic bones into the floor. Move the shoulders and shoulder blades down away from neck. Keep elbows bent. There should be no strain on the lower back. Exhale, as you lower the upper back and head down to return to beginning position.

To prevent injury, caution participants to not overextend the neck. Encourage them to keep the spine of the neck in line with the spine of the upper back and to look forward (not up) or slightly down.

<u>Modifications</u>:

1. <u>Sitting in Chair</u>: Place feet firmly on floor with hands on thighs and move shoulders down away from ears. Roll upper arms back to open chest. Squeeze shoulder blades slightly into each other and pull abdomen in toward spine.
2. If there is discomfort in the wrists, do Sphinx Pose instead (p. 61).

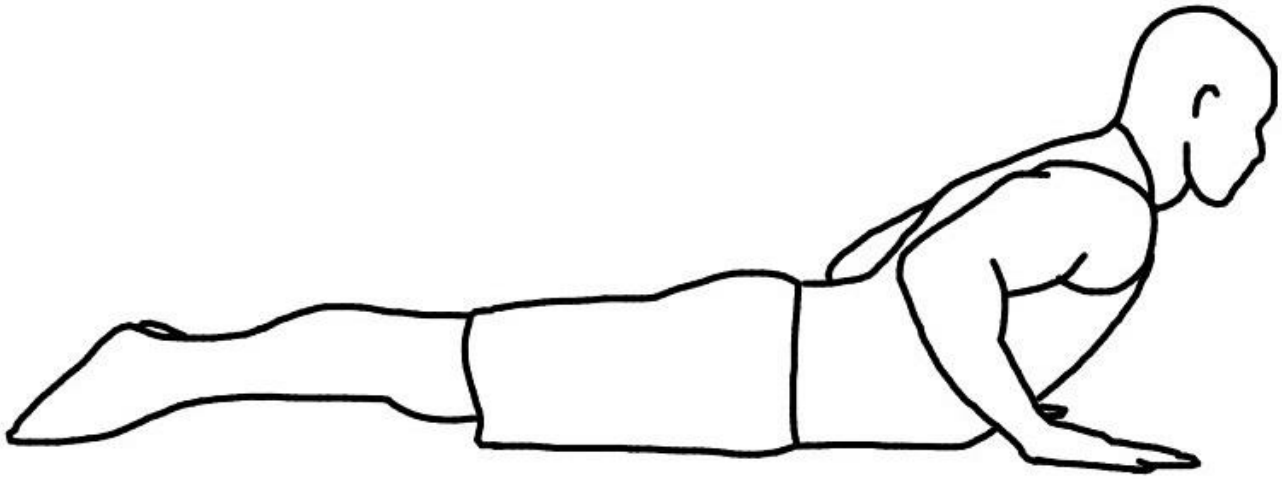

© Boston Medical Center
Veterans Back to Health Trial

Guidelines for Teaching Specific Yoga Poses

Crescent Moon Pose

Description: Standing pose that stretches the torso (intercostal muscles between the ribs), opens up the spine and shoulders, improves posture, and helps prepare the spine for deeper stretches.

How to teach: From Mountain Pose, separate the feet wider than the hips, inhale and extend arms up straight up overhead. While bending to the right, take left arm up and overhead toward the right with the palm facing the floor, keeping the right hand on the waist. Repeat on the left side.

Modifications:

1. If there is shoulder discomfort, keep both hands on the waist while bending sideways.
2. Hold the left wrist with the right hand. Exhale, bend slowly to the right, extending the left arm up and over to the right. Press down through the left foot to lengthen through the entire left side of the body. Inhale and come back up to Mountain. Hold the right wrist with the left hand and exhale while bending to the left.
3. Sitting in Chair: Sit upright with back straight, inhale and extend arms straight up overhead. Hold the left wrist with the right hand. Exhale, bend slowly to the right extending the left arm up and over to the right. Inhale, come back up. Hold the right wrist with the left hand and exhale while bending to the left.

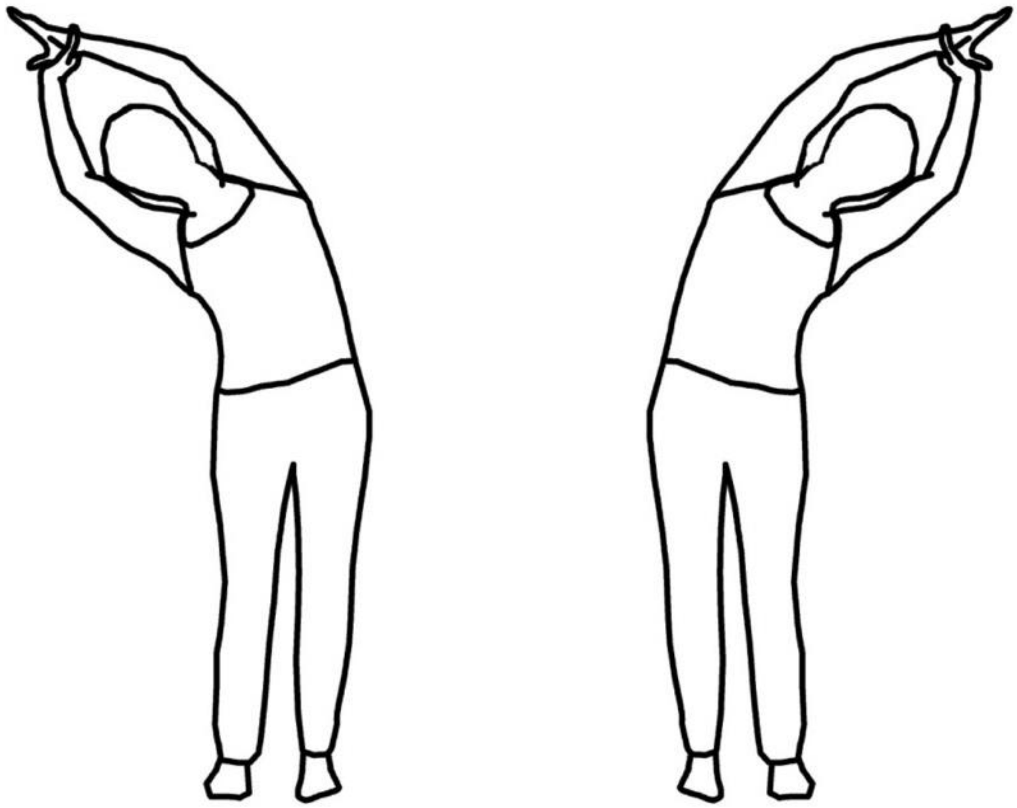

© Boston Medical Center
Veterans Back to Health Trial

Guidelines for Teaching Specific Yoga Poses

Downward Dog Pose

Description: Standing pose and mild inversion that builds strength while stretching the hamstrings, shoulders, calves, hands, and spine.

How to teach: Start on hands and knees and walk hands in front of shoulders with hands shoulder-distance apart. With knees in line with palms, move knees backwards. Inhale, lift hips up toward ceiling and straighten legs, pressing the front of the legs back. Spread fingers and press them evenly on the floor. Exhale, stretch arms keeping elbows straight, lengthening arms, torso, and back toward legs. With legs straight, inhale, lift hips higher while pressing heels down toward the floor. Your upper arms should be alongside your ears. Exhale, come back to Table Top on your hands and knees.

Modifications:
1. Chair Dog (p. 42) with hands on back or seat of chair.
2. If keeping legs straight causes back pain, bend knees to help release tension.
3. If hands on the mat are slippery, put thumb and index fingers against a wall with palms turned out and slightly wider than shoulders.
4. Sitting in Chair: Sit on edge of a chair, extend both legs out, hip-width apart and raise both arms straight up over head, shoulder-distance apart.

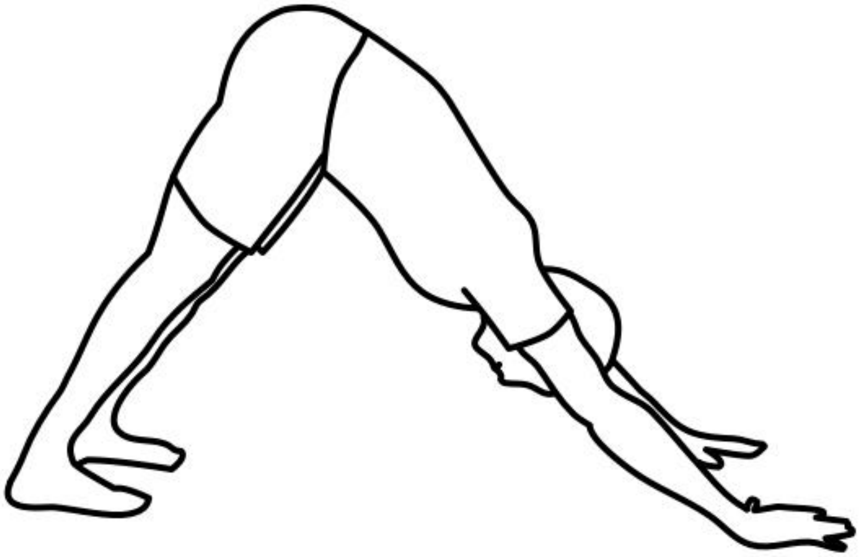

© Boston Medical Center
Veterans Back to Health Trial

Guidelines for Teaching Specific Yoga Poses

Eye of the Needle Pose

Description: Supine pose that stretches the outer hips (piriformis muscles) and low back

How to Teach: Start by lying on back with both feet on the floor. Place the right ankle on the left knee and flex the right foot. For some participants, this may be enough of a stretch to start with. If there is no pain in the hip in this starting position, with your right hand, gently push the right thigh, just below the knee, away from your head. Keep your hips, spine, and head on the floor and relax your neck. For a deeper stretch, clasp your hands behind your left hamstring and hug it in toward your torso, with your head still on the ground. Hold for a minute, and repeat on the other side.

Modifications:

1. Sitting in Chair: Place the right ankle on the left knee. If it does not cause discomfort, lean forward for stretch while keeping back and neck straight. Repeat with left ankle on right knee. The chair version of this pose usually offers a more difficult stretch than on the mat.

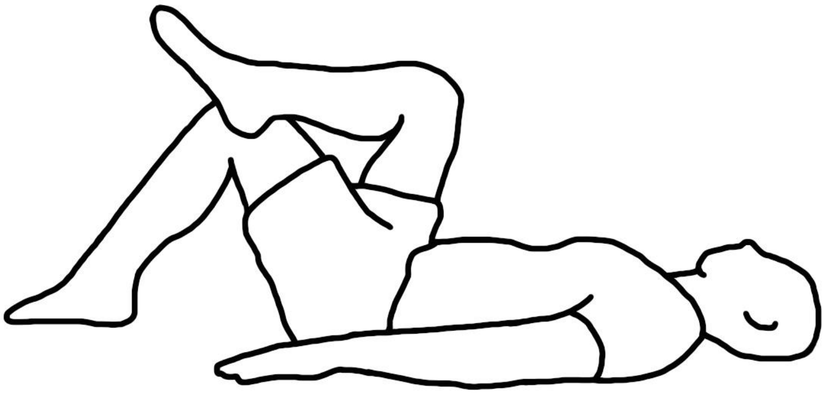

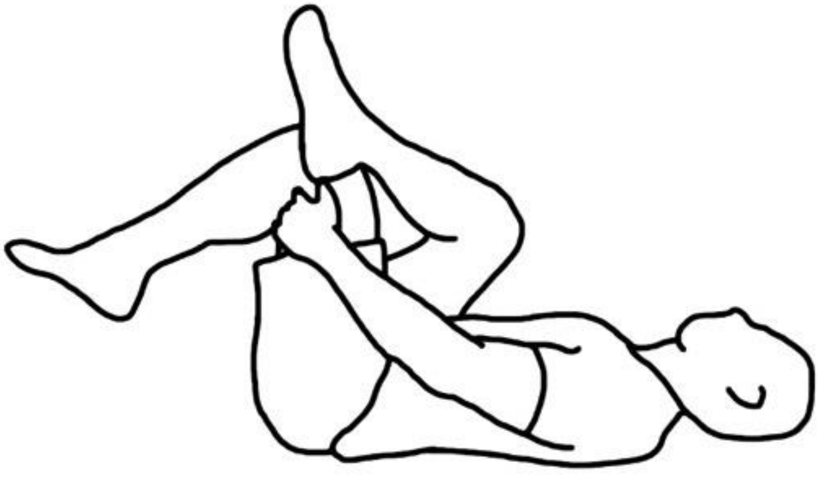

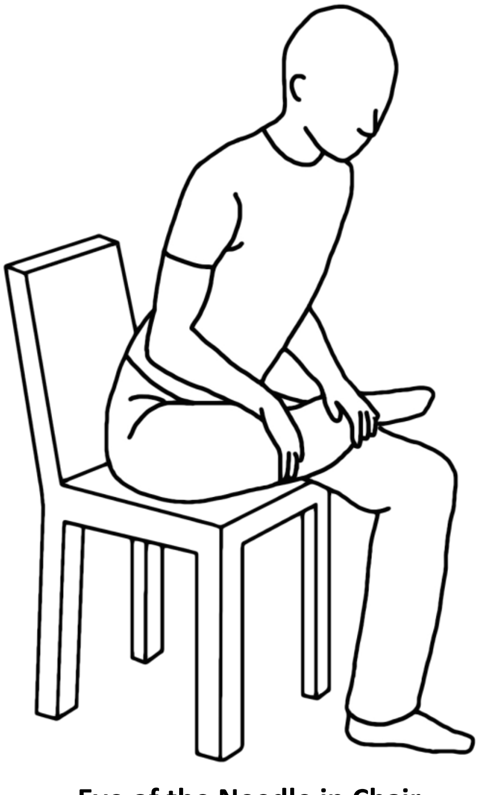

Eye of the Needle in Chair

© Boston Medical Center
Veterans Back to Health Trial

Guidelines for Teaching Specific Yoga Poses

Forward Bend Pose

Description: Standing pose to stretch the back and legs (hamstrings).

How to Teach: From Mountain Pose, stand facing the wall with feet hip-width apart. Step one foot forward and step the other foot back away from the wall with back foot slightly turned out in a wide stance. With the hands on the wall at shoulder height or slightly above, bend forward from the hips, keeping the back straight with the back of the head staying in line with the tailbone. Most participants may not be able to bend forward to be parallel with the floor, so encourage them not to push themselves too far. Keeping the weight balanced evenly in the feet and the legs straight and perpendicular to the floor, straighten the arms and press the hands down to lift the chest up, keeping the back of the neck lengthened. Try to keep the hips level side to side. Move the shoulders back toward the waist and the shoulder blades down into the body to feel the back muscles contracting and the front body lengthening.

In forward bends, the angle between their hip and torso should never be below ~70°.

Modifications:
1. Place the hands on the back of a chair, a chair seat, or on blocks.
2. Use two blocks on either side of front leg to lessen pressure on low back and increase balance. Use this as an alternative to bending the knee or in addition to the bent knee.
3. **With Heel at Wall:** Stand with right side facing the wall, feet wider than hip-width apart and right hand on wall. Turn the left foot to point away from the wall and turn right foot slightly inward, keeping the heel at the wall. Point hips directly away from the wall. Exhale, bend forward from the hips, pressing right heel into wall gently. Keep hands on hips or bring hands down to chair or blocks.
4. Sitting in Chair: Extend one leg forward with the heel on the floor and the other leg bent with the foot on the floor. Exhale, bend forward at the hips until there is a feeling of a stretch in the back of the leg. Inhale sit back up. Repeat and hold for 3 breaths. Repeat on other side.

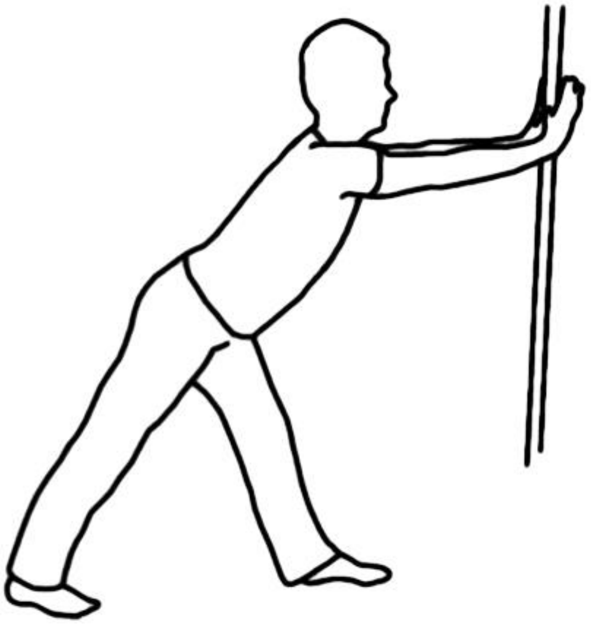

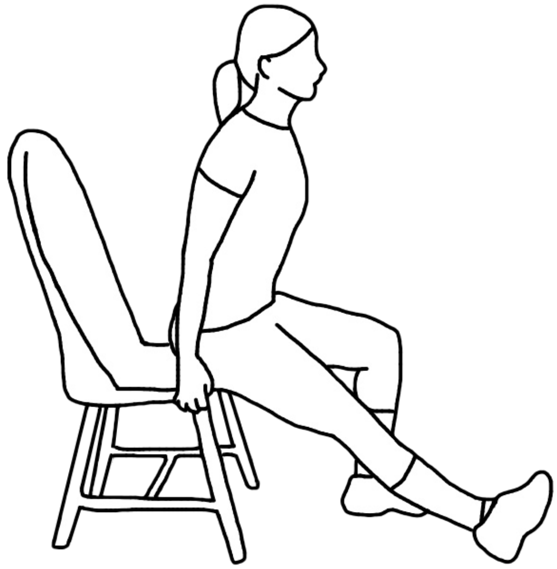

Forward Bend in Chair

© Boston Medical Center
Veterans Back to Health Trial

Guidelines for Teaching Specific Yoga Poses

Knees to Chest Pose

Description: Supine pose that releases tension in the belly area (psoas muscles) and low back.

How to teach: Start by lying on back with both feet on the floor. Inhale, lift one leg up to bring knee into chest. Hold leg around shin or thigh and on an exhale, gently bring knee in toward chest. Repeat twice on each side. Then, bring both knees into chest.

To do this pose at the end of practice, bring both knees into chest and hold while breathing.

Modifications:

1. Place a belt around shin or thigh if unable to reach with hands.
2. If unable to bring knees to chest with hands or belt, place feet on a wall or chair.
3. Sitting in Chair: Raise one knee at a time, holding leg up with hands under thigh or belt.

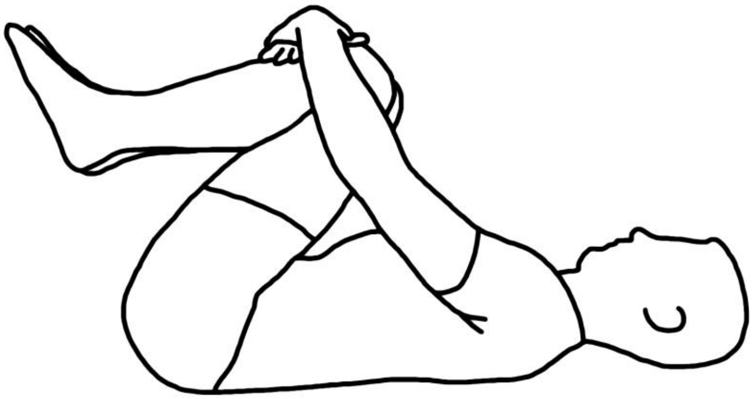

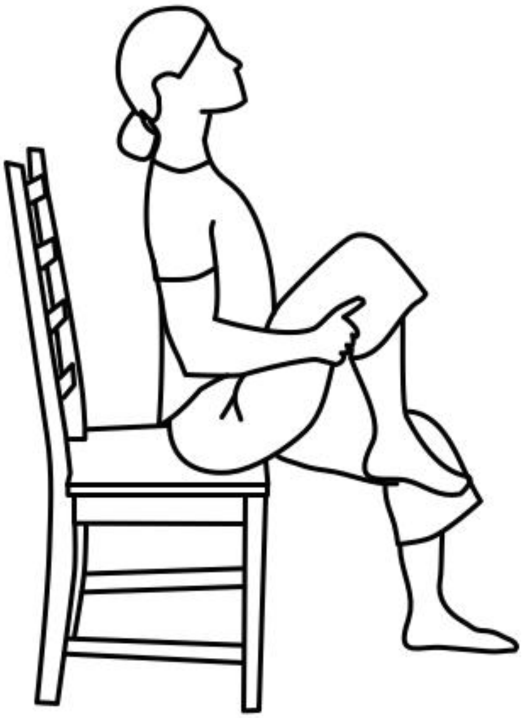

Knees to Chest in Chair

© Boston Medical Center
Veterans Back to Health Trial

Guidelines for Teaching Specific Yoga Poses

Knees Together Twist Pose

Description: Supine pose that strengthens the spine, back, and the abdominal muscles (particularly the obliques), and reduces stiffness in the spine, lower back, and hips (hip abductors in outer hip/thigh).

How to teach: Start lying on back with feet on the floor, with knees together and bent. Lay arms out on the floor, palms facing up, and level with shoulders. Exhale, bring both knees down to the left, keeping right shoulder down on the floor. Inhale, raise knees back to center. On next exhale, bring both knees down to the right, keeping left shoulder down on the floor. Repeat three times, moving with the breath.

To do this pose at the end of practice, hold knees at side for multiple breaths then repeat on other side.

Some people may find that their back pain may be worsened in this position, while others find it may relieve pain. Check in with participants while doing this pose and advise them accordingly.

Modifications:

1. If opposite shoulder cannot stay down on the floor, place a blanket(s) or block under knees on each side.

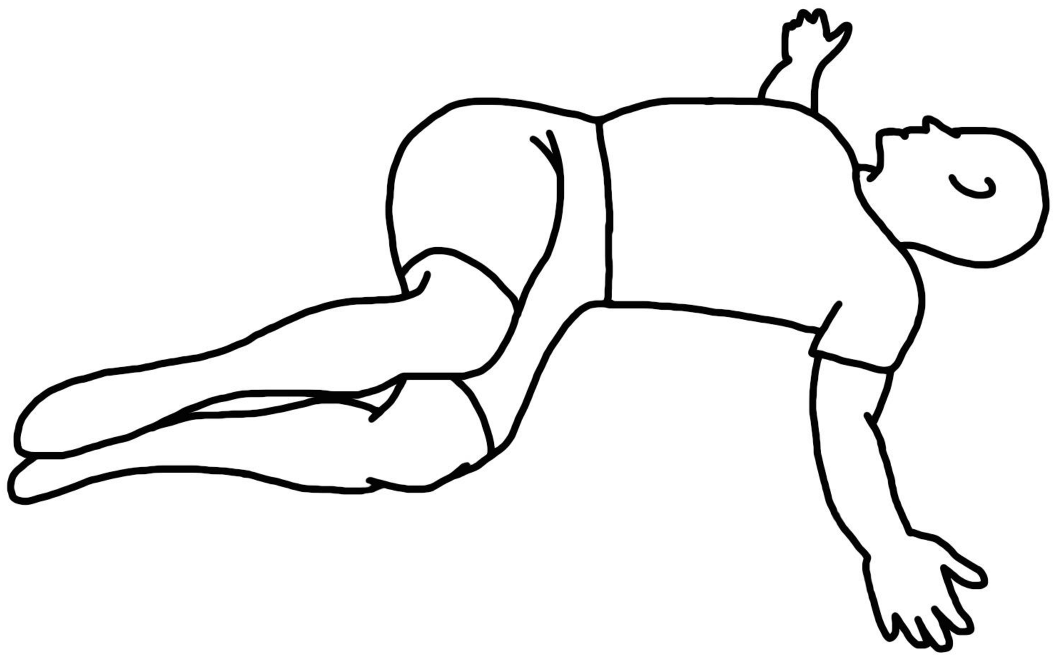

© Boston Medical Center
Veterans Back to Health Trial

Guidelines for Teaching Specific Yoga Poses

Legs up the Wall Pose

Description: Restorative pose that gives sensation of inversion.

How to Teach: Sit sideways to the wall with knees bent. Lie down on one side and roll onto back while bringing legs up the wall.

Caution participants that anyone with elevated eye pressure or glaucoma should avoid this pose.

Modifications:
1. Place folded blanket(s) or bolster under pelvis.
2. For tight legs, have pelvis away from wall 6”-10”.
3. Participants with chronic neck pain or “arthritis” of the neck should try pose with pelvis away from wall 6”-12” or avoid pose altogether.

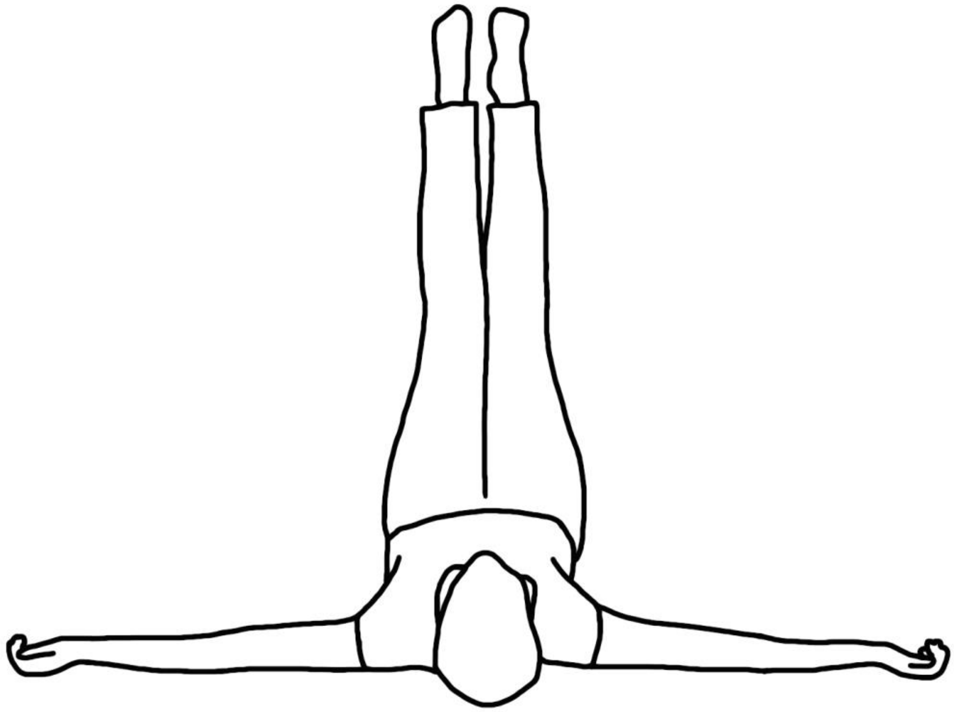

© Boston Medical Center
Veterans Back to Health Trial

Guidelines for Teaching Specific Yoga Poses

Locust Pose

Description: Mild backbend that strengthens and increases flexibility throughout the entire back of the body, including the spine, legs, buttocks, and all of the muscles surrounding the ribs and upper torso.

How to teach: Lie on belly with head on folded forearms or forehead on the mat. Be on the tips of the toes. To start, raise one leg at a time, keeping front hips and tailbone pressing into the floor. Foot on the floor can have toes curled under or feet flat on the floor. Then raise both legs together, pressing tailbone down into floor. Lower legs and then with arms down by the sides of body and palms facing up, raise head, chest and arms, while pressing legs down.

To prevent injury, caution participants to not overextend the neck. Encourage them to keep the spine of the neck in line with the spine of the upper back and to look forward (not up) or slightly down.

Modifications:

1. Bring arms by the sides with the palms facing up. Point big toes behind and away from you. Inhale and lift head, upper chest, arms and legs off the mat at the same time. Lightly press pubic bone into the mat to create more space in low back. To release, exhale as head, arms and legs come back to the floor.
2. Stand facing a chair: Bend forward and hold on the seat of a chair. Extend one leg back, inhale, and slowly raise the leg straight up off the floor. Exhale, bring it down.
3. Sitting in Chair: Place feet firmly on floor with the arms by the sides and move the shoulders down away from the ears. Roll the upper arms back to open the chest. Squeeze shoulder blades slightly into each other and pull abdomen in towards the spine. Extend arms straight back and behind.

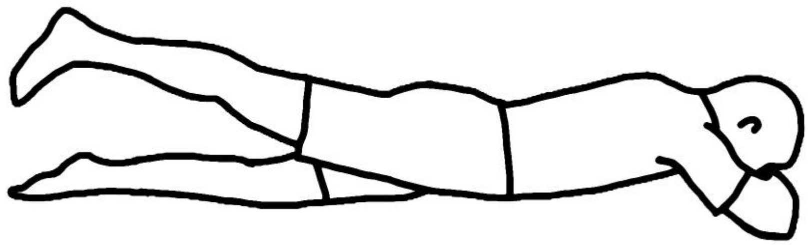

Half Locust Pose with One Leg Lifted

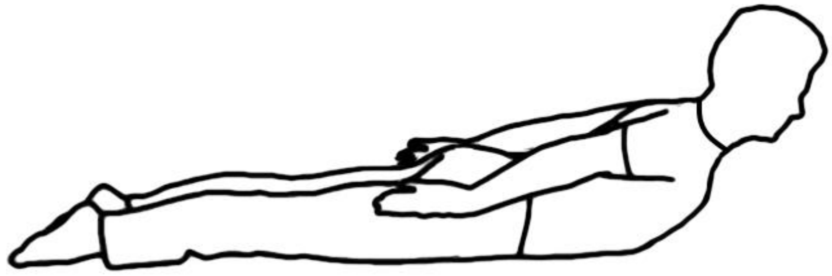

Half Locust Pose with Upper Body Lifted

© Boston Medical Center
Veterans Back to Health Trial

Guidelines for Teaching Specific Yoga Poses

Mountain Pose

Description: Foundational standing pose that helps improve posture, balance, and calm focus.*

How to teach: Stand with feet facing forward and hip-width apart, balancing weight evenly on the four corners of feet (big toe, little toe, inner heel, and outer heel). Align thighs over ankles, hips over thighs, shoulders over hips, and ears over shoulders. Bring shoulder blades down into back while lifting chest up. Lengthen neck and look straight ahead. Extend arms by the sides.

Modifications:

1. Stand with the heels, sacrum, and shoulder blades (but not head) against a wall.
2. Lie on floor with legs straight and feet pressing into wall. Arms by sides, shoulders pressing down toward the floor. Elongate back of the neck.
3. Sitting in Chair: With back straight, find where the weight is even on both sitting bones by leaning slightly to alternating sides. With hands on hips, slightly tip hips forward and back to find center. Align head over hips, ears over shoulders, and shoulders over hips. Extend arms down by the side of the torso. Relax the shoulders and lift chest up. Look straight ahead with a relaxed face and long neck.
4. Balance: Shift weight to the left leg without letting the left hip push out to the side. Spread the toes of the left foot and feel the body weight come onto the left foot evenly on the four corners of the foot. Engage the thigh muscles by contracting the quadriceps and hamstrings. Standing tall and firmly on the left leg, slowly raise the heel of the right leg up off the floor. Hold for a moment. Repeat on right.

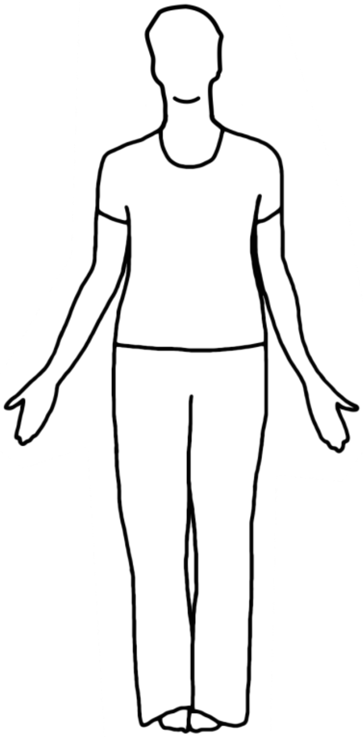

*For Veterans that have left the military more recently, this pose may be reminiscent of 'standing at attention' and could cue them to tighten the spine and buttocks out of habit. Teachers may choose to use verbiage that recognizes the similarity but tells students to do something different, remain relaxed, and be aware of slow breathing throughout. For example, "*This pose can be similar to standing at attention in the military; however, instead of tensing up all the muscles, allow them to soften*."

© Boston Medical Center
Veterans Back to Health Trial

Guidelines for Teaching Specific Yoga Poses

Pelvic Tilt Pose

Description: Supine pose with subtle spinal movements that strengthen the supporting muscles around the low back, particularly the abdominals.

How to teach: Lying on back, bend knees bringing feet in toward lower pelvis, thighs parallel to each other. Extend the arms down toward the feet, palms downward on the mat and relax the shoulders. Inhale and relax, maintaining the natural curve of the lower back. Exhale, gently press the lower back into the floor, flatting the natural curve and slightly lifting the lower pelvis and tailbone up toward the ceiling. Inhale, relax and return to the original position with the natural curve of the back restored. Repeat slowly several times, keeping the shoulders and head relaxed. This will result in a gentle rocking motion of the pelvis.

Modifications:

1. Use a block between the knees to keep them parallel to each other if the knees are splaying out wider than hips.
2. Pelvic Tilt at Wall (a bit more difficult): Stand with back and head against wall, feet away from wall and slightly bent. On exhale, gently press lower back into wall, relax on inhale.
3. Sitting in Chair: With back straight and feet firmly planted on the floor, exhale, gently press lower back toward the back of the chair, slightly rounding the back. On inhale, sit back upright. Repeat several times.

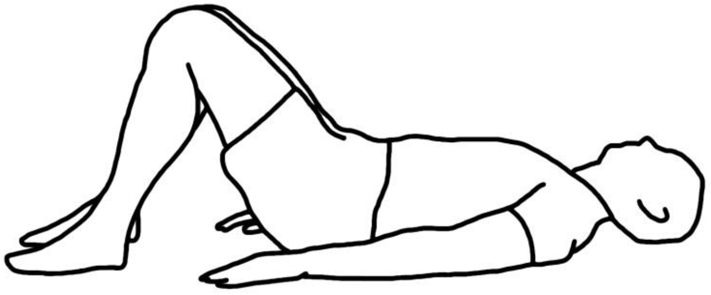

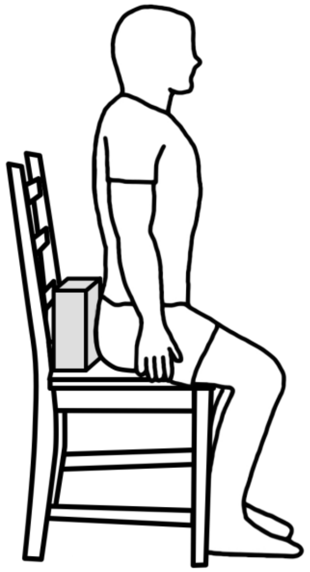

Pelvic Tilt in Chair

© Boston Medical Center
Veterans Back to Health Trial

Guidelines for Teaching Specific Yoga Poses

Plank Pose

Description: Arm-balancing pose that tones abdominals while strengthening arms and spine.

How to Teach: Starting on hands and knees, extend legs backward one at a time keeping feet in line with palms and toes curled under. As you inhale, press down with the hands to push the floor away, pull your rib cage away from the floor, and without rounding the spine, move into the plank position, bringing the shoulders over the wrists and aligning the shoulders, hips and ankles in one straight line. Keep the muscles of the front of the thighs engaged; contract the abdominal muscles in toward the spine; and the head raised in line with the spine. Hold for two to three slow breaths.

Modifications:

1. **Half Plank Pose**: Keep knees on the ground, making sure to keep body and neck in a straight line.
2. If wrists are uncomfortable, bring elbows down and come onto forearms; or roll end of yoga mat under hands to relieve pressure.
3. Keep soles of feet pressed against a wall and balls of feet on the floor.
4. Wall Plank: Stand facing a wall with feet hip-width apart. Place palms against wall, arms extended, and slowly lean forward, letting body rest on hands. Keep arms and body in a straight line as you slowly bend elbows, inching closer to the wall. When nose touches wall (or if you feel pain), slowly push back to standing.

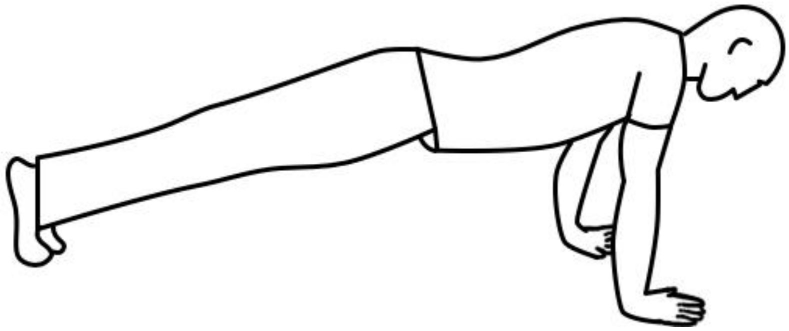

Plank Pose

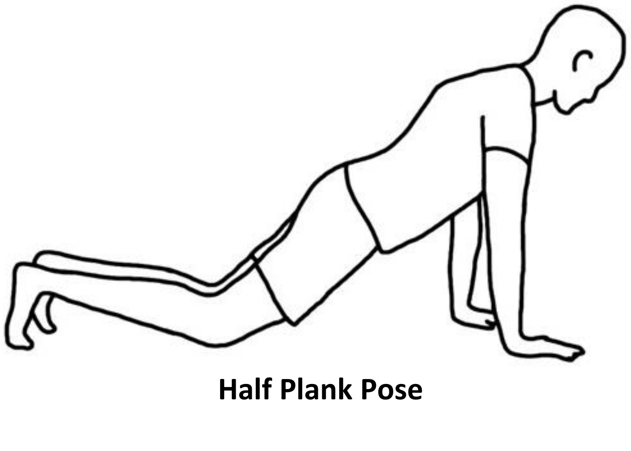

Half Plank Pose

© Boston Medical Center
Veterans Back to Health Trial

Reclining Chest Opener Pose

Description: Resting prone pose that opens the chest to facilitate breathing and relaxation.

How to teach: Place a narrowly folded blanket horizontally across the mat so that it will be under the upper back but not under the shoulders. The blanket comes under the bottom of the arms when the arms are stretched out on the floor sideways at shoulder height. Lie down in Final Resting Pose with the back of the head on the floor or on a blanket.

Modifications:
1. For more of a chest opening, use two blankets or a block. Be sure the neck stays in a neutral position and does not hyperextend.
2. If there is lower back discomfort, place a bolster or rolled blanket(s) under knees, bend knees, or come out of the pose.

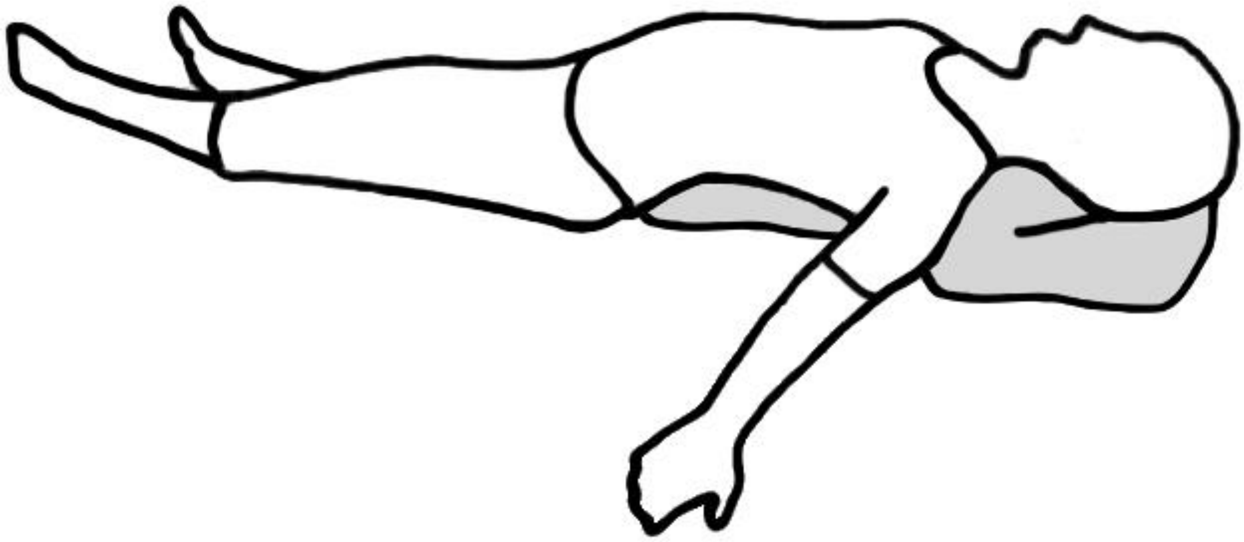

© Boston Medical Center
Veterans Back to Health Trial

Guidelines for Teaching Specific Yoga Poses

Reclining Cobbler Pose

Description: Supine pose to stretch and strengthen inner thighs and hips.

How to teach: Begin by lying on back with knees bent. Bring soles of feet together and let knees drop to either side of the mat. Place folded blankets, blocks, or bolsters under outer thighs for support if needed. Make sure the pose is comfortable and there is no strain in the hips or inner thighs. Place a blanket over lower half of body if desired.* Breathe and relax in posture. To come out, take hands and gently move knees together.

Modifications:
1. For lower back discomfort, fold 1-2 blankets lengthwise to support length of spine from waist to head. Do not have the blankets right up to the tailbone, but rather have space from waist to tailbone.
2. If there is back discomfort after coming out of posture, lie flat on the floor and hug both knees to the chest, gently rocking side to side.

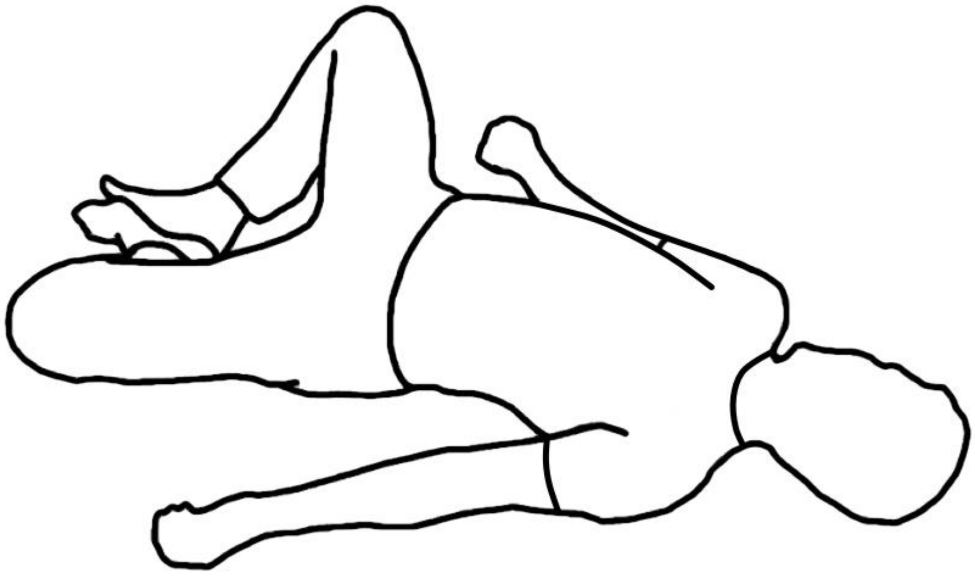

*A Veteran may feel psychologically uncomfortable or vulnerable in this position, particularly if there is a history of trauma. Provide the option to all Veterans of using a blanket to cover the lower half of the body in they prefer.

© Boston Medical Center
Veterans Back to Health Trial

Guidelines for Teaching Specific Yoga Poses

Shoulder Opener Poses

Description: Standing pose to stretch shoulders and upper back.*

How to teach: From Mountain Pose, hold a belt behind the back with the palms facing up and hands apart. Stretch the arms straight down as the chest lifts up. Bring the shoulders down. If possible raise the arms up without rounding the shoulders or dropping the chest. Breathe and relax into the stretch of the front chest. Slowly lower arms down and release hands.

Modifications:

1. Clasp hands behind back and slowly raise arms backwards to a comfortable stretch, making sure not to collapse chest. Continue to breathe and draw shoulder blades back toward each other.
2. Sitting in Chair: All of the above can also be completed while seated.

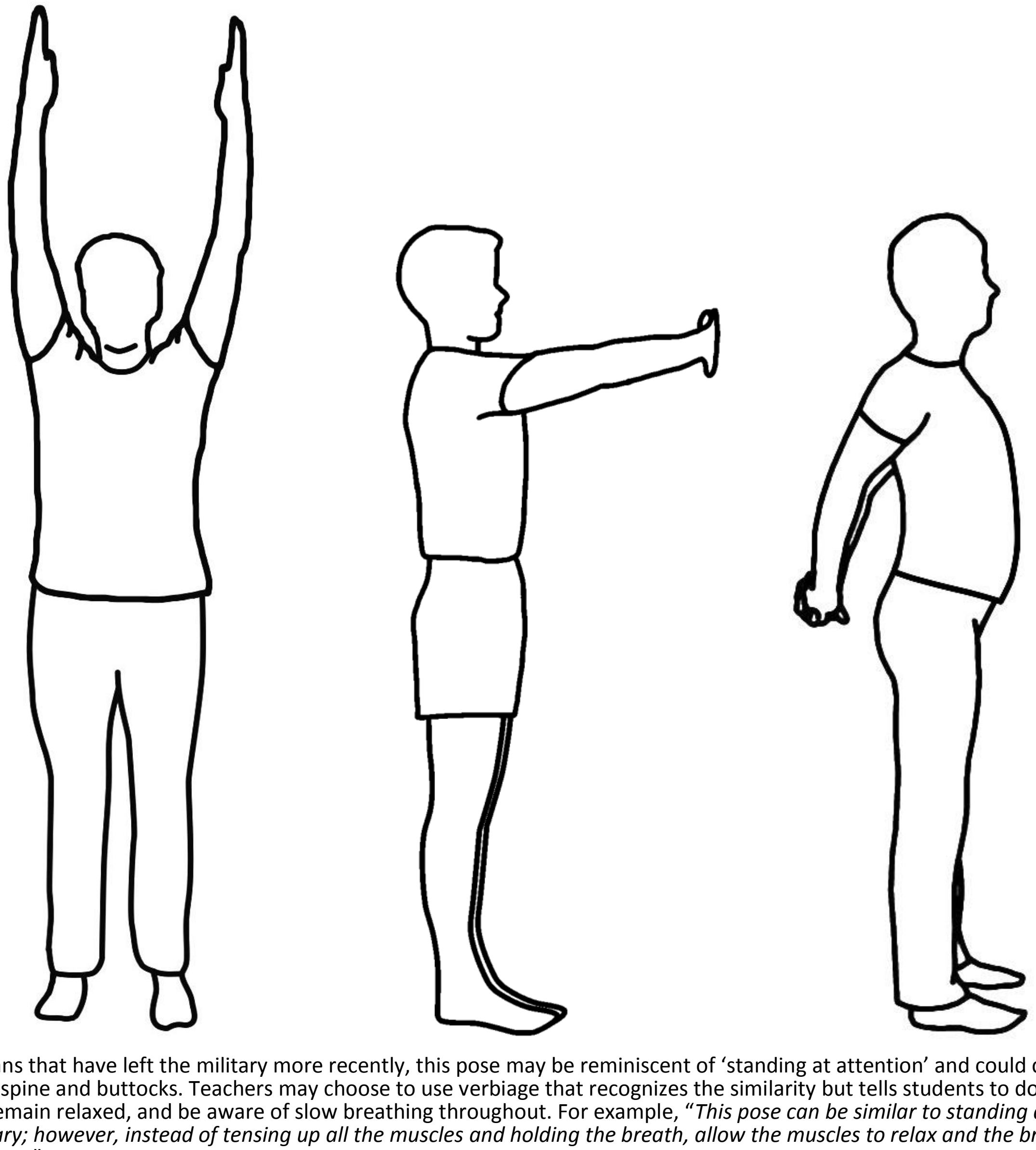

*For Veterans that have left the military more recently, this pose may be reminiscent of 'standing at attention' and could cue them to tighten the spine and buttocks. Teachers may choose to use verbiage that recognizes the similarity but tells students to do something different, remain relaxed, and be aware of slow breathing throughout. For example, "*This pose can be similar to standing at attention in the military; however, instead of tensing up all the muscles and holding the breath, allow the muscles to relax and the breath be slow and deep*."

© Boston Medical Center
Veterans Back to Health Trial

Guidelines for Teaching Specific Yoga Poses

Side Hip Strengtheners

Description: Movements to strengthen hip abductors (muscles in outer thighs/buttocks).

How to teach: Lie on your left side with a blanket under your head to keep your neck comfortable. To get into the starting position (1), bend the knees and hips at 90° angles, so your thighs are perpendicular to your belly and hips stacked on top of one another. Extend the right leg straight out so the heel is in line with the crown of the head, allowing the lower leg to be relaxed. Rotate the right thigh inward, so the knee and toes point down (2). Inhale and lift the right leg (3). Exhale and slowly release the leg down into the leg extended position (2). Begin with a single repetition and gradually increase up to three repetitions, if appropriate. Repeat laying on right side, lifting the left leg.

Modifications:

1. Do not extend top leg before lifting. Lying on the left side in the starting position (1), isolating the uppermost portion of your outer right thigh, lift the right leg on an inhale, keeping it bent at a 90° angle and allowing the lower leg to be relaxed. On the exhale, slowly lower the leg down. Start with two repetitions and gradually increase, if appropriate, to six repetitions.
2. If at any point the weight of the leg feels too heavy, bend the knee appropriately.
3. Repeat leg movements at different angles. Try movements with the thighs at a 135° angle to the torso (4:30 position, if head is 12:00 and legs straight down is 6:00).

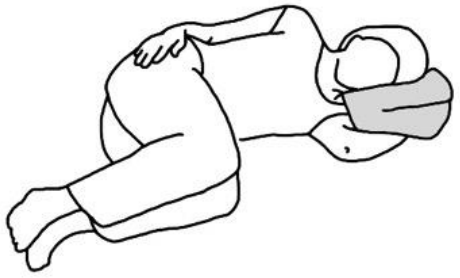

1. Starting Position

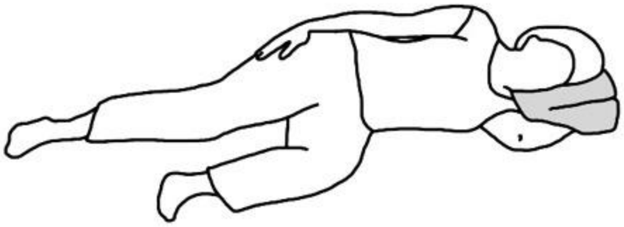

2. Leg Extended Position

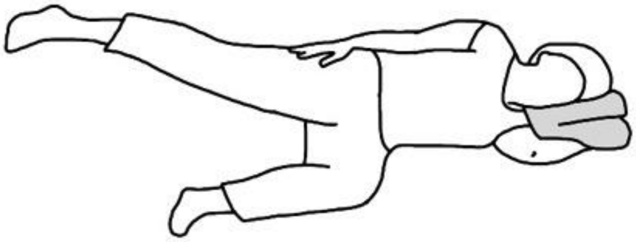

3. Leg Extended and Lifted Position

© Boston Medical Center
Veterans Back to Health Trial

Guidelines for Teaching Specific Yoga Poses

Side Plank Pose

Description: Balancing pose to strengthen abdomen, arms, shoulders, and spine.

How to teach: From the plank position with feet at the wall, turn onto the outer edge of your right foot and bend your right forearm, placing the left hand on the waist. Press the right hand into the floor and keep arm straight. Lift and lengthen the sides of the rib cage and hold for 2-3 breaths. Repeat this variation of Side Plank on the other side.

Modifications:

1. **Half Side Plank**: Lie on the right side with the ankle, hip, shoulders and head aligned in a straight line with the feet at a wall. Bend the right leg under the top leg, keeping the top leg straight. Have the left hand on the waist. Keeping the head, shoulders, hips and left ankle in a straight line, on an exhale press down on the hand and inner edge of the left foot to raise the hips up off the floor. Lift and lengthen the chest. Keep the head in a straight line with the body. Do not let it drop forward. Hold for 2-3 breaths; exhale and come down. Repeat on other side.
2. If wrists are uncomfortable, bend right/left elbows, bringing the forearm onto the floor, with hand pointed forward. A block can be placed under the forearm for more height.
3. Chair Support: From the plank position using a chair, follow the above directions to do Side Plank Pose with hand on a chair.

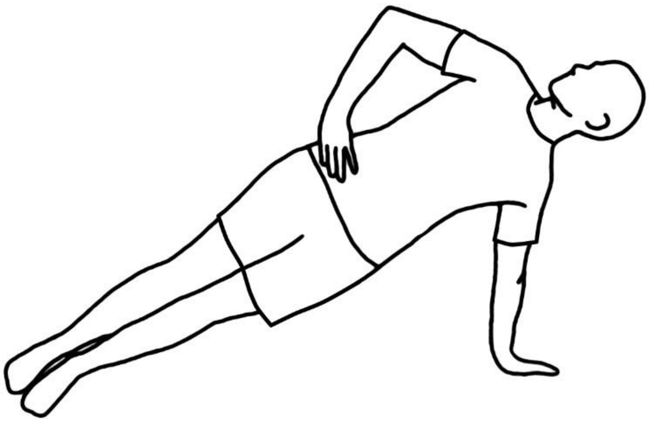

Side Plank Pose

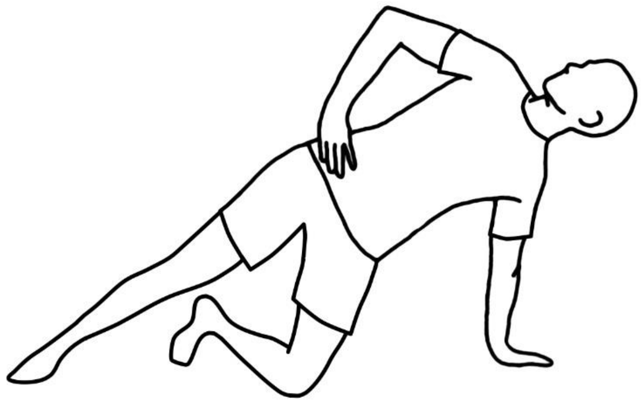

Half Side Plank Pose

© Boston Medical Center
Veterans Back to Health Trial

Guidelines for Teaching Specific Yoga Poses

Sphinx Pose

Description: Beginning/mild backbend that helps to open the chest, lungs, and low back.

How to teach: Lying on stomach place elbows shoulder-distance apart, slightly in front of shoulders, fingers pointing straight ahead. Press forearms firmly down while lifting head and chest up. Minimize weight on elbows or arms by engaging upper back muscles. While inhaling, move shoulders down and pull them back, bringing chest forward. Move elbows forward more if there is discomfort in the back. There should be no strain on the lower back. Exhale, release to the floor.

To prevent injury, caution participants to not overextend the neck. Encourage them to keep the spine of the neck in line with the spine of the upper back and to look forward (not up) or slightly down.

Modifications:

1. Sitting in Chair: With feet firmly on the floor and hands on thighs, allow shoulders to drop down away from ears. Roll upper arms back to open chest. Move shoulder blades slightly towards each other and pull abdominal muscles in. Lift head and chest slightly up.

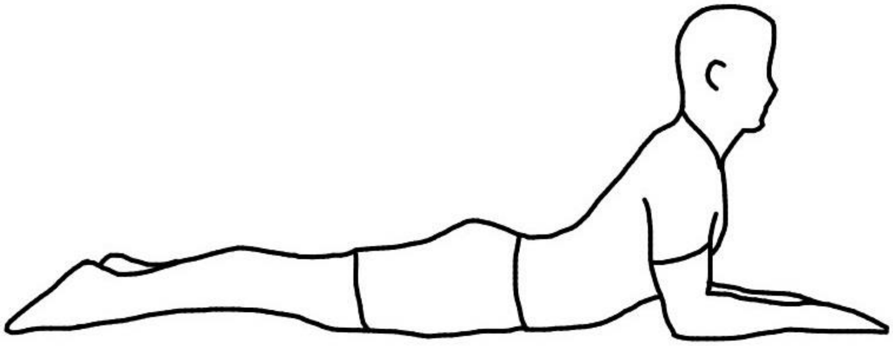

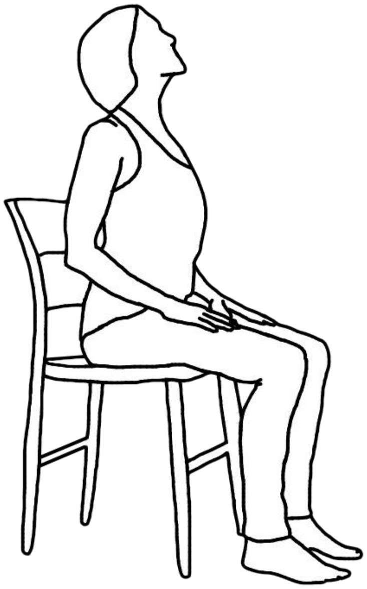

Sphinx in Chair

© Boston Medical Center
Veterans Back to Health Trial

Guidelines for Teaching Specific Yoga Poses

Table Top Pose

Description: Pose that builds abdominal and lower back strength, brings flexibility to the spine, shoulders, and hips, and gently stretches the torso.

How to teach: Begin on hands and knees. Place hands directly under shoulders and knees directly under hips. Back is flat and level like a table. Inhale, extend right leg behind with leg straight and toes on the floor. Exhale, press back through heel. Lift abdomen up into body. Inhale, bring right knee back to return to all fours, and repeat on the other side by extending left leg behind.

To prevent injury, caution participants to not overextend the neck. Encourage them to keep the spine of the neck in line with the spine of the upper back and to look forward (not up) or slightly down.

Modifications:

1. If there is wrist discomfort, make a fist with both hands resting weight on knuckles with thumbs facing down.
2. If participants need more cushioning under knees or hands, roll sides of mat under the knees or top of the mat under hands.
3. Rest your forearms on the floor rather than your hands.
4. Standing with hands on the back of a chair (bending forward) or against the wall, extend one leg at a time back with the toes down on the floor.
5. Sitting in Chair: Sit on the edge and extend one leg out in front with the heel on the floor. Press through the heel. Repeat with opposite leg.

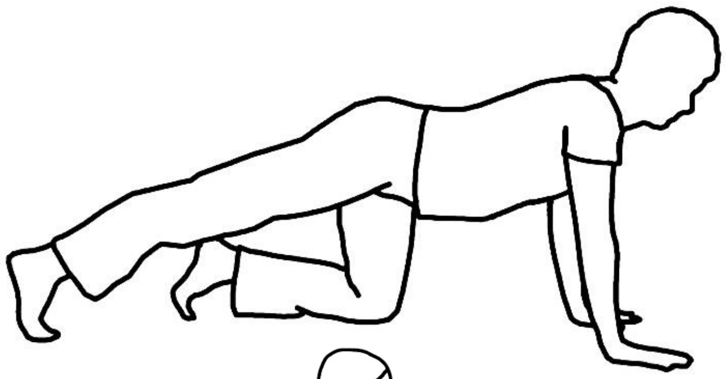

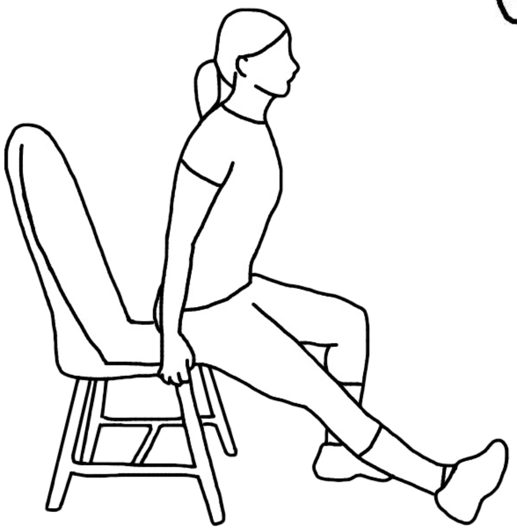

Forward Bend in Chair

© Boston Medical Center
Veterans Back to Health Trial

Guidelines for Teaching Specific Yoga Poses

Triangle Pose

Description: Standing pose that stretches hamstrings and hips, and strengthens muscles in the thighs, hips, and back.

How to teach: Separate feet wider than hip-width, keeping feet parallel and toes pointing forward. Extend arms out to sides at shoulder height with palms facing down. Turn left foot out 90°. Turn right foot slightly inwards. Exhale, bend to the left, bending sideways from the hips. Press hips to the right (opposite of bend) and drop left hand to block, chair seat, or leg directly under shoulder. If there is no pain, raise right arm above shoulder, otherwise place right hand on hip or rest on side of body. Keep legs, arms and back straight and in the same plane. Keep hips facing forward. Inhale back to standing. Repeat on other side.

Modification:

1. **Triangle at Wall**: Stand with left side facing the wall, feet wider than hip-width apart and left hand on wall. Turn left leg toward wall with toes close to or touching the wall. Turn right foot slightly in so that the right foot is at a diagonal. Bend at hip toward the wall, and move right hand up above head or keep on waist. Keep head, left leg, and left foot aligned with each other in the same plane.
2. **Heel at Wall**: Stand with right side facing the wall, feet wider than hip-width apart and right hand on wall. Turn the left leg out so that the left foot and thigh are in a straight line pointing away from the wall. Turn right foot slightly inward, keeping the heel at the wall. Exhale, bend left from the hips, pressing right heel into wall gently.
3. Stand with the back to a wall using the wall to lean the hip, upper back and head on.
4. Sitting in Chair: Extend left leg out to side turning the whole leg out 90° with the toes pointing to the wall at the side. The right leg is bent with the foot on the floor. Bend to the left side at the hips, bending directly over the extended leg and place the left hand on the leg.

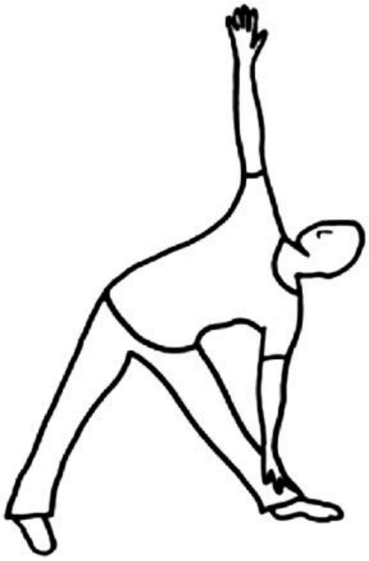

Triangle Pose

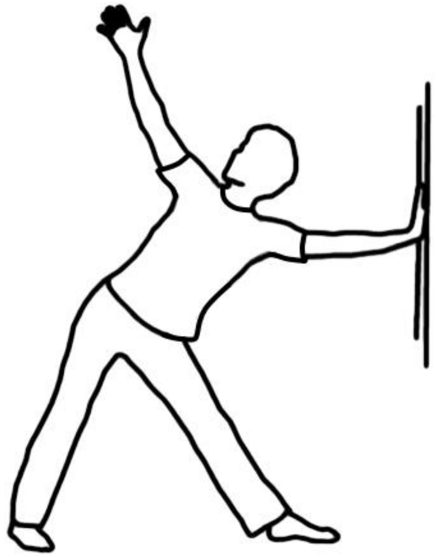

Triangle at Wall Pose

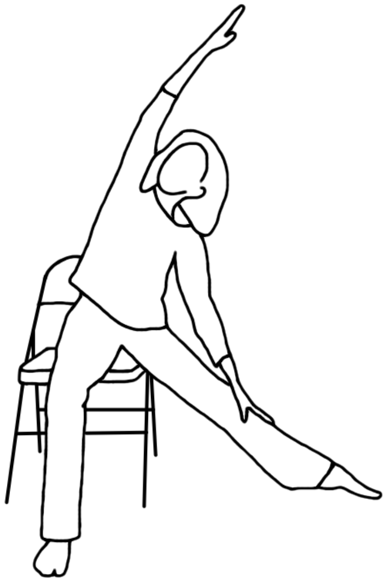

Triangle Pose in Chair

© Boston Medical Center
Veterans Back to Health Trial

Guidelines for Teaching Specific Yoga Poses

Warrior I Pose

Description: Standing pose that stretches the whole front side of the body while strengthening the thighs, ankles, and back.

How to teach: From Mountain Pose, step back with left foot so the legs are wide apart in a comfortable stance. The left foot should not be directly behind the right foot but should be behind the left hip. Turn the back foot and thigh slightly out and keep the front foot pointing straight. Turn the hips to face forward, possibly guiding them with hands on hips. Stretch the arms out to the sides at shoulder level. Straighten the back leg and while lifting the abdomen up toward the chest, bring the tailbone down closer to the floor. While pressing the back heel down and lifting the abdomen upwards toward crown of the head, bend the front knee so that the knee is directly over the ankle and the thigh is as parallel to the floor as possible. If unsteady, make a wider stance by moving back foot further away from the midline. Keeping the legs stable, lift the side ribs up. On an inhale, raise arms straight up in line with the ears while lengthening the spine, being careful to not overarch the lower back. To come out of pose, exhale and move arms down, straighten the front leg, and step forward. Repeat on other side.

Modifications:

1. Bend elbows to make arms like goalposts or place hands on hips if shoulders are painful.
2. Offer option to straighten and bend front leg, flowing in & out of pose with the breath.
3. **Heel at Wall**: Stand with back facing the wall and step right foot forward a few feet. Turn the back foot out at a diagonal, keeping the heel at the wall, and stretch the arms out to the sides, level with the shoulders. Guide hips to face forward. Press the back heel into the wall to stabilize the leg while moving the tailbone down. While lifting the crown of the head, torso and abdomen upwards, bend the front knee so that the knee is directly over the ankle and the thigh is as parallel to the floor as possible. Inhale and raise arms straight up.
4. Sitting in Chair: Using a chair without arms, sit sideways on the chair with the right leg bent into a right angle and the left leg extended behind. Facing sideways on the chair, raise the arms up overhead and lift chest, or keep arms on the hips if more comfortable.

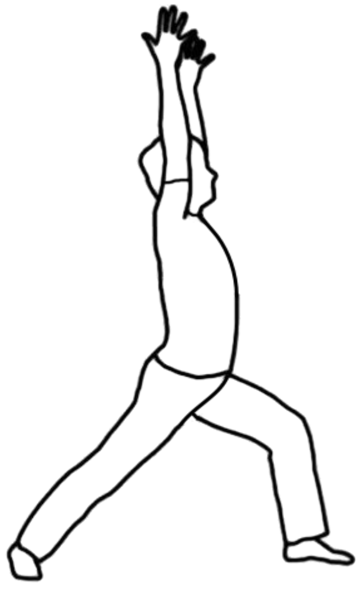

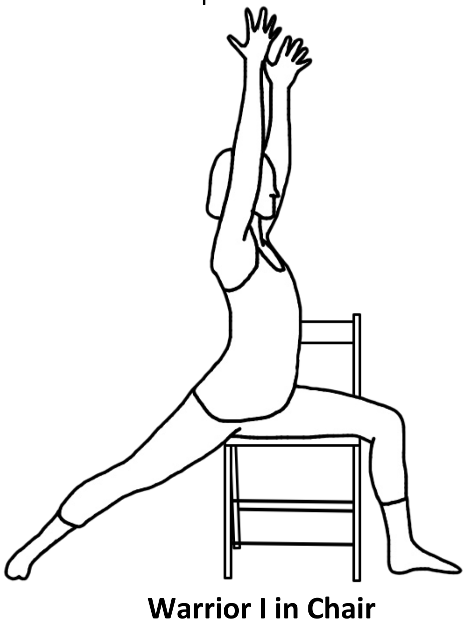

Warrior I in Chair

© Boston Medical Center
Veterans Back to Health Trial

Guidelines for Teaching Specific Yoga Poses

Warrior II Pose

Description: A hip-opening pose that strengthens muscles in the thighs and buttocks, tones the abdomen, and opens the chest and shoulders.

How to teach: Move the feet apart into a wide but comfortable stance. Keep feet parallel with toes pointing forward. Extend arms out to the sides with palms facing down. Move shoulder blades down away from ears while lifting chest up. Turn right leg and foot out 90˚ and turn left foot in slightly. Press the outer edge of the left heel down to straighten and stabilize the leg. Exhale and bend into the right knee so the knee is over the ankle, making the thigh as parallel to the floor as possible. Adjust the distance between the feet so that the knee is over the ankle. Keep back leg straight and firm by pressing the outer edge of the foot firmly down. Move the tailbone down toward the floor and lift up with the abdominals. Look toward the right hand. Extend equally through both arms and legs to keep the shoulders over the hips, the spine straight, and the sides of the trunk equal in length. To come out of pose, look forward, straighten right leg, turn feet forward and repeat on other side, this time bending the left leg.

Modifications

1. Offer option to straighten and bend front leg, flowing in & out of pose with the breath.
2. **Heel at Wall**: Stand with the side of the left foot against a wall. Step the right foot out a few feet. Turn the right leg out to face away from the wall and turn the left foot slightly in keeping the heel at the wall. Extend arms out to the sides and move shoulders down. Exhale, press the left heel into the wall gently, and bend into the right knee. Keeping left leg stable by pressing into the wall, move the tailbone down toward the floor and lift up with the abdominals. Look toward the right hand while extending the left arm back with the left fingers reaching to the wall at shoulder level. Extend through arms and legs.
3. Facing a wall, place a block between bent knee and wall to align and stabilize the knee.
4. Sitting in Chair: With the right knee bent into a right angle, sit the right thigh on the chair seat with the left leg stretching back in the opposite direction. The torso is facing the front, not sideways. Keeping the spine vertical with the shoulders aligned over the hips, stretch both arms out equally away from each other at shoulder height. Look to the right.

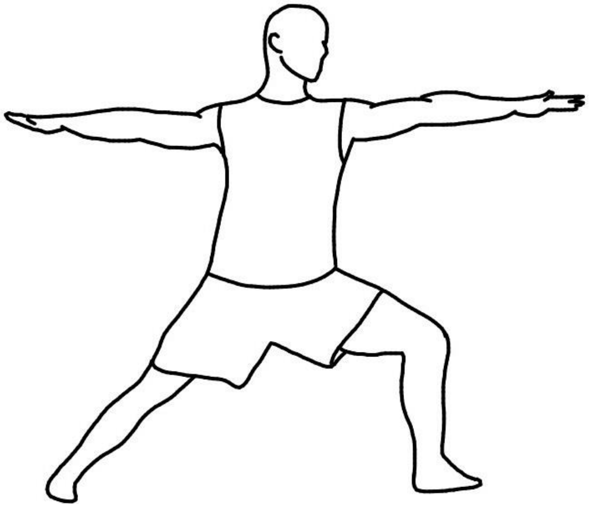

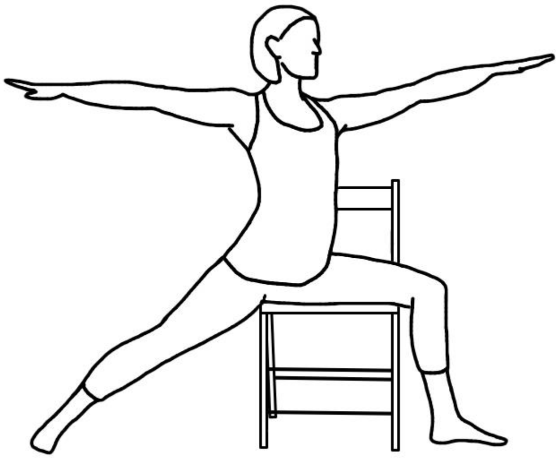

Warrior II in Chair

© Boston Medical Center
Veterans Back to Health Trial

Guidelines for Teaching Specific Yoga Poses

Warrior at Wall Pose

Description: A version of Warrior I using a wall for support and balance.

How to teach: Stand facing the wall, with hands on the wall at shoulder height. Step right foot up close to the wall and step left leg back to a wide but comfortable stance. Turn back foot out slightly and stretch through back leg, pressing the back heel firmly down to stabilize the leg. Lift abdomen up while moving the lower back down, making sure not to arch lower back. Lift chest up and move the shoulders down. On an exhale, keep the tailbone moving down toward the floor and lifting up with the abdominals as the front leg bends so that the knee is directly over the ankle and the thigh is as parallel to the floor as possible. Keep the spine upright with the shoulders over the hips. Press slightly down in the hands to bring the shoulders down and the chest up. Stay for several breaths, inhale and straighten front leg to come out. Step back foot forward and repeat on left.

Modifications:

1. Offer option to straighten and bend front leg, flowing in and out of pose with the breath.
2. Place a block between the bent knee and the wall to align the knee over the foot and to stabilize it.
3. Sitting in Chair: See modification #4 of Warrior I on p. 64.

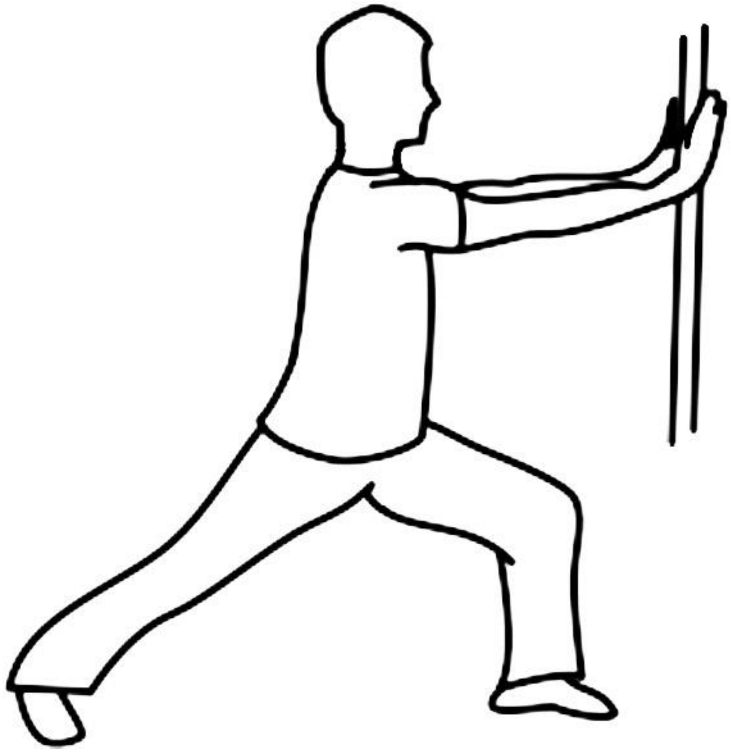

© Boston Medical Center
Veterans Back to Health Trial

Guidelines for Teaching Specific Yoga Poses

Wheel Pose

Description: A posture that requires movement between two positions that contract and stretch the back and neck. In this pose, practitioners do not hold the postures, but rather emphasize a slow continuous movement with the breath.

How to teach: Start on hands and knees, with your shoulders positioned directly over the wrists and your hips directly over the knees. As you inhale, gently arch the back, lifting the chest away from the belly. Be careful to keep the chin slightly down and avoid compressing the low back. Exhale, gently contract the belly – rounding the back slightly as you move the chest toward the thighs and the hips backward toward the heels. As you inhale again, come back to all fours with the chest lifted and the back gently arched. Continue to repeat this sequence to create a flowing movement with the breath.

Modifications:

1. If flat hands cause wrist discomfort, make hands into fists, resting weight on knuckles with thumbs facing down. Or roll top of mat under hands.
2. If knees are uncomfortable, roll sides of mat under knees for padding or do it in a chair.
3. If feet cramp when flat on ground, keep toes curled under feet.
4. Sitting in Chair: Sit at the edge of the chair with thighs parallel to the ground, knees hip-width apart and the feet under the knees. The hands rest palms down on the thighs or knees. Inhale, gently slightly arching the back lifting the chest up without compressing the lower back and keeping the chin slightly down as to avoid overextending the neck. On an exhale, gently contract the belly, rounding the back slightly without collapsing the chest over the belly so that the head moves toward the floor, while the hands move down the legs to the calves. This movement should be natural – no need to try to go as far down as possible with the hands.

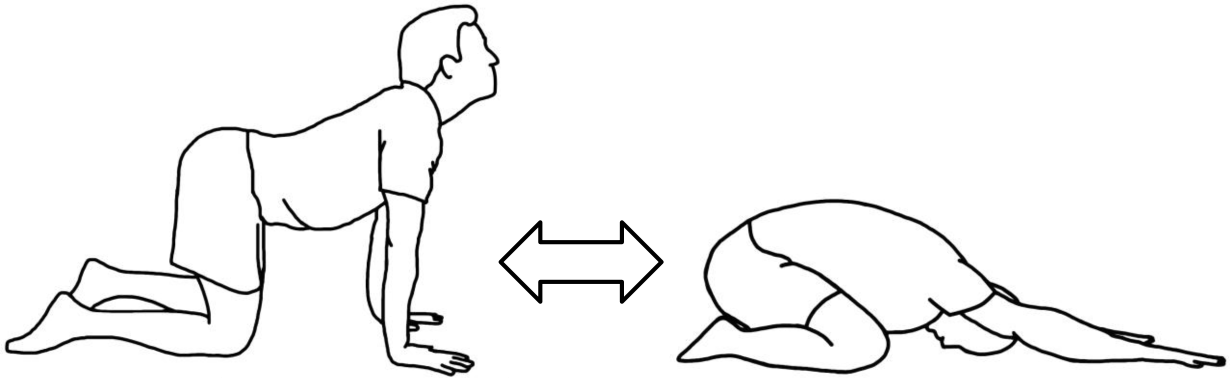

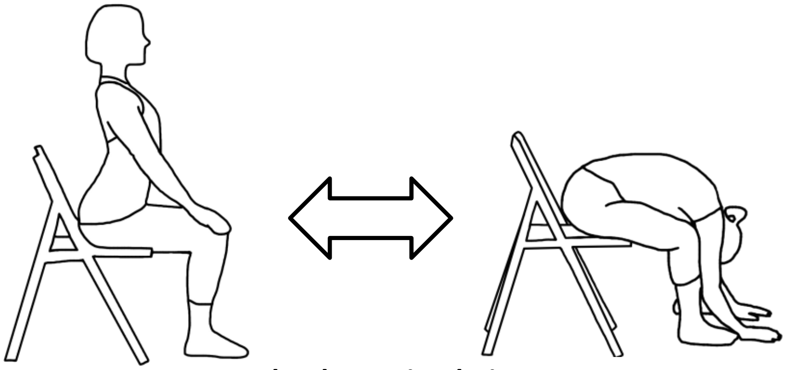

Wheel Pose in Chair

© Boston Medical Center
Veterans Back to Health Trial

Guidelines for Teaching Specific Yoga Poses

Wide-Leg Bend Pose

Description: Standing pose to stretch the back and hamstring muscles.

How to teach: Move the feet apart into a wide but comfortable stance, keeping toes pointing forward. Inhale and lift your chest, making the front torso slightly longer than the back. Bend forward from the hips, keeping the back straight and the neck in line with the backbone. Many participants will not be able to make their torso parallel with the floor, so hands can be placed on the back of a chair, move down to the seat of a chair, or rest on blocks. Or hands can be placed on the wall at shoulder-height to start, and then moved down gradually over time. Keep the weight balanced evenly on both feet and keep the legs straight. Stay in the pose for a few breaths. To come out, lift and lengthen your front torso, keeping a flat back, into Mountain Pose.

In forward bends, the angle between the legs and torso should never be below ~70° (a right angle is 90°).

Modifications:

1. For tight hamstrings, bend the knees.
2. Place the side of one foot against a wall for support if lowering hands to blocks or a chair.

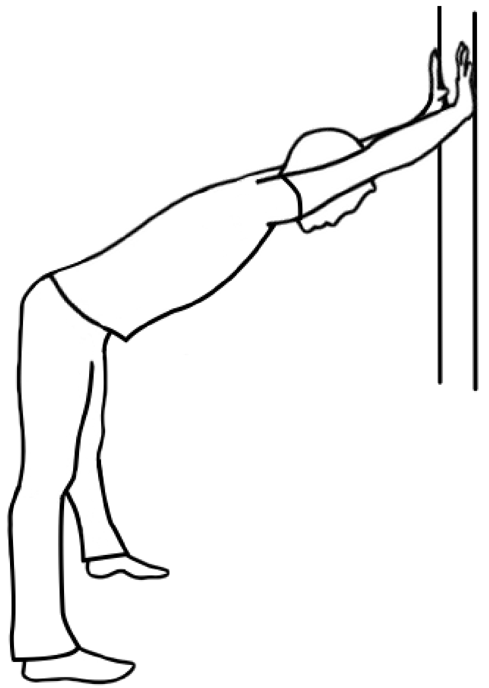

Wide-Leg Bend at Wall

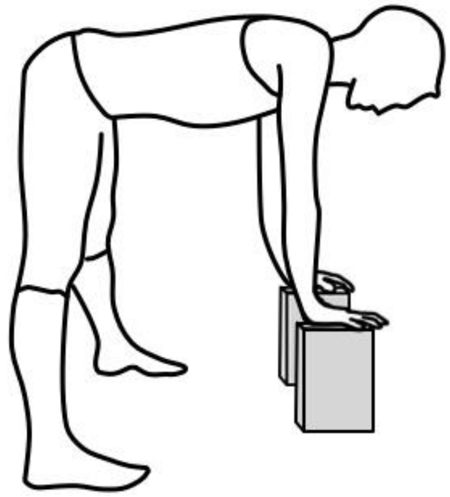

Wide-Leg Bend with Blocks

© Boston Medical Center
Veterans Back to Health Trial

Guidelines for Teaching Yoga Breathing Exercises

Preparing for Breathing Exercises

Specific training in breathing exercises is done at the beginning of each yoga class and should last for about 5-6 minutes. Of course, this is not the only part of the class where breathing is emphasized. Teachers should continually encourage and remind participants to maintain yoga breathing throughout the class, and in particular try to coordinate the breath with the yoga postures. Given that many Veterans may have little or no experience with consciously controlling the breath for yoga, a special period in the beginning of each class is set aside to gain greater confidence in this. During the first few weeks, explain the purpose of the breathing exercises, for example, as follows: *We begin class this way to help us release all the activities of the day and shift the focus on yourself – your body, breath, and movement. The breathing and centering exercise allows you to let go of everything that happens off your mat and begin to focus on your body and the present moment. Breathing exercises can also help ease pain, reduce stress and anxiety, promote restful sleep, and increase attention and focus.*

Breathing exercises may be done sitting in a chair, sitting against a wall or on the floor, lying down on the back, or in any other comfortable position. For participants lying on the mat, consider setting up 2 blankets, one folded in a long rectangular shape to go the length of the spine from the waist to the top of the head. The other folded like a pillow can be placed on top of the first blanket, going under the neck and head. A bolster or rolled blanket(s) can be placed under the knees. Participants may also choose to keep the knees bent and feet flat on the floor hip-width apart.

Guiding Participants through Breathing Exercises

Use the breathing exercises listed on the following pages to guide participants. Of the many yoga breathing exercises available, the core breathing exercise to be taught in this study is slow deep paced diaphragmatic breathing. This is termed 'Yoga Breathing' or 'The Yoga Breath' for the participants. For many of the participants, it may take several weeks for Veterans to become comfortable with this new practice. Encourage participants to practice breathing at home with the home practice video. For Veterans who become comfortable with The Yoga Breath, you can introduce the *Ujjayi* Breath. This should be termed 'The Ocean Breath' for participants. The instructions below are meant as a guide. Teachers can use their discretion in how they teach Yoga Breathing and the pace by which they progress through the instructions. These exercises do not necessarily have to be fully introduced all at once. Since consciously learning how to control the breath may be new, teachers should encourage participants to practice yoga breathing without self-judgment or criticism.

Once participants have found a comfortable position, start with relaxation, closing eyes lightly or keeping them open if preferred. Always begin by giving a moment for participants to become aware of their normal breathing. Emphasize that it is very important that breathing always remains comfortable, never forcing it or pushing it. If there is any struggle, return to normal breathing to recover.

Ending the Breathing Exercise

To end a breathing exercise, instruct participants to let go of the attention to the breath and let natural breathing resume when they are ready. Have them open their eyes, if they chose to

© Boston Medical Center
Veterans Back to Health Trial

close them, and bring their awareness back to the yoga class room. Give them a moment to remain in the calm, peaceful space they have created. Remind them to keep with the rhythm of their breath throughout yoga practice. If they lose this rhythm, they can take a moment to pause and reconnect. Reassure them that this takes practice.

© Boston Medical Center
Veterans Back to Health Trial

Guidelines for Teaching Yoga Breathing Exercises

Yoga Breathing or The Yoga Breath

Purpose and Benefits: Increases oxygen levels by expanding lung capacity, quiets and calms the nervous system, reduces stress and anxiety, and improves self-awareness.

Suggested Instruction

Awareness of the Normal Breath

Breathing through the nose with the mouth closed, notice the feel of the air as it comes into your body and then goes out. If you are unable to breathe through the nose, it is fine to breathe through the mouth. Be aware of your body breathing in, saying to yourself, 'I am breathing in.' Be aware of your body breathing out and saying, 'I am breathing out.' Repeat. Just allow the breath to come. Do not try to control the breath, just be aware of your body breathing without any effort.

Allow the mind and its thoughts to come and go like clouds drifting by. If you notice you have drifted off with a thought, bring your attention back to the breath, noticing your inhale and your exhale.

As you pay attention to your breath, think about the following questions. These questions do not necessarily need answers, they are meant to focus your attention and observe the breath without judgment.

- Notice the quality of the breath. Does the breath feel even? Choppy? Shallow? Is the breath getting stuck in the belly or chest? Tense? Is the breath bumpy or smooth, light or heavy, starting and stopping?
- Notice the feel of the air as it comes into your body and then goes out. Where do you feel the breath in your body the most?
- Notice the temperature of the air as it comes into the nose at the nostrils and again as the air goes out. Does the temperature of the air change from the inhale to the exhale?
- Notice the length of the inhale and exhale. Is the inhale longer or shorter than the exhale?

Three Part Diaphragmatic Breathing

Part 1: The diaphragm is a muscle that is at the base of your lungs and divides your lung cavity from your abdominal cavity. When you take a deep breath during inhalation, the lungs expand. The expansion of the lungs will naturally push down on the diaphragm. As the diaphragm pushes down on the contents of the abdominal cavity, the pressure of the abdominal cavity increases. This increase in pressure causes the belly to rise or expand during the inhale. Place a hand on your belly and notice the movement of the belly. As you inhale, the abdomen naturally rises like a balloon is inside and it is slowly expanding. By doing this during your inhalation, the air initially fills your lower lungs.

As you exhale, the opposite occurs. The lungs deflate as the air leaves. This causes the diaphragm to rise. As the diaphragm rises, the pressure within the abdominal cavity decreases, and the belly falls. Feel the abdomen as you exhale. It should fall as if a balloon inside is slowly deflating.

© Boston Medical Center
Veterans Back to Health Trial

Guidelines for Teaching Yoga Breathing Exercises

If you look at a newborn babies, they naturally breathe like this. However, as adults we forget this natural breath. So do not be disappointed if it takes some practice to retrain yourself.

Part 2: The second part of the three part breath refers to the expansion of the mid-chest with inhalation and contraction of the mid-chest with exhalation. Place your hands on the ribs on each side of your chest. With a relaxed belly, gently breathe in and feel the rib cage expand out into your hands. As you breathe out, feel the rib cage contract away from your hands. Practice breathing, moving the rib cage out into hands and back in, like an accordion—going in and out. By doing this during your inhalation, the air will fill your middle lungs.

Part 3: The third part of the three part breath refers to the expansion and contraction of the upper chest with the breath. The upper part of the lungs are often not used in normal, more shallow breathing. Place your hands on the upper chest, right under the collarbones. Notice if the upper chest has any movement with easy, normal breaths, being careful not to tense the neck and shoulders. If you do this, you will feel the upper chest rise with inhalation. As you breathe out you should feel the upper chest fall. By doing this third part during your inhalation, the air will fill your lower lungs.

Putting It All Together: Breathing of course is a continuous process and cannot truly be broken into discreet and different parts. However, it can be helpful to imagine your inhale as starting with Part 1–rise and expansion of the belly while the lower lungs fill; then beginning Part 2–rise and expansion of your ribcage to allow the mid-lungs to fill with air; and then finally Part 3–rise and expansion of the upper chest allowing the very top of the lungs to fill.

Exhaling can be thought of as a continuous process also but in reverse. The exhale begins with the upper chest contracting or falling as the upper lungs empty out their air. The ribcage then begins to contract emptying out the air from the mid-lungs. And finally, the belly falls and abdominal wall contracts, causing the lower lungs to compress and push out their air. And the inhalation – exhalation cycle repeats.

Lengthening the Exhale

Once participants are comfortable with the mechanics of the three part breath, pacing the breath and ultimately lengthening the exhale to be greater than the inhale can be introduced.

Inhale using the three part breath through the nose. Slowly, deeply, and steadily inhale until you feel full. Lengthening the inhale helps to invigorate and energize us. Notice that there is a slight natural pause at the end of an *inhale* before a new *exhale* begins. Exhale slowly, easily but not all at once.

Continue with three part inhales. Begin to attempt to comfortably lengthen the exhale so it is slow, deep, and steady. Notice that there is a slight natural pause at the end of the exhale before a new inhale begins. Lengthening the exhale helps us relax and release tension and stress. The short natural pause at the end of exhalation can symbolize for you an internal sense of peace and calm.

After several cycles of slow, deep, and steady breaths, begin to lengthen the breath by counting each inhale and exhale. Inhale, slowly counting: 1…2…3. Pause and exhale, slowly counting 1…2…3, pause.

© Boston Medical Center
Veterans Back to Health Trial

Guidelines for Teaching Yoga Breathing Exercises

Note to Teachers: Let participants choose to either stay with a count of 3 on the in-breath and out-breath, or try to lengthen the in-breath and out-breath to a count of 4. If and when they are ready, introduce the idea of lengthening the exhale to a count of 5, while maintaining the out-breath count to 4. Participants can further lengthen the exhale in relation to the inhale using a counting ratio of 6 to 4.

Remind participants to keep their breathing comfortable, never forcing or pushing it. Stay with the rhythm of this breath for a few minutes. Ultimately, if participants feel they master this, they can have the option of not formally counting to themselves.

Ocean Breath

Purpose and Benefits: This is an advanced yoga breathing exercise which can be introduced to Veterans who master the three part breath as described above. It can create smooth, slower, and more controlled inhalations and exhalations. It may allow participants to hold poses longer and receive a greater calming effect from the breath. Do not use Sanskrit names of exercises with the Veterans. In this case, refer to it as 'Ocean Breath.'

Suggested Instruction

Exhale through the nose if possible. If not, you can exhale through an opened mouth. Slightly close or contract the back of the throat while exhaling to make a soft quiet "hhh" sound. Keep the back of the throat contracted while inhaling to quietly make a soft quiet "sss" sound.

To learn to make the sound, start an exhale through the mouth. Try using one or several of these images to teach the sound:

- Breathe out as you would to fog a hand mirror.
- Breathe like when you whisper.
- Breathe as you would if you were trying to see your breath on a cold day.
- Breathe so it sounds like a quieter Darth Vader of *Star Wars* fame.

If the sound is not coming, let it go. It will come on its own with practice.

Listening to the sound of the breath helps us stay focused and also gives information about its quality, rhythm and pace. Continue with this breath for several minutes as you notice the sound and sensation at the back of your throat.

© Boston Medical Center
Veterans Back to Health Trial

Guidelines for Teaching Yoga Relaxation Exercises

Preparing for Relaxation Exercises

Relaxation is done at the end of yoga class and should last, in combination with closing, for about 12 minutes. For the first few weeks, explain the purpose of the relaxation exercise. For example, teachers could say: *These practices allow us to find a deep sense of relaxation while still remaining awake and alert. It is a guided meditation that takes us on a tour of our body while maintaining an inner state of awareness. You may find the experience as one of being a being asleep and awake at the same time. The practice settles the mind and relaxes and restores the body.*

Let participants know that if they fall asleep during the relaxation exercise, they should not feel bad. It is common and can be a sign of not getting adequate rest or sleep for the day.

Relaxation exercises are typically done while in Final Resting Pose but this might not necessarily be a relaxing position for some participants. Yoga teachers may present Reclined Chest Opener Pose (p. 56), Legs up the Wall Pose (p. 51), or any of the modifications for Final Resting Pose (p. 76) so all participants can find a comfortable position. Invite participants to choose a position that is most comfortable for them. For some, this could even be sitting up against a wall if they prefer not to lay on their back. Participants may choose to use a blanket to keep warm, as the body temperature tends to drop during the practice when lying down. Teachers may make the following points to help participants find a relaxing position:

- *Remove glasses if you wear any. Remove hair clips from the back of your head.*
- *Keep yourself at a comfortable temperature. If you would like, you can put on a sweater or cover yourself with a blanket.*
- *Find a comfortable position for relaxation. If you are lying down, allow yourself to take up space, moving your legs beyond the edges of the mat and let them turn them outward as they relax. Allow your legs and feet to roll out to the side. Rest your arms away from your sides with your palms facing upwards. You can make any additional adjustments so you are comfortable, such as placing a rolled blanket or bolster under your knees or bending your knees and keeping your feet flat on the ground.*
- *Let your eyes soften or close them if you would like. Let your head be heavy. Circle your wrists and ankles. Breathe naturally. Let your body sink into the floor. If it is comfortable, move your shoulder blades in toward your back, opening and broadening your chest. Gently move your neck from side to side. Relax your face. Let your jaw relax. Let your tongue rest inside your mouth. Feel how the earth supports the weight of your body. Let go of all effort.*

Intentions and Anchors

As the relaxation practice starts, invite participants to begin by setting an intention. Teachers can explain this by saying: *An intention is something that you may want to let go of. It can be a quality or situation that you would like to bring into your life or even a dedication to a loved one.*

Also invite them to connect with an anchor. Teachers can explain this by saying: *An anchor is a person, object, or event that helps ground you and evokes a sense of joy, love, or compassion.*

© Boston Medical Center
Veterans Back to Health Trial

Guidelines for Teaching Yoga Relaxation Exercises

Anchors are helpful during relaxation practice because it grounds us or returns us to the present moment if our mind takes us elsewhere. Let them know that you will remind them throughout the practice to reconnect with their anchor. As we take our relaxation journey, know that your anchor will keep you safe.

Guiding Participants through Relaxation Exercises

The two guided relaxation techniques used for the study are the body scan and progressive muscle relaxation. Suggested outlines and wording for these are provided on the following pages. However, you are not required to follow the exact outline or specific wording for the body scan and progressive muscle. Try to vary which relaxation exercise is used throughout the study classes. With these relaxation exercises, encourage participants to be aware of any physical sensations and feelings that may arise, but not to judge or criticize what they may feel.

Regularly remind them to return to their anchor by saying: *If your mind or body has disconnected from my voice, I invite you to return to your anchor. Imagine the person, place or object that makes you feel grounded, loved and filled with joy. Allow this sensation to radiate in a circular motion starting in the belly, getting bigger and bigger as it radiates out to the sides of your body, to the crown of your head and all the way down to your feet.*

Ending the Relaxation Exercise

At the end of the relaxation exercise, guide participants to bring their awareness back to the room, feeling the mat or chair beneath them and listening to the sounds around them. Have them start to wiggle their fingers and toes and blink their eyes. Instruct them to take their time coming up to a seated position. Let them know that if they would prefer to continue to lie down, they can stay where they are and come up when they are ready.

As the participants bring their awareness back to the room, remind them to return to any intention they set at the beginning of relaxation. Have them take a deep breath.

Before moving into closing, end relaxation with a statement to reinforce the day’s practice. Have the participants take a moment to give themselves thanks for taking the time to care for their mind and body today. Continually remind participants to use the tools they have learned through breathing, relaxation, and poses to help them outside of class in daily life.

© Boston Medical Center
Veterans Back to Health Trial

Guidelines for Teaching Yoga Relaxation Exercises

Final Resting Pose

Description: Lying supine on mat, palms facing upwards, feet hip-width apart.

How to teach: Lie flat on back with arms resting comfortably, slightly out from the sides of body, palms upward. Move legs out like making a snow angel and allow legs and feet to roll out to each side. Move shoulder blades in toward back opening and broadening chest. Release neck and gently move it from side to side.

Modifications:

1. If there is back discomfort, place bolster or rolled blanket(s) under knees to release pressure. Teachers may want to offer this option to everyone to start.
2. Place a folded blanket under the head like a pillow.
3. Seated: Sit against wall or on chair with spine supported and blanket over legs and/or with head resting on knees.
4. Side: Lying on side with support of blanket under head, under top arm and another supporting and/or between the knees.
5. Belly: Lying on belly, with feet slightly turned in toward each other and with hands or a blanket folded under head.

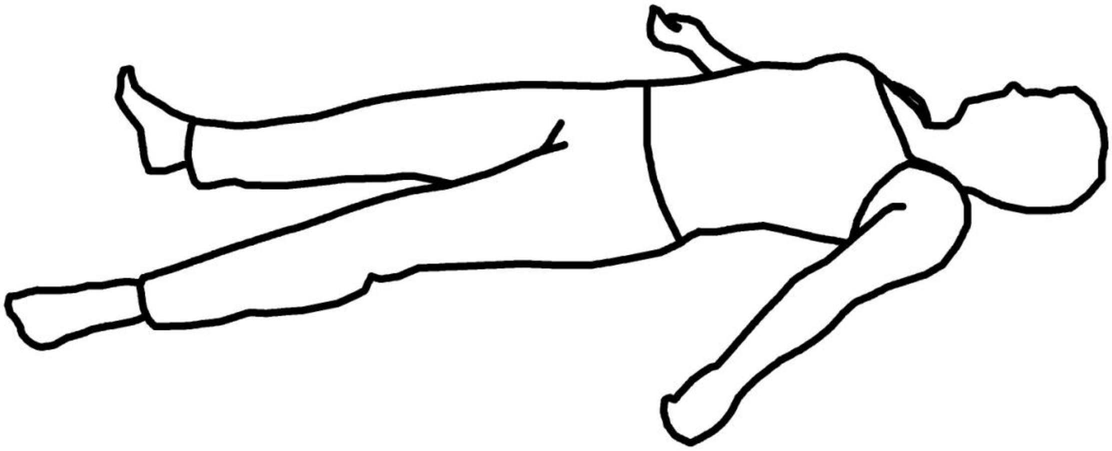

Final Resting Pose

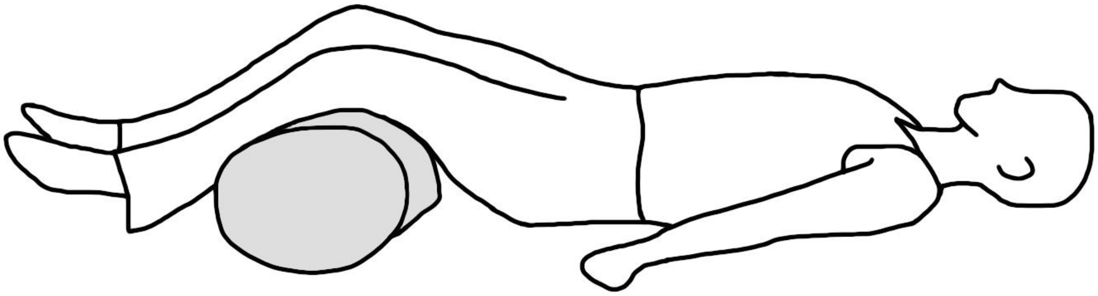

Supported Final Resting Pose

© Boston Medical Center
Veterans Back to Health Trial

Guidelines for Teaching Yoga Relaxation Exercises

Body Scan

- Begin by focusing on the breath, allowing your stomach to rise as you inhale and fall as you exhale. Become aware of normal breathing.
- Move the focus to the body. Become aware of any sensations, uncomfortable parts, and the temperature and feel of the body.
- Guide participants to relax all visible parts of the body starting with the feet and moving to the crown of the head. Start with the right and left toes, then feet, ankles, calves, knees, upper legs, hips, pelvis, tailbone, low back, abdomen, mid and upper back, chest, fingers, wrists, arms, shoulders, neck, jaw, mouth, nose, eyes, forehead, ears, and whole face.
- Notice any sensations without judgment. Know that all the sensations, positive, negative or neutral, are completely normal. These sensations are like waves in the ocean, rising and falling, sometimes feeling intense, yet they always pass and leave calm waters. Watching the rise and fall allows us to navigate in pure awareness.
- To end, start by wiggling toes and then fingers, slowly moving the body. Notice any changes in the body without judgment or resistance.

Progressive Muscle Relaxation

- Take a minute to notice the breath moving in and out.
- Shift attention to the body. Slowly tense the muscles in the right foot and toes, hold briefly, and then relax. Feel the tension flow away and notice how your foot feels as it becomes lax.
- Shift attention to the left foot and toes. Follow the same sequence of muscle tension and release.
- Move slowly up the body, contracting and relaxing the muscles as you go. The most popular sequence runs as follows:
    1. Right foot and toes
    2. Left foot and toes
    3. Right leg
    4. Left leg
    5. Hips and buttocks
    6. Stomach
    7. Right hand (make a fist)
    8. Right arm
    9. Left hand (make a fist)
    10. Left arm
    11. Neck and shoulders
    12. Face – jaw, mouth, lips, forehead, eyelids, entire face
- It may take some practice at first, but try not to tense muscles other than those intended. It is normal if this does not come naturally in the beginning.

© Boston Medical Center
Veterans Back to Health Trial

Poems and Readings

Week 1: Defining Yoga

Do yoga with no goal but
to be in the moment. This
breath, this stretch, this wave
of emotion rolling in. Watch it
crest, and break, then dissipate.

Hold the body like a
lover in a close embrace,
listening with intimacy,
touching with tenderness.

Yoga is a threshold into
mystery; each pose an
open doorway and an
invitation to unfold.

Sensations rise and fall,
and through it all the
deathless center radiates
the simple truth of union.

-Danna Faulds

from Go In and In: Poems From the Heart of Yoga

© Boston Medical Center
Veterans Back to Health Trial

Poems and Readings

Week 2: Self-Compassion

WALK SLOWLY

It only takes a reminder to breathe,
a moment to be still, and just like that,
something in you settles, softens, makes
space for imperfection. The harsh voice
of judgment drops to a whisper and you
remember again that life isn't a relay
race; that waking up to life is what we
were born for. As many times as you
forget, catch yourself charging forward,
that many times you can make the choice
to stop, to breathe, to be, and to walk
slowly into the mystery.

-Danna Faulds

from Go In and In: Poems From the Heart of Yoga

© Boston Medical Center
Veterans Back to Health Trial

Poems and Readings

Week 3: Awareness

CONSCIOUS BREATHING

There are a number of breathing techniques you can use to make life vivid and more enjoyable.
The first exercise is very simple. As you breathe in, you say to yourself, 'Breathing In, I know that I am breathing in.'
And as you breathe out say, 'Breathing out, I know that I am breathing out.'
Just that. You recognize your in-breath as an in-breath and your out-breath as an out-breath.
This technique can help you keep your mind on your breath.
As you practice, your breath will become peaceful and gentle, and your mind and body will also become peaceful and gentle.
Breathing in and out is very important, and it is enjoyable.
Our breathing is the link between our body and our mind.
By concentrating on our breathing, 'In' and 'Out,' we bring body and mind back together and become whole again.
Conscious breathing is an important bridge.

-Thich Nhat Hanh

from Peace is Every Step

© Boston Medical Center
Veterans Back to Health Trial

Poems and Readings

Week 4: Acceptance

LET IT GO

Let go of the ways you thought life would unfold:
the holding of plans or dreams or expectations - Let it all go.
Save your strength to swim with the tide.
The choice to fight what is here before you now will
only result in struggle, fear, and desperate attempts
to flee from the very energy you long for. Let go.

Let it all go and flow with the grace that washes
through your days whether you received it gently
or with all your quills raised to defend against invaders.
Take this on faith; the mind may never find the
explanations that it seeks, but you will move forward
nonetheless. Let go, and the wave's crest will carry
you to unknown shores, beyond your wildest dreams
or destinations. Let it all go and find the place of
rest and peace, and certain transformation.

-Danna Faulds

from Go In and In: Poems From the Heart of Yoga

© Boston Medical Center
Veterans Back to Health Trial

Poems and Readings

Week 5: Self-Control

Our true home is in the present moment.

To live in the present moment is a miracle.

The miracle is not to walk on water.

The miracle is to walk on the green Earth in the present moment,

to appreciate the peace and beauty that are available now.

Peace is all around us—

In the world and in nature-

And within us-

In our bodies and our spirits.

Once we learn to touch this peace,

We will be healed and transformed.

It is not a matter of faith;

It is a matter of practice.

-Thich Nhat Hanh

from Touching Peace: Practicing the Art of Mindful Living

© Boston Medical Center
Veterans Back to Health Trial

Poems and Readings

Week 6: Honesty

LOVE AFTER LOVE

The time will come
when, with elation,
you will greet yourself arriving
at your own door, in your own mirror,
and each will smile at the other's welcome,

and say, sit here. Eat.
You will love again the stranger who was yourself.
Give wine. Give bread. Give back your heart
to itself, to the stranger who has loved you

all your life, whom you ignored
for another, who knows you by heart.
Take down the love letters from the bookshelf,

the photographs, the desperate notes,
peel your own image from the mirror.
Sit. Feast on your life.

-Derek Walcott

© Boston Medical Center
Veterans Back to Health Trial

HAPPY LIFE

With all your heart say out loud, 'I want to live a happy life.'
Listen to what you just said.
Take notice of things.
Surround yourself with people you love.
Listen to the wind.
Imagine.
Let everything change all the time.
Let go of the why.
Welcome miracles.
Thank God constantly.
Breathe.
Tell the truth about how you feel.
Make choices.
Want what you want.
Let the animals reach you.
Have children teach you.
Take good care of your body.
Love passionately.
Share your dreams.
Spread your gifts.
Check it out.
Forgive the past.
Dive in.
Eat it up.
Take chances.
Be real.

-Julie Evans

© Boston Medical Center
Veterans Back to Health Trial

Poems and Readings

Week 8: Balance

LOOK TO THIS DAY

Look to this day:

For it is life, the very life of life.

In its brief course

Lie all the verities and realities of your existence.

The bliss of growth,

The glory of action,

The splendor of achievement

Are but experiences of time.

For yesterday is but a memory,

And tomorrow is only a vision;

But today well-lived, makes

Every yesterday a dream of happiness

And every tomorrow a vision of hope.

Look well therefore to this day;

Such is the salutation to the every-new dawn!

-Kālidāsa

© Boston Medical Center
Veterans Back to Health Trial

Poems and Readings

Week 9: Moderation

LOVE MEDITATION

May I be peaceful, happy, and light in body and spirit.
May I be safe and free from injury.
May I be free from anger, afflictions, fear, and anxiety.

-Thich Nhat Hanh

CALMING, SMILING, PRESENT MOMENT, WONDERFUL MOMENT

Breathing in, I calm my body. Breathing out, I smile.
Dwelling in the present moment I know exactly where I am;
I know this is a wonderful moment.

-Thich Nhat Hanh

© Boston Medical Center
Veterans Back to Health Trial

Poems and Readings

Week 10: Healthy Lifestyle

THE GUEST HOUSE

This being human is a guest house.
Every morning a new arrival.
A joy, a depression, a meanness,
some momentary awareness comes
as an unexpected visitor.
Welcome and entertain them all!
Even if they are a crowd of sorrows,
who violently sweep your house
empty of its furniture,
still, treat each guest honorably.
He may be clearing you out
for some new delight.
The dark thought, the shame, the malice.
Meet them at the door laughing and invite them in.
Be grateful for whatever comes.
Because each has been sent
as a guide from beyond.

-Jelaluddin Rumi,
translation by Coleman Barks

© Boston Medical Center
Veterans Back to Health Trial

Poems and Readings

Week 11: Compassion for Others

May I be well.

May I be happy.

May I be healed from suffering.

May I be at peace.

May we be well.

May we be happy.

May we be free from suffering.

May we be at peace.

May everyone be well.

May everyone be happy.

May everyone be healed from suffering.

May everyone be at peace.

-Ancient Quotation

© Boston Medical Center
Veterans Back to Health Trial

Poems and Readings

Week 12: Community

NAMASTE

I honor the place in you
in which the entire universe dwells.
I honor the place in you
which is of love, of truth,
of light and of peace.
When you are in that
place in you,
and I am in that place in me,
we are one.

-Traditional Yogic Salutation

© Boston Medical Center
Veterans Back to Health Trial

Bibliography

1. Deyo RA, Weinstein JN. Low back pain. N Engl J Med 2001;344:363-70.
2. Deyo RA, Mirza SK, Martin BI. Back pain prevalence and visit rates: estimates from U.S. national surveys, 2002. Spine 2006;31:2724-7.
3. Licciardone JC. The epidemiology and medical management of low back pain during ambulatory medical care visits in the United States. Osteopath Med Prim Care 2008;2:11.
4. Guo HR, Tanaka S, Halperin WE, Cameron LL. Back pain prevalence in US industry and estimates of lost workdays. Am J Public Health 1999;89:1029-35.
5. Andersson GB. Epidemiological features of chronic low-back pain. Lancet 1999;354:581-5.
6. Luo X, Pietrobon R, Sun SX, Liu GG, Hey L. Estimates and patterns of direct health care expenditures among individuals with back pain in the United States. Spine 2004;29:79-86.
7. Martin BI, Deyo RA, Mirza SK, et al. Expenditures and health status among adults with back and neck problems. JAMA 2008;299:656-64.
8. Dagenais S, Caro J, Haldeman S. A systematic review of low back pain cost of illness studies in the United States and internationally. Spine J 2008;8:8-20.
9. Gaskin DJ, Richard P. The economic costs of pain in the United States. J Pain 2012;13:715-24.
10. Engel GL. The need for a new medical model: a challenge for biomedicine. Science 1977;196:129-36.
11. Kerns RD. Transforming pain care in the United States Department of Veterans Affairs: the role of the VA Pain Research Network. Clin J Pain 2013;29:93-4.
12. Lew HL, Otis JD, Tun C, Kerns RD, Clark ME, Cifu DX. Prevalence of chronic pain, posttraumatic stress disorder, and persistent postconcussive symptoms in OIF/OEF veterans: polytrauma clinical triad. J Rehabil Res Dev 2009;46:697-702.
13. Institute of Medicine (US) Committee on Advancing Pain Research, Care, and Education. Relieving pain in America: a blueprint for transforming prevention, care, education, and research. Washington (DC): National Academies Press; 2011.
14. Haskell SG, Brandt CA, Krebs EE, Skanderson M, Kerns RD, Goulet JL. Pain among veterans of Operations Enduring Freedom and Iraqi Freedom: do women and men differ? Pain Med 2009;10:1167-73.
15. Kirsch B, Berdine H, Zablotsky D, et al. Management strategy: identifying pain as the fifth vital sign. VHSJ 2000:49-59.
16. Barlas FM, Higgins WB, Pflieger JS, Diecker K. 2011 Health Related Behaviors Survey of Active Duty Military Personnel. Arlington, VA: U.S. Department of Defense; 2013.
17. Seal KH, Cohen G, Waldrop A, Cohen BE, Maguen S, Ren L. Substance use disorders in Iraq and Afghanistan veterans in VA healthcare, 2001-2010: Implications for screening, diagnosis and treatment. Drug Alcohol Depend 2011;116:93-101.
18. Burstrom K, Johannesson M, Diderichsen F. Swedish population health-related quality of life results using the EQ-5D. Qual Life Res 2001;10:621-35.

© Boston Medical Center
Veterans Back to Health Trial

Bibliography

19. Kosinski MR, Schein JR, Vallow SM, et al. An observational study of health-related quality of life and pain outcomes in chronic low back pain patients treated with fentanyl transdermal system. Curr Med Res Opin 2005;21:849-62.
20. Cohen SP, Nguyen C, Kapoor SG, et al. Back pain during war: an analysis of factors affecting outcome. Arch Intern Med 2009;169:1916-23.
21. Lincoln AE, Smith GS, Amoroso PJ, Bell NS. The natural history and risk factors of musculoskeletal conditions resulting in disability among US Army personnel. Work 2002;18:99-113.
22. Galloway KT, Buckenmaier CC, III, Polomano RC. War on Pain: Understanding pain and pain mechanisms. Am Nurse Today 2011;6.
23. Otis JD, Keane TM, Kerns RD. An examination of the relationship between chronic pain and post-traumatic stress disorder. J Rehabil Res Dev 2003;40:397-405.
24. McWilliams LA, Cox BJ, Enns MW. Mood and anxiety disorders associated with chronic pain: an examination in a nationally representative sample. Pain 2003;106:127-33.
25. Kemp J, Bossarte R. Suicide Data Report, 2012. U.S. Department of Veterans Affairs;2012.
26. Marty M, Rozenberg S, Duplan B, Thomas P, Duquesnoy B, Allaert F. Quality of sleep in patients with chronic low back pain: a case-control study. Eur Spine J 2008;17:839-44.
27. Jensen MP, Romano JM, Turner JA, Good AB, Wald LH. Patient beliefs predict patient functioning: further support for a cognitive-behavioural model of chronic pain. Pain 1999;81:95-104.
28. Waddell G, Newton M, Henderson I, Somerville D, Main CJ. A Fear-Avoidance Beliefs Questionnaire (FABQ) and the role of fear-avoidance beliefs in chronic low back pain and disability. Pain 1993;52:157-68.
29. Morasco BJ, Corson K, Turk DC, Dobscha SK. Association between substance use disorder status and pain-related function following 12 months of treatment in primary care patients with musculoskeletal pain. J Pain 2011;12:352-9.
30. Nampiaparampil DE. Prevalence of chronic pain after traumatic brain injury: a systematic review. JAMA 2008;300:711-9.
31. Kar N. Cognitive behavioral therapy for the treatment of post-traumatic stress disorder: a review. Neuropsychiatr Dis Treat 2011;7:167-81.
32. Haskell SG, Ning Y, Krebs E, et al. Prevalence of painful musculoskeletal conditions in female and male veterans in 7 years after return from deployment in Operation Enduring Freedom/Operation Iraqi Freedom. Clin J Pain 2012;28:163-7.
33. Mernoff ST, Correia S. Military blast injury in Iraq and Afghanistan: the Veterans Health Administration's polytrauma system of care. Med Health R I 2010;93:16-8, 21.
34. Cifu DX, Taylor BC, Carne WF, et al. Traumatic brain injury, posttraumatic stress disorder, and pain diagnoses in OIF/OEF/OND Veterans. J Rehabil Res Dev 2013;50:1169-76.

© Boston Medical Center
Veterans Back to Health Trial

Bibliography

35. National Institute of Mental Health. Post-Traumatic Stress Disorder (PTSD). 2015.

36. Maguen S, Litz B. Moral injury in veterans of war. PTSD Research Quarterly 2012;23:1-6.

37. Litz BT, Stein N, Delaney E, et al. Moral injury and moral repair in war veterans: a preliminary model and intervention strategy. Clin Psychol Rev 2009;29:695-706.

38. McKee AC, Robinson ME. Military-related traumatic brain injury and neurodegeneration. Alzheimers Dement 2014;10:S242-53.

39. Dougherty AL, MacGregor AJ, Han PP, Viirre E, Heltemes KJ, Galarneau MR. Blast-related ear injuries among U.S. military personnel. J Rehabil Res Dev 2013;50:893-904.

40. Chou R, Huffman LH. Nonpharmacologic therapies for acute and chronic low back pain: a review of the evidence for an American Pain Society/American College of Physicians clinical practice guideline. Ann Intern Med 2007;147:492-504.

41. Kroesen K, Baldwin CM, Brooks AJ, Bell IR. US military veterans' perceptions of the conventional medical care system and their use of complementary and alternative medicine. Fam Pract 2002;19:57-64.

42. Office of The Army Surgeon General Pain Management Task Force. Providing a standardized DoD and VHA vision and approach to pain management to optimize the care for warriors and their families. 2010.

43. Saper RB, Eisenberg DM, Davis RB, Culpepper L, Phillips RS. Prevalence and patterns of adult yoga use in the United States: results of a national survey. Altern Ther Health Med 2004;10:44-9.

44. Yoga Journal. New Study Finds More Than 20 Million Yogis in U.S. 2012. Available from: http://www.yogajournal.com/uncategorized/new-study-finds-20-million-yogis-u-s/

45. Sherman KJ, Cherkin DC, Erro J, Miglioretti DL, Deyo RA. Comparing yoga, exercise, and a self-care book for chronic low back pain: a randomized, controlled trial. Ann Intern Med 2005;143:849-56.

46. Williams K, Abildso C, Steinberg L, et al. Evaluation of the effectiveness and efficacy of Iyengar yoga therapy on chronic low back pain. Spine 2009;34:2066-76.

47. Sherman KJ, Cherkin DC, Wellman RD, et al. A randomized trial comparing yoga, stretching, and a self-care book for chronic low back pain. Arch Intern Med 2011;171:2019-26.

48. Tilbrook HE, Cox H, Hewitt CE, et al. Yoga for chronic low back pain: a randomized trial. Ann Intern Med 2011;155:569-78.

49. Saper RB, Boah AR, Keosaian J, Cerrada C, Weinberg J, Sherman KJ. Comparing once- versus twice-weekly yoga classes for chronic low back pain in predominantly low income minorities: a randomized dosing trial. Evid Based Complement Alternat Med 2013:658030.

50. Galantino ML, Bzdewka TM, Eissler-Russo JL, et al. The impact of modified Hatha yoga on chronic low back pain: a pilot study. Altern Ther Health Med 2004;10:56-9.

© Boston Medical Center
Veterans Back to Health Trial

Bibliography

51. Williams KA, Petronis J, Smith D, et al. Effect of Iyengar yoga therapy for chronic low back pain. Pain 2005;115:107-17.

52. Saper RB, Sherman KJ, Cullum-Dugan D, Davis RB, Phillips RS, Culpepper L. Yoga for chronic low back pain in a predominantly minority population: a pilot randomized controlled trial. Altern Ther Health Med 2009;15:18-27.

53. Cox H, Tilbrook H, Aplin J, et al. A randomised controlled trial of yoga for the treatment of chronic low back pain: results of a pilot study. Complement Ther Clin Pract 2010;16:187-93.

54. Tekur P, Nagarathna R, Chametcha S, Hankey A, Nagendra HR. A comprehensive yoga programs improves pain, anxiety and depression in chronic low back pain patients more than exercise: An RCT. Complement Ther Med 2012;20:107-18.

55. Posadzki P, Ernst E. Yoga for low back pain: a systematic review of randomized clinical trials. Clin Rheumatol 2011;30:1257-62.

56. Cramer H, Lauche R, Haller H, Dobos G. A Systematic Review and Meta-analysis of Yoga for Low Back Pain. Clin J Pain 2013;29:450-60.

57. Sherman KJ, Wellman RD, Cook AJ, Cherkin DC, Ceballos RM. Mediators of yoga and stretching for chronic low back pain. Evid Based Complement Alternat Med 2013;130818.

58. Balasubramaniam M, Telles S, Doraiswamy PM. Yoga on our minds: a systematic review of yoga for neuropsychiatric disorders. Front Psychiatry 2013;3:117.

59. Janakiramaiah N, Gangadhar BN, Naga Venkatesha Murthy PJ, Harish MG, Subbakrishna DK, Vedamurthachar A. Antidepressant efficacy of Sudarshan Kriya Yoga (SKY) in melancholia: a randomized comparison with electroconvulsive therapy (ECT) and imipramine. J Affect Disord 2000;57:255-9.

60. Kinser PA, Bourguignon C, Whaley D, Hauenstein E, Taylor AG. Feasibility, acceptability, and effects of gentle hatha yoga for women with major depression: findings from a randomized controlled mixed-methods study. Arch Psychiatr Nurs 2013;27:137-47.

61. Michalsen A, Jeitler M, Brunnhuber S, et al. Iyengar yoga for distressed women: a 3-armed randomized controlled trial. Evid Based Complement Alternat Med 2012;408727.

62. Pilkington K, Kirkwood G, Rampes H, Richardson J. Yoga for depression: The research evidence. J Affect Disord 2005;89:13-24.

63. Saeed SA, Antonacci DJ, Bloch RM. Exercise, yoga, and meditation for depressive and anxiety disorders. Am Fam Physician 2010;81:981-6.

64. Uebelacker LA, Epstein-Lubow G, Gaudiano BA, Tremont G, Battle CL, Miller IW. Hatha yoga for depression: critical review of the evidence for efficacy, plausible mechanisms of action, and directions for future research. J Psychiatr Pract 2010;16:22-33.

65. Woolery A, Myers H, Sternlieb B, Zeltzer L. A yoga intervention for young adults with elevated symptoms of depression. Altern Ther Health Med 2004;10:60-3.

© Boston Medical Center
Veterans Back to Health Trial

Bibliography

66. Streeter CC, Whitfield TH, Owen L, et al. Effects of yoga versus walking on mood, anxiety, and brain GABA levels: a randomized controlled MRS study. J Altern Complement Med 2010;16:1145-52.

67. Staples JK, Hamilton MF, Uddo M. A yoga program for the symptoms of post-traumatic stress disorder in veterans. Mil Med 2013;178:854-60.

68. Descilo T, Vedamurtachar A, Gerbarg PL, et al. Effects of a yoga breath intervention alone and in combination with an exposure therapy for post-traumatic stress disorder and depression in survivors of the 2004 South-East Asia tsunami. Acta Psychiatr Scand 2010;121:289-300.

69. Kohn M, Persson LU, Bryngelsson IL, Anderzen-Carlsson A, Westerdahl E. Medical yoga for patients with stress-related symptoms and diagnoses in primary health care: a randomized controlled trial. Evid Based Complement Alternat Med 2013;215348.

70. Michalsen A, Grossman P, Acil A, et al. Rapid stress reduction and anxiolysis among distressed women as a consequence of a three-month intensive yoga program. Med Sci Monit 2005;11:CR555-61.

71. Vollestad J, Nielsen MB, Nielsen GH. Mindfulness- and acceptance-based interventions for anxiety disorders: a systematic review and meta-analysis. Br J Clin Psychol 2012;51:239-60.

72. Afonso RF, Hachul H, Kozasa EH, et al. Yoga decreases insomnia in postmenopausal women: a randomized clinical trial. Menopause 2012;19:186-93.

73. Khalsa SB. Treatment of chronic insomnia with yoga: a preliminary study with sleep-wake diaries. Appl Psychophysiol Biofeedback 2004;29:269-78.

74. Evans S, Moieni M, Subramanian S, Tsao JC, Sternlieb B, Zeltzer LK. "Now I see a brighter day": expectations and perceived benefits of an Iyengar yoga intervention for young patients with rheumatoid arthritis. J Yoga Phys Ther 2011;1.

75. Veterans Integrated Service Network. VA Healthcare VISN 4 Strategic Plan 2013-2018. 2013.

76. Gaudet TW. VA Office Developing Innovative Patient-Centered Model of Care for Veterans. VA Office of Public and Intergovernmental Affairs 2011. Available from: http://www.va.gov/opa/pressrel/pressrelease.cfm?id=2034

77. Veterans Health Administration. Patient Centered Care. 2013. Available from: http://www.va.gov/health/newsfeatures/20120827a.asp

78. Groessl EJ, Weingart KR, Aschbacher K, Pada L, Baxi S. Yoga for veterans with chronic low-back pain. J Altern Complement Med 2008;14:1123-9.

79. Groessl EJ, Weingart KR, Johnson N, Baxi S. The benefits of yoga for women veterans with chronic low back pain. J Altern Complement Med 2012;18:832-8.

80. Armed Forces Health Surveillance Center (AFHSC). Ambulatory visits among members of the active component, U.S. Armed Forces, 2012. MSMR 2013;20:18-23.

81. Emerson D, Sharma R, Chaudhry S, Turner J. Trauma-sensitive yoga: principles, practice, and research. Int J Yoga Therap 2014;19:123-8.

© Boston Medical Center
Veterans Back to Health Trial

Appendices

Supplemental Poems and Readings 96
Yoga Teacher Notes Form 104
Yoga Home Practice Log 105

© Boston Medical Center
Veterans Back to Health Trial

Appendix 1: Supplemental Poems and Readings

GRATITUDE

Gratitude unlocks the fullness of life.

It turns what we have into enough and more.

It turns denial into acceptance,

Chaos to order, confusion to clarity.

It can turn a meal into a feast,

A house into a home,

A stranger into a friend.

Gratitude makes sense of our past,

Brings peace for today,

And creates a vision for tomorrow.

-John O'Donohue

© Boston Medical Center
Veterans Back to Health Trial

Appendix 1: Supplemental Poems and Readings

WITHIN THE BODY YOU ARE WEARING

Within the body you are wearing, now
inside the bones and beating in the heart,
lives the one you have been searching for so long.
But you must stop running away and shake hands,
the meeting doesn't happen without your
presence . . . your participation.
The same one waiting for you there
is moving in the trees, glistening on the water,
growing in the grasses and lurking in the shadows you create.
You have nowhere to go.
The marriage happened long ago.
Behold your mate.

-Robert Hall

© Boston Medical Center
Veterans Back to Health Trial

Appendix 1: Supplemental Poems and Readings

Breathing in, I know I'm breathing in.

Breathing out, I know

As the in-breath grows deep,

The out-breath grows slow.

Breathing in makes me calm.

Breathing out makes me ease.

With the in-breath, I smile.

With the out-breath, I release.

Breathing in, there is only the present moment.

Breathing out, it is a wonderful moment.

-Thich Nhat Hanh

© Boston Medical Center
Veterans Back to Health Trial

Appendix 1: Supplemental Poems and Readings

FROM ROOT TO BLOOM

Sap is flowing once again from root to trunk,
from trunk to branch,
to every leaf
and bud
and blossom
on the tree.
The frozen days are past.
Now is the season of flowering,
when the full force of energy moves up
and up
to manifest its destiny.
It is everything to me,
this new life springing forth
from ash and compost,
this chance to nurture,
weed,
and prune,
to patiently receive the miracle that blooms
as if no force on earth
can turn aside its beauty.

-Danna Faulds
From Root to Bloom: Yoga Poems and Other Writings

© Boston Medical Center
Veterans Back to Health Trial

Appendix 1: Supplemental Poems and Readings

AWARENESS KNOWING ITSELF

Settle in the here and now.
Reach down into the center
where the world is not spinning
and drink this holy peace.

Feel relief flood into every
cell. Nothing to do. Nothing
to be but what you are already.
Nothing to receive but what
flows effortlessly from the
mystery into form.

Nothing to run from or run
toward. Just this breath,
awareness knowing itself as
embodiment. Just this breath,
awareness waking up to truth.

-Danna Faulds

© Boston Medical Center
Veterans Back to Health Trial

Appendix 1: Supplemental Poems and Readings

YOGA'S REACH

It is a wonder
how a simple stretch
deepens breath,
and an elegantly held pose
grows to touch the
whole of me.

Like sugar stirred into tea,
the potency
of yoga spreads
from body into mind
and heart, revealing
an ocean of energy
that heals and opens,

holds me close
and sets me free
all in the same moment.

-Danna Faulds

© Boston Medical Center
Veterans Back to Health Trial

Appendix 1: Supplemental Poems and Readings

CIRCLE OF BREATH

The night my father died
the moon shone on the snow.
I drove in from the west;
mother was at the door.
All the light in the room extended
like a shadow. Truant from knowing,
I stood where the great dark fell.

There was a time before, something
we used to tell--how we parked
the car in a storm and walked into
the field to know how it was to be
cut off, out in the dark alone.
My father and I stood together
while the storm went by.

A windmill was there in the field
giving its little cry, while we
stood calm in ourselves, knowing
we could go home.
But I stood on the skull of the world
the night he died, and knew that
I leased a place to live
with my white breath.
Truant no more, I stepped forward
and learned his death.

-William Stafford

© Boston Medical Center
Veterans Back to Health Trial

Appendix 1: Supplemental Poems and Readings

BUOYANCY

Love has taken away my practices and filled me with poetry.
I tried to keep quietly repeating, “No strength but yours,” but I couldn't.
I had to clap and sing.
I used to be respectable and chaste and stable,
but who can stand in this strong wind and remember those things?
A mountain keeps an echo deep inside itself.
That's how I hold your voice.
I am scrap wood thrown in your fire, and quickly reduced to smoke.
I saw you and became empty.
This emptiness, more beautiful than existence, it obliterates existence, and yet when it comes,
existence thrives and creates more existence!
The sky is blue. The world is a blind man squatting on the road.
But whoever sees your emptiness sees beyond blue and beyond the blind man.
A great soul hides moving through a crowd in a city where no one knows him.
To praise is to praise how one surrenders to the emptiness.
To praise the sun is to praise your own eyes.
Praise, the ocean. What we say, a little ship.
So the sea-journey goes on, and who knows where!
Just to be held by the ocean is the best luck we could have. It's a total waking up!
Why should we grieve that we've been sleeping?
It doesn't matter how long we've been unconscious.
We're groggy, but let the guilt go.
Feel the motions of tenderness around you, the buoyancy.

-Rumi, 13th Century Persian mystic

© Boston Medical Center
Veterans Back to Health Trial

Appendix 2: Yoga Teacher Notes Form

Yoga Teacher Class Notes

Please use the space below to record any notes, observations, or concerns from today's class.

Teacher Name: ______________________ **Date:** ____________

Observations: ______________________________________

Comments about the yoga protocol? ______________________

Other: ______________________________________

If there are any Adverse Events that came up during class or more supplies are needed, please call study staff *immediately*.

© Boston Medical Center
Veterans Back to Health Trial

Appendix 3: Yoga Home Practice Log

Weekly Home Practice Log

We suggest that you practice yoga for 30 minutes on days you do not have class. It is okay if you are unable to practice every day. We just ask that you are honest when filling out this log.

Please record when and for how long you did yoga at home, starting from your last class.
Please do not include yoga practice done during class on this log.

	Date:__/__/__	Date:__/__/__	Date:__/__/__	Date:__/__/__	Date:__/__/__	Date:__/__/__	Date:__/__/__
Have you practiced yoga at home today?	☐ Yes ☐ No	☐ Yes ☐ No	☐ Yes ☐ No	☐ Yes ☐ No	☐ Yes ☐ No	☐ Yes ☐ No	☐ Yes ☐ No
Minutes:	_______ min.	_______ min.	_______ min.	_______ min.	_______ min.	_______ min.	_______ min.
Comments:							

Please tell a yoga teacher or a research staff member if you experience any injury during the course of the study. You can call research staff at **781-687-2000 ext. 5520**.

Version 1.0

STAFF USE ONLY

COLLECTION		DATA ENTRY	
Initials: __________	Date: ________	Initials: __________	Date: ________

© Boston Medical Center
Veterans Back to Health Trial
