# Supplementary material for: Yoga versus education for Veterans with chronic low back pain: study protocol for a randomized controlled trial
Source: Trials. 2016 Apr 29;17:224. doi: 10.1186/s13063-016-1321-5 (PMC4850721; doi:10.1186/s13063-016-1321-5)
Supplement: Additional file 7: — Manual of Procedures. Comprehensive manual of standard operating procedures for recruitment, enrollment, data collection/management, interventions, qualitative interviews, and adverse events. (PDF 5.66 mb) [file 13063_2016_1321_MOESM7_ESM.pdf]

MANUAL OF PROCEDURES

© Boston Medical Center
Veterans Back to Health Trial

Table of Contents

Introduction

Study Overview 1.1
Study Organization 1.2
Overall Study Flow Diagram 1.4

Recruitment, Screening, and Enrollment

Recruitment Overview 2.1
Recruitment Flow Sheet 2.2
Detailed Screening Procedures 2.3
Specific Recruitment Methods 2.7
Challenging Situations Procedure 2.10
Information Sessions Procedure 2.11
Informed Consent Progress Note 2.14
Monitoring of Recruitment Progress 2.15
Eligibility Verification Checklist 2.16

Yoga Intervention

Yoga Overview 3.1
Yoga Flow Sheet 3.2
Yoga Randomization Script 3.3
Detailed Yoga Procedures 3.4
Week by Week Yoga Tasks 3.6
Yoga Class Reminder Script 3.7
Yoga Home Practice 3.8

Education Intervention

Education Overview 4.1
Education Flow Sheet 4.2
Education Randomization Script 4.3
Detailed Education Procedures 4.4
Week by Week Education Tasks 4.6
Education Check-In Script 4.7
Education Check-In Questions 4.8
The *Back Pain Helpbook* Curriculum 4.10
Education Newsletter Volume 1 .... 4.17
Education Newsletter Volume 2 .... 4.18

© Boston Medical Center

Table of Contents

Education Newsletter Volume 3 .... 4.21
Education Newsletter Volume 4 .... 4.22

<u>Qualitative Interviews</u>

Qualitative Overview 5.1
Qualitative Flow Sheet 5.2
Qualitative Recruitment Script 5.3
Interview Information Sheet 5.4
Detailed Qualitative Procedures 5.5
Qualitative Interview Guides 5.7
Interview Progress Note 5.15
Qualitative Honoraria Form 5.16
Drop-Out Procedures 5.17

<u>Data Collection and Management</u>

Data Overview 6.1
Data Flow Sheet 6.2
Survey Administration Procedures 6.3
Survey Signature Page 6.8
Data Collection Schedule 6.9
Honoraria Distribution 6.10
Randomization Procedure 6.11
Randomization and Enrollment Form 6.12
Data Entry Procedures 6.13
StudyTRAX Guidelines 6.17
Data Management Procedures 6.20
Study Completion Form 6.23

<u>Adverse Events</u>

Adverse Events Overview 7.1
Documentation and Reporting 7.2
Procedure Diagrams 7.4
Data and Safety Monitoring 7.6

© Boston Medical Center

Introduction to Manual of Procedures

1.0

© Boston Medical Center
Veterans Back to Health Trial

Study Overview

Veterans Back to Health is a 24-week randomized controlled trial (RCT) comparing weekly hatha yoga classes supplemented by home yoga practice vs. education using a comprehensive self-care book for 120 Veterans with chronic low back pain (cLBP).

The intervention period is 12 weeks followed by a 12-week follow-up period. During the 12-week follow-up period, yoga participants will be encouraged to continue home practice and education participants will be encouraged to continue following recommendations from the book. This study has four specific aims:

1. Determine the effectiveness of a structured reproducible 12-week series of hatha yoga classes, supplemented with home practice, compared to education, for decreasing pain intensity and improving back-related function.
2. Evaluate the effectiveness of yoga compared to education for reducing comorbid post-traumatic stress symptoms and other psychosocial outcomes, including depression, traumatic brain injury, anxiety, self-efficacy, coping, and family functioning.
3. Evaluate the cost-effectiveness of yoga for cLBP at 12 weeks and 6 months from three perspectives: society, the Veterans Health Administration, and the Veteran.
4. Explore the effect of cLBP on family relationships, as well as yoga's impact on the Veteran, family, and family functioning through interviews with 20 Veteran yoga participants and their partners.

The purpose of this manual is to serve as a guide through the various phases of this study. The standardized procedures will help to ensure that research members who carry out this research study are utilizing the most effective and appropriate approaches in administering and facilitating the various aspects of the study.

Specifically, this manual provides scripts, forms, and an explanation of detailed procedures for all phases of the study. These phases include recruitment and eligibility screenings, informed consent, data collection and management, treatment arms, qualitative interviews, and adverse events.

The detailed study protocol, detailed staff information, delegation log, and other regulatory documents can be found in the Regulatory Binder, kept and updated by the BMC Research Project Manager.

© Boston Medical Center
Veterans Back to Health Trial

Study Organization

Study Sites

Boston University Medical Campus (BUMC), which includes Boston Medical Center (BMC), is the main site for this study.

The Edith Nourse Rogers Memorial Veterans (Bedford VA) Hospital will serve as the site for which recruitment, enrollment, interventions, and data collection procedures will take place.

The Boston University School of Social Work (BUSSW) will be conducting all aspects of the qualitative portion of this study.

Roles and Responsibilities

The Principal Investigator holds primary responsibility for ensuring ethical conduct of the study, including:

- Protecting human subjects' rights, safety and welfare
- Protocol compliance
- Adherence to institutional, state, and federal regulations and guidance
- Ensuring informed consent is appropriately obtained from each participant and study records are appropriately maintained
- Compliance with the financial and administrative policies and regulations, overall fiscal management of the project, and conflict of interest disclosure
- Oversee protocol design, recruitment, data collection, analysis and interpretation of results
- Ensuring that all research team members have appropriate education, training and qualifications to assume delegated study tests

The VA Site PI is responsible for ensuring the study conduct at the VA is in compliance with the study protocol and this manual.

Project Managers at BUMC and the VA will maintain regular contact and oversee daily study activities, such as:

- Maintaining IRB protocol, including submitting protocol amendments, continuing reviews, and safety reports to the IRB for review
- Maintaining regulatory binder(s) including the delegation of authority log for personnel
- Working with yoga teachers to ensure compliance with protocol-specified guidelines
- Maintaining financial documentation and assists PI with overall fiscal management
- Managing participant enrollment and ensure compliance with the protocol and other applicable regulations, including but not limited to:
    - Participant recruitment & eligibility screening
    - Obtaining informed consent

© Boston Medical Center
Veterans Back to Health Trial

- Facilitating subject retention and follow-up per protocol

- Reporting serious adverse events and/or unexpected problems as appropriate
- Overall data management for the study including maintaining electronic database(s)

All study team members are responsible for ensuring that the conduct of the study is compliant with the study protocol.

More detailed information about research staff, delegation of authority, and responsibilities can be found in the Regulatory Binder.

The research team meets regularly in order to maintain communication and properly monitor study progress. The table below shows the frequency and attendees for these regular meetings.

Attendees	Frequency	Purpose
PI & VA Site PI<br>Project Managers<br>Other Research Staff	Weekly	Assess recruitment and data collection progress, treatment adherence, and refine team strategies and procedures as necessary
DSMB<br>PI & VA Site PI	Biyearly	Review adverse events, enrollment progress, and data collection
PI & VA Site PI<br>Project Managers<br>Yoga Teachers	Monthly	Discuss challenges and successes, address protocol compliance, and discuss staffing needs
Entire Team	Yearly	Provide entre research team with update on study progress including recruitment and treatment

© Boston Medical Center
Veterans Back to Health Trial

Overall Study Flow Diagram

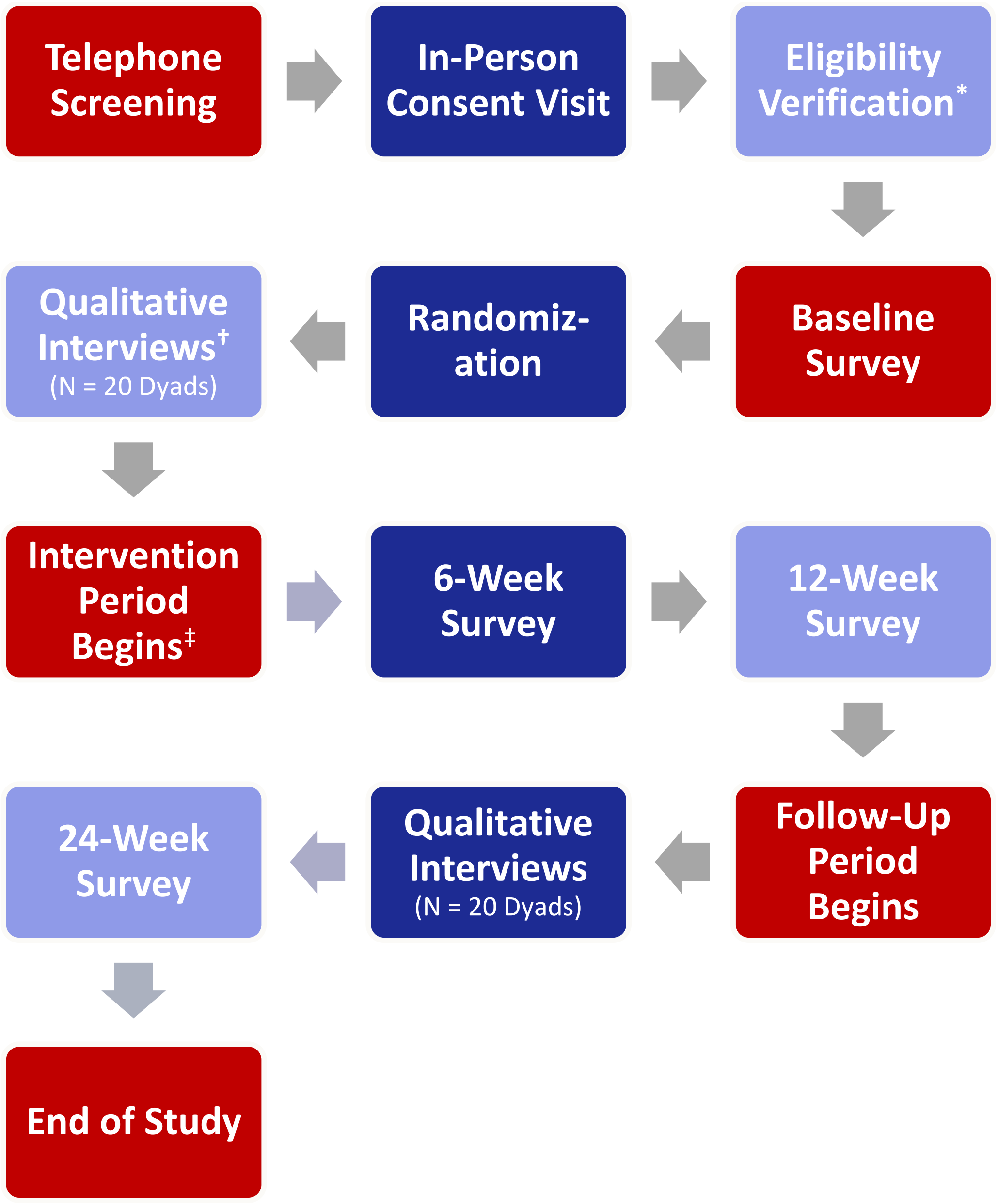

*Eligibility will be verified *only* if more than 45 days have elapsed between since screening and the baseline survey.

†Qualitative interviews may take place during the initial weeks of the intervention period if needed to achieve qualitative aims.

‡Interventions will begin within two weeks of the baseline survey.

© Boston Medical Center
Veterans Back to Health Trial

Recruitment and Enrollment Procedures

2.0

© Boston Medical Center

Recruitment Overview

Male and female Veterans age 18 years or older with chronic low back pain (cLBP) will be recruited using a tiered recruitment strategy to initially target OEF/OIF/OND Veterans. The study will be publicized as an unbiased comparison of two helpful treatments for cLBP. We will use an aggressive tiered recruitment strategy:

1. Identify all Veterans within Bedford VAMC seen in the previous two years that have back pain on their problem list. Mail letters signed by site champions to these Veterans.
2. Designate “study champions” within different Bedford VA clinical areas to publicize and encourage recruitment within their area.
3. Place of study flyers and brochures in exam rooms, waiting rooms, nursing stations throughout the Bedford VAMC and community Veteran organizations.
4. Use the Veteran network established by There and Back Again (TABA), a nonprofit organization offering mind-body approaches to help Veterans of all branches of the U.S. Armed Forces.
5. Use the network of National Guard and Reserve contacts established by the co-investigator at the BU School of Social Work through previous research.

We will employ a PDSA (Plan–Do–Study–Act) quality improvement process to weekly monitor and improve recruitment progress. All recruitment procedures will be in accordance with HIPAA guidelines and VHA policies.

The screening and enrollment process involves three parts:

1. <u>Verbal consent and telephone eligibility screening</u>. Eligibility screening takes place over the telephone by research staff using a questionnaire. The staff member asks for verbal consent before screening.
2. <u>In-person informational meeting with research staff</u>. If an individual appears to be eligible after the telephone screening, he/she is asked to meet with research staff for an information session at the Bedford VA. The session includes a short presentation about the study and the treatments, followed by a Q&A session.
3. <u>Administration of informed consent and signing written informed consent form (ICF)</u>. After the presentation, interested individuals are given time to review the ICF with a staff member. For those interested in joining the study, informed consent is obtained verbally and by signing the ICF. The participant is given a copy of the ICF to keep.

© Boston Medical Center
Veterans Back to Health Trial

Recruitment Flow Sheet

Events	Relevant Documents and Forms
**Recruitment Activities**	• Recruitment Spreadsheet
↓	
**Telephone Eligibility Screening**	• Verbal Consent*<br>• Detailed Screening Procedures<br>• Eligibility Screening Questionnaire*
↓	
**In-Person Consent Meeting**	• Information Session Reminder Call Script<br>• Informed Consent Progress Note[†]<br>• Written Informed Consent Form
↓	
**Eligibility Verification**	• Eligibility Verification Checklist*
↓	
**Baseline and Randomization[‡]**	• Baseline Survey Forms<br>• Randomization and Enrollment Form

START OF STUDY

* Form is filled out on StudyTRAX only.
[†] Form is filled out on paper (to be filed in participant file) and on StudyTRAX.
[‡] This event is discuss in detail in another section of this manual.

© Boston Medical Center
Veterans Back to Health Trial

Detailed Screening Procedures

TELEPHONE SCREENING PART ONE: ON THE PHONE

STEP 1: ANSWERING THE PHONE

Be ready by having the materials ready while by a phone:

- Open and login to StudyTRAX website (my.studytrax.com)
- Recruitment spreadsheet
- Outlook calendar (to schedule info session, if applicable)

Script: *Hello, thanks for calling Back to Health, this is* [name]. (Wait for response.)

If this is a Veteran calling to learn about the study, IMMEDIATELY record their name, contact information, and how they found out about the study (e.g., flyer, mailing, etc.).

Then given them some more information about the study:

This is a study that is comparing yoga vs. education (or self-management) to figure out how well yoga works for chronic lower back pain. This is a randomized study, which means that everyone that decides to join is randomly assigned to the yoga or self-management groups, like flipping a coin. There is a 50/50 chance of being put in either group. The study lasts for about 6 months and you will be paid to participate and travel to the VA. **[Pause for questions if needed]**

Let me tell you a little more about the groups. Yoga is a series of stretches and gentle exercises that take place in a class once per week at the Bedford VA for 12 weeks. Classes are 75 minutes long and usually contain 5-10 Veterans with 2 yoga instructors. **[Pause for questions if needed]**

The education or self-management group received a book called "The Back Pain Helpbook" that walks you through managing lower back pain on your own in daily life. It includes sections on getting to know your spine, pain management options (including yoga, medications, etc.), and stretching/strengthening your core and back. **[Pause for questions if needed]**

To find out if you are eligible for the study, we can do a quick screening questionnaire over the phone and if you are eligible, we will ask that you meet us at the Bedford VA to go over more information and sign a consent form. Do you have any questions? Does this sound like something you may be interested in?

> **IF INTERESTED:** *Great! The screening takes about 5-10 minutes. Is this a good time to do the questionnaire?*
>
> > **IF YES:** *Great! Let me pull it up. One moment please.*
> >
> > > [SKIP to Step 2: Collecting Contact Information (below)]
> >
> > **IF NO:** *When would be a good time for us to call you back for the screening?*
> >
> > > [ACTION: Verify contact information and make note of time and date to call back in the recruitment spreadsheet and highlight in white]
> > >
> > > **After choosing call-back time:** *Thank you, we will speak with you on* [day] *at* [time] *have a nice day!* [HANG UP]

© Boston Medical Center
Veterans Back to Health Trial

IF NOT INTERESTED: *Okay, thank you for taking the time to talk to me today.* [If no reason given…] *We try to keep track of how many people we talk to about the study, so may I ask why you are not interested in participating?* [ACTION: RECORD REASON in the recruitment spreadsheet – time, travel, no LBP, unwilling to be randomized, etc.]

IF NO (try to avoid this): *That's okay, thank you for your time, have a nice day!* [HANG UP]

STEP 2: VERBAL CONSENT

1. Determine the next Screening ID # you will use.
2. Click "New Subject" in StudyTRAX and enter the SID into the *Reference ID* box. Hit Enter on the keyboard.
3. The screen should say **Not in Project**. Click **Screen Subject** and select the appropriate Cohort number.
4. Click on the icon next to "Screening" and complete the Verbal Consent by following the script on screen. Select "Yes" or "No" as appropriate.
5. **SAVE** before leaving this screen.

STEP 3: ELIGIBILITY SCREENING

If the participant gives consent, move on to the Eligibility Screening questionnaire. Click on "Eligibility Screening" on the left side of the screen under the Table of Contents.

- Follow prompts on screen. Even if the individual gives an answer that you know will make them ineligible, complete the questionnaire fully.
- If they answer "I don't know," ask them politely to take some time to think about it. If they truly don't know, then choose 'DK/REF' (Don't Know or Refuse).
  - A numerical response must be entered for age – if the individual does not give an age, input '-88' in this field.
- When the questionnaire is complete, Screening Status will give the result by saying **Passed Phone Screen** or **Failed Phone Screen**. If you reach the end of the screening and the Screening Status is blank, go back and look for questions that were skipped.
- **SAVE OFTEN**. It may take a few moments to save and refresh, so you can say, "*One moment as I load the page.*"
  - It is recommended to **SAVE** immediately after the questionnaire is complete. While the page refreshes, you can say, *"That is the end of the screening questionnaire. Please wait while my computer evaluates your answers."*
  - After the questionnaire is complete and saved, the circle next to "Eligibility Screening" under the Table of contents on the left should be green. If it is yellow, there are questions left blank.

© Boston Medical Center
Veterans Back to Health Trial

Follow the scripts on screen to tell the participant their screening status:

IF ELIGIBLE: *Thank you for answering our questions. Based on your answers, you may be eligible for the research study. I have a couple additional questions to ask you then I will explain the next step in our screening process.* [ACTION: Ask Follow-Up Questions]

IF INELIGIBLE: *Unfortunately, according to our study guidelines, you are not eligible for this research study. I want to thank you for your time and interest in the study. Good-bye.* [HANG UP]

- If participant asks why he/she is ineligible, say, "*I'm sorry, I am not trained to interpret the results of the questionnaire*."
- If participant becomes upset, refer to the Challenging Situations procedure.

STEP 4: SCHEDULE IN-PERSON VISIT

Continue reading the script on screen for individuals that have passed the screening questionnaire:

Okay, now there is one more step you need to complete before you can enroll in the study. I'd like to make an appointment for the second part of our eligibility screening where you will meet with a member of our research team at the Bedford VA, learn more about the study, and possibly enroll. This meeting will take about 45 minutes to an hour. Is there a certain time of day or day of the week that works best for you? [ACTION: Look at calendar for possible information session times. Suggest options that work with their schedule if they are flexible.]

IF TIME SCHEDULED: *Great! We will see you on* [date] *at* [time]. *We will be meeting at the Bedford VA Hospital,* [specific location]. *We will give you a call the day before to confirm that this time still works for you. Do you have any questions at this time?* [Answer questions] *Thank you for your time and have a great day!*

IF UNABLE TO SCHEDULE TIME/DATE: *I'm sorry that we aren't able to find a time that works for you. If these times do not work for you, it may be difficult for you to participate in the study. Would you like to take some time to think about your schedule and call us back later?* [Alternatively, ask them for a time they would like staff to call them back.]

© Boston Medical Center
Veterans Back to Health Trial

TELEPHONE SCREENING PART TWO: AFTER HANGING UP

STEP 5: POST-PHONE CALL PAPERWORK

1. **In StudyTRAX**: Check to make sure all questions were answered and that each section is marked as green.
    - If follow-up needed, contact the appropriate person for more information. Record any issues or comments under Progress Notes after clicking on 'Edit Subject' (record initials and date).
2. **Recruitment spreadsheet**: Record that the individual was screened and scheduled for info session, if applicable.
3. **Calendar**: Make sure the scheduled info session is recorded correctly and completely with the SID.

© Boston Medical Center
Veterans Back to Health Trial

CHECKING VOICEMAIL

- The study line voicemail is checked at the beginning of each day by a research staff member.
- This information is then entered in the "Recruitment Tab" of the recruitment spreadsheet. The staff member records the inquiry date, Veteran's name, telephone number, and any additional notes left on the message. The field is highlighted in WHITE and the Veteran should be contacted as soon as possible.
- Voicemails are deleted after the staff member records the information.

TARGETED MAILINGS

We will use the VA Corporate Data Warehouse (CDW) to identify all Veterans within the Bedford VA Hospital seen in the previous two years that have back pain on their problem list (using ICD-9 codes for low back pain). We will mail letters signed by clinical site champions, a flyer, and a pre-stamped opt-out card to identified Veterans inviting them to join the study.

The postcard allows Veterans to "opt out" of future contact from research staff by sending back the card with the "I do not wish to participate" box checked. About 10-14 days after a letter is sent, research staff will contact Veterans that have not returned an opt-out card and have not called staff by phone to ask if they may discuss the study and its objectives with the Veteran.

Tracking Opt-Out Cards: Opt-out postcards are pre-labeled with a number corresponding to a specific Veteran on the mailing list. Each Veteran receives a unique postcard number so the opt-out cards can be tracked if they are returned. In the recruitment spreadsheet, the "Mailing Tab" contains the Veteran's information and corresponding opt-out card number. If an opt-out card is received by the staff, the staff should enter the date received and a note stating "OPT OUT CARD RECEIVED" in the note section of the spreadsheet. The Veteran's information should then be highlighted in RED to indicate that this Veteran should not be contacted. The opt-out card is then shredded.

Contacting Veterans after Mailing: After 10-14 days, the study staff can begin contacting any Veterans that did not return opt-out cards or already called staff. Staff should go through the list of Veterans who were send the mailing as listed in the "Mailing Tab" of the recruitment spreadsheet. If a Veteran does not answer, note the date of the phone call in the notes section. If the call is successful, follow the script below:

Script: *Hello, is ____________ available?*

> **IF YES**: *Hello ______________. This is* [name] *calling from the Bedford VA Hospital about a letter you may have received in the mail inviting you to join a research study for low back pain.*
>
> > **IF THEY DID NOT RECEIVE LETTER**: *I'm sorry about that. Would you like us to send another one or would you like to hear more about the study now?*
> >
> > > **IF WANTS A LETTER**: *Sure, I will prepare that and send it out as soon as possible. Can you tell me the correct address to send it to?* [ACTION: record address and prepare another letter/flyer/postcard mailing. Make note that

© Boston Medical Center
Veterans Back to Health Trial

another letter was sent with correct address and that the Veteran should be called for follow-up in about a week.]

IF INTERESTED, see Detailed Recruitment Procedures STEP 1 for script.

IF NOT INTERESTED: *Thank you for your time. Can I ask why you are not interested?* [Record reason for their disinterest in joining the study. Highlight their name and information in RED to indicate they should not be contacted in the future]

IF NO: *Do you know when I could call back to get ahold of him/her?*

IF YES: *Great, thank you. I will try back at that time.* [ACTION: Record time to call back. Set an alarm or reminder if necessary.]

IF NO*: Okay, thank you.*

CLINICIAN REFERRALS

Clinicians notify the VA Project Manager when they have a patient that they have discussed the study with and that is interested in being contacted by research staff with more information. Once a referral has been received, the Veteran's name and telephone information are recorded in the "Recruitment Tab." The name of the clinician who referred the Veteran is also recorded under referral method. The Veteran should be contacted within 1-3 days of receiving the referral. When calling Veterans who were referred to by a clinician use the following script:

Script: *Hello, is ____________ available?*

IF YES: *Hello ______________. This is* [name] *calling from the Bedford VA Hospital about a research study for low back pain.* [Clinician's Name] *provided us with your information and may have discussed the study with you at a previous visit. We are contacting you to see if you would be interested in participating.*

IF INTERESTED, see Detailed Recruitment Procedures for script.

IF NOT INTERESTED: *Thank you for your time. Can I ask why you are not interested?* [Record reason for their disinterest in joining the study. Highlight their name and information in RED to indicate they should not be contacted in the future]

IF NO: *Do you know when I could call back to get ahold of him/her?*

IF YES: *Great, thank you. I will try back at that time.* [ACTION: Record time to call back. Set an alarm or reminder if necessary.]

IF NO*: Okay, thank you.*

RECRUITMENT SPREADSHEET TRACKING

Once communication is initiated and maintained with a Veteran, their information should be present in the "Recruitment Tab" of the recruitment spreadsheet. This information includes: inquiry date, phone screen date, info session date, relevant recruitment notes, staff member

© Boston Medical Center
Veterans Back to Health Trial

responsible, referral method, screening ID #, name, and phone number(s). Information not yet available/received should be updated as soon as it becomes available.

In order to track the status of the Veteran, the following color should be used to highlight the Veteran's row of information:

WHITE = Veteran has not been screened, but contact has been initiated

ORANGE = Veteran has been screened and passed screening but has not attended an information session

GREEN = Veteran has attended an information session and enrolled (signed ICF)

BLUE = Veteran has completed baseline survey and been randomized

RED = Veteran has declined, failed screening, or should not be contacted for another reason

© Boston Medical Center
Veterans Back to Health Trial

Challenging Situations Procedure

1. A challenging situation may be one where someone is:
   - Defensive
   - Accusatory
   - Suspicious
   - Rude
   - Condescending
   - Acting peculiar
   - Potentially intoxicated
   - Upset from a mistake/misunderstanding

2. If the individual MAY meet any of these criteria:

SAY: *"It sounds like you have a lot of questions/concerns. I think that my supervisor can better help you. I am transferring you to her now."*

[ACTION: Put them on hold, call coordinator and explain the situation so she can take it from there.]

What Coordinator will do:

- Offer to send more info by mail (to create space and give them time)
- Speak more extensively to assess concerns and eligibility
- Refer participant to Principal Investigator

3. How to record this:
   - **StudyTRAX**: Write "CS" in StudyTRAX under 'Edit Subject' along with a short 2-word explanation (using words listed above). Make sure to explain the situation to anyone who is scheduled to speak or meet with the individual.
   - Recruitment Spreadsheet: Write "CS" in Notes.

4. Report to team at upcoming team meeting so everyone is aware in case the individual calls again.

© Boston Medical Center
Veterans Back to Health Trial

Information Session Procedure

The information session is a chance for the participants to meet with a member of the research team and learn more about the study. At this time, the participant may ask questions and choose whether or not they will enroll in the study.

Reminder Call
Potential participants will be called the day before their scheduled information session. The staff will follow the specified script and leave a message or voicemail if the individual is not available. This will be documented in the recruitment spreadsheet.

Script:

Hello, is _________ available?

IF YES: *Hello ___________. This is* [name] *calling from the Bedford VA Hospital back pain study to remind you of our meeting scheduled tomorrow,* [day of the week] *at* [time]. *We are located in Building 70 and you can knock on the side door when you arrive*. [Give further directions if necessary]. *Is that still a good time for you to meet with us?*

IF NO: *Do you know when I could call back to get ahold of him/her?*

IF YES: *Great, thank you. I will try back at that time.* [ACTION: Record time to call back. Set an alarm or reminder if necessary.]

IF NO: *Okay, thank you.*

Preparation

- Find an empty room with adequate privacy to hold the session.
- Set up and prepare for the meeting – have information packet and gift card ready.
- Check the "Notes" section in the recruitment spreadsheet for any important reminders.

Materials

- Information Sheet/Flyer
- Written Informed Consent Form (ICF)
- HIPAA Authorization
- Study Pen
- Gift Card and Gift Card Signature Log

Introduction
Begin the information session by introducing yourself and the purpose of the meeting. The script below is a suggested introduction to open the session.

Hello. My name is [name]. *Thank you for coming in today. We will start this meeting by reviewing detailed information about the study with a chance for questions, and then we will discuss informed consent.*

Presentation
Before the presentation, say *"If there are any questions during the presentation, please do not hesitate to ask."* Proceed with the presentation slides/overview of the information and elaborate as necessary.

© Boston Medical Center
Veterans Back to Health Trial

Information Session Procedure

Question & Answer Session

After the presentation, ask if there are any questions. Some potential questions are:

✓ What are the chances of being in [GROUP]?

You have a 50/50 chance of being in either the yoga or education group.

✓ Can I choose or specify which treatment group I want to be in?

No one can influence which group a person will be placed into. A computer will randomly assign each participant to a group. Random means "by chance" like flipping a coin.

✓ How difficult are the yoga classes?

Our yoga classes are gentle beginner classes designed especially for Veterans with back pain.

✓ Can I participate in the other treatments outside of the study if I enroll?

When a person voluntarily enrolls in the study, we ask that they stick with their assigned group only and do not start new treatments for back pain outside of the study. Please discuss this with your doctor if necessary.

✓ Can I drop out of the study once I enroll?

You have the right to end your participation at any time. The decision to join the study and continue participation is voluntary and completely up to you.

✓ How does the compensation work?

We will give cash stipends after you complete surveys in-person at the Bedford VA Hospital. We will give $25 after the first survey, the 6-week survey, and the 12-week survey and $50 after the 24-week survey. We will also provide $10 travel compensation for all study visits.

✓ Can I switch out of my assigned group to another one that I prefer?

Once you are assigned to a treatment group, you cannot switch into a different treatment group. You should only enroll in the study if you are willing to participate in either group.

Obtaining Informed Consent

If the individual is no longer interested in participating after detailed information is presented, ask politely why they have changed their mind to make sure there isn't a misunderstanding. After they leave, record the reason they declined participation/consent in the recruitment spreadsheet and StudyTRAX (under In-Person Meeting).

If the individual is still interested, walk them through each section of the ICF, even if it is repetitive. Ask if they have questions often and help clarify any unclear points and/or misunderstandings. If the person confirms that they understand everything that has been discussed and they wish to enroll in the study, there are two forms that must be signed:

1. **Informed Consent Form** (7 pages)
    - Veteran should PRINT their name, SSN, and DATE on top of the last page
    - Veteran should SIGN and DATE on the first signature/date lines of last page

© Boston Medical Center
Veterans Back to Health Trial

  - Staff member obtaining consent must SIGN and DATE the last page after verifying Veteran correctly completed the above
  - Staff member writes Veteran's name (verify correct spelling), date, and the last 4 digits of the SSN on the other pages of the ICF

2. **HIPAA Authorization** (4 pages)
   - Staff writes Veteran's name and last 4 digits of SSN on top of first page
   - Veteran should SIGN and DATE the first signature/date lines of last page

The participant must write the date on their own next to their signature on both forms. Staff should verify it is the correct date (the date includes the month, day, and year) and that the date matches what is written by the staff member.

The participant should be given a copy of the signed ICF and HIPAA Authorization to keep.

Welcome the participant into the study. You may say, "*Congratulations! You are now fully enrolled in the Back to Health study. We will be in touch soon to let you know when we are ready to start the study. It should be about* [time frame estimate]. *Do you have any questions for me?* [Answer questions] *Thanks again and we're excited that you're going to be a part of our study. Have a wonderful day!*"

After the Information Session

1. A copy of the HIPAA Authorization must be sent to VA Medical Records. The VA Project Manager will make a note in the Veteran's electronic medical record that he/she enrolled in the research study.
2. Complete the Informed Consent Progress Note, recording any questions that arose or concerns. File this form in the participant's file.
3. Document the information session, consent, and progress note in StudyTRAX under the In-Person Meeting encounter.

© Boston Medical Center
Veterans Back to Health Trial

Informed Consent Progress Note

Staff Person Obtaining Consent: Day of Informed Consent

Completion Assessment

Participant Signature? ☐ YES ☐ NO

Correct Date? ☐ YES ☐ NO

Notes (*Any areas of major concern?*)

____
____
____
____

Printed First and Last Name	Signature	Date

Review and Verification of Informed Consent

Completion Assessment

Participant Signature? ☐ YES ☐ NO

Correct Date? ☐ YES ☐ NO

Notes

____
____
____
____

Printed First and Last Name	Signature	Date

© Boston Medical Center
Veterans Back to Health Trial

Monitoring of Recruitment Progress

Research staff employ a PDSA (Plan–Do–Study–Act) quality improvement process on a weekly basis to continuously monitor and improve recruitment progress. Statistics for the past week, cohort-to-date, and study-to-date are presented and discussed at the study team weekly meeting using a standardized format. Based on these data, the team discusses progress and can make weekly decisions regarding whether to make any changes in recruitment strategy.

Recruitment data collection begins with the initial inquiry. At this stage, contact information and the method via which the individual learned about the study (e.g., mailing, posted flyer, provider recommendation, etc.) are collected. The full eligibility screening survey is then administered and data are entered directly into StudyTRAX. Numbers of individuals who are found to be eligible, number ineligible, and reasons for ineligibility are recorded. Eligible individuals then are invited to an in-person informational session where they receive detailed information about the study, have opportunities to ask questions, and enroll through signing the written ICF if desired.

Recruitment and eligibility data are compiled and presented weekly. Selected metrics that are highlighted in weekly meeting summaries include:

- Recruitment activities completed (e.g., number of letters mailed, flyers posted, etc.)
- Numbers of inquiries by interested individuals
- Number of Veterans called by staff from mailing(s)
- Frequencies of different ways which individuals learned about the study
- Eligibility pass/fail rates
- Frequencies of specific ineligibility reasons
- Total number of participants enrolled
- Number of in-person informational sessions scheduled
- Follow-ups needed for participants with uncertain eligibility
- Follow-ups needed for individuals who have inquired about the study but have not yet completed eligibility screening

© Boston Medical Center
Veterans Back to Health Trial

Eligibility Verification Checklist

SID: ______________________

A form with this information is filled out on StudyTRAX

The following questions must be asked to participants prior to scheduling the baseline survey if 45 days or more will pass between completion of screening and the baseline survey.

If the participant is unable to give an answer to any of the below questions, they will be considered ineligible to participate in the study.

The participant must answer **YES** to the following question in order to be eligible:

	Yes	No
Have you had low-back pain at least half the days in the past 6 months?	☐	☐

The participant must answer **NO** to the following questions in order to be eligible:

	Yes	No
In the next 6 months, are you planning to move out of the area or have a significant change in your schedule that would make it impossible to participate in the study?	☐	☐
Have you practiced yoga multiple times per month in the past 6 months?	☐	☐
Have you read *The Back Pain Helpbook* in the past 6 months?	☐	☐
**WOMEN UNDER 55 ONLY**: Are you pregnant or do you suspect you're pregnant or are you planning to try to get pregnant in the next 6 months?	☐	☐

The participant must answer **between 4 and 10** on the following question to be eligible:

Please rate your low back pain on average in the past 7 days on a scale of 0 to 10, where 0 is no pain and 10 is the worst imaginable pain? ☐

Did the participant meet all of the above criteria for participation in the study?

☐ **PASS**
☐ **FAIL**

STAFF MEMBER VERIFYING ELIGIBILITY:

Name **Signature** **Date**

© Boston Medical Center
Veterans Back to Health Trial

Yoga Intervention Procedures

3.0

© Boston Medical Center
Veterans Back to Health Trial

Yoga Overview

Veterans in the yoga intervention will be asked to attend one class per week for 12 weeks. Classes will be 75 minutes long and will include yoga breathing exercises, a reading of yoga philosophy with a poem, yoga postures with an emphasis on yoga breathing, and a relaxation exercise. Yoga teachers will follow the yoga protocol as outlined in the Yoga Teacher Manual. Attendance will be taken by research staff. Mats and props will be available for participants to use during classes. The postures gradually increase in difficulty throughout the 12 weeks.

Yoga participants will be encouraged to practice at home for 30 minutes on days when they do not attend class. They will be provided at baseline or their first class with a Yoga Participant Handbook, mat, block, and strap to aid home practice. Veterans will be given a link to view yoga home practice videos online. If the Veteran does not have access to internet at home, a DVD will be given with the videos. DVDs will also be given upon request.

Yoga classes will take place in group rooms located at the Bedford VAMC where we will assure that there is more than adequate room for classes of 8-10 Veterans and two yoga teachers. Two yoga instructors teach each class to assure a low Veteran-to-instructor ratio. This ensures adequate individual attention from the teacher, maximizes safety and effectiveness, and allows flexibility in yoga teacher scheduling based on the study's needs.

© Boston Medical Center
Veterans Back to Health Trial

Veterans Back to Health

Yoga Flow Sheet

Events	Relevant Documents and Forms
**Randomization**	• Yoga Randomization Script<br>• Yoga Welcome Packet
**Week 1**	• Yoga Reminder Call Script<br>• Yoga Take-Home Supplies
**Weeks 2-12**	• Yoga Reminder Call Script<br>• Yoga Attendance Tracking
**12-week Survey**	• Follow-Up Phase Home Practice Logs
**24-week Survey***	• Corresponding Survey and Forms<br>• Survey Administration Procedures<br>• Honoraria Signature Form

END OF STUDY

* This event is discuss in detail in another section of this manual.

© Boston Medical Center
Veterans Back to Health Trial

Yoga Randomization Script

Overview

Once participants have completed their baseline survey, they will receive their treatment assignment by an unblinded staff member.

Suggested Script

You have been randomly selected to be in the yoga group. We ask that you attend yoga class each week. Which class time do you think best fits with your schedule? [Record Class Time] *We would like you to commit to one class time but if you miss your scheduled class you will be able to attend any of the other yoga classes that week to make-up the missed class. We just ask that you please call us in advance if you will miss class and need to reschedule. We will give you a reminder call the day before your class each week.*

Your yoga class will take place on [day(s) of the week] *starting with* [start date]. *Your yoga class will be held at* [location]. *We will be sending you a letter in the mail with this same information.*

[Reference Yoga Information Sheet] *Please come to class wearing loose fitting clothing so that you can easily move about. We will be asking you to take your shoes off and also your socks if you are comfortable doing so. If you'd like, you are welcome to bring a towel with you in case you perspire, although keep in mind the class will not involve very strenuous exercise.*

[Reference Yoga Home Practice Log] *We recommend you practice for 30 minutes each day that you do not have class and then record it on this sheet. The Home Practice Log is for you to fill out each time you practice at home. We ask that you bring it with you to turn it at yoga class each week.*

© Boston Medical Center
Veterans Back to Health Trial

Detailed Yoga Procedures

OVERVIEW OF ROLES & RESPONSIBILITIES

The primary responsibilities in coordinating the yoga arm is to keep in contact with yoga participants and teachers to:

- Schedule yoga classes for each cohort that align with yoga teacher availability, Veteran preference, and room availability
- Make sure all forms and supplies are fully stocked for classes each week
- Conduct reminder phone calls and check-in phone calls
- Collect attendance and distribute gift cards for travel at classes each week
- Collect home practice logs, de-identify, and complete data entry as needed

BEFORE BASELINE SURVEY WEEK

Prepare materials for about half of the expected number of participants for the cohort. After randomization, the participants receive:

1. Yoga Welcome Packet, which includes Welcome Packet Introduction, Yoga Information Sheet, Study Contact Information, Home Practice Logs, Yoga Calendar, and Class Listing
2. Yoga block, strap, participant guide, mat, and mat bag
3. Study water bottle
4. Blue study tote bag

Home practice supplies (#2-4 above) may be given to participants when they attend their first class.

DURING BASELINE SURVEY WEEK

After completion of the baseline survey, the participant will be randomized. Participants should be introduced to the yoga group assignment using the Yoga Randomization Script. When going through the Yoga Welcome Packet, make sure to go through each element, particularly the class times. Have the participant choose a class time that works best for them. If a participant says they do not need/want a class reminder call, say, "*We will call everyone before the first class to make sure they know when and where the class is, but if you do not want weekly calls after that, just let us know after the first class.*"

Record each participant's choice on a *Yoga Class Sign-Up* paper sheet. At the end of the day, record this information on the *Yoga Class Divisions* spreadsheet to keep track of how many people are in each class. Fill out the reminder card for the participant with their chosen class time (inside Welcome Packet). If the participant is NOT able to schedule a specific class time during the baseline visit, give them the class options and tell them staff will call within 3 days to pick a class time that works best for them.

ENGAGEMENT AND RETENTION

Participants should receive reminder phone calls the day before each class (See Yoga Class Reminder Script). If a participant misses a class, they should be called the following day to schedule a make-up class time and/or troubleshoot attendance issues. Any notable content of

© Boston Medical Center
Veterans Back to Health Trial

these phone calls should be recorded in *Yoga Attendance Tracking* worksheet for future reference as necessary.

TRACKING ATTENDANCE

Attendance is recorded during each yoga class when participants sign-in before class. A research staff member is present to make sure attendance is completed accurately and to distribute gift cards for travel to attendees.

A Yoga Attendance Tracking spreadsheet should be maintained to document reminder calls, follow-up calls, reasons for missing classes, and making up missed classes. For the entire 12-week treatment period, compliance means the participant has attended ≥75% (≥9) classes.

Week #				
**SID**	**Scheduled Class Time**	**Classes Attended**	**Attendance To-Date**	**Notes**
901	Tues.	1	100%	Left VM reminder 7/8/13 -[INITIALS]
902	Sat.	0	29%	Left VM reminder 7/12/13 & follow-up VM on 7/15/13 after missed class. -[INITIALS]
903	Sat.	1	57%	Scheduled make-up on Tue after missing last week but couldn't go—try again next week. -[INITIALS] 7/7/13
904	Tues.	0	43%	Reports hurt hand & didn't go to class d/t pain—AE recorded -[INITIALS] 7/12/13
905	Tues.	1	86%	Verbal confirmation reminder 7/8/13 -[INITIALS]
906	Sat.	2	100%	Attending class on Tues b/c out of town this weekend -[INITIALS] 7/9/13
907	Sat.	2	86%	Dropped-in to Tues class to make up last week -[INITIALS] 7/8/13
908	Tues.	1	71%	Got postcard in mail & wants to improve attendance -[INITIALS] 7/8/13

A summary Yoga Attendance Tracking spreadsheet should also be maintained with all yoga participants' attendance data. The spreadsheet allows the study team to track week-to-week attendance, total number of classes attended, and individual attendance rates (classes attended/total classes offered). Weekly review of this tracking sheet at team meetings allows targeted efforts to help with engagement and retention.

**Participant**	**Week #**												**Total Classes Attended**	**Attendance Rate**
	**1**	**2**	**3**	**4**	**5**	**6**	**7**	**8**	**9**	**10**	**11**	**12**		
**501**	1	1	1	1	1	1	1	1	2	1	1	0	12	**100%**
**502**	1	1	1	0	1	1	1	1	1	1	1	0	10	**83%**
**503**	1	1	1	0	1	1	0	0	0	1	1	0	7	**58%**
**504**	0	0	1	1	2	0	0	0	0	0	0	1	5	**42%**
**505**	1	1	1	1	1	1	0	1	1	1	0	0	9	**75%**

© Boston Medical Center
Veterans Back to Health Trial

Week by Week Yoga Tasks

Week 1	Before first classes, double check that all supplies and blank forms are in the appropriate locations and confirm final teacher schedules.<br>Reminder calls for all participants the day before first class (and possibly the day of, if needed). Follow-up calls for those that did not attend scheduled class. Keep track of the participants that have opted-out of weekly reminder calls in the Yoga Attendance Tracking spreadsheet.<br>At the first yoga class, distribute home practice supplies and make sure sufficient explanation is given about each item in the bags. A yoga teacher or a research staff member can give this information as appropriate.
Weeks 2-5	Reminder and follow-up calls for class attendance. Update the Yoga Attendance Tracking spreadsheet weekly. During Weeks 4-5, remind participants that the 6-week survey is coming up and schedule a survey time if possible. Update the Yoga Attendance Tracking spreadsheet weekly.
Week 6 **(SURVEY)**	Reminder calls and follow-up calls for class attendance. These calls can also be the time to remind participants about their 6-week survey appointment and that they should bring completed home practice logs to their survey appointment if they haven't turned them in during class. Ensure their survey appointment is before or more than 24 hours after their yoga class.
Weeks 7-11	Reminder and follow-up calls for class attendance. During Weeks 10-11, remind participants that the 12-week survey is coming up and schedule a survey time if possible. Update the Yoga Attendance Tracking spreadsheet weekly.
Week 12 **(SURVEY)**	Reminder calls and follow-up calls for class attendance. These calls can also be the time to remind participants about their 12-week survey appointment and that they should bring any completed home practice logs that they have not already turned in to their survey appointment. Ensure their survey appointment is not scheduled in the 24 hours following their yoga class. Update the Yoga Attendance Tracking spreadsheet weekly.
Week 13	Ensure all yoga participants have been notified classes have ended and they should continue with home practice. Make it clear that the study is *not* over.
Week 24 **(SURVEY)**	Survey reminder calls and an informal check-in.

© Boston Medical Center
Veterans Back to Health Trial

Yoga Class Reminder Script

Overview

Participants will receive a reminder call for yoga classes every week unless they choose to opt out. All participants will receive a reminder call during week 1. They will receive a phone call the day before their scheduled class each week, unless they request otherwise.

Suggested Script

Hello, is _______________ available?

> **IF YES:** *Hello __________. This is* [name] *from the Bedford VA yoga study calling to remind you about your yoga class tomorrow at* [time] *in* [location]. *Will you be able to come?*
>
> *Great! We will see you then.*
>
> **IF NO:** *When is the best time for me to call back to reach _________? Thank you.*

© Boston Medical Center
Veterans Back to Health Trial

Yoga Home Practice

Participants will be encouraged to practice for 30 minutes each day on days when they do not attend class. They will be provided with a participant handbook, yoga mat, block, and strap to aid home practice. Yoga participants will be given a specific link to where home practice videos will be placed online. If Veterans report that they do not have consistent internet access at home, DVDs will be provided with the videos.

Yoga participants are asked to record their home practice on a Home Practice Log and turn in their completed logs each week when they attend yoga class. After class, yoga teachers will remind participants to turn in their completed Home Practice Logs and practice at home each day when possible. Yoga teachers will also encourage participants that did not already turn in a completed log to fill one out before leaving. All collected logs will have the participant's name written at the top. These logs will be completely de-identified before data entry.

Here is an example of what a completed Home Practice Log might look like:

John Doe

Weekly Home Practice Log

We suggest that you practice yoga for 30 minutes on days you do not have class. It is okay if you are unable to practice every day. We just ask that you are honest when filling out this log.

Please record when and for how long you did yoga at home, starting from your last class.
Please do not include yoga practice done during class on this log.

EXAMPLE

	Date: 1/1/15	Date: 1/2/15	Date: 1/3/15	Date: 1/4/15	Date: 1/5/15	Date: 1/6/15	Date: 1/7/15
Have you practiced yoga at home today?	☒ Yes ☐ No	☐ Yes ☒ No	☒ Yes ☐ No	☒ Yes ☐ No	☒ Yes ☐ No	☐ Yes ☒ No	☐ Yes ☒ No
Minutes:	25 min.	____ min.	15 min.	30 min.	25 min.	____ min.	____ min.
Comments:							class

Please tell a yoga teacher or a research staff member if you experience any injury during the course of the study. You can call research staff at **781-687-2000 ext. 5520**.

Version 1.0

STAFF USE ONLY

COLLECTION Initials: ________ Date: ________

DATA ENTRY Initials: ________ Date: ________

© Boston Medical Center
Veterans Back to Health Trial

Education Intervention Procedures

© Boston Medical Center
Veterans Back to Health Trial

Education Overview

Individuals randomized to the education group will receive *The Back Pain Helpbook*, an educational book with strategies for self-care including an exercise program, lifestyle modification, and tips for managing flare-ups. In addition, they will receive a checklist outlining specific chapters to read over the course of the 12 weeks. This checklist sheet divides the book into four sections of about 35–60 pages each to help participants split the readings over the study period.

Before weeks 3, 6, 9, and 12, participants will receive 1-2 page newsletters written at a 6th grade reading level that highlight main points from the assigned chapters. Unblinded staff will conduct check-in calls about a week after newsletters are mailed to assess progress with the readings.

© Boston Medical Center
Veterans Back to Health Trial

Education Flow Sheet

Events	Relevant Documents and Forms
**Randomization**	• Education Randomization Script<br>• Education Welcome Packet<br>• Take-Home Supplies
**3-week Check-In**	• Newsletter #1<br>• Education Check-In Questions
**6-week Survey & Check-In**	• Corresponding Survey and Forms<br>• Newsletter #2<br>• Education Check-In Questions
**9-week Check-In**	• Newsletter #3<br>• Education Check-In Questions
**12-week Survey & Check-In**	• Corresponding Survey and Forms<br>• Newsletter #4<br>• Education Check-In Questions
**24-week Survey & Check-In**	• Corresponding Survey and Forms<br>• Education Check-In Script

END OF STUDY

© Boston Medical Center
Veterans Back to Health Trial

Education Randomization Script

Overview

Once participants have completed their baseline survey, they will receive their treatment assignment by an unblinded staff member.

Suggested Script

You have been randomly selected to be in the education group. You will receive The Back Pain Helpbook, which will give you strategies to help you manage your own back pain. During the study, we ask that you read the book, practice the suggested exercises, and try the recommendations in each section. We have created a Curriculum and Checklist to help you finish reading the book over the next 12 weeks of the study. We have made these suggestions to divide the reading about evenly so that you will read about a quarter of the book every three weeks. The checklist is only meant to be a guide so please read the book at your own pace as you feel comfortable.

© Boston Medical Center
Veterans Back to Health Trial

Detailed Education Procedures

OVERVIEW OF ROLES & RESPONSIBILITIES

The primary responsibilities in coordinating the education arm is to keep in contact with education participants to:

- Send newsletters
- Conduct check-in phone calls (including adverse event inquiries and compliance data)

BEFORE BASELINE SURVEY WEEK

Prepare materials for about half of the expected number of participants for the cohort. After randomization, the participants receive:

1. Education Welcome Packet, which includes Welcome Packet Introduction, Education Information Sheet, Study Contact Information, *The Back Pain Helpbook* Curriculum and Checklist, and Education Calendar
2. *The Back Pain Helpbook* with missing pages inserted and pencil pouch containing one highlighter, one study pen, one study bookmark, and one stack of study post-its
3. Study water bottle
4. Blue study tote bag

DURING BASELINE SURVEY WEEK

After completion of the baseline survey, the participant will be randomized. Participants should be introduced to the education group assignment using the Education Randomization Script. When going through the Education Welcome Packet, make sure to go through each element, particularly the Curriculum and Checklist and the Education Calendar. The Curriculum and Checklist is a rough guideline for Education participants to follow—*please encourage participants to read at their own pace as they are comfortable*.

DEFINITION OF COMPLIANCE

At *individual check-ins,* compliance means the participant has read ≥75% (¾) of the recommended chapters from the curriculum and checklist.

Over the entire *12-week Treatment Period,* compliance means the participant has read ≥75% (¾) of the entire book. This is assessed by a final question at 12 weeks. If the participant is not available for the 12-week check-in, compliance can be calculated by adding together the amounts read at individual check-ins. Adding together the amounts reported at each check-in may not take into consideration readings that do not line up with the recommended curriculum (e.g., catching up on missed chapters after a check-in call). Thus, the staff member conducting check-in calls should ask if additional reading was completed outside the suggested chapters, whether previous or later chapters.

ORGANIZATION AND COLLECTION OF COMPLIANCE DATA

Check-in calls will assess how the participant is doing with the book, gently encourage them to continue the reading, and see if they are following the suggested pace set by the curriculum.

© Boston Medical Center
Veterans Back to Health Trial

Detailed Education Procedures

After weeks 3, 6, 9, and 12, education participants will be asked about their progress. Refer to the Education Check-in Script for more detail. ***NOTE**: It may be useful to ask during check-in calls 2-4 if the participant went back to read previously recommended chapters since the last check-in call. If they did, it should be recorded as part of the check-in call because these calls will determine compliance.*

Compliance data should be recorded during each check-in call. This information will be entered into StudyTRAX by the person that collects it. A spreadsheet that tracks check-in call data is useful for presenting updates at team meetings.

SID	Date	How much did you read?					Notes	AE?
		None	¼	½	¾	All		
601	7/12/12			✓			Did not receive newsletter—KDS updated contact info	N
602	7/13/12				✓			N
603	7/13/12					✓	Hurt his back shoveling around 7/10. AE reported on 7/13—KDS entered into StudyTRAX	Y
604	7/11/12					✓	Read ahead to Ch. 15	N
605	7/12/12			✓				N
606	7/12/12				✓		Said newsletter is very helpful!	N
607	7/10/12		✓				Has been busy with new job.	N
608	7/12/12			✓				N
609	7/10/12	✓					Says she hasn't been able to find time to read—requested additional check-in/reminder in 2 weeks.	N
610	7/12/12			✓			Didn't complete all chapters but read last assignment since last call. Has read ~¾ of curriculum so far	N

HOW TO ENTER CHECK-IN DATA INTO STUDYTRAX

1. From the enrolled participant's Encounters Update page, add the appropriate check-in encounter.
2. The screen will have the appropriate check-in question(s) to fill out. Click Save and Return after entering the data.

EDUCATION NEWSLETTERS

Newsletters are sent to education participants before weeks 3, 6, 9, and 12. Alternatively, education participants may receive their respective newsletters during the 6-week and 12-week surveys. The newsletters supplement the readings from *The Back Pain Helpbook.* Much like the pace of the Curriculum and Checklist, the four newsletter volumes each correspond to about ¼ of the book.

© Boston Medical Center
Veterans Back to Health Trial

Week by Week Education Tasks

Week 2	Prepare and mail Newsletter #1.
Week 3	Check-In #1. This is a good time to remind participants that the 6-week survey is coming up. Ask if they would like to schedule a day and time to come in at that moment or if they would prefer to be called the following week.
Week 5	Prepare and mail Newsletter #2.
Week 6 **(SURVEY)**	Check-In #2. This can be conducted while a participant is visiting in-person for survey administration. Make sure participants received the newsletter(s) in the mail and be prepared to hand out newsletters to participants that say they haven't gotten one. Update contact/mailing information as appropriate.
Week 8	Prepare and mail Newsletter #3.
Week 10	Check-In #3. This is a good time to remind participants that the 12-week survey is coming up. Ask if they would like to schedule a day and time to come in at that moment or if they would prefer to be called the following week.
Week 11	Prepare and mail Newsletter #4.
Week 12 **(SURVEY)**	Check-In #4. This can be conducted while a participant is visiting in-person for survey administration. Make sure participants received the newsletter(s) in the mail and be prepared to hand out newsletters to participants that say they haven't gotten one. Update contact/mailing information as appropriate.<br>Remind participants that the study isn't over and they will hear from staff in about 6 weeks for a check-in call.
Week 24 **(SURVEY)**	An informal check-in either during survey scheduling or in-person. Record who is called, when, and any relevant notes.

© Boston Medical Center
Veterans Back to Health Trial

Education Check-In Script

Overview

A Bedford VA research staff member will call education participants to check-in during weeks 3, 6, 9, and 12. Check-ins during weeks 6 and 12 can be done during in-person survey administration.

Unblinded study staff will also check-in with education participants around week 18 of the study. Staff should encourage participants to continue following information from the book. This phone call should not be rushed so that staff can help participants feel engaged in the study.

Suggested Script

Hello, is _______________ available?

> **IF YES:** *Hello ________. This is* [name] *from the Back to Health study at the Bedford VA. How are you? I'm calling to make sure you've received your newsletter and see how you're doing with The Back Pain Helpbook.*
>
> [See Education Check-In Questions on following pages]
>
> *Do you have any other questions for me today? If you need anything, don't hesitate to call us. Have a nice day!*
>
> **IF NO:** *Do you know the best time for me to call back to reach him/her? Thank you!*

© Boston Medical Center
Veterans Back to Health Trial

Week 3 Check-In

We would like to get an idea about your progress with The Back Pain Helpbook. For weeks 1-3, we recommended that you complete chapters 1-3. It is okay if you haven't been able to finish these chapters. We would like you to answer as honestly as possible.

For weeks 1-3, how much of chapters 1-3 have you been able to complete?

☐ None ☐ Half ☐ All

☐ Less than half ☐ More than half

Have you had any changes in your health status during the study?

☐ Yes ☐ No

IF YES, please complete Adverse Event Form.

Name/Signature of Staff: ____ **Date:** _____________

Week 6 Check-In

We would like to get an idea about your progress with The Back Pain Helpbook. For weeks 4-6, we recommended that you complete chapters 4-11. It is okay if you haven't been able to finish these chapters. We would like you to answer as honestly as possible.

For weeks 4-6, how much of chapters 4-11 have you been able to complete?

☐ None ☐ Half ☐ All

☐ Less than half ☐ More than half

Have you had any changes in your health status during the study?

☐ Yes ☐ No

IF YES, please complete Adverse Event Form.

Name/Signature of Staff: ____ **Date:** _____________

© Boston Medical Center
Veterans Back to Health Trial

Week 9 Check-In

We would like to get an idea about your progress with The Back Pain Helpbook. For weeks 7-9, we recommended that you complete chapters 12-17. It is okay if you haven't been able to finish these chapters. We would like you to answer as honestly as possible.

For weeks 7-9, how much of chapters 12-17 have you been able to complete?

- ☐ None ☐ Half ☐ All
- ☐ Less than half ☐ More than half

Have you had any changes in your health status during the study?

- ☐ Yes ☐ No

IF YES, please complete Adverse Event Form.

Name/Signature of Staff: ______________________ **Date:** __________

Week 12 Check-In

We would like to get an idea about your progress with The Back Pain Helpbook. For weeks 10-12, we recommended that you complete chapters 18-22. It is okay if you haven't been able to finish these chapters. We would like you to answer as honestly as possible.

For weeks 10-12, how much of chapters 18-22 have you been able to complete?

- ☐ None ☐ Half ☐ All
- ☐ Less than half ☐ More than half

Throughout the entire 12 weeks of the study, how much of the book have you completed overall?

- ☐ None ☐ Half ☐ All
- ☐ Less than half ☐ More than half

Have you had any changes in your health status during the study?

- ☐ Yes ☐ No

IF YES, please complete Adverse Event Form.

Name/Signature of Staff: ______________________ **Date:** __________

© Boston Medical Center
Veterans Back to Health Trial

The Back Pain Helpbook Curriculum

We ask that you read *The Back Pain Helpbook* over the next 12 weeks. Every 3 weeks, a member of our study staff will call you to check-in and see how you are doing with the book. To help you, we've divided the book into four sections and we suggest you read the following chapters within the times mentioned below. Please feel free to read at your own pace. This sheet is meant to serve only as a guide in completing the assignment in 12 weeks.

Curriculum and Checklist: Weeks 1-3

Chapters 1-3 (Pages 3-43)

I. Before You Start

Chapter 1: A Self-Assessment (p. 3 – 12)

- ☐ A. Introduction to the book – p. 3.
- ☐ B. Questions to help you understand how back pain affects your life – p. 4 – 12.

Date Chapter Completed: _____ /_____ /_______

II. Understanding Back Pain for Effective Self-Care

Chapter 2: Back Pain and You (p. 15 – 38)

- ☐ A. The biology of back pain, including diagrams of bones, muscles, and joints involved – p. 16 – 25.
- ☐ B. How your brain and nerves affects your back pain – p. 24 – 25.
- ☐ C. Common sources of back pain – p. 26 – 31.
- ☐ D. Activity: List all the things that reduces and increases your back pain – p. 38.

Date Chapter Completed: _____ /_____ /_______

Chapter 3: Reversing the Downward Spiral of Back Pain (p. 39 – 43)

- ☐ A. Why "Taking It Easy" for too long is the wrong approach – p. 39.
- ☐ B. Why trying to avoid your pain adds strain to your back – p. 39 – 40.
- ☐ C. Ways you can safely stay active – p. 41 – 42.
- ☐ D. Action Summary: Tips on how to safely perform your normal activities – p. 42 – 43.

Date Chapter Completed: _____ /_____ /_______

Week 3 – Expect a phone call from study staff this week!

© Boston Medical Center
Veterans Back to Health Trial

The Back Pain Helpbook Curriculum

Curriculum and Checklist: Weeks 4-6

Chapters 4-11 (Pages 47-110)

III. Effective Self-Care

Chapter 4: Effectively Managing Your Back Pain (p. 47 – 52)

☐ A. Steps to managing your back pain – p. 48 – 52.
☐ B. Activity: Write down your long-term goals in the first box. In the second box, write down some things that will help you reach your goal – p. 49.
For example:
- *Goal #1: This year, I'm going to complete a 5K run/walk in Boston.*
- *How to achieve my goal: I'll start off by walking once around the block. Every week I'll increase my distance little by little.*

☐ C. Four-step process on how to make your plan of action – p. 50.
☐ D. Tips on how to boost your confidence – p. 51.
☐ E. Action Summary: Five steps to planning for better living – p. 52.

Date Chapter Completed: _____ /_____ /_______

Chapter 5: Managing Flare-Ups and Emergencies (p. 53 – 56)

☐ A. Three important tips for managing flare-ups, or severe back pain – p. 53.
☐ B. What to do when you have a flare-up and how to control the pain – p. 53 – 56.
☐ C. Three bullet points about how long pain from flare-ups typically last – p. 56.

Date Chapter Completed: _____ /_____ /_______

IV. Treatments for Back Pain: No Magic Bullets

Chapter 6: Working with Doctors and Other Health Professionals (p. 59 – 64)

☐ A. Getting the most out of an office visit – p. 59 – 61.
☐ B. Alternatives and options – p. 61 – 63.
☐ C. Action Summary: How to talk to your doctor about your back pain. The next time you visit your physician, try out the steps listed – p. 63 – 64.

Date Chapter Completed: _____ /_____ /_______

Chapter 7: Medicines for Controlling Back Pain (p. 65 – 74)

☐ A. Tips for taking over-the-counter medicine – p. 66.
☐ B. Different types of medications for back pain and their side effects – p. 66 – 74.
☐ C. Information you need from your doctor – p. 73.
☐ D. What to tell your doctor when getting a new prescription – p. 74.

Date Chapter Completed: _____ /_____ /_______

© Boston Medical Center
Veterans Back to Health Trial

Chapter 8: Physical Methods of Pain Control (p. 75 – 81)

- ☐ A. The many benefits of exercise – p. 75.
- ☐ B. Tips for applying cold and heat – p. 76.
- ☐ C. Different physical methods for back pain such as, massage, spinal manipulation and mobilization, surgery, acupuncture, etc. – p. 77 – 81.

Date Chapter Completed: _____ /_____ /_______

Chapter 9: Pain Control through Mind-Body Techniques (p. 83 – 95)

- ☐ A. Learning to distract yourself from the pain – p. 84.
- ☐ B. Activity: Identifying opportunities for distraction – p. 84.
- ☐ C. Reinterpreting the pain – p. 85.
- ☐ D. Breathe deeply to relieve stress – p. 85.
- ☐ E. Using deep breathing or music to help you relax – p. 87.
- ☐ F. Other mind-body techniques, such as meditation and prayer, guided imagery, etc. – p. 87 – 94.
- ☐ G. Action Summary: Three tips on how to use mind-body techniques – p. 94.
- ☐ H. Planning For Better Living: Four questions to answer on using mind-body techniques to manage your pain – p. 94 – 95.

Date Chapter Completed: _____ /_____ /_______

Chapter 10: Handling the Effects of Pain on Thoughts and Emotions (p. 97 – 105)

- ☐ A. How avoiding frustration, managing angry feelings, and confronting fears can help your back pain – p. 97 – 100.
- ☐ B. How negative thoughts lead to negative emotions – p. 100 – 102. On p. 102, there's a box that gives examples of common negative thoughts among people with back pain and how to replace those thoughts with balanced ones.
- ☐ C. How you can put an end to your negative thoughts – p. 102 – 103.
- ☐ D. Action Summary: Tips for decreasing negative feelings – p. 104.
- ☐ E. Planning For Better Living: Three questions to answer that will help you change your negative thoughts about back pain – p. 104 – 105.

Date Chapter Completed: _____ /_____ /_______

Chapter 11: Recognizing Depressive Conditions When You Have Back Pain (p. 107 – 110)

- ☐ A. Common signs of depression – p. 108.
- ☐ B. Treatment options and thoughts of suicide – p. 108 – 109.
- ☐ C. Action Summary: Tips on coping with depression – p. 110.

Date Chapter Completed: _____ /_____ /_______

Week 6 – Expect a call from study staff this week!

© Boston Medical Center
Veterans Back to Health Trial

The Back Pain Helpbook Curriculum

Curriculum and Checklist: Weeks 7-9

Chapters 12-17 (Pages 113-174)

V. Physical Activity and Exercise

Chapter 12: A Balanced Approach to Physical Activity (p. 113 – 117)

- ☐ A. A balanced approach to exercise and activity – p. 113 – 114.
- ☐ B. How to pace your activities and decide the type of exercise that is right for you – p. 115 – 116.
- ☐ C. Four steps to make lifestyle changes – p. 117.

Date Chapter Completed: _____ /_____ /_______

Chapter 13: The Comfort Zone: Key to Good Posture & Body Mechanics (p. 119 – 139)

- ☐ A. How to find your neutral position in three different ways: 1) standing, 2) sitting, and 3) reclining – p. 121 – 123.
- ☐ B. How to keep good posture – 123 – 125.
- ☐ C. Strategies for performing everyday activities safely, including getting out of bed, bathing, putting on shoes, driving your car, lifting, etc. – p. 125 – 138.
- ☐ D. Planning For Better Living: Four questions to answer that will teach you how to use your neutral position during activities – p. 138 – 139.

Date Chapter Completed: _____ /_____ /_______

Chapter 14: Stretch to Prevent Pain and Stiffness (p. 141 – 152)

- ☐ A. Why flexibility and stretching is good for your back – p. 141 – 143.
- ☐ B. Pictures and descriptions of stretches for back flexibility – p. 143 – 146.
- ☐ C. Pictures and descriptions of stretches for hip and leg flexibility – p. 147 – 150.
- ☐ D. An all-over stretch to do before getting out of bed in the morning – p. 151.
- ☐ E. Action Summary: Read these tips for performing flexibility exercises – p. 151.
- ☐ F. Planning For Better Living: Four questions to answer that will help you improve your flexibility – p. 152.

Date Chapter Completed: _____ /_____ /_______

Chapter 15: Exercises for Building Strength and Endurance (p. 153 – 162)

- ☐ A. How strength and endurance will improve your back pain – p. 153 – 154.
- ☐ B. Strengthening exercises for back support muscles – p. 154 – 159.
- ☐ C. Guidelines for using gym equipment – p. 159.
- ☐ D. Action Summary: Tips for increasing strength and endurance – p. 160.
- ☐ E. Planning For Better Living: Four questions to answer that will help you build strength and endurance – p. 161.

Date Chapter Completed: _____ /_____ /_______

© Boston Medical Center
Veterans Back to Health Trial

Chapter 16: Feeling Better through Aerobic Activities

- ☐ A. Why extra weight is bad for your back – p. 164.
- ☐ B. How to choose aerobic exercises and calculate your heart rate – p. 154 – 165.
- ☐ C. Back care tips for specific aerobic exercises, such as dance, basketball, etc. – p. 165 – 166.
- ☐ D. Planning For Better Living: Four questions to answer that will help you start your exercise routine – p. 168.

Date Chapter Completed: _____ /_____ /_______

Chapter 17: Staying Active in an Inactive World

- ☐ A. Finding time for exercise when you have no free time – p. 170 – 171.
- ☐ B. Action Summary: Six steps for finding time to exercise – p. 171.
- ☐ C. How to make exercise a habit and begin your exercise program – p. 171 – 172.
- ☐ D. Planning For Better Living: Four questions to answer that will help you make your exercise goals – p. 173 – 174.

Date Chapter Completed: _____ /_____ /_______

Week 9 – Expect a phone call from study staff this week!

© Boston Medical Center
Veterans Back to Health Trial

The Back Pain Helpbook Curriculum

Curriculum and Checklist: Weeks 10-12

Chapters 18-22 (Pages 177-213)

VI. Everyday Insights for Better Living

Chapter 18: Solutions for Sleep Problems (p. 177 – 183)

- ☐ A. Good beds for bad backs – p. 177 – 178.
- ☐ B. Comfortable sleeping positions for your back – p. 178 – 179.
- ☐ C. How to develop a healthy sleep pattern – p. 179 – 180.
- ☐ D. Avoiding future sleep problems – p. 180 – 182.
- ☐ E. Planning For Better Living: Four questions to answer that will help you improve the quality of your sleep – p. 182-183.

Date Chapter Completed: _____ /_____ /_______

Chapter 19: Strengthening Your Relationships (p. 185 – 191)

- ☐ A. How to deal with people who are overly protective of you – p. 185 – 186.
- ☐ B. How to maintain open communication with others – p. 186 – 1874.
- ☐ C. Tips for expressing your feelings – p. 187 – 188.
- ☐ D. Ways to ask for help when you need it, and decline it when you do not – p. 189.
- ☐ E. Action Summary: How to avoid problems in your relationships – p. 189 – 190.
- ☐ F. Planning For Better Living: Four questions to answer that will help you improve your relationships – p. 190 – 191.

Date Chapter Completed: _____ /_____ /_______

Chapter 20: Intimacy and Sex (p. 193 – 198)

- ☐ A. Broadening your sexual pleasure and talking to your partner about sex – p. 193 – 194.
- ☐ B. How to control back pain during sex and overcome fears of pain and injury – p. 194 – 196.
- ☐ C. Bringing intimacy back to your daily life – p. 196 – 197.
- ☐ D. Planning For Better Living: Five questions to answer that will help you enhance intimacy, sensuality, and sexuality – p. 197 – 198.

Date Chapter Completed: _____ /_____ /_______

© Boston Medical Center
Veterans Back to Health Trial

Chapter 21: Back Pain and Your Job (p. 199 – 205)

- ☐ A. Tips on how to prevent back problems while you are at work – p. 199 – 201.
- ☐ B. What to do when back pain keeps you from going to work – p. 201 – 203.
- ☐ C. What to do if you suffered an on-the-job back injury and what to say when applying for a new job – p. 203 – 205.
- ☐ D. Action Summary: Tips for workers with back pain – p. 205.

Date Chapter Completed: _____ /_____ /_______

VII. Living Your Plan

Chapter 22: Final Thoughts on Feeling and Doing Better (p. 209 – 213)

- ☐ A. Tips on remaining in control of your life despite back pain. Each tip notes the chapter it was discussed in if you want to go back and re-read it more in detail – p. 210.
- ☐ B. Reminders for preventing relapses – p. 210 – 211.
- ☐ C. Planning For Better Living: Three questions to answer that will help you stay on track – p. 211 – 212.
- ☐ D. Planning For Better Living: Four final questions to answer that will help you organize your back pain goals – p. 212 – 213.

Date Chapter Completed: _____ /_____ /_______

Appendix – The American Chronic Pain Association's Ten Steps for Dealing with Pain (p. 215 – 217)

Index (p. 219 – 224)

Week 12 – Expect a phone call from study staff this week!

© Boston Medical Center
Veterans Back to Health Trial

Veterans Back to Health

Education Newsletter Volume 1

How to Safely Resume Your Normal Activities

**What should I do if I'm having severe pain?**	Use gentle stretching. This will help you reduce the tension in your back.
**What if I am having a severe flare-up and it is too painful to move?**	You may find that using medication, and either heat or ice, makes it easier to stretch and get moving again.
**What should I be doing as my severe pain gets better?**	Continue with your normal activities within 1 to 3 days as your worst pain gets better. Try to keep up with as many household chores, family outings, and other routine activities as possible.
**Because of my back pain, should I be doing fewer activities?**	No. You should gradually increase your activities over a few days or up to a week.
**What if an activity increases my pain throughout the day?**	If an activity causes increased pain for the rest of the day, do less of the activity or find a substitute.
**When I have a flare-up, I like to stay in bed or frozen in one position.**	Do not do this! Movement, even if uncomfortable, can help you get through a flare-up quickly. You can start by walking within your home. Try walking for a few minutes every hour.
**So I should push through the pain?**	Not necessarily. Pace yourself and use common sense. Give yourself permission to take it easy for a few days until the worse pain goes down. Pushing through severe pain might trigger a second back spasm.
**After a flare-up, should I do as little as possible?**	No. Doing too little may lead to a longer period of pain. Pacing yourself means finding out how much of an activity you can tolerate, and then performing that much on a consistent basis.

© Boston Medical Center
Veterans Back to Health Trial

Education Newsletter Volume 2

Effective Self Care

4 Steps to Making a Plan of Action

1. **Exactly what you are going to do:** For example, is your plan to start a walking program, do stretching exercises, or perhaps begin to do social activities that you have been putting off?
2. **How much you will do:** For example, will you walk for 15 or 50 minutes?
3. **When will you do it:** Will you walk before breakfast, during your lunch break, or at night after the evening news?
4. **How often you will do the activity:** It is best to say that you will do something five times a week rather than every day? That way, if you miss the activity one day you will not feel guilty or feel like you have failed.

The Three R's for Managing Flare-Ups

RELIEVE severe pain and inflammation during a flare-up.

RESUME your normal activities as the flare-up goes down. Do not wait until you are free of pain; begin to stretch, exercise, and take up your normal activities.

RESTORE your self-confidence and emotional well-being. It is important to identify and address the worry, distress, and negative thoughts that come with a severe episode of back pain.

How to Talk to Your Doctor about Back Pain

- Be specific when you talk to your doctor about what is worrying you.
- Describe what the pain is preventing you from doing.
- Tell your doctor whether you want a prescription pain reliever.
- Have realistic expectations.
- Ask questions.
- If you are interested, ask for information about other pain-reducing techniques.
- Tell your doctor about any pain control methods you may already be using.

Tips for Taking Over-the-Counter Medicine

(more tips listed on page 66)

- Choose medicine with a single ingredient, for example, some pain relievers have caffeine in them, which can disrupt your sleep.
- Read the label carefully.
- Ask the pharmacist for help.
- Avoid mixing medicines.
- Do not exceed the recommended dosage.
- Never use a drug from an unlabeled container.

© Boston Medical Center
Veterans Back to Health Trial

Education Newsletter Volume 2

Managing Flare-Ups and Emergencies

**Do not panic** ⬇	Avoid negative thoughts and don't get upset.
**Find out whether the problem is a serious risk to your health** ⬇	Read the "Red Flags of Serious Illness" on page 17. If you have any of these symptoms, contact your doctor and the study staff as soon as possible.
**Control your pain** ⬇	If you are in severe pain, take medicine right away.
**Ice the painful area** ⬇	Whether you are having back or leg pain, use ice on the lower back. Do not use heat because it might increase swelling.
**Take it easy** ⬇	It is ok to lie down and rest for a while but do not stay in bed any longer than necessary. Try to get up and move around after 30 to 60 minutes.
**Use relaxation, distraction, and reassuring self-talk** ⬇	Try meditation or focused breathing. Go to chapter 9 to learn these methods.
**Slowly start to move** ⬇	The sooner you start to move, the better. If you are in too much pain to move at first, try again after taking pain medicine and using ice.
**Stretch** ⬇	Stretching will help you get through a flare-up faster, so begin gentle stretching as soon as possible. Expect some discomfort, but avoid stretching that causes severe pain.
**Keep up your strength and endurance** ⬇	Within a couple of days you should be able to return to some light strengthening and endurance exercises.
**Continue aerobic exercise** ⬇	Once the worst of your pain has decreased, you can start with walking on level ground at a pace and distance that is comfortable to you.
**Ways to control pain**	After you have recovered, it is helpful to keep using over-the-counter medications, heat, ice, and other methods. You are also better off going to work and doing your normal activities.

© Boston Medical Center
Veterans Back to Health Trial

Education Newsletter Volume 2

Physical Methods for Back Pain

- Exercise: The best for managing back pain.
- Applying Cold and Heat: For a sudden flare-up, applying ice is the most helpful. If ice does not work for you, try using a heating pad.
- Massage: A gentle, stroking massage is the safest.
- Spinal Manipulation and Mobilization: Performed by chiropractors and osteopaths.
- Traction, "Deep Heat," and Other Techniques: read about other pain control techniques on pages 80 – 81.

Mind-Body Techniques

- Breathe Deeply to Relieve Stress: Relaxing to reduce stress can lower muscle tension and back pain.
- Progressive Muscle Relaxation: Helps you find muscle tension before it builds to pain. Learn how to do this at the bottom of page 87, continuing on pages 88 – 89.
- "Letting Go" Relaxation: Similar to progressive muscle relaxation, but it does not require you to tighten your muscles. Learn how to do this on pages 89 – 90.
- Meditation and Prayer: Involves concentrating on your breathing and repetition of the special word or phrase.
- Guided Imagery: Similar to daydreaming. Close your eyes, and ask a family member or a friend read the script on pages 91 – 92.

Using Deep Breathing as a Relaxation Tool

- Make time for deep breathing: It only takes a few minutes each day.
- Get as comfortable as possible: Sit in a chair with back support or lie comfortably on the floor or bed.
- Monitor your breathing: Place one hand on your stomach and one on your chest. Practice breathing until the hand on your stomach moves more than the one on your chest.
- Let go of tension: Inhale through your nose and exhale through slightly puckered lips. Notice that you feel more relaxed as you exhale.

Tips for Coping with Depression

- Visit or call friends, go on outings, join a group, or volunteer
- Make plans and carry them out
- Remain as active and involved in normal activities as possible
- Exercise regularly
- Go to work, accomplish things at home, or spend time on a hobby
- Reward yourself when you accomplish something
- Have fun
- Avoid alcohol
- If you are taking medicine, talk to your doctor about any possible side effects
- Seek professional help

© Boston Medical Center
Veterans Back to Health Trial

Education Newsletter Volume 3

Physical Activity and Exercise

Four Basics to Good Mechanics

1. When lifting or carrying something, keep the weight close to your body
2. Keeps your ears, shoulders, and hips in line
3. Keep your back straight and do not slouch
4. Try not to twist your back

Take a look at the pictures and descriptions of how to perform everyday activities on pages 126 – 137.

Activities include:

- Waking up, using the sink, and getting dressed – pages 126 – 129
- Sitting and standing up – pages 129 – 132
- Lifting, pulling, pushing and reaching – pages 132 – 135
- Cooking, cleaning, outdoor activities (like shoveling snow) – pages 136 – 137

Exercises for Building Strength and Endurance

Take a look at the pictures and descriptions of simple floor exercises for your back pain on pages 154 – 159.

Six Steps for Finding Time for Exercise

1. Write down your daily routine
2. Find activities that can be combined with some type of exercise
3. Find times when you are not busy
4. Find activities you might be willing to give up for exercise
5. Think about how you relax after a busy day
6. Set aside a special time to exercise during the day

Benefits of Stretching

- Stretching can reduce pain: This is probably the main reason people with back pain stretch regularly.
- Stretching prevents muscle strains and pulls: If you have good flexibility, you can more safely move through a wider range of motion.
- Flexibility helps you to keep a neutral position during your daily activities: Leg and hip flexibility will give your spine the most support against gravity and will give you the best protection from injury, tiredness, and pain.

Tips for Increasing Strength and Endurance

- Do the exercises at least two times each week
- Use strength-training exercises before you do aerobic exercises
- Warm up your muscles
- Start by doing as many repetitions as you can and add more as you get stronger
- Breathe naturally and smoothly
- Build endurance as well as strength
- Use your trunk muscles to support your back and stay within your comfort zone when exercising
- Stop exercising if you become significantly tired
- Slowly increase the exercise time and intensity
- Challenge yourself to do more

© Boston Medical Center
Veterans Back to Health Trial

Veterans Back to Health

Education Newsletter Volume 4

Everyday Insights for Better Living

How to Re-Establish a Normal Sleeping Pattern

- Set a regular waking time and stick with it
- Set a time for going to bed
- Try not to vary your schedule from day to day
- Avoid daytime naps
- Re-evaluate how much sleep your body needs
- Sleep better by spending less time in bed

How to Prevent Future Sleep Problems

- Avoid caffeine
- Avoid alcohol
- Exercise regularly
- Take time to slow down as bedtime approaches
- Do not lie awake at night
- Find another time for worrying and planning
- Do not overuse sleeping pills

How to Avoid Problems in Your Relationships

- <u>Focus on others</u>: instead of your back
- <u>Pay attention</u>: to the ways your back problem causes distress for others
- <u>Notice any sacrifices</u>: others have made for you and acknowledge them
- <u>Ask about the thoughts and feelings</u>: of your family and friends
- <u>Remain as active and self-reliant as possible</u>
- <u>Interact with others</u>: just as you would if your back was not a problem
- <u>Avoid taking out your frustrations</u>: on others
- <u>Tell others directly</u>: if you occasionally need their help, or if you do not need their help, with a task that they offer to do for you
- <u>Do not withdraw from life</u>: Maintain your normal social interactions

Tips for Workers with Back Pain

- Use good body mechanics
- Pace yourself
- Know your limitations
- Design your workstation to minimize the use of poor posture and body mechanics
- Discuss any potential risk of injury with your boss
- When flare-ups occur, try to keep working, even if it is at a modified job
- If you're not well enough to go to work, maintain contact with your boss and coworkers
- When you are looking for a new job, it is usually best to present yourself as healthy and capable, as long as you are sure you can perform the job

The American Chronic Pain Association's Ten Steps for Dealing with Pain

1. Accept the Pain
2. Get Involved
3. Learn To Set Priorities
4. Set Realistic Goals
5. Know Your Basic Rights
6. Recognize Your Emotions
7. Learn To Relax
8. Exercise
9. See Total Picture
10. Reach Out

© Boston Medical Center
Veterans Back to Health Trial

Qualitative Interview Procedures

Veterans Back to Health

5.0

© Boston Medical Center
Veterans Back to Health Trial

Qualitative Overview

We will conduct qualitative interviews, pre- and post-intervention, with a subset of the Veterans in the yoga group to explore any association of chronic low back pain (cLBP) and co-morbid mental health disorders with family functioning. This will also give us a greater detailed understanding of yoga's impact on the Veteran and possibly the Veteran's family.

After randomization, a purposive sample of 20 Veteran participants with partners in the yoga arm will be recruited to participate. To be eligible for interviews, Veterans must have a cohabitating or long-term committed partner/spouse by self-report. In addition, both the Veteran and the Veteran's partner must give consent in order to participate in interviews.

Separate and conjoint interviews will be conducted by trained researchers from Boston University School of Social Work (BUSSW) with the Veteran and his/her partner prior to participation in yoga and again after ending classes (80 individual interviews total). Interviews will be conducted with members of the couple concurrently followed by a short conjoint interview, if both members are able to attend at the same time. Semi-structured interview protocols will focus on perception of impact of cLBP on Veteran, perception of Veteran's cLBP on overall family mood and functioning, attitude toward yoga, perception of change in Veteran's cLBP perception of any possible change in Veteran's psychological concerns after yoga, and perception of any change in overall family mood and functioning after yoga.

If a couple drops out from the qualitative sub-study during the intervention (i.e., after pre-intervention interview), we will attempt to replace that couple with another couple to complete a post-intervention interview in order to maintain an N of 20 dyads with pre- and post-interviews. If a couple drops out prior to the initial interview, we will attempt to replace that couple with a new couple.

© Boston Medical Center
Veterans Back to Health Trial

Qualitative Flow Sheet

Events	Relevant Documents and Forms
**Randomization to Yoga**	• Qualitative Recruitment Script<br>• Interview Information Sheet
**Initial Interview**	• Veteran and Partner Qualitative ICFs<br>• Corresponding Interview Guide(s)<br>• Interview Progress Notes<br>• Qualitative Honoraria Form
**Post-Yoga Interview**	• Corresponding Interview Guide(s)<br>• Interview Progress Notes<br>• Qualitative Honoraria Form

END OF QUALITATIVE SUB-STUDY

© Boston Medical Center
Veterans Back to Health Trial

Qualitative Recruitment Script

Overview

Once participants have been randomized to the yoga group and have received information about their group, the unblinded staff member will determine if they are considered partnered based on their responses on the baseline survey. If they are partnered, the unblinded staff member will present the qualitative sub-study using the following script and the Interview Information Sheet.

Suggested Script

We are interviewing some yoga participants to better understand and treat chronic low back pain. We are very interested in understanding your thoughts on how back pain has impacted you and your family and your thoughts about yoga. We are interested in speaking with both you and your partner because we know that when one person has a chronic health issue it may also impact their partner and their family.

To do this, we would like to invite you and your partner to take part in the interviews. Participation includes completing in-person interviews with a member of our research team before yoga class starts and again after 12 weeks of yoga. Interviews will last about 60-90 minutes each time. Each interview will have an individual part and a part together with your partner. The interviews will be more of a discussion with the research team member of your experiences with chronic low back pain and your thoughts about yoga for back pain. You and your partner will each be compensated $35 for each interview, which includes compensation for your time and also for travel.

Does this sound like something you might be interested in doing?

If yes:

> *Great! We would like you to ask your partner if he/she is also interested. We would like you to share this information sheet about the interviews with your partner. We will call you in a few days if we haven't heard from you yet to find out if you and your partner would like to participate. If you have any questions or would like to get in contact with us, our phone number is here on the information sheet.*

If no:

> *Okay, thank you for listening. May I ask why?*

© Boston Medical Center
Veterans Back to Health Trial

Back to Health

Interview Information Sheet

What is the *Back to Health* Study?

- *Back to Health* is a 6-month study for Veterans with chronic low back pain.
- Participants in the yoga group attend 75-minute group classes once each week at the Bedford VA Hospital for 3 months.
- We are interviewing yoga participants and their partners to learn more about the relationship between low back pain, emotional health, and family functioning. We are also trying to understand if yoga impacts Veterans and their families.

How the Interviews Work

- We will ask Veterans and their partners to participate in an interview before yoga classes begin and again after 12 weeks of yoga.
- Trained researchers will be conducting individual interviews with you and a separate interview with your partner. The interviews will be about 60-90 minutes. Then we will ask you and your partner to do a short interview together.
- We will ask to audio record these sessions. These recordings will not be associated with your name and only research staff will have access to these recordings.
- All interviews will take place at a time and place that is convenient for you. Interviews will be scheduled ahead of time. We hope that there is a time that both you and your partner can come to the interviews at the same time.
- You have the right to refuse to take part in an interview. Your participation is voluntary. You do not have to answer any questions that you don't want to. You may take breaks during the interviews or stop an interview altogether at any time.

Compensation for Your Time, Travel, and Effort

- You and your partner will both receive compensation for each interview visit you participate in. We provide $25 for your time and effort plus $10 for travel, if applicable.

PLEASE CALL US AT 781-687-2000 ext.5520 IF YOU HAVE ANY QUESTIONS OR WOULD LIKE TO PARTICIPATE IN THESE INTERVIEWS.

WE WILL FOLLOW-UP WITH YOU IN A FEW DAYS TO TALK MORE ABOUT THE INTERVIEWS.

© Boston Medical Center
Veterans Back to Health Trial

Detailed Qualitative Procedures

After Randomization

Unblinded Bedford VA research staff will communicate directly with BUSSW qualitative staff after introducing the qualitative sub-study to a potentially interested participant. BUSSW staff will contact the Veteran after about 3 days to find out if he/she has discussed the interviews with his/her partner. Bedford VA research staff will notify BUSSW staff if the Veteran calls regarding the interviews sooner than 3 days after randomization.

Scheduling Interviews

If the Veteran and partner would like to participate in interviews, BUSSW staff will review the information on the Interview Information Sheet and will make sure the Veteran and partner have an opportunity to ask questions before scheduling the interviews. Staff will reiterate that the couple will be contacted again to complete a second set of interviews after 12 weeks of yoga.

Interviews will be scheduled at the convenience of the Veteran and partner and will take place at a location most convenient to the participants, keeping in mind privacy needs. If the Veteran and partner are not able to attend interviews at the same time, separate interviews will be conducted within approximately 72 hours. Staff will attempt to complete all pre-yoga interviews within 2 weeks of the baseline survey.

Conducting Interviews

Prior to the interviews, the Veteran and partner will be consented using the Bedford VA Veteran Qualitative ICF and the Partner Qualitative ICF, respectively. BUSSW staff will obtain verbal and written consent after explaining the qualitative interviews, risks, benefits, privacy, and other information. The Veteran and partner will be asked to give permission for audio recording of the interviews. HIPAA consent will also be obtained using the HIPAA Authorization Form.

Individual interview will be conducted simultaneously by two separate interviewers whenever possible. After the individual interviews, the Veteran and partner will be brought together for a short conjoint interview to be conducted by one or both of the interviewers who conducted the individual interviews. These will also be recorded if both the Veteran and partner consent to audio recording.

After completion of each interview, staff will complete an Interview Progress Note to document that the interview occurred and any relevant information about the interview. Progress notes will also be entered into the Veteran participant's Bedford VA medical record. Paper progress notes will be filed by research staff in the participant's file. These files will be stored in a locked file cabinet in a locked office at the Bedford VA. Only research staff will have access to these files.

Compensation

Gift cards will be given to interviewees after completion of interviews. Each interviewee will receive a $25 honoraria after each completed interview. Each interviewee will also receive $10

© Boston Medical Center
Veterans Back to Health Trial

travel compensation if the interview takes place outside of their home. Interviewers will have participants sign the Qualitative Honoraria Form that contains the date, gift card number, gift card amount, and participant name. The interviewer will also sign the form as a witness. BUSSW staff will be responsible for tracking all gift cards given to participants for qualitative interviews in accordance with Bedford VA, BUMC, and BUSSW policies.

Transcription

Audio recordings of interviews will be transcribed in one of two ways:

1. Transcription by research staff. BUSSW research staff will upload the audio recordings to a secure VA SharePoint site for transcription. Research staff with access to this site will transcribe the audio recordings and retain all transcription documents on the secure VA network.
2. Transcription via VA-approved transcriptionist. BUSSW research staff will upload the audio recordings to a secure VA SharePoint site for transcription. Staff will then contact the transcription service by email to tell them the audio recordings are available on SharePoint for transcription. The transcription service will notify the appropriate staff member when transcription is complete.

© Boston Medical Center

Qualitative Interview Guides

Interview Guide for Veteran's Partner

INTRODUCTION

Suggested Script: *I would like to thank you for coming today to share your experiences, thoughts, and opinions with us. We are looking for your feedback in order to improve the way we study and treat low back pain. This is an open discussion and everything you share with me today is valuable. If there are questions that don't feel relevant for you, please feel free to let me know. If there are any topics that we don't touch on, but that you feel are relevant, please feel free to let me know at the end of the interview. This discussion will be recorded; however, we will make sure to keep this recording confidential and for use by our study staff only.*

PRE-INTERVENTION QUESTIONS

1. Please tell me a bit about your family.
    a. Who are the members?
    b. What are their ages?
    c. Do they live in your home?
    d. How do they spend their time?

2. Please tell me about [Veteran's] experience with back pain. *(Let respondent tell the "story" of their partner's experience with pain. Gently probe for severity, his/her perception of ways it might interfere with typical activities, ways it might interfere with interactions with family members or friends, ways it might impact mood, etc.)*
    a. What's a typical day like?
    b. What was a typical day like before he/she experienced pain?
    c. How does he/she tend to accommodate to the challenge presented by the pain?
    d. Are there activities he/she is unable to do because of the low back pain?

3. How does [Veteran's] back pain impact his/her mood? Can you give me an example?
    a. How do you think his/her back pain impacts your own mood? In what way? Can you give me a specific example?
    b. What different factors influence how his/her back pain impacts your mood? (probe for severity of pain, stressful interactions, etc.) Can you think of specific examples?

4. How does [Veteran's] back pain impact his/her functioning? (*Respondent may feel that he/she has already answered this question so you should explain that you may ask some questions in a number of different ways*)
    a. How does the impact of [Veteran's] back pain affect <u>your</u> functioning? Can you think of an example?
    b. What factors influence how [Veteran's] back pain impacts your functioning? Can you tell me about a specific example or situation where one of these things happened? How would [Veteran] respond to this question about factors that influence your functioning due to back pain? What example would he/she share?

© Boston Medical Center
Veterans Back to Health Trial

5. What impact do you think [Veteran's] back pain has on your family's mood and functioning?
    a. What would another family member (*specify who*) say in response to this question?
    b. What factors do you think influence how [Veteran's] back pain impacts the family's mood and functioning? Can you think of a specific example or situation where one of these things happened? What example might another family member give in response to this question?

6. Have you ever taken a yoga class or done yoga? What was that like for you?
    a. How do you feel about yoga as a treatment for low back pain?
    b. How do you think yoga influences back pain?
    c. What other physical changes do you think could be influenced by yoga? How?
    d. What psychological or emotional changes do you think could be influenced by yoga? How?

7. Has [Veteran] ever taken a yoga class or done yoga? How do you think he/she feels about yoga overall?
    a. How do you think he/she thinks about yoga as a treatment for back pain?
    b. What do you think he/she believes about psychological or emotional changes from yoga?

POST-INTERVENTION QUESTIONS

1. Please tell me about [Veteran's] experience in the yoga class or doing yoga at home?
    a. Are there one or two experiences that stand out?
    b. Can you tell me about those times?
    c. Why do you think those experiences stand out for you?

2. How do you think [Veteran's] back pain has changed as a result of the yoga class?
    a. How does this compare to what he/she expected?
    b. Do you think there have been any other physical changes for him/her?
    c. How do you perceive changes in your partner due to the yoga class?
    d. Are there one or two examples that come to mind?

3. How do you think [Veteran's] psychological or emotional status has changed as a result of the yoga class?
    a. Can you give me an example of a psychological or emotional change?
    b. Do you think he/she has experienced changes in anxiety? Depression? Post-traumatic stress symptoms?
    c. Do you think the yoga class has had an impact on your relationship? In what ways? Can you give me one or two examples?

© Boston Medical Center
Veterans Back to Health Trial

d. Do you think [Veteran’s] changes in mood or emotional status has changed your mood? If so, how? Can you think of an example?

4. How do you think your family’s overall mood or function has changed as a result of yoga?
   a. How do you think [Veteran’s] back pain changes have influenced your family? Can you give me an example?
   b. How do you think [Veteran’s] psychological changes have influenced your family? Can you give me an example?

<u>CONCLUSION</u>

Is there anything else you want to talk about that we haven’t already discussed or that you think it would be helpful for us to know about?

© Boston Medical Center
Veterans Back to Health Trial

Qualitative Interview Guides

Interview Guide for Veteran

<u>INTRODUCTION</u>

<u>Suggested Script:</u> *I would like to thank you for coming today to share your experiences, thoughts and opinions with us. We are looking for your feedback in order to improve the way we study and treat low back pain. This is an open discussion and everything you share with me today is valuable. If there are questions that don't feel relevant for you, please feel free to let me know. If there are any topics that we don't touch on, but that you feel are relevant, please feel free to let me know at the end of the interview. This discussion will be recorded; however we will make sure to keep this recording confidential and for use by our study staff only.*

<u>PRE-INTERVENTION QUESTIONS</u>

1. Please tell me about your experience with back pain. (*Let respondent tell the "story" of their experience with pain. Gently probe for severity, ways it might interfere with typical activities, ways it might interfere with interactions with family members or friends, ways it might impact mood, etc.*)
    a. What's a typical day like?
    b. What was a typical day like before you experienced pain?
    c. How do you tend to accommodate to the challenge presented by the pain?
    d. Are there activities your miss that you are unable to do because of the low back pain?

2. How does your back pain impact your mood? Can you give me an example?
    a. What would your partner say in response to this question? How does he/she think your back pain impacts your mood?
    b. How do you think your back pain impacts your partner's mood? In what way? Can you give me a specific example?
    c. What different factors influence how your back pain impacts your mood? (*probe for severity of pain, stressful interactions, etc.*) Can you think of specific examples? How would your partner respond to this question about what back pain factors influence your mood?

3. How do you think your back pain impacts your functioning? (*Respondent may feel that he/she has already answered this question so you should explain that you may ask some questions in a number of different ways*)
    a. What does your partner think about the way your back pain impacts your functioning? Can you think of an example he or she would share?
    b. What factors influence how your back pain impacts your functioning? Can you tell me about a specific example or situation where one of these things happened? How would your partner respond to this question about factors that influence your functioning due to back pain? What example would he/she share?

© Boston Medical Center
Veterans Back to Health Trial

4. What impact do you think your back pain has on your partner's mood and functioning?
    a. What would he/she say in response to this question?
    b. What factors do you think influence how your back pain impacts your partner's mood and functioning? Can you think of a specific example or situation where one of these things happened? What example might your partner give in response to this question?

5. What impact do you think your back pain has on your family's mood and functioning?
    a. What would a family member (*specify who*) say in response to this question?
    b. What factors do you think influence how your back pain impacts the family's mood and functioning? Can you think of a specific example or situation where one of these things happened? What example might a family member give in response to this question?
    c. Have you ever taken a yoga class before or done yoga? What was that like for you? How do you feel about yoga as a treatment for low back pain?
    d. How do you think yoga may influence low back pain?
    e. What other physical changes do you think could be influenced by yoga? How?
    f. What psychological or emotional changes do you think could be influenced by yoga? How?

6. Has your partner ever taken a yoga class or done yoga?
    a. How do you think he/she feels about yoga overall?
    b. How do you think he/she thinks about yoga as a treatment for low back pain?
    c. What do you think he/she believes about psychological or emotional changes from yoga?

<u>POST-INTERVENTION QUESTIONS</u>

1. Please tell me about your experience in the yoga class?
    a. Are there one or two experiences that stand out?
    b. Can you tell me about those times?
    c. Why do you think those experiences stand out for you?

2. How do you think your back pain has changed as a result of the yoga class?
    a. How does this compare to what you expected?
    b. Do you think there have been any other physical changes?
    c. How do you think your partner perceives changes due to the yoga class?
    d. Can you tell me a bit about your partner's perceptions? Is there one example that comes to mind?

3. How do you think your psychological or emotional status has changed as a result of the yoga class?
    a. Can you give me an example of a psychological or emotional change?

© Boston Medical Center
Veterans Back to Health Trial

b. Do you think you have experienced changes in anxiety? Depression? Post-traumatic stress symptoms?
c. Do you think the yoga class has had an impact on your relationship with your partner? In what ways? Can you give me one or two examples?
d. What would your partner say about psychological or emotional changes for you as a result of the yoga class?
e. Can you give me one example that your partner might be thinking about?

4. How do you think your family's overall mood or function has changed as a result of yoga?
   a. How do you think your low back pain changes have influenced your family? Can you give me an example?
   b. How do you think your psychological changes have influenced your family? Can you give me an example?
   c. What would your partner say about the impact of your change in low back pain or psychological change on your whole family mood or function?
   d. Do any examples come to mind?

CONCLUSION

Is there anything else you want to talk about that we haven't already discussed or that you think it would be helpful for us to know about?

© Boston Medical Center
Veterans Back to Health Trial

Interview Guide for Brief Conjoint Interview

INTRODUCTION

Suggested Script: *Thank you again for coming in today to share your experiences, thoughts, and opinions with us. We would now like to continue our discussion together to revisit some of the areas that were already discussed. We are specifically interested in understanding more about how you manage back pain together as a couple. As we went over previously, if there are questions that don't feel relevant for you or any topics that we don't touch on but that you feel are important, please let me know. You do not have to answer any question you feel uncomfortable with. If at any time you would like to take a break during the interview or stop the interview, just let me know. This discussion will be recorded but will be kept confidential and used by study staff only.*

PRE-INTERVENTION QUESTIONS

1. We talked about your day to day experience with back pain. We wanted to circle back to understand the role that back pain takes during a typical day (in activities you do together, chores, managing work, running the home, etc). In what ways are things different now compared to before [Veteran] experienced back pain?

2. We talked about how back pain impacts mood. We wanted to revisit that and understand how that impacts you as a couple (e.g., [Veteran's] mood, interactions as a couple). We also wanted to understand what different factors might be at play (e.g., pain level impacting activities that in turn impacts mood). Can you think of an example to share about this?

3. We talked about how back pain impacts functioning. Can we talk some more about how the ability to function impacts you as a couple? Can you think of an example to share?

4. We talked about how back pain impacts your family's functioning. We're interested to hear more about how family functioning impacts your relationship as a couple. Can you think of an example to share?

5. We talked about how low back pain impacts your family's mood. Can you talk some more about how functioning/back pain impacts your mood together? Can you think of an example to share?

6. We talked about your previous experience(s) with/impressions of yoga. We were interested to learn if you knew other people who had taken yoga and what you previously thought as a couple about yoga's ability to influence back pain and other physical/emotional changes? How did your thoughts about yoga impact [Veteran's] participation? Can you think of an example to share?

© Boston Medical Center
Veterans Back to Health Trial

Qualitative Interview Guides

POST-INTERVENTION QUESTIONS

1. We talked about [Veteran's] experience in the yoga class (and/or practicing yoga in the home) and we're interested to hear more about one or two experiences that stand out in your minds around that.

2. We already discussed the ways [Veteran's] back pain may have changed as a result of participating in the yoga classes. Can you talk a bit more about how that may have compared to your expectations before the class? Any other physical changes? Other types of changes? Are there one or two examples you might want to share to explain these changes?

3. We talked about how [Veteran's] mood may have changed as a result of yoga class. Can you talk a bit more about one or two examples of these changes (including any changes in anxiety, depression, post-traumatic stress symptoms, or overall mood)? Can you also talk a bit more about any ways you think yoga has impacted your relationship?

4. We wanted to revisit our earlier discussion and hear a bit more about any changes in your family's overall mood or functioning as a result of yoga's impact on you both as a couple.

CONCLUSION

Is there anything else you want to talk about that we haven't already discussed or that you think it would be helpful for us to know about?

© Boston Medical Center
Veterans Back to Health Trial

Interview Progress Note

SID: ___________

PART I: Completion of Informed Consent

Date of Informed Consent: ____________________

Did the participant sign the consent form and write the correct date? ☐ YES ☐ NO

Notes about consent process, areas of concern, etc: ____________________________________

____

____

PART II: Pre-Intervention Interview

Did the participant complete an individual interview? ☐ YES, on ______________ (MM/DD/YY) ☐ NO

Did the participant complete a conjoint interview? ☐ YES, on ______________ (MM/DD/YY) ☐ NO

Notes about interview(s), e.g., reason why interview(s) not completed, any questions the participant refused to answer, important events during the interview process, etc:

____

Name of Interviewer — Signature of Interviewer — Date

PART III: Post-Intervention Interview

Did the participant complete an individual interview? ☐ YES, on ______________ (MM/DD/YY) ☐ NO

Did the participant complete a conjoint interview? ☐ YES, on ______________ (MM/DD/YY) ☐ NO

Notes about interview(s), e.g., reason why interview(s) not completed, any questions the participant refused to answer, important events during the interview process, etc:

____

Name of Interviewer — Signature of Interviewer — Date

© Boston Medical Center
Veterans Back to Health Trial

Qualitative Honoraria Form

Participant Name: ____

Interview Type

☐ Pre-Yoga Interview

☐ Post-Yoga Interview

Gift Card Information

Card Number: _________________________________

Amount: $_________________

Signature of Participant: ____ Date: ______________

Signature of Staff: ____ Date: ______________

© Boston Medical Center
Veterans Back to Health Trial

Drop-Out Procedures

Procedures have been developed for multiple drop-out scenarios in order to maintain the sample size of N=20 couples. These scenarios are described below:

1. **The Veteran and/or partner consent to participate in interviews but change their mind(s) before the interviews are conducted.** Staff will compensate the Veteran and partner for travel expenses but will not provide them with honoraria. The reason(s) that the interviews were not completed will be recorded on the Interview Progress Note to be filed in the participant's file. BUSSW staff will then attempt to replace the couple with another couple to complete interviews before the yoga intervention and after 12 weeks of yoga classes.

2. **The Veteran and/or partner complete pre-yoga interviews but do not want to/are unable to return for a post-yoga interview.** BUSSW qualitative research staff will make all reasonable efforts to contact the Veteran and partner to schedule post-yoga interviews. If the attempts are not successful or if either member of the couple indicates that they are not interested in completing additional interviews, staff will consider the couple "dropped-out" of the qualitative sub-study. The reason(s) that the interviews were not completed will be recorded on the Interview Progress Note to be filed in the participants' respective files. Staff will attempt to replace the couple with another Veteran in the yoga group and his/her partner to complete a post-yoga interview visit.

3. **The Veteran discontinues the yoga intervention and is still willing to be contacted by research staff.** BUSSW qualitative staff will make all reasonable efforts to contact the Veteran and partner to schedule post-yoga interviews. If the couple agrees to attend an interview visit, the interviewer will attempt to explore the reasons the Veteran discontinued participation in the yoga intervention. If attempts to contact the couple are not successful or the couple indicates that they would not like to attend interviews, staff will consider the couple "dropped-out" of the qualitative sub-study. The reason(s) that the interviews were not completed will be recorded on the Interview Progress Note to be filed in the participants' respective files. Staff will then attempt to replace the couple with another yoga participant and his/her partner to complete a post-yoga interview visit.

4. **The Veteran discontinues the yoga intervention and is not willing to be contacted by research staff.** If the Veteran discontinues the yoga intervention and indicates that he/she would not like further contact from research staff, all efforts will be made to recruit another yoga participant and his/her partner to complete a post-yoga interview visit. The reason(s) that the interviews were not completed will be recorded on the Interview Progress Note to be filed in the participants' respective files.

© Boston Medical Center
Veterans Back to Health Trial

Data Collection and Management Procedures

6.0

© Boston Medical Center

Data Overview

Male and female Veterans age 18 years or older with cLBP will be recruited for the study. Block stratified randomization will be used to assure similar numbers of Veterans in both treatment groups that are ≤45 years old, experience post-traumatic stress symptoms (PCL≥30), and have a partner (spouse, live-in partner, or live-out committed relationship).

The co-primary outcomes will be pain intensity and function, measured by the Defense and Veterans Pain Rating Scale (DVPRS) and modified Roland Morris Disability Questionnaire (RMDQ), respectively. Secondary outcomes will include pain medication use, health-related quality of life, a range of psychological measures targeting key mental-health symptoms facing Veterans, and 24-week intermediate-term follow-up data. Other secondary outcomes will include the minimal dataset recommended by Report of the NIH Task Force on Research Standards for Chronic Low-Back Pain. Cost-effectiveness analyses will be performed from the perspective of society, VHA, and the Veteran.

Participants will fill out paper surveys at baseline, 6 weeks, 12 weeks, and 24 weeks. Post-baseline surveys may be completed over the telephone if paper administration is not possible. Additional treatment-specific data will be collected including education adherence data, yoga attendance rates, and yoga home practice.

Policies and procedures regarding all data collected during the screening phase (i.e., prior to the baseline survey) is discussed in the Recruitment Procedures section of this manual. Detail about treatment-specific data is discussed in the respective treatment sections of this manual.

Staff Blinding

Activity	Blinded Staff	Unblinded Staff
Eligibility Screening	✓	✓
Obtain Informed Consent	✓	✓
Baseline Survey Administration	✓	✓
-- RANDOMIZATION --		
Check-in & Reminder Calls		✓
Scheduling Survey Administration		✓
Collecting Surveys*	✓	✓
Update Medications Form	✓	✓
Administer AE & Cost Questionnaire		✓
Survey Double Data Entry	✓	
Home Practice Log Data Entry		✓

* Surveys are filled out by participants using pen and paper. Research staff are in nearby rooms and periodically check-in while participants complete these surveys but are not routinely involved in answering the questions. Thus, knowledge of treatment arm does not have the potential to bias responses during survey collection.

© Boston Medical Center
Veterans Back to Health Trial

Data Flow Sheet

Events	Relevant Documents and Forms
**Eligibility Screening**	• Eligibility Screening Questionnaire*
**Enrollment**†	• ICF/HIPAA Authorization<br>• Informed Consent Progress Note
**Eligibility Verification**†	• Eligibility Verification Checklist*
**Baseline Survey**	• Baseline Questionnaire & Forms<br>• Survey Administration DOs & DON'Ts<br>• Honoraria Signature Form
**Randomization**	• Randomization and Enrollment Form
**6-week Survey**<br>**12-week Survey**<br>**24-week Survey**	• Corresponding Questionnaire & Forms<br>• Survey Administration DOs & DON'Ts<br>• Honoraria Signature Log
**End of Study**	• Study Completion Form

* Form is filled out on StudyTRAX only.
† This event is discuss in detail in another section of this manual.

© Boston Medical Center
Veterans Back to Health Trial

Survey Administration Procedures

Overview

Participants will fill out paper surveys at preset times in private rooms at the Bedford VA during data collection weeks. Surveys should be completed during a 14-day window that includes the designated survey week and the week after. Exceptions to the 14-day window may only be allowed at PI discretion. Research staff will be available to answer questions and review surveys for completion. Surveys must not take place during the 24-hour period following a yoga class. If a participant is unable to travel to the Bedford VA during the specified data collection week, post-baseline surveys may be completed over the telephone with research staff.

Preparation

The following supplies should be prepared ahead of time as applicable:

- Daily attendance sheet
- Pens
- Data collection forms (with extra copies in case a participant shows up unexpectedly):
  - Paper Surveys
  - Medication Forms
  - Adverse Event and Cost Questionnaire
  - Survey Visit Checklist
- Honoraria Signature Log
- Treatment-related materials, e.g., blank yoga home practice logs, education newsletters, education check-in questions, etc.
- Other information that may have been given out earlier but that participants may need again: study team contact information, education curriculum sheets, etc.

Survey Administration

The diagram below shows the in-person paper survey administration process.

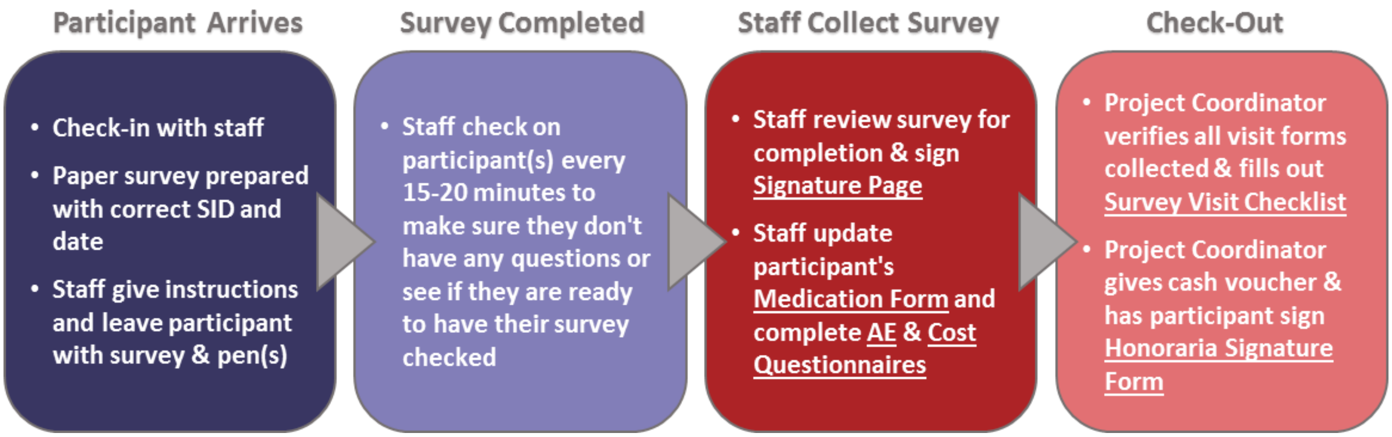

Arrival: Participants should be given detailed information about where to meet research staff. If the location of survey administration is in a section of the building that the participant is not familiar with, staff should have the participant call the study phone line when they are in a central area of the building (e.g., at a reception or information desk) so staff can meet them and escort them to the room where they will complete their survey.

© Boston Medical Center
Veterans Back to Health Trial

Survey Administration Procedures

Once the participant arrives in the location of survey administration, study staff will use the daily attendance sheet to check-in the participant and give them a paper survey with the correct SID written on it. All paper surveys should contain the participant's SID number and date of completion on the top right corner of every page *before* it is given to the participant to complete. The staff member that gives the participant the survey to complete should verify that the SID number written on the survey is correct.

Staff will lead the participant to a private room where they can sit to complete the survey. Staff should make sure the participant knows where to find nearby restrooms and staff in case he/she has any questions or issues. Before leaving the room, staff should make sure there are working pens available for use (NO pencils) and give instructions for completing the survey:

> *This survey asks some information about how you rate your own health and how it affects your daily activities. It should take about 45 to 60 minutes*[*] *to complete. There are no right or wrong answers, just answer each question as best as you can. Please take the time to read the instructions before each section and choose the response that is closest to how you feel. Please remember that your answers are confidential. If you have any questions about the survey, I will be in* [location] *and I will come back to see how you are doing in a little bit.*

Collection: Once a participant completes their survey, a team member will review the form for completeness. All research staff should be familiar with questions that are commonly skipped or misunderstood. If the participant did not answer one or more question(s), first assess if he/she skipped the question(s) by mistake or intentionally. If the question(s) were skipped by mistake, ask them to please respond. Otherwise, follow the below tips:

1. If the participant is uncomfortable with some of the survey questions, tell him/her that it is not a problem and continue reviewing the survey.
2. If a participant intentionally skipped a question but does not offer an explanation right away, ask why he/she had difficulty responding.
3. Help the participant answer the question by slowly re-reading the question and answer choices *verbatim*. Do not rephrase or interpret meaning.[†]
4. If the participant asks what a question or response choice means, encourage him/her to answer the question as best as they can, and let them know that it is okay to interpret the question however makes the most sense to them.
5. If the participant is still unable to answer the question(s), accept the incomplete survey.

If any questions are left blank, staff should make a note on the survey with the **reason, initials, and date.** If questions are left blank without an explanation, it will be assumed staff did not follow the above procedure to assure survey completeness. The blinded staff member that reviews the form for completeness will sign and date the Survey Signature Page.

The Medications Form can be updated by either blinded or unblinded staff. This form is cumulative and is updated at each survey period. After the survey has been reviewed and

---

[*] Surveys at baseline, 12 weeks, and 24 weeks take approximately 45-60 minutes to complete. The 6-week survey is shorter and takes about 20-30 minutes to complete.

© Boston Medical Center
Veterans Back to Health Trial

signed by blinded staff, the participant will meet with unblinded staff to fill out an Adverse Event and Cost Questionnaire. The unblinded staff member will then give the participant any treatment-related supplies or questionnaires. All of these supplemental questionnaires have a Survey Signature Page that must be signed by the administering staff person.

Check-Out: After all the above has been completed, the Project Manager will use the appropriate Survey Visit Checklist to verify all information has been collected for the visit and do a final check of the forms for completeness. The checklist, survey, AE questionnaire, cost questionnaire, and updated medications form. The Project Manager will then give the participant an honoraria voucher and have them sign the Honoraria Signature Form.

DOs and DON'Ts of Survey Administration

DO:
- ✓ Be warm and friendly
- ✓ Encourage participants to read instructions and complete all questions
- ✓ Repeat questions and answer choices *verbatim*
- ✓ Encourage participants to answer questions based on what they think each item means

DON'T:
- ✓ Discuss the participant's health, emotions, or group prior to administering the survey
- ✓ Accept incomplete surveys without first encouraging participants to respond to unanswered questions and/or finding out why questions were left blank
- ✓ Change the wording or response choice of any question
- ✓ Interpret or explain the meaning of questions[†]

Telephone Survey Administration

If a participant is unable to travel to the Bedford VA during the specified data collection week, blinded research staff may administer surveys over the telephone as an alternative. However, participants that complete the survey over the phone must still travel to the Bedford VA to receive their honoraria.

Telephone surveys should be scheduled ahead of time just as in-person surveys are. In cases where a participant is particularly difficult to get ahold of and the timeframe for survey collection is running out, staff should attempt to complete as much of the survey as possible over the phone as soon as they are able to get the participant on the line.

Duties of staff members are unchanged from in-person surveys. At least one blinded and one unblinded staff member must be present for surveys completed by telephone. The blinded staff member will enter survey data directly into StudyTRAX™ in real time.

Follow the below tips for completing a survey over the telephone:

[†]If a participant is not sure what a specific word means, staff can give them the definition of the word using a dictionary. Staff should take care to give the correct meaning and avoid offering interpretation of the context in the survey.

© Boston Medical Center
Veterans Back to Health Trial

Survey Administration Procedures

1. Have the participant's StudyTRAX™ page open before calling them. After they answer the phone and confirm they are able to complete the survey, add the appropriate survey on the *Encounters* page.
2. Follow the scripts on screen, reading questions exactly as written. Save often. If there is a brief delay while the page reloads, you can say, "Please wait one minute while my computer loads."
3. After all questions on the survey have been answered, make sure all the survey section titles are marked with green circles to indicate they are complete. If any are yellow, return to that section and answer any incomplete questions.
4. Update the participant's paper Medications Form.
5. An unblinded staff member will take the phone to complete the Adverse Event and Cost Questionnaire. The unblinded staff member should also verify all visit components have been completed using the Survey Visit Checklist before hanging up the phone. This staff member should also arrange a time for the participant to receive and sign for their honoraria. Ideally, this would occur next time they come to the hospital campus for a different purpose in order to limit transportation costs.

After hanging up the phone, staff must immediately complete the following:

- The staff member that completed the survey will click on *Edit Subject* from the participant's StudyTRAX™ dashboard.
  - Fill in initials and date of the survey under the *Data Entry Sign-in* section. Write 'N/A' in the initials field for double entry of the survey to indicate that there will be no secondary entry.
  - If the participant refused to complete any questions, make a note under *Progress Notes* just as if it were a paper survey (include initials and date).
- Make note on the Survey Visit Checklist that the survey was completed by telephone and attach the completed paper forms. The checklist will still be filed in the participant's folder and the date of the survey will be written on the front of the folder.
- The unblinded staff member will notify other staff when the participant is scheduled to pick up and sign for their honoraria. The participant still must sign the Honoraria Signature Log when they pick up their voucher.

Common Problems and Issues

What if the participant asks for clarification of a question?

- Assist the participant by slowly re-reading the question exactly as it is written.
- If the participant asks what a phrase means, do not offer an explanation; rather, suggest that he/she use their own interpretation of the question as best as they can. A suggested response is: "I know that it may be hard to think this way, but which of these categories is closest to what you are thinking?"
- If the participant asks what a specific word means, tell them you will look it up for them and provide them with a definition from a dictionary.
- If they are still unable to answer the question, make a note and initial/date.

© Boston Medical Center
Veterans Back to Health Trial

Survey Administration Procedures

What if the participant wants to know what their answers mean?

- Simply state, “I’m sorry, I am not trained to score or interpret the survey.”
- For eligibility screening, “The purpose of this survey is to determine your eligibility. I am entering your answers into a system that only tells me if you are eligible for the study. Study staff are not trained to interpret your answers.”
- If the participant continues to insist on receiving an interpretation of their answers, let them know you will discuss it with the PI and get back to them as soon as possible.

What if the participant refuses to complete a survey?

- Explain that completing the survey is part of participating in the study and is very important for our research. The survey information helps us learn the benefits of yoga and education to help treat back pain in the future.
- Explain that they survey responses are essential to getting a complete picture of the participant’s health.
- If the participant still refuses, thank them for their time and end the call.

What if the participant doesn’t answer all of the questions?

- Assess if he/she skipped the question(s) by mistake or intentionally.
- If the question(s) were skipped by mistake, ask them to please respond.
- If the participant is uncomfortable with some of the survey questions, tell him/her that it is not a problem and continue reviewing the survey.
- If a participant intentionally skipped a question but does not offer an explanation right away, ask why he/she had difficulty responding. Help the participant answer the question by slowly re-reading the question and answer choices *verbatim*. Do not rephrase or interpret meaning.
- If the participant is still unable to answer the question(s), accept the incomplete survey.

© Boston Medical Center
Veterans Back to Health Trial

Survey Signature Page

FOR STAFF ONLY

Survey Administration:

______________________________ ___________________________________ ______/_____/____
Printed Name Signature Date

Initial Data Entry:

______________________________ ___________________________________ ______/_____/____
Printed Name Signature Date

Double Data Entry:

______________________________ ___________________________________ ______/_____/____
Printed Name Signature Date

Data Audit:

______________________________ ___________________________________ ______/_____/____
Printed Name Signature Date

STAFF

© Boston Medical Center
Veterans Back to Health Trial

Data Collection Schedule

Measures	Baseline	6 weeks	12 weeks	24 weeks
**BASELINE INFORMATION**				
Sociodemographics*	✓			
Expectations and Preference	✓			
LBP History and Comorbidities	✓			
**PRIMARY OUTCOMES**				
Low Back Pain Intensity (DVPRS)	✓	✓	✓	✓
Back-Related Function (RMDQ)	✓	✓	✓	✓
**SECONDARY OUTCOMES**				
Pain Medication Use	✓	✓	✓	✓
PTSD CheckList—Civilian version (PCL-C)	✓	✓	✓	✓
Health-related Quality of Life (SF-12)	✓	✓	✓	✓
Satisfaction with Treatment	✓	✓	✓	✓
Global Improvement		✓	✓	✓
**COST EFFECTIVENESS OUTCOMES**				
Work Productivity	✓	✓	✓	✓
Medical Utilization	✓	✓	✓	✓
**EXPLORATORY OUTCOMES**				
LBP Treatments	✓	✓	✓	✓
PROMIS-29 Profile & Pain Interference	✓	✓	✓	✓
Depression (PHQ-9) & Anxiety (GAD-7)	✓	✓	✓	✓
Exercise History	✓	✓	✓	✓
Dyadic Adjustment Scale (DAS)	✓		✓	✓
Alcohol, Drugs, & Smoking	✓		✓	✓
Pain Self-Efficacy (PSEQ)	✓		✓	✓
Sleep Quality (PSQI)	✓		✓	✓
Coping Strategies (CSQ)	✓		✓	✓
Post-Concussive Symptoms (NSI)	✓		✓	✓
Adverse Events		✓	✓	✓

*Sociodemographic information includes gender, age, relationship status, ethnicity, race, income, education, housing, and military service history.

DVPRS = Defense and Veterans Pain Rating Scale; RMDQ = modified Roland Morris Disability Questionnaire; PROMIS = Patient Report Outcome Measurement Information System; SF-12 = Short Form 12-item Health Survey; PHQ-9 = 9-item Patient Health Questionnaire; GAD-7 = 7-item General Anxiety Disorder questionnaire; PSQI = Pittsburgh Sleep Quality Index; NSI = Neurobehavioral Symptom Inventory

© Boston Medical Center
Veterans Back to Health Trial

Honoraria Distribution

Participants will receive cash honoraria after the completion of each survey. Participants receive a total of $125 in honoraria if they complete all surveys. Participants will also receive $10 travel compensation in the form of gift cards for each visit with research staff (consent meetings and all surveys) and up to 12 yoga classes. If a subject withdraws from the study, they will not receive any future payments.

Bedford VA Cash Vouchers

Cash vouchers are created by VA research staff in the participant's VA medical record. Research staff will document handing out cash honoraria using the Honoraria Signature Form.[‡] After research staff have completed the necessary information in the medical record, the participant receives cash from the Bedford VA Cashier. A history of cash vouchers received/redeemed can be viewed in individual medical records.

Gift Cards

Gift cards will be ordered by BMC in $10 increments. Gift cards will be ordered well in advance (possibly a month ahead of time) to account for processing delays and avoid rush shipping charges. All gift card purchases will be approved by the Principal Investigator via signature on the BMC Gift Card Purchase Form. Both CVS and Target are able to provide a spreadsheet with all gift card numbers included in the order. BMC staff will request this spreadsheet upon activating gift cards and will send the spreadsheet to Bedford VA staff for tracking purposes.

All gift cards will be stored in a locked file cabinet in a locked office at the Bedford VA. Access to the gift cards will be restricted and monitored.

Gift cards will be given to participants to offset travel expenses associated with participation in the study. If any visits are combined into one trip to the Bedford VA Hospital, either as a result of the participant's or staff's preference/request, only one $10 gift card will be given to the individual for the trip. For example, if a participant completes their 6-week survey while on campus for their yoga class, only one travel gift card will be given for that visit.

After participants have completed their visit, they will sign the Gift Card Signature Log[‡] to confirm they have received a gift card. This log will also be initialed by the staff member that witnessed the participant sign. During survey administration, it is often most convenient for this to occur during final verification of survey completeness when the cash honoraria will also be given to the participant. Gift cards for travel reimbursement will not be given to participants that complete a survey over the telephone or via mail.

All Gift Card Signature Logs are kept in a binder in the study office that is organized and stored by the VA Project Manager. Information from these logs will also be kept electronically on a spreadsheet[§] containing the gift card number, the gift card amount, the name of the person it was given to, and the date it was handed out.

[‡]Per BMC's *Research Participant Compensation Policy (39.04.462)*, this log must contain the study participant name, compensation amount, and date issued at minimum. Gift card number should also be included for tracking.

[§]Per BMC's *Research Participant Compensation Policy (39.04.462)*, this spreadsheet log must contain the gift card amount, quantity, participant name, and date handed out.

© Boston Medical Center
Veterans Back to Health Trial

Randomization Procedure

After baseline data collection, participants will be randomized in a 1:1 ratio to yoga or education using a computer-generated blocked-stratified randomization with varying block sizes of 2 or 4. Stratification will be done on three dichotomous variables to assure similar distribution among the two groups:

1. Age (≤45, >45 years). Back pain in older Veterans may be different structurally and age may be a confounder to the response to yoga.
2. PTSD CheckList – Civilian Version (PCL-C) score (<30, ≥30). A score ≥30 is considered indicative of experiencing post-traumatic stress symptoms (PTSS). This will assure equivalent numbers of Veterans that experience PTSS in both treatment arms.
3. Partnered (yes/no). A partnered Veteran will be defined as one that reports having a spouse, cohabitating partner, or long-term committed partner. This will help assure adequate numbers of partnered Veterans in the yoga group for qualitative interviews.

Unblinded staff will notify participants of their group assignment and give take-home supplies after completion of the baseline survey. If this is not possible, participants will be notified within one week after the baseline visit and at least one week prior to the first yoga class.

Randomization will not occur prior to the collection of the baseline survey.

Responsibility

After scoring of the baseline PCL-C questionnaire, staff will enter the participant's age, partner status, and PCL-C score into StudyTRAX by clicking Edit Subject on the participant's page. The application will not allow randomization to be executed until these fields are complete.

The VA Project Manager will complete randomization using StudyTRAX. Another unblinded VA staff member may be granted this role if the Project Manager is unavailable. After filling out the stratification factors, randomization is completed by pressing the orange Randomize Subject button on the participant's dashboard and verifying the stratification data in the window that comes up. After the participant has been randomized, click the microscope logo next to the newly created Randomization ID will reveal the group assignment.

The Bedford Project Manager will create a password-protected spreadsheet with SIDs and group assignments. This spreadsheet will be stored on a secure network drive at the Bedford VA and will not be accessible by blinded research staff. The staff member that executes randomization will complete the Randomization and Enrollment Form for each randomized participant. This form does not contain specific group assignments and will be filed in individual participant files. Only unblinded staff will be granted access to view group assignments on StudyTRAX™.

© Boston Medical Center
Veterans Back to Health Trial

Randomization and Enrollment Form

SID: ____________________________

This form should be completed for all enrolled participants (i.e., signed the ICF) to confirm that all eligibility criteria are met and the baseline survey is completed prior to randomization.

1. ELIGIBILITY

Date Screened: ____/____/____

If screened more than 45 days before baseline survey…

Date of Eligibility Verification: ____/____/____

Does the participant satisfy all inclusion and exclusion criteria? ☐ Yes ☐ No

Have all baseline survey components been completed? ☐ Yes ☐ No

Is the participant still willing to proceed with the study? ☐ Yes ☐ No

If NO to any of the above, skip section 2 below

IF YES TO ALL ABOVE QUESTIONS…

2. BASELINE & RANDOMIZATION

Date of Baseline Survey: ____/____/____

Date of Randomization: ____/____/____

3. ONLY COMPLETE IF NOT RANDOMIZED

If not randomized please indicate reason:

☐ Failed eligibility verification ☐ Failed to return

☐ Declined participation ☐ Other: ______________________________

____

STAFF NAME **SIGNATURE** **DATE**

© Boston Medical Center
Veterans Back to Health Trial

Data Entry Procedures

Paper surveys will be entered into StudyTRAX by blinded research staff at BMC. The staff member that administered the survey cannot complete primary entry but can complete the secondary entry. Primary and secondary entry must be completed by two different blinded staff members.

Surveys completed over the telephone will be entered into StudyTRAX by research staff in real time. These surveys will not be double entered.

Primary Data Entry

1. Before preparing to enter a survey, make sure you did not administer it.
2. Login to StudyTRAX (my.studytrax.com). From the Project Dashboard, type the participant's SID in the *Reference ID* box on StudyTRAX and click GO.
3. Look at the SID in the top left corner and verify it is the same as the paper form.
4. Click the correct survey on the Dashboard or add it from the Encounters page.
5. Change the encounter date in top left corner to match date of survey administration.
6. Enter data ***exactly*** as it appears on the survey. For exceptions to this rule, see **Data Entry Troubleshooting** on the following page.
7. Save progress often. It is recommended that data entry is saved at the end of each StudyTRAX page before moving onto the next page.
8. Before finalizing data entry, make sure all sections are marked with a green circle. If any are yellow, they are incomplete. Go back to the section and check that everything is filled out.
9. After the survey is completely entered and saved, select Save and Return to go back to the Dashboard. Click on Edit Subject and locate the Data Entry Sign-in section. Write your initials (3 if possible) and the date you completed data entry in the appropriate fields.
10. Sign and date the paper Survey Signature Page attached to the survey.
11. Once completed, place survey in *For Double Data Entry* section of file cabinet.

Secondary Data Entry

1. Before preparing to do a secondary entry, make sure you did not complete the first entry.
2. On the StudyTRAX Project Dashboard, type the participant's SID in the *Reference ID* box on StudyTRAX and click GO. Look at the SID in the top left corner and verify it is the same as the paper form.
3. Go to the Encounters page. Click on "New" in the *Secondary Date* column to bring up the blank survey for double entry.
4. Change the encounter date in top left corner to match date of survey administration.
5. Enter data ***exactly*** as it appears on the survey. For exceptions to this rule, see **Data Entry Troubleshooting** on the following page.
6. Save progress often. It is recommended that data entry is saved at the end of each StudyTRAX page before moving onto the next page.
7. Before finalizing data entry, make sure all sections are marked with a green circle. If any are yellow, they are incomplete. Go back to the section and check that everything is filled out.

© Boston Medical Center
Veterans Back to Health Trial

8. After the survey is completely entered and saved, select Save and Return to go back to the Dashboard. Click on Edit Subject and locate the Data Entry Sign-in section. Write your initials (3 if possible) and the date you completed data entry in the appropriate fields.
9. Sign and date the paper Survey Signature Page attached to the survey.
10. Once double entry is complete, place survey in *For Reconciliation* section of file cabinet.

General Data Entry Tips

- Attempt to answer every field. Use the codes below if any answers are missing. Note that the code depends on whether or not the variable requires a numeric response or a text response.
- Review the table of common issues.
- SAVE often.
- If you make ANY notes on the paper survey, always put your initials and the date. If you cross out a previous entry, use a single line only.
- Record the SID and specific question of any responses that common issues listed below to ask the Project Manager and/or PI.
- Always sign the paper signature page and enter your initials in StudyTRAX after entry.

Missing Values	Numeric	Text
Not Applicable/Doesn't Apply	-99	N/A
Don't Know or Left Blank	-88	DK/REF

Data Entry Troubleshooting

The below table shows some common data entry issues.

**If…**	**Then…**
The participant writes down a pain medication but spells it incorrectly.	Look up the correct spelling. Enter the correct spelling and make a note on the paper survey with initials and date.
The participant answers a question with a number range.	Enter the average of the two numbers. E.g., 2-4 should be entered as '3'. Make note on the paper survey with initials and date.
A question is left blank.	Enter **DK/REF** (multiple choice or text response) or **-88** (numerical response).
Two answers are given for a multiple choice or yes/no question.	If *adjacent* answers are marked on a multiple choice question, the BMC Project Manager will use a random number generator to select an answer.<br><br>If the answers are *non-adjacent* or the answer choices are

© Boston Medical Center
Veterans Back to Health Trial

	yes/no, the question should be treated as blank. Make note of what was entered on the paper survey with initials and date.
A numerical answer is given that is out of the range of expected answers.	Treat the response as blank. E.g., the participant wrote that they sleep 30 hours each night (exceeds # of hours in a day). Make note on the paper survey that the answer is out of range, what was answered, your initials, and the date.
On a set of questions that contain an “Other” option (e.g., pain medications, LBP treatments), the participant left the text and checkboxes empty.	Follow the trend of the preceding questions. If the participant checked off the answers that apply to them but left others blank, all should be entered as **DK/REF**. If the questions are all answered with a **Yes** or **No**, enter **No** for the “Other” options. During data analysis, No and DK/REF responses for these “Other” questions are considered equivalent. This is not necessarily true for questions elsewhere in the survey, so *don’t apply this rule to other questions*.
For a question that asks the participant to specify an “other” response, the participant wrote down a text answer but did not check off “Yes” or “Other.”	Check **Yes** in StudyTRAX and enter the text. In StudyTRAX, if No or DK/REF are selected, an answer cannot be entered in the text box and the answer will be lost. Make note of what was entered on the paper survey with initials and date.
For a question that asks the participant to specify an “other” response, the participant checked “Yes” or “Other” but did not write an answer.	Check **Yes** or **Other** in StudyTRAX (depending on the question) and enter ‘**DK/REF**’ in the text box. Make note of what was entered on the paper survey with initials and date.
A question asks for a date in MM/YY format but the participant did not write down the full date.	Enter the unknown part of the date as ‘**DK**’. E.g., if the participant only wrote ‘1990’ and StudyTRAX requires the MM/YY format, enter as ‘**DK**/90’. For date of enrollment in VA healthcare, if the entire date is missing, enter ‘**-88**’ per the instructions on StudyTRAX.<br><br>If the answer should be a range (e.g., dates of military service or deployment in the format MM/YY-MM/YY) and the start or stop date is unknown, enter it as ‘**DK**’ (*not* ‘DK/DK’). If there is no stop date, i.e., the participant is still on duty of some type, enter the stop date as ‘**NA**’.
There is an ambiguous or confusing response on a paper survey that is not explicitly described above.	Ask the BMC Project Manager how the question should be entered. He/she will review the survey and make a decision or will defer the issue to the PI. Make note of what was entered on the paper survey with initials and date.

© Boston Medical Center
Veterans Back to Health Trial

Questionnaire-Specific Issues

Most questionnaires in StudyTRAX are set up in a ‘matrix’ format (i.e., all or part of VR-12, PROMIS, PCL-C, GAD-7, PHQ-9, CSQ, DAS, PSEQ, PSQI, and NSI) that may look slightly different than the paper survey due to the addition of the DK/REF column on the right. A StudyTRAX matrix looks like:

	0	1	2	3	4	5	6	DK/REF
I think of things I enjoy doing.								
I just think of it as some other sensation, such as numbness.								
I feel terrible and I feel it is never going to get any better.								
I don't pay attention to it.								

When the DK/REF option is added to the table in StudyTRAX but is not present on the paper survey, the answers may line up differently in relation to the right side of the table. Looking at the matrix above, for example, ‘5’ is the third button from the left, but on paper, ‘5’ would be the second choice from the left. Staff should take time to make sure the responses entered into StudyTRAX are correct and should not depend on how the responses line up with the right side of the page.

In the Pittsburgh Sleep Quality Index (PSQI), question 5J allows the participant to specify a reason not previously listed that causes trouble sleeping. There are a few things to keep in mind when entering this question that affect how the questionnaire is scored for analysis:

- If the participant does not write down an “other reason” but marks a frequency in the second part of the question, select the frequency that they chose and enter **DK/REF** in the text box that comes up.
- If the participant writes down a reason that is already mentioned elsewhere in question 5 (i.e., bad dreams, pain, too cold, too hot, etc.), check to see if the frequency is the same as the first time the reason is mentioned. If the frequency is the same, enter 5J as if the response is ‘none.’ If the frequencies are different, enter **DK/REF** for 5J.

© Boston Medical Center
Veterans Back to Health Trial

StudyTRAX Guidelines

StudyTRAX is a web-based data management system used to capture study data. Research staff can login to this system at www.my.studytrax.com using the username and password provided. This guide is meant to serve as an introduction to StudyTRAX usage for research staff. The full program User's Guide is available online at http://wiki.studytrax.com/display/STX/Users+Guide.

General tips for using StudyTRAX:

DO

- Use the navigation buttons within the application ONLY (i.e., use "Cancel" or "Save and Return" to return to the previous page)
- Allow a page to completely load before clicking again
- Single-click
- Click logout before closing the browser window
- If you are logged out due to inactivity, close and re-open the browser to clear the cache
- Frequently click on Save (there is no auto-save)

DON'T

- Use the browser's Back button (use the Back button in the StudyTRAX toolbar)
- Double-click
- Forget to log out before closing the browser
- Use Ctrl+N or another tab to open another session of StudyTRAX in the same browser

Once logged in, make sure that the correct project is listed in the top left corner of the screen. To change projects, select the correct project from the drop-down *Project Menu*. The "Veterans Back to Health" project contains all survey data. The "VBH Unblinded Data" project contains data that is only accessible by unblinded staff, i.e., home practice logs, education check-in data, and adverse events.

In order to access a specific participant's profile within StudyTRAX, enter the corresponding SID into the *Reference ID* box under the Quick Search section then select GO. Veterans Back to Health screening IDs are each three numbers.

Once a participant's profile is open, the Dashboard screen will show the participant's Timeline and Data Collection Summary to date. This is color-coded so that green means "complete," yellow means "incomplete," and red means "unknown."

The menu on the left shows the other aspects of the profile:

- Attributes contains enrollment progress notes, data entry sign-in, and discontinuation information, if applicable. Subject attributes are viewed by clicking Attributes and are edited by clicking Edit Subject to the right of the *Project Menu*.

- Tasks allow users to see pending tasks (e.g., eligibility follow-up) for the participant. If a task does exist, the user can see the title of the task, who the task is assigned to, a due date, and priority level.

- The Encounters page shows all of the data collection encounters that have been completed and entered for this participant. Unlike the Dashboard, this page shows both primary and secondary data entry information. The date of survey completion is in the

© Boston Medical Center
Veterans Back to Health Trial

Date column and the *Secondary Date* column indicates the status of double entry for the encounter. If the survey date is present under the *Secondary Date* column, the survey has been double entered. The absence of a date under the *Secondary Date* column for *Screening* and *In-Person Visit* indicates that double entry is not possible for these intervals.

- The right side of the Encounters page has the list of intervals that can be added for the participant. When appropriate, the correct encounter (e.g., in-person meeting, survey, etc.) can be added by clicking the icon next to the interval name. Intervals can usually also be added from the Dashboard page by clicking on the Timeline.

Once you select an interval from Encounters or from the Dashboard, the Encounters Update page will come up. On this page:

- The Date of the survey is shown in the top left corner. The default value will be today's date. The date should be changed to reflect when the participant completed the survey.
- The Table of Contents under the date box shows the sections of the survey. The page numbers are to the right of the section titles. The current page will be indicated by bolded page numbers. Sections marked with are complete and sections marked with are incomplete. A ? indicates that the completion status of that section is unknown. Click on the next survey section listed in blue to advance to the next page in StudyTRAX. **NOTE: Page numbers in StudyTRAX do not correspond to page numbers on paper surveys.**
- The Save and Return button on the top left will save all entered data and return to the last page open before entering the survey. If the Save and Return and Save buttons are bolded, changes have been made on the page since the last save.

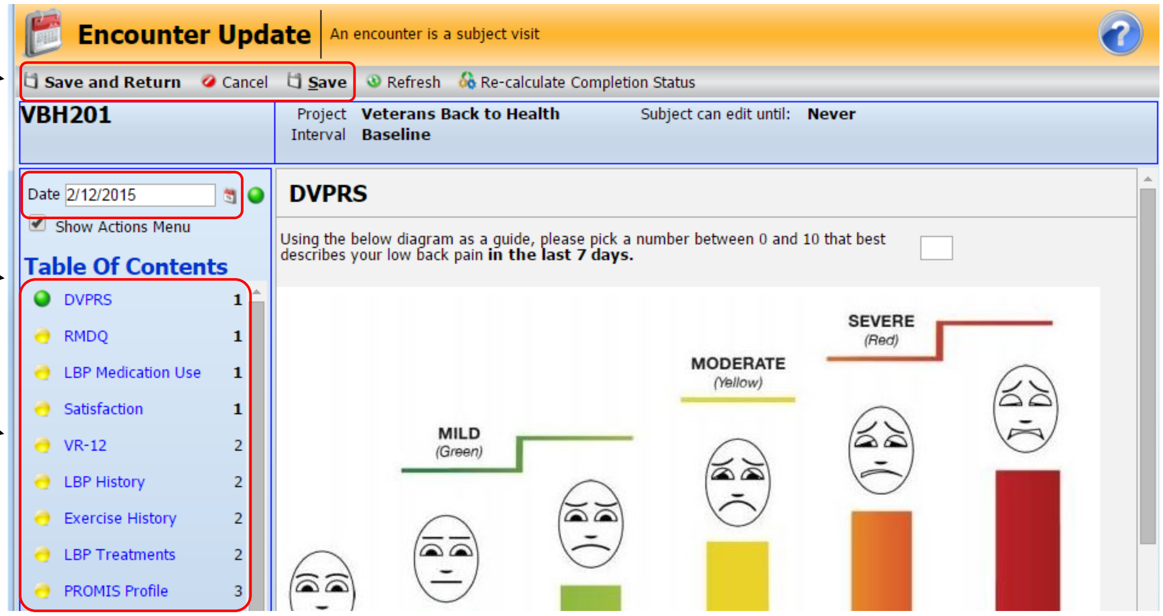

© Boston Medical Center
Veterans Back to Health Trial

The VBH Unblinded Data project is only accessible by study staff that are granted access to participants' treatment assignments. Blinded staff members are not able to access this project. Access to projects and specific information within projects in StudyTRAX can be granted and changed by the BMC Project Manager.

Adding Participants into the StudyTRAX Unblinded Project

Participants are added to the main Veterans Back to Health project when they are screened but they will not automatically be added to the VBH Unblinded Data project. They will need to be added to this project after randomization by an unblinded staff member. To enroll participants into the unblinded project:

1. Login to StudyTRAX and select VBH Unblinded Data from the dropdown menu at top left corner. If it is not listed, access must first be granted by the BMC Project Manager.
2. Click New Subject next to the dropdown menu.
3. Enter the SID into the box labeled Reference ID then click Save and Return.
4. Click Enroll Subject.
5. You should now be at the Dashboard, where you will see the Timeline. Click Edit Subject.
6. Select the appropriate treatment from the Treatment Arm dropdown menu then click Save and Return. The participant is now enrolled in the unblinded project.

© Boston Medical Center
Veterans Back to Health Trial

Data Management Procedures

Survey forms will be stored in a locked file cabinet at the Bedford VA immediately after collection. Surveys will then be securely delivered to BMC for double data entry into StudyTRAX™, an electronic data capture system where each user is assigned a unique user ID and password with restricted access. Access is regulated by the BMC Project Manager.

All survey forms that contain SIDs will not have any identifiable participant information on them. If staff notice that any identifiable information is present on survey forms, they should redact the information permanently. All redactions should include the initials of the person making the redaction, the date of the change, and an explanation of the marking. Paper surveys will be filed in individual participant files and will be kept in accordance with the Veterans Health Administration (VHA) Records Control Schedule.

Data will be periodically exported into Excel for analysis purposes. These documents will not contain names and will be password-protected on a secure network drive at BMC or the VA.

TRACKING DATA COLLECTION AND ENTRY

An Excel document is kept to track the receipt and entry of all surveys at BMC. This information is also stored in StudyTRAX. These spreadsheets will document the date of survey collection, who completed primary entry, who completed secondary entry, and any relevant notes about the survey. This document should be updated by BMC staff responsible for overseeing data entry and management.

PRIMARY DATA ENTRY

- Paper surveys will be entered by blinded research staff at BMC. The blinded staff member that completes primary data entry cannot be the same person that administered the survey.
- All efforts will be made to enter data within a week of delivery to BMC.
- Surveys that need to be entered are in the *For Data Entry* section of the DATA cabinet.
- Primary entry will be documented in two ways:
    1. Name, signature, and date on the paper Survey Signature Page.
    2. Initials and date in StudyTRAX Data Entry Sign-in.
- Once primary entry is complete, surveys are filed in the *For Secondary Entry* section of the DATA cabinet.

SECONDARY DATA ENTRY

- All paper surveys will be double entered by blinded research staff.[5]
- Surveys that have been entered once but need to be entered a second time will be located in the *For Secondary Entry* section of the DATA cabinet.
- Secondary entry will be documented in two ways:

[5] Staff can enter data for one round of data entry (primary OR secondary) for each paper survey. Unlike the primary entry, the secondary entry can be completed by the staff member that administered the survey.

© Boston Medical Center
Veterans Back to Health Trial

    1. Name, signature, and date on the paper Survey Signature Page.
    2. Initials and date in StudyTRAX Data Entry Sign-in.
- Once secondary entry is complete, surveys are filed in the *For Reconciliation* section of the DATA cabinet.

RECONCILING PRIMARY AND SECONDARY ENTRIES

StudyTRAX produces a Double Data Entry Delta Report that shows all differences between primary and secondary survey entries. This report can be found under the Reports tab on the top of the screen for those that are granted access to reports. The BMC Project Manager will reconcile entries after all surveys have been double entered.

1. Under the Reports tab, click on Double Data Entry Delta Report (DO NOT include Secondary Encounters that Have Not Been Entered). Click Submit and print the report.
2. Review the report for discrepancies between primary and secondary entries. The SID is in bold (e.g., 117, 202) above the date and name of the survey interval (e.g., 4/20/15 - Baseline). Under the name of the survey is the name of the section in bold (e.g., Sociodemographics, LBP Medication Use) and two columns for the primary and the secondary entries. The StudyTRAX variable name is displayed for each discrepancy (e.g., AF_Active, Other med1, etc).

**117**		
4/20/2015 - Baseline		
**Sociodemographics**	**Primary**	**Secondary**
AF_Active		12/88-00/00
**202**		
4/21/2015 - Baseline		
**LBP Medication Use**	**Primary**	**Secondary**
Other med1	0	-88
Other med2	0	-88

3. If there are discrepancies between entries, find the participant's paper survey that corresponds to the report.
4. Find the question that is on the report, review the participant's answer, and circle the correct entry on the paper report.
    - If neither entry is correct, write the correct answer to the left of the secondary column.
    - If the participant's entry is not clear, review the survey with the PI.
5. On StudyTRAX, navigate to the participant's Encounters page. Revise the primary and/or secondary entries as appropriate. After saving the page, StudyTRAX will ask for a change reason. Enter 'Data clean.'
6. After all discrepancies have been fixed in StudyTRAX, re-run the Double Data Entry Delta Report to make sure everything was correctly fixed. If there are still discrepancies listed on the report, repeat steps 2-5.
7. Return the hard-copy of the survey to the *To Be Filed* section of the DATA file cabinet.
8. On the first page of the paper report(s), write 'Entered' followed by your initials and the date you made the corrections. File the report(s) in the Double Data Entry Folder.

© Boston Medical Center
Veterans Back to Health Trial

Data Management Procedures

1. Newly received paper forms immediately filed in "For Data Entry" section of locked DATA file cabinet

2. Initial entry of paper forms into StudyTrax™ by blinded research staff completed

3. Forms filed in the "For Secondary Entry" section of locked DATA file cabinet

4. Second entry of paper forms into StudyTrax™ by different blinded research staff completed

5. Forms filed in the "For Reconciliation" section of locked DATA file cabinet

6. StudyTRAX™ Double Data Entry Report is produced, entries are reconciled using paper surveys, and forms are moved to "To Be Filed" section of DATA cabinet

7. Forms filed in individual participant folders in locked cabinets labeled "Participant Files"

© Boston Medical Center
Veterans Back to Health Trial

Study Completion Form

Study ID#: ________________________________

Date of Study Completion

Date: ____/____/____

Primary reason for terminating participation in the study:

- ☐ Completed study
- ☐ Participant voluntarily withdrew from study
- ☐ Participant was determined after enrollment to be ineligible*
- ☐ Participant withdrew consent
- ☐ The PI determined it was not in the participant's best interest to continue*
- ☐ Other*

*Comments: ____

____

____

Date of Last Survey

Date: ____/____/____

Last survey completed:

- ☐ Baseline
- ☐ 6 Weeks
- ☐ 12 Weeks
- ☐ 24 Weeks

Date of Last Known Treatment Session

Date: ____/____/____

☐ Participant did not attend any treatment sessions

Name of Research Staff: ____

Signature of Research Staff: ____

© Boston Medical Center
Veterans Back to Health Trial

Adverse Event Procedures

Veterans Back to Health

© Boston Medical Center
Veterans Back to Health Trial

Adverse Events Overview

The Data and Safety Monitoring Plan (DSMP) for this study provides more detailed information about policies and procedures for protecting confidentiality, managing risks, and monitoring safety.

An **Adverse Event (AE)** is defined as any untoward medical occurrence in a subject temporally associated with participation in the clinical study. An adverse finding can include a sign, symptom, or abnormal assessment (e.g., laboratory test value) or any combination of these. AEs will be classified according to seriousness, severity, relatedness, and expectedness.

A **Serious Adverse Event (SAE)** is one that results in one or more of the following: death, a life-threatening event, inpatient hospitalization or prolongation of existing hospitalization, a persistent or significant disability/incapacity, a congenital anomaly or birth defect, or an important medical event based upon appropriate medical judgment.

An AE that is an **Unanticipated Problem (UP)** is one that meets all three of the following criteria:

1. Is *unexpected* (in terms of the nature, severity, or frequency), i.e., is not listed in the DSMP as an expected risk
2. Is *possibly* or *definitely related* to participation in the research
3. Is by definition *serious* OR suggests *increased risk* of participation than previously known

© Boston Medical Center
Veterans Back to Health Trial

Documentation and Reporting

COLLECTING AND REVIEWING AES

The same strategy for collecting AE data is implemented for both study arms. AEs will be solicited through the following:

1. Participants are instructed at enrollment to contact the study staff immediately if they experience any AE during the course of their involvement in the study. All participants have 24-hour emergency contact information for the VA Site PI and a member of the research staff.
2. All post-baseline surveys include questions on whether the participant has experienced any changes in his/her health status. Unblinded research staff and the Site PI, as necessary, follow-up on all these reports.
3. During education check-in calls, research staff will ask if the participant has experienced any health status changes during the study.
4. Yoga teachers will be instructed to contact staff immediately if any potential AE occurs during a yoga class or if a participant indicates at any time that he/she has had a change in health status.

Unblinded research staff at the Bedford VA will be responsible for AE tracking, preparing reports for Data Safety Monitoring Board (DSMB) meetings, and preparing summary reports for annual reporting to IRBs. All new AEs are reviewed at weekly research team meetings. Follow-up to previous AEs is also discussed at weekly team meetings as needed.

AEs will be classified according to the following:

- Seriousness – If the AE meets the definition of a Serious Adverse Event, it will be considered serious.
- Severity - based on the impact on participant's daily function. An AE will be termed 'mild' if it does not have a major impact, 'moderate' if it causes some functional limitation but does not impair activities of daily life (ADLs) and 'severe' if it causes a substantial disruption to function necessitating assistance with ADLs.
- Relatedness – based on how likely the AE is to be related to the study intervention. Specifically, they will be labeled either definitely, possibly or not related to the study intervention.
- Expectedness – possible and expected risks of the interventions are listed in the DSMP. All others will be classified as unexpected.

DOCUMENTATION

A master spreadsheet will be kept to document all AEs. This spreadsheet will contain the following information:

- Randomization ID/Screen ID
- Treatment Group
- Date AE Occurred
- Classifications (expectedness, seriousness, relatedness, severity)
- Brief Description
- If the AE caused the Veteran to stop the intervention, either temporarily or permanently

© Boston Medical Center
Veterans Back to Health Trial

- Further notes about the nature, timeline, and outcome of the AE that support the classifications

The staff member that becomes aware of the AE should contact the PI immediately if the AE is potentially serious and/or for further discussion about the nature of the AE. All AEs, regardless of the need for follow-up, should be reviewed at weekly team meetings to assure all reports are handled properly.

REPORTING

AEs that are unexpected, serious, and possibly or definitely related to the study intervention are considered Unanticipated Problems (UPs) and will be promptly reported to the DSMB, IRBs (Bedford VA and BUMC), and NCCIH within two business days of the staff becoming aware of the occurrence. The Bedford VA IRB requires UPs to be reported within five business days.

The Bedford VA IRB has additional reporting requirements for SAEs. Unexpected Serious Adverse Events (SAEs), regardless of relatedness, will be reported to the VA IRB using the VA *Report of Serious Adverse Event (SAE) and/or Unanticipated Problem (UAP) Involving Risks to Participants or Others* form within 5 business days of staff becoming aware of the event. All other non-serious AEs will be reported to the VA IRB in summary format during annual reviews.

All AEs that are not UPs will be reported annually in summary format to the BUMC IRB and to NCCIH.

© Boston Medical Center
Veterans Back to Health Trial

Procedure Diagrams

ADVERSE EVENTS VS. UNANTICIPATED PROBLEMS

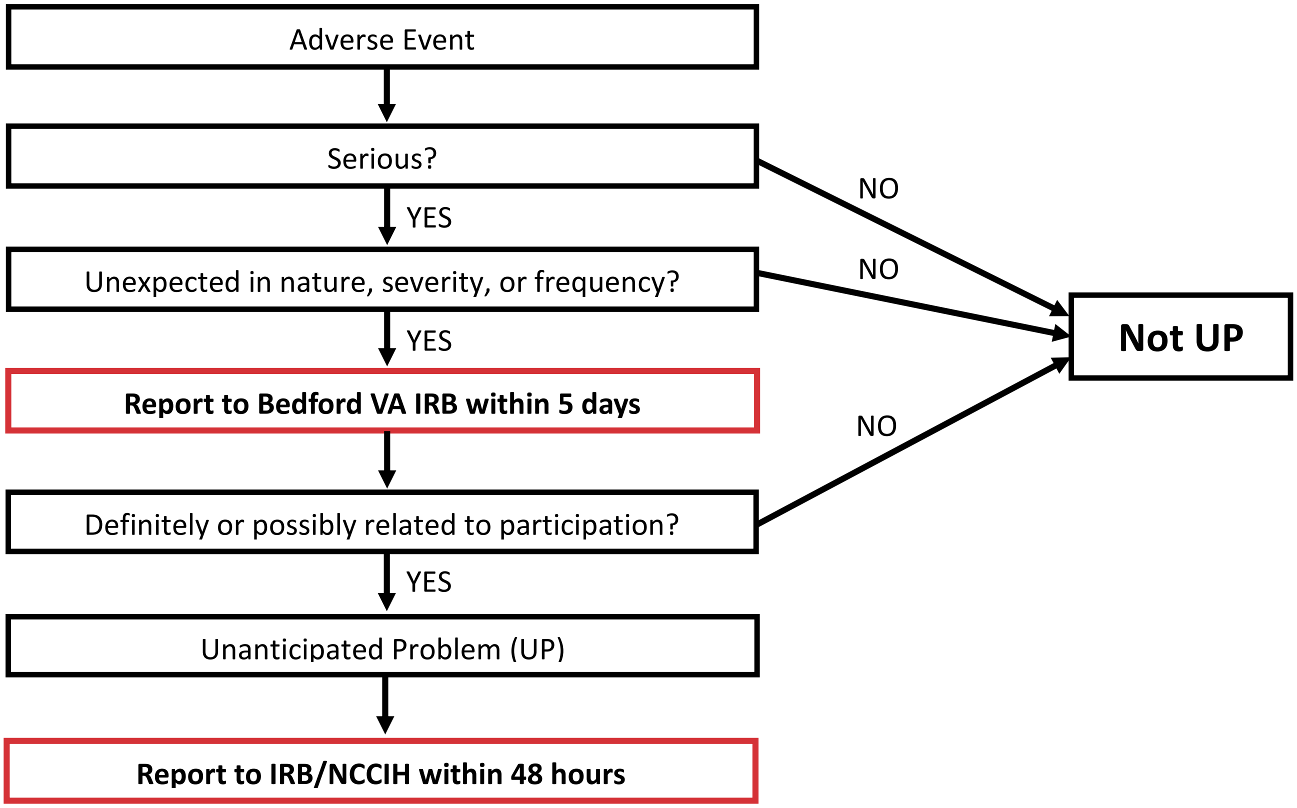

UPs that are not AEs (e.g., confidentiality breach) should also be reported to the IRBs/NCCIH within 48 hours.

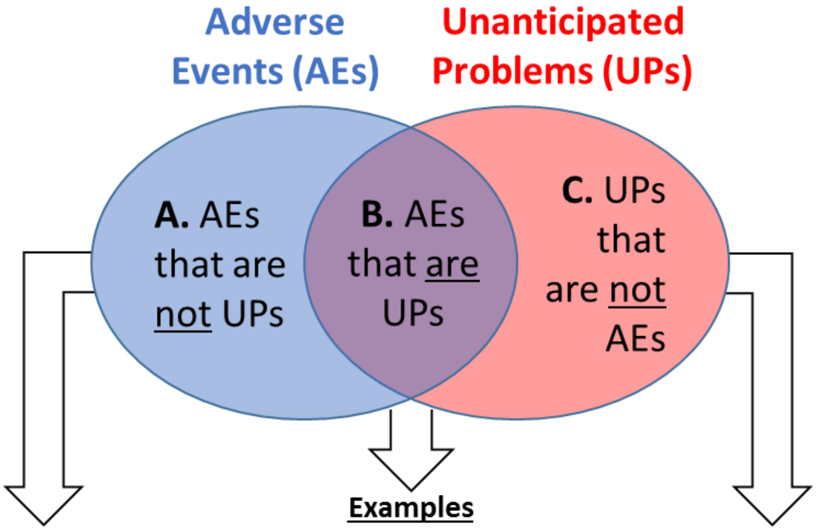

A. For example, muscle soreness, dizziness, nausea (listed in DSMP)

B. Inpatient hospital stay for pinched nerve (serious, unexpected, possibly related)

C. Breach of confidentiality (social/economic harm instead of physical harm)

© Boston Medical Center
Veterans Back to Health Trial

Procedure Diagrams

<u>AE COLLECTION AND REPORTING FLOW DIAGRAM</u>

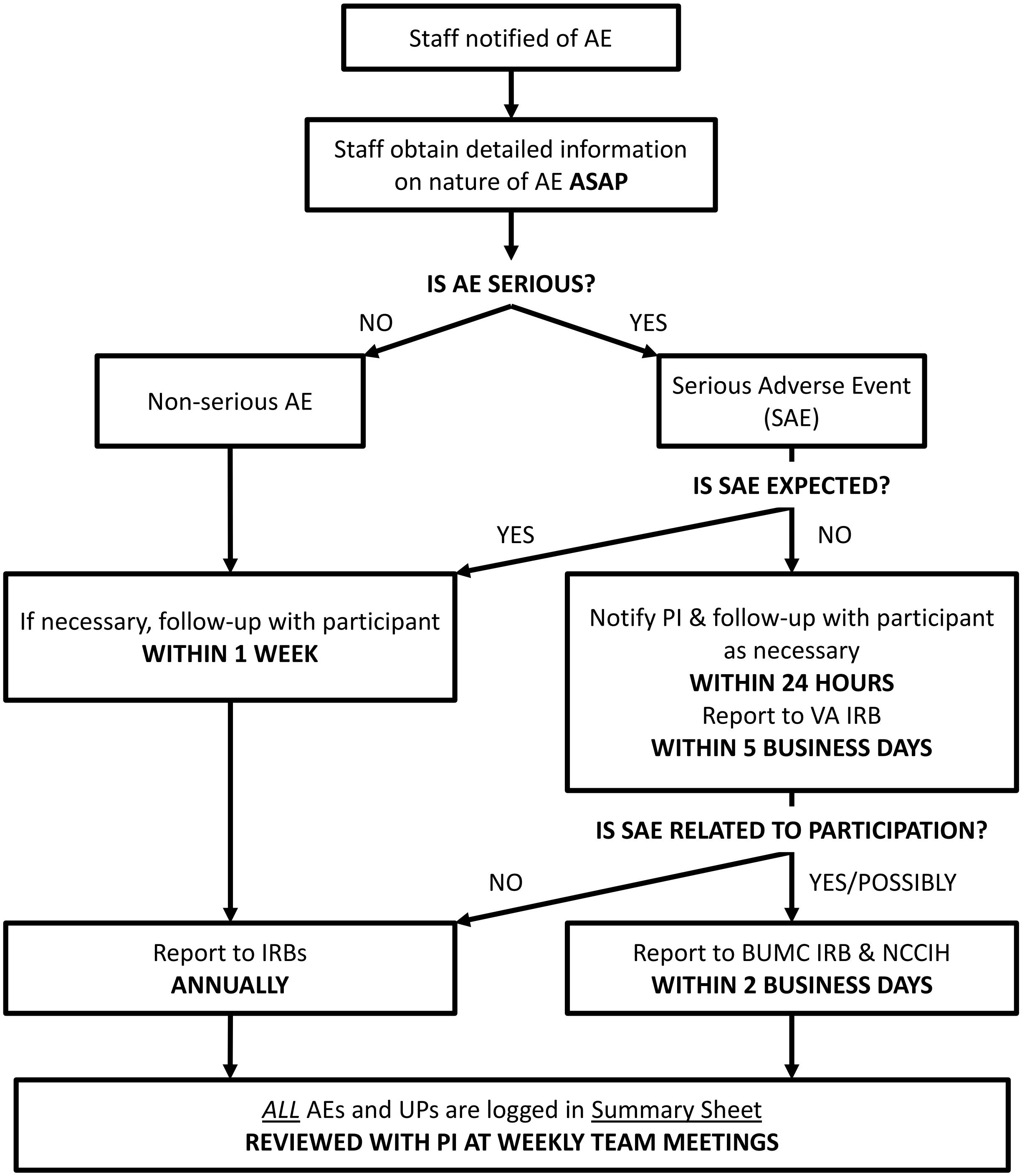

© Boston Medical Center
Veterans Back to Health Trial

Data and Safety Monitoring

The DSMB will review study progress and AEs on a semi-annual basis. DSMB meeting reports will be filed with the IRBs and funder as required.

The DSMB will be given a summary document of all AEs that have occurred since the start of the study or the last DSMB meeting. They will not be given any identifiable information about participants.

The AE Summary document will be in table format with the following headings:

Veterans Back to Health Adverse Event Summary [Start Date-End Date]									
SID	Date Occurred	Serious?	Severity (Functional Impact)	Expected?	Related?	Description	Outcome	Stop Intervention?	Notes

The document will have separate tables for each treatment arm. Each summary table will have AEs in order by relatedness (definitely related → possibly related → not related) then sequentially by SID so the board can review related AEs first and can identify if any participants experienced multiple AEs for the specified time period. The columns should be filled out in a consistent manner:

- **Randomization ID**: Participant's 4-digit randomization identification number
- **SID**: Participant's 3-digit screening identification number
- **Date Occurred**: Date AE occurred
- **Serious?**: Yes or No
- **Severity**: Mild; Moderate; or Severe
- **Expected?**: Yes or No
- **Related?**: Definitely Related; Possibly Related; Not Related
- **Description**: A couple words to describe the sign, symptom, or abnormal assessment reported. The cause of the symptom would not be written here. For example, "increased back pain" or "knee pain" would be correct but "car accident" or "hospital visit" would not.
- **Stop Intervention?**: Yes, permanently; Yes, temporarily; No
- **Notes**: A few lines that contain additional relevant information about the AE that support the classifications made in other columns, such as the cause of the symptom, a diagnosis given, details of severity, timeline of reporting/resolution (if applicable), and/or any action taken by research team. All of this information may not need to be written in this box for every AE.

After the board reviews and discussed all AEs, they will convey verbally to the PI their findings. A final report will be prepared and signed by the DSMB Chair. DSMB meeting reports will include the reviewed AE Summary document. In addition, the reports will address:

1. Whether adverse event rates are consistent with pre-study assumptions;
2. Reason for dropouts from the study;
3. Whether all randomized participants met entry criteria;
4. Whether continuation of the study is justified on the basis that additional data are needed to accomplish the stated aims of the study; AND
5. Conditions whereby the study might be terminated prematurely, if applicable.

© Boston Medical Center
Veterans Back to Health Trial
